# Active site coupling in *Plasmodium falciparum* GMP synthetase is triggered by domain rotation

**DOI:** 10.1038/ncomms9930

**Published:** 2015-11-23

**Authors:** Lionel Ballut, Sébastien Violot, Santosh Shivakumaraswamy, Lakshmi Prasoona Thota, Manu Sathya, Jyothirmai Kunala, Bauke W. Dijkstra, Raphaël Terreux, Richard Haser, Hemalatha Balaram, Nushin Aghajari

**Affiliations:** 1 BioCrystallography and Structural Biology of Therapeutic Targets Group, Molecular and Structural Bases of Infectious Systems, UMR5086 CNRS-University of Lyon 1, 7 passage du Vercors, 69367 Lyon Cedex 07, France; 2 Molecular Biology and Genetics Unit, Jawaharlal Nehru Centre for Advanced Scientific Research, Jakkur, Bangalore 560064, India; 3 Laboratory of Biophysical Chemistry, University of Groningen, Nijenborgh 7, 9747 AG Groningen, The Netherlands; 4 Bioinformatics: Structures and Interactions Group, Molecular and Structural Bases of Infectious Systems, UMR5086 CNRS-University of Lyon 1, 7 passage du Vercors, 69367 Lyon Cedex 07, France

## Abstract

GMP synthetase (GMPS), a key enzyme in the purine biosynthetic pathway performs catalysis through a coordinated process across two catalytic pockets for which the mechanism remains unclear. Crystal structures of *Plasmodium falciparum* GMPS in conjunction with mutational and enzyme kinetic studies reported here provide evidence that an 85° rotation of the GATase domain is required for ammonia channelling and thus for the catalytic activity of this two-domain enzyme. We suggest that conformational changes in helix 371–375 holding catalytic residues and in loop 376–401 along the rotation trajectory trigger the different steps of catalysis, and establish the central role of Glu374 in allostery and inter-domain crosstalk. These studies reveal the mechanism of domain rotation and inter-domain communication, providing a molecular framework for the function of all single polypeptide GMPSs and form a solid basis for rational drug design targeting this therapeutically important enzyme.

## Abstract (web summary)

GMP synthetase, a key enzyme in purine biosynthesis, is of interest for understanding purine metabolism processes and for developing therapeutic applications. Here, the authors propose a molecular mechanism and the structural basis for the catalytic activity of this enzyme.

## Graphical abstract

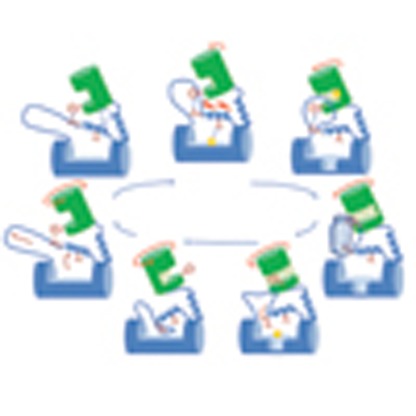

The parasite *Plasmodium falciparum* has evolved a unique set of biochemical pathways to adapt to specific milieus in which it resides. Metabolic pathways indispensable for parasite survival constitute obvious targets for the development of new anti-malarials. Although the human host has both the *de novo* and salvage pathways for purine nucleotide synthesis, the salvage pathway is the sole source of purine nucleotides to the rapidly multiplying parasite^1^,^2^,^3^.

During the parasite's intra-erythrocytic stages, adenosine and hypoxanthine salvaged from the human host serve as precursors for synthesizing inosine monophosphate (IMP), which serves as precursor for the synthesis of both adenosine monophosphate (AMP) and guanosine monophosphate (GMP). GMP synthesis proceeds through two steps, the first being the conversion of IMP to xanthine monophosphate (XMP) catalysed by IMP dehydrogenase followed by GMPS (guanosine 5′-monophosphate synthetase; EC 6.3.5.2) converting XMP to GMP^4^. GMPS is a class-I (trpG-type) amidotransferase in which the catalytic reaction occurs in two physically distant domains, a GATase (glutamine amidotransferase) domain and an ATPPase (ATP pyrophosphatase) domain^5^. Hydrolysis of glutamine to glutamate and ammonia occurs in the GATase domain, whereas formation of the intermediate AMP-XMP from ATP and XMP occurs in the ATPPase domain. Ammonia generated in the GATase domain attacks the intermediate to generate GMP (Supplementary Fig. 1). Coordination of activity across the two domains is believed to occur through channelling of ammonia from GATase to the effector domain, via a yet unknown mechanism^6^.

A tight functional coordination of the two active sites exists as binding of substrates to the ATPPase domain enhances the GATase activity, indicating that an obligate structural reorganization is required for coordinated catalysis^6^,^7^,^8^,^9^. The two catalytic domains of GMPS are either fused and encoded by a single gene as in *Plasmodium falciparum* and most prokaryotic and eukaryotic organisms or encoded by two separate genes as in many archaea^10^. In the ‘two-domain' type family, the GATase domain is connected via its C-terminus to the ATPPase domain^11^. Available ‘two-domain' type GMPS structures *Ec*GMPS (*Escherichia coli* GMPS, PDB-ID 1GPM^11^), *Tt*GMPS (*Thermus thermophilus* GMPS, PDB-ID 2YWB) and *Hs*GMPS (*Homo sapiens* GMPS, PDB-ID 2VXO^12^) do not give any indications of inter-domain communication. Neither the available AMP- and XMP-bound enzyme structures harbour structural features reflecting ligand-mediated inter-domain crosstalk. As no clear channel for ammonia transfer emerges from these structures, a drastic structural reorientation could be expected in a fully liganded enzyme. This view was recently suggested by biochemical studies of *Ec*GMPS^13^.

Aiming at unravelling the two-domain cross-talk mechanism, we determined the three-dimensional structures of the malaria relevant *P. falciparum* GMPS (*Pf*GMPS), of a glutamine-bound inactive mutant (*Pf*GMPS_C89A/Gln) and of the isolated GATase domain (*Pf*GATase). The glutamine-bound C89A mutant is the first GMPS structure harbouring an original conformation, in which the GATase domain (1–236) has undergone an important reorientation as compared with that seen for the native enzyme. This glutamine-bound structure corresponds to a pre-catalytic state which to the best of our knowledge has never been observed before for this enzyme family. Our studies reveal the conformational dynamics along the reaction pathway, and demonstrate how an 85° rotation and 3 Å translation along this rotation axis (going through residues Ile29 and Lys104) of the GATase domain contributes to the reorientation of the flexible, catalytic loop (residues 376–401). Comparative studies of our structures, coupled with site-directed mutagenesis disclose the mechanism of inter-domain communication and structural aspects triggering domain rotation. The invariant nature of the residues mediating these processes suggests the universality of the mechanism across all GMPS and provides a platform for future investigations on this therapeutically important enzyme.

## Results

### GMPS architecture

We purified and crystallized *Pf*GMPS, *Pf*GMPS_C89A and *Pf*GATase, and solved their structures by molecular replacement to resolutions of 3.6, 3.15 and 2.6 Å, respectively (Table 1). A 2.72 Å resolution crystal structure of *Pf*GMPS/XMP is available in the Protein Databank (PDB-ID 3UOW—unpublished).

**Figure 1:**
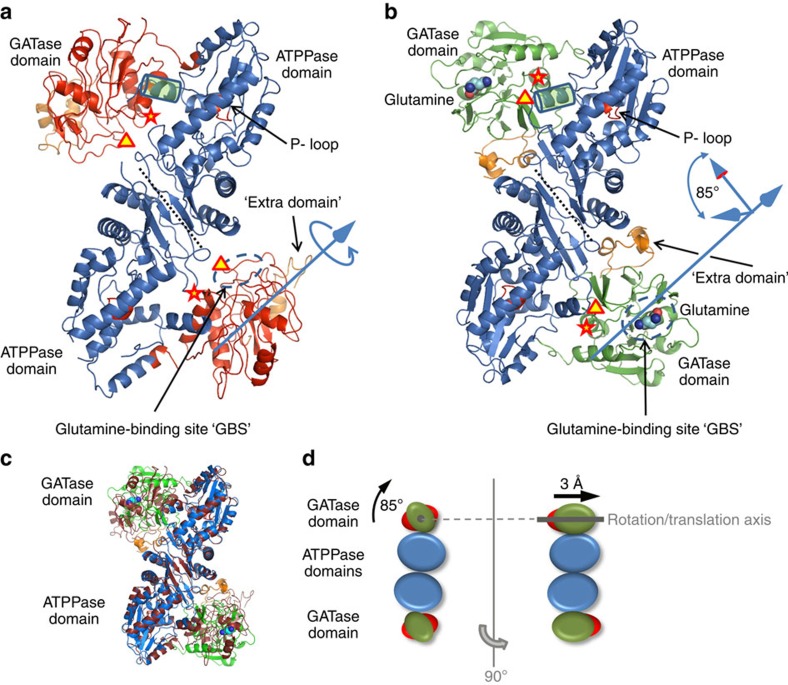
Crystal structures of *Pf*GMPS and glutamine bound *Pf*GMPS_C89A. Monomers are composed of two catalytic domains, an N-terminal GATase domain (1–236, in red wild type (**a**) and in green mutant (**b**)), and a C-terminal ATPPase domain (237–555, blue). The GATase segment forms an independent domain (α/β-structure of nine β-strands surrounded by five α-helixes) and exhibits a unique extension (119–151, orange) inserted between β5 and β6. Tyr212 is labelled as a triangle, Cys113 as a star. Glutamine (substrate) is represented by spheres (**b**) and the dashed line shows the glutamine-binding site (**a**,**b**). The GATase domain is connected through a 13-residue linker (228–240) to the ATPPase domain. This latter is composed of a five-stranded parallel β-sheet sandwiched between nine α-helices and hosts a characteristic PPi-binding site and a P-loop (262–267, red). The 85° GATase domain rotation (for details see the ‘Characterization of GATase domain rotation' paragraph in the Methods section) is indicated by an axis along the GATase domain. The dotted line demarcates the two subunits in the dimer. (**c**) Superimposition of the wild-type (wine-red) and mutant structures (colour coding as in **b**). (**d**) Schematic representation of the transition from conformational state I to conformational state II (colour coding as in **c**).

For the full-length enzymes (dimers as shown experimentally, see below), monomers are composed of two catalytic domains, an N-terminal independent GATase (1–236) and a C-terminal ATPPase domain (237–555) (Fig. 1a,b). The GATase segment displays an α/β-structure of nine β-strands surrounded by five α-helixes. Compared with *Hs*- and *Ec*GMPS, the *Pf*GMPS GATase domain exhibits an extension (119–151) inserted between β5 and β6. This unique insertion lacks even in other *Plasmodium* GMPS and its role is yet to be determined. The structure of the isolated *Pf*GATase domain is similar to those in the full-length *Pf*GMPS_C89A/Gln and *Pf*GMPS wild-type with root mean square deviations (RMSDs) of 0.67 and 1.37 Å.

**Figure 2:**
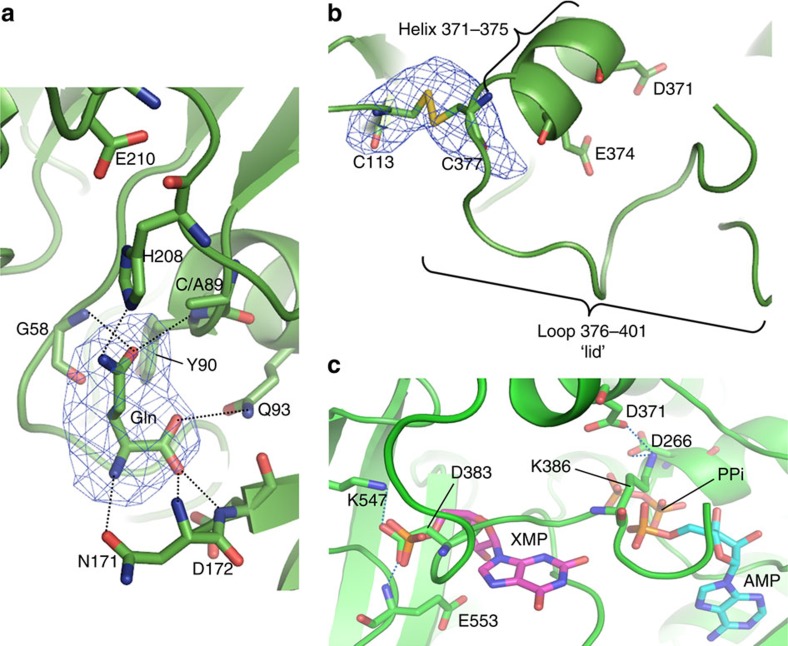
Structural features of *Pf*GMPS_C89A/Gln. (**a**) Glutamine in the GMPS active site (*F*_o_–*F*_c_ omit map contoured at 3.0 σ). Cys89 (alanine in *Pf*GMPS_C89A), His208 and Glu210 form the catalytic triad. The Nα group of glutamine interacts with the Asn171 side chain carbonyl while the α-carboxyl interacts with the main chain amino group of Asn171 and Asp172 and with the side chain of Gln93. The oxyanion hole is formed by the main chain amino group of Tyr90 following the catalytic residue and by the main chain amino group of Gly58 on an adjacent loop. Glutamine interacts with the catalytic His208 and Ala89 (this latter mutated) through its amide group. (**b**) *F*_o_–*F*_c_ omit map contoured at 3.0 σ around the S–S bond between Cys113 and Cys377, which in the crystal locks the inter-domain interface, allowing the stabilization and the visualization of helix 371–375 and loop 376–401. (**c**) Interactions of Asp383 and Lys386 within loop 376–401 at the bottom of the *Pf*ATPPase domain in *Pf*GMPS_C89A/Gln are responsible for the stabilization of this latter. Asp383 forms a salt bridge and a hydrogen bond with Lys547 and the main chain amino group of Glu553, respectively, mimicking XMP/GMP phosphate. Lys386 forms salt bridges with Asp266 and Asp371, and forces the loop to occupy the ATP/AMP+PPi-binding site, blocking the enzyme in an inactive state. AMP and XMP were assigned by superimposition with AMP-bound *Ec*GMPS (PDB-ID: 1GPM) and XMP-bound *Pf*GMPS (PDB-ID: 3UOW) structures. Asp383 mimics XMP-α-phosphate and Lys386 occupy the AMP-binding site, positioning the loop at the position normally occupied by the two substrates.

Residues Cys89 (mutated to alanine in *Pf*GMPS_C89A), His208 and Glu210 form the catalytic triad. In the structure of *Pf*GMPS_C89A/Gln, glutamine interacts with catalytic site residues Gln93, Asn169, Asn171 and Asp172. This is the first time that a glutamine co-substrate has been observed in a GMPS structure (Fig. 2a). Its carbonyl oxygen is 3.7 Å from the main chain nitrogen of Gly58 and 3.6 Å from the main chain nitrogen of Tyr90 allowing hydrogen-bonding after thioester adduct formation with the catalytic Cys89. Glutamine is ideally oriented for oxyanion hole formation (Fig. 2a). The substrate amido-NH_2_ group stabilizes the orientation via hydrogen bonds to the Asn169 main chain carbonyl and the catalytic His208 side chain. This latter acts as a general base by accepting a proton from Cys89, which in turn performs the nucleophilic attack^14^,^15^. The remaining part of the glutamine is oriented via hydrogen bonds between the Cα carbonyl group of the substrate and the side chains of Gln93 and Asp172 and by the main chain Asn171 and Asp172 amino groups. In this orientation, glutamine is within hydrogen-bonding distance (3.3 Å) of the catalytic residue Cys/Ala89, the ensemble mimicking the pre-catalytic state of the GATase domain.

GATase- and ATPPase domains are connected through a linker (228–240). The ATPPase domain displaying a five-stranded parallel β-sheet sandwiched between nine α-helices (Fig. 1a,b), hosts a characteristic PPi-binding site and a P-loop, including residues 262–267. Residues 376–401 form a ‘lid' region closing the active site (Fig. 2b).

### Dimer interface

The 108 C-terminal residues of the ATPPase domain are involved in dimerization. This interface (similar in *Pf*GMPS and *Pf*GMPS_C89A/Gln, Fig. 1a,b) is mainly formed by helices α16 and α17 and a three-stranded parallel β-sheet (β15, β16 and β17/β18). Interestingly, the nucleotide-binding site of *Pf*GMPS comprises residues from two adjacent monomers, strongly suggesting that the dimer is essential for enzymatic activity. Two *cis*-prolines (Pro548–Pro549) enable a tetrahedral configuration of Asp543, Thr551, Glu553 and Arg539′ (prime denotes a residue from the other subunit) whose side chains contribute to the homo-dimerization by electrostatic interactions (Supplementary Fig. 2). Residues, forming intermolecular hydrogen bonds, are conserved in prokaryotic *Tt*GMPS and *Ec*GMPS but not in *Hs*GMPS, which exhibits a 100 amino-acid residues insertion in the ATPPase/dimerization domain.

### Small angle X-ray scattering structure confirms dimer

Small angle X-ray scattering (SAXS) confirmed the dimeric structure of *Pf*GMPS in solution (Supplementary Fig. 3a,b) corresponding to the active form of the enzyme, as seen from the good fit of the dimer crystal structure superimposed onto the calculated 17 Å resolution *Pf*GMPS SAXS envelope. *Pf*GMPS_C89A/Gln SAXS data (24 Å resolution) confirm the dimeric arrangement (Supplementary Fig. 3c,d).

### GATase conformational change

**Figure 3:**
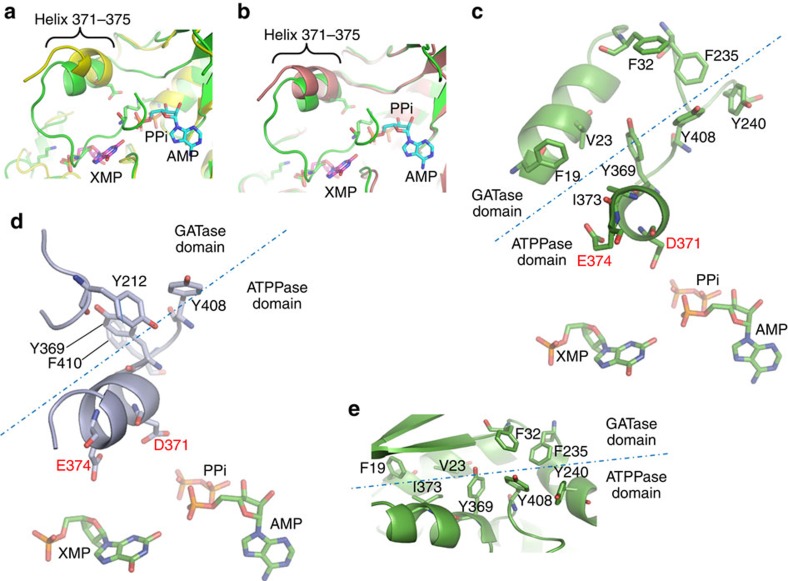
Destabilization of the domain interface by helix reorientation. (**a**) Superposition of ligand-free *Pf*GMPS (yellow) and *Pf*GMPS_C89A/Gln (green) (**b**) Superposition of the XMP-bound (pink) and *Pf*GMPS_C89A/Gln structures (green). Loop 376–401 stabilized at the bottom of the ATPPase catalytic site forces helix 371–375 to bend towards XMP and AMP (adenyl-XMP). In ligand-free and XMP-bound native enzymes, the unbent helix is linked to a destabilized 376–401 loop. (**c**) A strong hydrophobic network formed by Tyr369 and Tyr408 (ATPPase domain), Tyr240 and Phe235 (linker) and Phe32 (GATase domain) in the apo-enzyme (conformational state I). Phe19, Val23 and Ile373 strengthen the hydrophobic interface. (**d**) Transition to conformational state II creates a new hydrophobic network involving Tyr369 and Phe410 (ATPPase domain) and Tyr212 (GATase domain). (**e**) As observed in conformational state I, a strong hydrophobic network is formed by Tyr369 and Tyr408 (ATPPase domain), Tyr240 and Phe235 (linker) and Phe32 (GATase domain). Phe19, Val23 and Ile373 complete the hydrophobic interaction interface.

**Figure 4:**
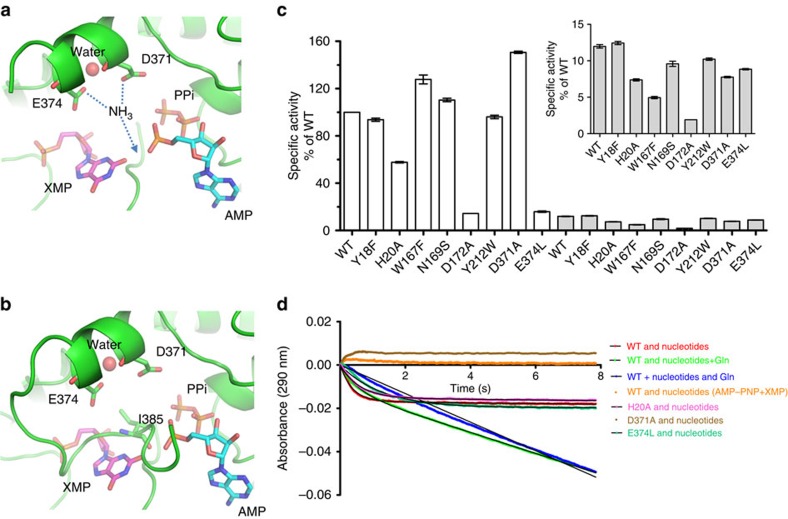
Structural and mutational analysis of *Pf*GMPS catalytic residues D371 and E374. (**a**) Asp371 and Glu374 are oriented towards the adenyl-XMP bond (positioned according to XMP in 3UOW and AMP+PPi in 1GPM). A water molecule (red), forming H-bonds with Asp371 and Glu374, could mimic NH_3_ in a favourable position for attacking the AMP-XMP intermediate, when the helix is bent. (**b**) In *Pf*GMPS_C89A/Gln, because of Ile385 at the position of the adenyl-XMP bond, the water molecule is situated slightly behind the plane in which helix 371–375 lies. (**c**) Comparison of GATase activity across wild-type and mutant enzymes. The glutamate produced was estimated at 25 °C using GDH as the coupling enzyme. To test leaky activity, 5 mM Gln, 0.5 mM NAD^+^, 8 U GDH and 25 μg *Pf*GMPS/mutants were used in a 250 μl reaction volume. To monitor GATase activation, 5 μg PfGMPS/mutants supplemented with 150 μM XMP, 2 mM ATP, 20 mM MgCl_2_ was used. White bars represent GATase activity in presence of ATP and XMP, solid grey bars denote leaky GATase activity. The specific activities are normalized to the GATase activity of the WT in presence of ATP and XMP. Inset shows leaky GATase activity with expanded Y-axis scale. The error bars represent s.e.m. of data from three experiments. (**d**) Stopped-flow absorbance traces of reaction catalysed by *Pf*GMPS and mutants at 25 °C. For individual traces, all data points were normalized against A_290_ at 110 ms. Data for reaction-progress curves obtained by mixing enzyme with either nucleotides or nucleotides and Gln were fitted to equation 1. Data for reaction-progress curves obtained by mixing nucleotide-pre-incubated-enzyme with Gln were fitted to equation 2. Data are represented as points, fits are black lines. No drop in absorbance was seen when either *Pf*GMPS_D371A was mixed with nucleotides or when ATP was replaced with AMP-PNP in the reaction catalyzed by *Pf*GMPS. The final concentration of the nucleotides was 150 μM XMP, 2 mM ATP/AMP-PNP. Each trace is an average of 3 or more shots and the experiment repeated twice yielded similar results. The averaged trace from one experiment is shown.

The *Pf*GMPS_C89A/Gln structure shows an unexpected conformational state of the GATase domain never observed in this family of enzymes. Upon superposition of the *Pf*GMPS- (conformational state I) and *Pf*GMPS_C89A/Gln (conformational state II) dimer structures, the two ATPPase domains remain invariant, whereas the two GATase domains differ by an 85° rotation and a 3 Å translation (for details see Methods) when going from states I to II (Fig. 1a–d). This 85° GATase domain rotation locked by a disulfide bond between Cys113 and Cys377 (Fig. 2b) implies a linker conformational change. Cys377, at the end of helix 371–375, is the first residue of the catalytic loop 376–401 which forms a ‘lid' closing the active site (Fig. 2b). Electron density is present for residues 376–389 and 397–401, while in the full-length form and in other reported structures (except *Hs*GMPS) the lid is not visible in the electron density. Indeed, the rotation forces the N-terminal part of the lid to enter the ATPPase domain. Other interactions stabilize residues 376–389 as well (Fig. 2c), whereas the remaining part (390–396) floating above the ATPPase catalytic site, is not well defined. By using structures of *Ec*GMPS (PDB-ID 1GPM^11^) containing AMP+PPi and *Pf*GMPS containing XMP, we docked AMP+PPi and XMP in the catalytic site of the ATPPase domain of *Pf*GMPS_C89A/Gln. Because of steric hindrance, both substrates cannot be accommodated with the lid simultaneously occupying the space of AMP and XMP (Fig. 2c). Also, the presence of the lid at the bottom of the ATPPase active site induces conformational changes in helix 371–375, forcing it to bend (Fig. 3a–c). This is observed when superposing conformational states I (3UOW and ligand-free *Pf*GMPS) and II (4WIO, *Pf*GMPS_C89A/Gln). The position of helix 371–375 bearing the catalytic Asp371 and Glu374 with respect to XMP and AMP+PPi in the structures of 3UOW and 1GPM, respectively, suggests that both are possibly oriented towards the reaction intermediate adenyl-XMP. In the *Pf*GMPS_C89A/Gln structure, Asp371 and Glu374 are perfectly positioned in front of the expected location of the adenyl-XMP bond (Figs 3c,d and 4a,b).

### Isolated domains retain activity

Size-exclusion chromatography shows that in solution *Pf*GATase is largely monomeric while *Pf*ATPPase is dimeric, confirming that dimerization is mediated via this latter domain (Supplementary Fig. 4a–d). The folding of isolated *Pf*GATase is similar to that seen in full-length *Pf*GMPS_C89A/Gln and *Pf*GMPS wild-type structures with RMSDs of 0.67 and 1.37 Å. *Pf*GATase exhibited leaky glutaminase activity, unlike other GMPS characterized to date^11^,^16^ but similar to *Pf*GMPS (Supplementary Table 1). Michaelis–Menten plots for glutamine with *Pf*GATase and unliganded *Pf*GMPS (lacking ATP, XMP and Mg^2+^) did not achieve saturation, hence true *V*_max_ and *K*_m_ values could not be estimated. The turnover number at 50 mM glutamine for its hydrolysis by *Pf*GATase and *Pf*GMPS were 35±4 and 60±13 min^−1^, respectively, while the *k*_cat_ for GMP formation is 37±3 min^−1^ (Table 2, Supplementary Fig. 1). However, apparent *K*_m_ values for glutamine for *Pf*GMPS in the absence of ATP and XMP (128±13 mM) and for *Pf*GATase (101±7 mM) were 280–360-fold higher than that for *Pf*GMPS under conditions of GMP formation (Table 2, Supplementary Table 1). Although *Pf*GATase alone has very-poor affinity for glutamine, the high specific activity values support (as seen in the structure) that the triad and oxyanion hole are positioned in the conformation required for catalysis and the loop movement occurring upon ligand-binding to ATPPase primarily leads to increase in substrate affinity.

The *k*_cat_ for ammonia-dependent *Pf*ATPPase activity was 5.9-fold lower than that for *Pf*GMPS while the *K*_m_ value was unaltered for XMP and 1.8 and 2.7-fold lower for ATP and NH_4_Cl, respectively (Supplementary Table 1). The modulation of *k*_cat_ and *K*_m_ values for the substrates of both active sites suggests a concerted action between the two domains, as supported by the structure (Figs 1a and 3c–e).

### Mutational analyses validate structural observations

Far-UV circular dichroism spectra of native and mutant *Pf*GMPS (Supplementary Fig. 4e) were similar, ruling out gross structural alterations brought on by the mutations.

*GATase mutants*. A hydrogen-bonding network between the side chains of Tyr18, Asn169 and Trp167 interconnecting the different structural elements of the GATase active site, are specific to *Pf*GMPS (Supplementary Fig. 4f) and were therefore subjected to mutagenesis studies. Concerning the glutamine-dependent activity, *Pf*GMPS_Y18F and *Pf*GMPS_W167F showed no major differences in kinetic characteristics, whereas *Pf*GMPS_N169S displayed a twofold increase in *k*_cat_/*K*_m_ (Supplementary Table 1). For the NH_4_Cl-dependent activity, *K*_m_ values for all three mutants were 2.5-fold lower than that for the wild-type enzyme, whereas *k*_cat_ values were 1.4-fold lower for *Pf*GMPS_Y18F and similar to that of the wild-type for *Pf*GMPS_W167F and *Pf*GMPS_N169S (Supplementary Table 1). Although all three mutants exhibited activation of GATase activity upon ligand-binding to the ATPPase domain, *Pf*GMPS_W167F compared with the wild-type enzyme showed 60% reduction in leaky GATase activity (Fig. 4c). These differences suggest that interactions seen in the crystal structure, involving these three residues, are important for at least the structural integrity of the GATase active site and thereby do have functional implications.

In the structures of imidazole-glycerol-phosphate synthase, cytidine-5′-triphosphate synthetase and formylglycinamide synthetase, all type-I GATases, the α-amino and carboxy groups of bound Gln/acivicin/6-diazo-5-oxo-norleucine (DON) interact with a conserved Asp/Glu in the GATase active site^17^,^18^,^19^,^20^,^21^. Also, in the *Pf*GMPS_C89A/Gln structure, D172 (Asp/Glu residue across GMPS) interacts with the α-carboxy of Gln. *Pf*GMPS_D172A exhibited a 170-fold increase in *K*_m_ for Gln under the conditions of GMP formation, suggesting that this residue is a key determinant of substrate-binding to the GATase active site. It should be noted that the steady-state kinetic parameters for NH_4_Cl-dependent activity were unchanged for *Pf*GMPS_D172A (Table 2).

*Non-active site GATase residues modulate GMP synthesis*. The GATase domain rotation in the *Pf*GMPS_C89A/Gln structure has brought Cys113 (GATase domain) and Cys377 (ATPPase domain) in close proximity leading to the formation of a disulfide bond. We generated the double mutant *Pf*GMPS_C89A/C113A to address domain rotation in solution. Previously, *Pf*GMPS_C89A was found to be devoid of Gln-dependent GMPS activity while the ammonia-dependent activity was intact^6^ (Table 2). Also, it was observed that binding of Gln to *Pf*GMPS_C89A decreased the rate of ammonia-dependent GMP formation. The double mutant behaved as *Pf*GMPS_C89A, lacking Gln-dependent GMPS activity and with Gln inhibiting ammonia-dependent activity. However, this double mutant displayed 6.7-fold decrease in *K*_m_ for NH_4_Cl compared with the wild-type and a 2.9-fold decrease compared with *Pf*GMPS_C89A. With largely unaltered *k*_cat_ values this mutant exhibited the highest *k*_cat_/*K*_m_ value for ammonia-dependent GMP formation (Table 2). Although the exact role of Cys113 is unknown, the effect of mutating Cys113 on the steady-state kinetic parameters suggests that a domain-flip occurs in solution and is part of the catalytic process. The domain rotation also reorganizes the aromatic cluster at the interface of the two domains resulting in a new interaction between Tyr212 (GATase domain) and Tyr369 (ATPPase domain) (Fig. 3d). While both *k*_cat_ and *K*_m_ values for NH_4_Cl for *Pf*GMPS_Y212W did not show any significant change, the *K*_m_ value for Gln showed a 2.8-fold increase with a 2-fold drop in *k*_cat_/*K*_m_ when compared with *Pf*GMPS (Table 2), again corroborating the structural observations. His20 (in close vicinity of Glu374 in the apo *Pf*GMPS structure) was mutated to Ala, giving a 23% drop in leaky activity (Fig. 4c). The *k*_cat_/*K*_m_ value for glutamine-dependent activity was 2.5-fold lower than the wild type (Supplementary Table 1) and this is reflected as 57% lower level of activation (Fig. 4c). Furthermore, this mutant has a reduced *K*_m_ value for ATP that is similar to that for *Pf*ATPPase in which the interaction between Glu374 and His20 would not be possible (Supplementary Table 1).

*ATPPase domain mutants*. The structure of *Pf*GMPS_C89A/Gln suggests that Asp371 and Glu374 are involved in catalysis as they are positioned directly in front of the adenyl-XMP bond (Fig. 4a). *Pf*GMPS_E374L was completely devoid of Gln-dependent GMPS activity whereas *k*_cat_/*K*_m_ for ammonia-dependent activity was 70-fold lower than that of *Pf*GMPS (Table 2). *Pf*GMPS_D371A exhibited weak NH_4_Cl-dependent GMP formation with a specific activity of 2 nmol min^−1^ mg^−1^ being 420-fold lower than that for *Pf*GMPS (834±4 nmol min^−1^ mg^−1^).

Using a stopped-flow device, we measured the rate of adenyl-XMP formation at 25 °C by the wild-type and the ATPPase mutants. Mixing *Pf*GMPS with a solution containing nucleotides and Gln yielded a progress curve with a time-dependent drop in absorbance having two distinct phases with apparent rate constants for the first and second phases being 111±2 and 27±2 min^−1^, respectively (Fig. 4d). The latter value is similar to the steady-state *k*_cat_ for GMP formation. Mixing the enzyme with ATP and XMP yielded a progress curve with a single phase and an apparent rate constant of 156±5 min^−1^. Control reactions where adenosine 5′-(β,γ-imido)triphosphate (AMP-PNP) was substituted for ATP or enzyme was premixed with ATP and XMP before initiation of the reaction, established that the first phase arises from an on-enzyme step involving the formation of an adenyl-XMP intermediate (Fig. 4d). Measurements on *Pf*GMPS_E374L in the presence of only nucleotides yielded a single phase-progress curve with an apparent rate constant of 44±3 min^−1^ that is 3.5-fold lower than that for *Pf*GMPS (Fig. 4d). While the rate of adduct formation is compromised by a factor of 3.5, the *k*_cat_ for ammonia-dependent GMPS activity is 8.6-fold reduced (Table 2). Similar experiment with only nucleotides, on *Pf*GMPS_D371A, using an enzyme concentration of 7.5 μM did not report on adenyl-XMP formation (Fig. 4d). However, HPLC analysis with 100 μM enzyme showed a small peak corresponding to adenyl-XMP (Supplementary Fig. 5) and corroborated the very weak activity measured by steady-state spectrophotometric assays.

The leaky glutaminase activities of *Pf*GMPS_D371A and *Pf*GMPS_E374L were uncompromised. Activation of GATase activity by the binding of ligands to the ATPPase domain was not seen in *Pf*GMPS_E374L while the activation of *Pf*GMPS_D371A was 150% of the wild type (Fig. 4c). This potent activation of glutaminase activity in *Pf*GMPS_D371A in the presence of both ATP and XMP (Fig. 4c) confirmed that the mutant binds the ligands. Initial velocity measurements of the glutaminase activity of *Pf*GMPS_D371A at varied concentrations of one substrate and saturating concentration of the other enabled estimating apparent *K*_m_ values that were 6.3±0.4 μM for ATP and 0.20±0.02 μM for XMP. These values were drastically lower than the corresponding *K*_m_ values for *Pf*GMPS (Table 2). This suggests that the wild-type enzyme has higher affinity for the transition-state that is along the reaction pathway for adenyl-XMP formation than for the substrate(s); whereas, although the mutant binds the substrates tightly, its ability to catalyse intermediate formation is severely impaired. Further, these results indicate that Glu374 is involved in both adenyl-XMP formation, inter-domain crosstalk and in the utilization of external NH_4_Cl. As the structure of *Pf*GMPS shows inter-domain interaction *via* Glu374 and His20, the rate of adenyl-XMP formation in *Pf*GMPS_H20A upon mixing the enzyme with ATP and XMP was measured. A progress curve with a single slope and an apparent rate constant of 96±6 min^−1^ was obtained (Fig. 4d), and the 1.6-fold drop of the rate constant validates the inter-domain interaction seen in the structure (Fig. 3c).

### Molecular dynamics simulation of the conformational change

A molecular dynamics study was performed to investigate the pathway going from conformational states I to II. Starting from ATPPase domains superimposed from *Pf*GMPS and *Pf*GMPS_C89A structures, the GATase domain was separated from the rest of the protein. To have a motion picture showing the movement going from conformation I to II when rotating about an axis defined by Thr175 and Lys30, eight different conformation frames were generated with an interframe position being inferior to 2 Å using Asn49 as reference point (Supplementary Fig. 6). As observed, the maximum energy is roughly equal to 20 kcal mol^−1^, which is the energy corresponding to thermal cavitation at room temperature. Calculated values between two consecutive conformations indicate that the eight conformations observed for the GATase domain within this dynamics study are attainable. Hence we conclude that the observed rotation is possible. The two lowest energy conformations correspond to positions 6 and 8.

## Discussion

**Figure 5:**
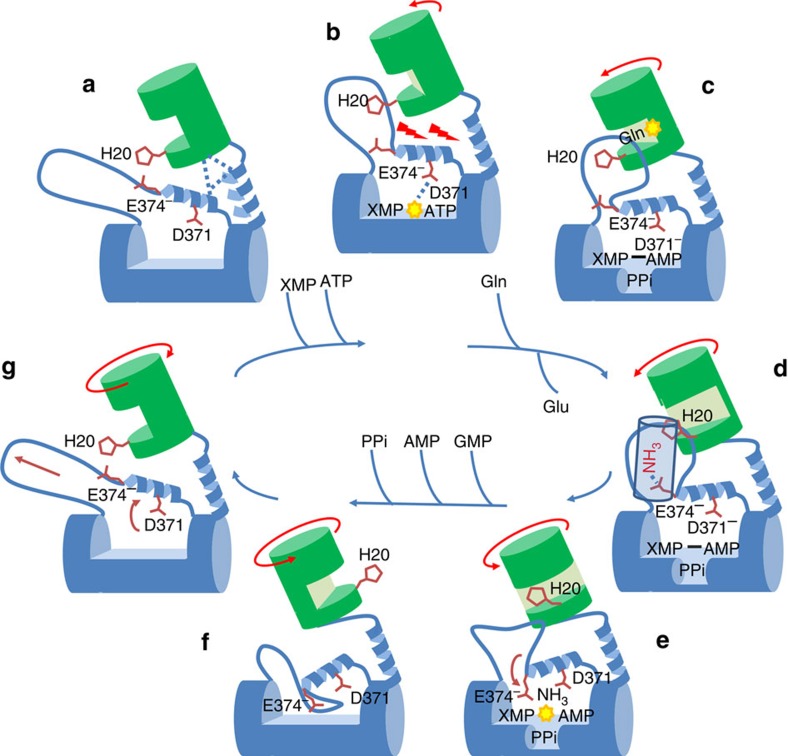
Model illustrating the catalytic mechanism of *Pf*GMPS. (**a**) In conformational state I, loop 376–401 is not in the ATPPase catalytic site, thereby allowing binding of XMP and ATP. (**b**) Upon binding of ATP and XMP, concomitant with ATP hydrolysis resulting in AMP-XMP and PPi, helix 371–375 starts to bend, disrupts interface interactions, reorients loop 376–401 forcing the GATase domain to rotate and creates a tighter binding site for glutamine. (**c**) Glutamine binds and the oxyanion hole formation induces its deamination. (**d**) Because of channel formation, NH_3_ moves towards the ATPPase acceptor site driven by interaction with Glu374. (**e**) Attack of adenyl-XMP to form GMP. (**f**) Once in conformational state II, loop 376–401 enters the ATPPase domain, and abstracts GMP, AMP and PPi. (**g**) The GATase domain, helix 371–375 and loop 376–401 regain their respective initial conformations. His20 is used as reference point for the rotation of the GAT domain.

GMP formation and deamination of glutamine in *Pf*GMPS are coordinated reactions (Supplementary Fig. 1) implying tightly controlled conformational changes^9^ (Fig. 5). Compared with the *Pf*GMPS three-dimensional structure, the GATase domain of the inactive mutant *Pf*GMPS_C89A/Gln crystal structure has undergone an important rotation giving rise to a conformational change never observed so far. This new conformation could present the missing step towards a comprehensive understanding of the catalytic mechanism. Substrate-binding to *Pf*GMPS is a sequential process (Supplementary Fig. 1), starting with ATP and followed by XMP^8^ and steady-state kinetic parameters reported here for the unfused GATase and unliganded *Pf*GMPS show more than 280-fold increase in affinity for glutamine upon ligand-binding. *Pf*GMPS/XMP (3UOW) and *Ec*GMPS/AMP+PPi^11^ structures display GATase domain orientations similar to the apoenzyme, suggesting that concomitant binding of ATP and XMP is mandatory before the 85° rotation. As no three-dimensional structure with both substrates bound concomitantly exists, the exact mechanism of domain rotation remains elusive. Our molecular dynamics simulations indicate that the GATase domain may perform an 85° rotation around an axis going from Lys104 to Ile29 through a 50° midpoint, in which glutamine is optimally oriented and in-line with the adenyl-XMP bond (Supplementary Fig. 6). This is in agreement with NH_3_ transfer occurring during a pause corresponding to a transition going from a low- to a high-energy intermediate.

The disulfide bond seen in the crystal structure that locks the enzyme in an inactive conformation is probably not relevant in solution as rapid domain movements may prevent bond formation. In routine assays with XMP concentration at 150 times that of enzyme, 40% conversion of substrate to product is achieved in 100 s. Hence, during this period each molecule has gone through 63 cycles of turnover. During catalysis the enzyme therefore does not get trapped in the S–S bonded state for otherwise, a turnover greater than one would not have been achieved. Examination of 60 GMPS sequences from various organisms shows that Cys113 and Cys377 are not conserved (Cys377 is only found in *Plasmodium*, Cys113 only in *P. falciparum*, *P. vivax* and *P. knowlesi*). Nevertheless, the fortuitous stabilization of the rotated state highly contributes to our understanding of the molecular mechanism of catalysis in GMPS. In conformational state II, Cys113 is brought in close proximity with Cys377 which is part of the loop following the helix holding Asp371, and as evident from mutagenesis, a key residue in AMP-XMP intermediate formation. Mutating Cys113 to Ala dramatically increases the catalytic efficiency of NH_4_Cl-dependent GMP formation and alters *K*_m_ values for ATP and XMP. This fully supports rotation of the GATase domain in solution and its key role in catalysis. Interestingly, when comparing structures of apo- or XMP-bound enzymes (state I) with the rotated mutant enzyme (state II) (Fig. 3), a bending of helix 371–375 was observed. This helix participates in the formation of hydrophobic and aromatic clamps involving residues from both domains, and from the linker which strongly stabilizes the domain interface (Fig. 3c,e). These aromatic clamps have disappeared in the *Pf*GMPS_C89A/Gln structure (Fig. 3d). Upon rotation, new functionally relevant interactions between Phe410, Tyr369 and Tyr212 are formed as corroborated by the 2.8-fold increase in *K*_m_ value for Gln for *Pf*GMPS_Y212W. Probably binding of ATP and XMP by changing the conformation of helix 371–375 could be responsible for the interface rearrangement. It also seems plausible that the charge-mediated interaction between Glu374 and His20, along with the hydrophobic interface contributes to stabilize *Pf*GMPS in the conformation observed for ligand-free and XMP-bound enzymes. The reorientation of Glu374 could be part of the rotation-triggering-signal via inter-domain communication through His20. While mutation of His20 to Ala lowered the *K*_m_ for ATP, the *k*_cat_ for GMP formation and the rate of adenyl-XMP formation, mutating Glu374 to Leu completely impaired inter-domain crosstalk upon ATP- and XMP-binding to the ATPPase domain. We conclude that Glu374 is the key determinant of allostery, as a single mutation completely abolishes glutamine hydrolysis. The invariant nature of this residue across all GMPS suggests conservation of its function (Supplementary Fig. 7).

Transition to conformational state II results in loop 376–401 (lid) being deeply buried into the ATPPase domain (Figs 2b,c, 3a,b and 4a,b). This conformation could correspond to the very last step of the catalytic reaction when the lid enters the catalytic site to release the reaction products GMP, AMP and PPi. Interestingly, when superposing the structures of individual GATase domains (*Pf*GATase, *Pf*GMPS_C89A/Gln, *Pf*GMPS) no conformational differences were observed, inferring that the major determinant for an increased glutamine-affinity in the presence of ATP and XMP should be found in the ATPPase domain close to the interface. Altogether, loop 376–401 emerges as a good candidate. The presence of ATP and XMP could force the lid to adopt a position above the two catalytic sites as suggested in Fig. 2c and Fig. 3a,b, forming concomitantly a tighter glutamine-binding site along with a pathway for channelling.

Hydrolysis of glutamine depends only on substrate-binding, and increasing the affinity is sufficient to increase the catalytic efficiency of the reaction. This is in agreement with glutamine in the mutant being perfectly positioned for adduct formation with the catalytic residue Cys89 (Ala89 in *Pf*GMPS_C89A/Gln), and suggests that no other part of the enzyme is needed for creating a proper catalytic pre-state (Fig. 2a). Leaky activity in absence of ATP and XMP as well as glutaminase activity when lacking the ATPPase domain corroborates this hypothesis. Hence, the loop-generated platform seems to be the major determinant for the increased affinity and activity of the GATase domain. Unfortunately, the corresponding conformation (Fig. 5) is probably very transitory and therefore, difficult to trap.

The lid induces conformational changes in helix 371–375 when present at the bottom of the ATPPase active site. We suggest that when the GATase domain has engaged its rotation, the lid would be above the adenyl-XMP closing the ATPPase active site. The rotation may gradually force the loop to enter the ATPPase domain, and thereby bend helix 371–375, placing NH_3_ in a favourable orientation. During this process, Asp371 along with Glu374 would place NH_3_ in front of the adenyl-XMP bond. It is tempting to imagine that the water molecule forming hydrogen bonds with Asp371 and Glu374 (Fig. 4a) could be replaced by NH_3_, so that when the helix is bent, NH_3_ would be in a favourable position for the reaction. Ultimately, after the conformational change to state II, the helix 371–375 bending would be at a maximum and the lid would be at the bottom of the ATPPase domain, displacing the products of the reaction. This hypothesis is reinforced by Asp371 interacting with Lys386 in *Pf*GMPS_C89A/Gln, thereby blocking the enzyme in an inactive conformation. Figure 5 summarizes key conformational changes occurring during the catalytic cycle. It is highly possible that release of the lid-loop triggers the rotation of the GATase domain back to its resting state. This is further supported by the fact that in all structures where the lid-loop is either disordered (all except *Hs*GMPS) or ordered but not in the ATPPase pocket (*Hs*GMPS), the GATase domain is not rotated.

*Pf*GMPS_C89A displays partial uncompetitive glutamine-inhibition with respect to all three substrates^6^ indicating that NH_4_^+^, ATP and XMP could participate in the conformational changes of helix 371–375 and the lid, and thereby increase the affinity for glutamine. In this mutant, *k*_cat_ for NH_4_Cl-dependent GMP formation is not impaired (Table 2), affinity for glutamine is still observed and as evident from the structure, rotation still occurs. However, during rotation, glutamine could interact with residues from the lid and thereby temporarily block the whole enzyme in an intermediate conformation, before achieving the rotation. In this conformation, NH_3_ should be present somewhere between the GATase- and ATPPase domain interacting with residues of helix 371–375 and/or the lid supporting the pathway for channelling (Figs 2c and 3a,b).

ATPPase domain dimerization is structurally conserved among *Ec*GMPS, *Tt*GMPS, *Pyrococcus horikoshii* GMPS (PDB-ID 2DPL), *Pf*GMPS and *Pf*GMPS_C89A/Gln (not shown). It was shown^12^ that *Hs*GMPS mimics this interface via sub-domain association (D1 and D2, the first being formed by a long insertion in the human enzyme compared with counterparts). This additional sub-domain which is not present in bacterial, archaeal and *Plasmodial* GMPS was also shown to be involved in dimerization and substrate-binding. This unique oligomeric packing has been suggested to indicate inter-monomer transfer of ammonia through the centre of the globular oligomer, illustrating a channelling radically different from the mechanism we propose. However, the authors do not exclude the possibility that the lid above the active site (ordered in *Hs*GMPS due to the stabilization by the dimer formation), could also be involved in ammonia channelling as suggested by our results. Understanding the nature of subunit interfaces is important when designing molecules impeding subunit association. As active sites in enzyme homologues from different organisms are highly conserved in primary- and tertiary structure, subunit interfaces lacking high degree of conservation are potential sites for species-specific inhibitor design. Hence, when considering development of specific inhibitors of *Pf*GMPS or *Ec*GMPS (also pointed out earlier^12^), the dimer interface becomes a potential target.

In conclusion, disulfide-bond formation induced by cysteines at key positions in *Pf*GMPS has allowed capturing and stabilizing (in the crystalline state) a rotated high-energy, intermediate state. This finding has provided for the first time a comprehensive understanding of details of the domain dynamics of GMPS, a longstanding lacuna. While the catalytic mechanism of the GATase domain is well explored^6^,^22^,^23^ our findings offer a glimpse of the catalytic process in the ATPPase domain. The unique conformation of the lid as seen in the structure of *Pf*GMPS_C89A/Gln has never been observed before. Our studies indicate that this loop is essential for all the key steps throughout the catalytic process (inter-domain communication, substrate affinity enhancement, formation of the ammonia channel, expulsion of reaction products and so on). Indeed, alignment of 600 sequences from prokaryote-, archaea- and eukaryote GMPS all contain a sequence of conserved residues ‘IK(T/S)HH' (Supplementary Fig. 7) within the lid-loop which are facing the catalytic residues Asp371 and Glu374 (also conserved) involved in adenyl-XMP formation and inter-domain signalling, respectively. Of particular importance is the isoleucine which pushes Asp371 and Glu374 at the back-side of helix 371–375 into an orientation, which is no longer compatible with catalysis, and also the lysine which forms a salt bridge at the bottom of the ATPPase binding pocket at the exact position of the product, PPi. Because of the invariant nature of these key residues, one may conclude that these steps of the reaction mechanism probably are applicable to all GMPS.

Moreover, hinge movements seem to be a general property of enzymes holding a GATase domain. In the crystal structure of DON-modified, glucose-6-phosphate-bound *Escherichia coli* glucosamine-6-phosphate synthetase, a 23° GATase domain rotation is seen^24^, while a 3.6° rotation of the GATase domain was reported for sulfate-bound CTP synthetase^20^. Our work therefore, provides a general mechanistic framework for the catalytic process of this important and therapeutically relevant enzyme. Since GMPS due to their two domains can be regarded as a kind of prototype for two families of metabolic enzymes, namely ‘class-I glutamine amidotransferases' and ‘N-type ATP pyrophosphatases', our results could also pave the way for future experiments contributing to the understanding of the functioning of these enzyme classes.

## Methods

### Cloning of *Pf*GMPS domains and mutagenesis

Sequence alignment of *Pf*GMPS with two subunit GMPS from archaea was used to demarcate GATase (residues 1–236) and ATPPase (residues 237–555) domains. *Pf*GATase and *Pf*ATPPase DNA fragments were amplified by PCR (Supplementary Table 2). The fragments were digested and cloned into the vector pET-21b(+). The resulting clones were confirmed by DNA sequencing. All proteins, except *Pf*ATPPase were fused to an N-terminal 6His tag.

Site-directed mutants of *Pf*GMPS were constructed using pET*Pf*GMPS^6^ as template. Mutants Y18F, C113A and W167F were constructed by the single primer method involving the introduction of a restriction site at the codon to be mutated followed by knockout of the site to generate the desired mutation^25^ (Supplementary Table 2). Plasmid pET*Pf*GMPS_C89A^6^ was the template for the C113A mutation. Mutants H20A, N169S, D172A, Y212W, D371A and E374L were constructed using the QuikChange II site-directed mutagenesis kit (Agilent Technologies) protocol. Phusion DNA polymerase (Thermo Scientific) was used for PCRs. All PCR products were digested with DpnI (New England Biolabs) before transformation and clones were verified by DNA sequencing.

### Protein Purification

Protein expression and purification of *Pf*GMPS wild type and mutants were carried out as described earlier^6^ with minor modifications. Wild-type *Pf*GMPS was expressed from pQE30*Pf*GMPS construct in *E. coli* strain AT2465 (*λ*−*, e14*−*, guaA21, relA1, spoT1* and *thi*−*1*) as reported^8^. pET*Pf*GMPS mutant constructs were transformed into BL21-CodonPlus(DE3)-RIL. A single colony was inoculated into 5 ml Terrific Broth containing 100 μg ml^−1^ ampicillin and 34 μg ml^−1^ chloramphenicol and grown overnight at 37 °C. An amount of 0.5% inoculum of the overnight culture was added to 50 ml Terrific Broth supplemented with antibiotics, grown at 37 °C for 6 h and further used at 0.5% to inoculate 2.8 l of Terrific Broth supplemented with antibiotics. This culture was grown to an OD_600_ of 0.6, induced with 0.5 mM IPTG (isopropyl-β-D-thiogalactopyranoside) and grown for further 18 h at 18 °C. After harvesting and storage (−80 °C), the cell pellet was resuspended in 50 mM Tris-HCl, pH 7.4, 10% (v/v) glycerol, 0.1 mM DTT (dithiothreitol), 0.1 mM PMSF (phenylmethanesulfonylfluoride)—Buffer A. The cell suspension was supplemented with 1 mM PMSF and sonicated on ice for four cycles of 1 min each. The cell lysate was centrifuged at 30,500*g* for 45 min following which the supernatant was loaded onto 2 × 1 ml HisTrap HP columns (GE Healthcare) connected in tandem using ÄKTA Basic HPLC (GE Healthcare). The column was washed with Buffer A containing 1 M NaCl followed by step gradients of 20, 40, 60, 80 and 100 mM imidazole in Buffer A. Protein was eluted with Buffer A containing 500 mM and finally 1 M imidazole. After 12% SDS–PAGE (SDS–polyacrylamide gel electrophoresis) analysis, fractions with relatively pure protein were pooled and concentrated (30 kDa cut-off Amicon Ultra-15 centrifugal filter units (EMD Millipore)). Further purification by size-exclusion chromatography using HiLoad 16/600 Superdex 200 pg column (GE Healthcare) was done in 20 mM Tris-HCl, pH 7.4, 10% (v/v) glycerol, 1 mM EDTA and 2 mM DTT. Aliquots of the purified protein were flash frozen and stored at −80 °C.

pET*Pf*GATase was transformed into *E. coli* Rosetta(DE3)pLysS, and transformants grown in Terrific Broth at 37 °C to an OD_600_ of 0.6 before induction with 0.05 mM IPTG. Cells were further grown for 6 h at 37 °C, centrifuged and the cell pellet resuspended in lysis buffer (20 mM Tris-HCl pH 7.4, 10% (v/v) glycerol, 1 mM DTT and 0.1 mM PMSF) and lysed using a French press. After centrifugation, the supernatant was incubated for 2 h at 4 °C with Ni-NTA agarose beads (Qiagen). Thereafter, the beads were washed with lysis buffer containing 100 mM NaCl followed by wash with lysis buffer containing 10 mM imidazole. Protein was eluted with lysis buffer containing 500 mM imidazole and 1 mM EDTA was added immediately after elution. After 10% SDS–PAGE, pure fractions were pooled and extensively dialysed against 20 mM Tris-HCl pH 8, 10% (v/v) glycerol, 2 mM DTT and 0.1 mM PMSF. Buffer exchanged protein samples were subjected to anion-exchange chromatography (Q-Sepharose high performance media (GE Healthcare)). Pure protein fractions (10% SDS–PAGE verification) were pooled and concentrated (10 kDa cut-off Amicon Ultra-15 centrifugal filter units (EMD Millipore)). Concentrated protein was further purified by gel filtration on HiLoad 16/600 Superdex 200 pg column in 20 mM Tris-HCl, pH 7.4, 10% (v/v) glycerol, 2 mM DTT, 0.1 mM PMSF and 1 mM EDTA. Protein aliquots were flash frozen and stored at −80 °C.

pET*Pf*ATPPase was transformed into *E. coli* BL21(DE3) cells and transformants grown in Terrific Broth at 37 °C to an OD_600_ of 0.6 before induction with 0.05 mM IPTG. Cells were further grown for 12 h at 23 °C, and pelleted and resuspended in lysis buffer (20 mM Tris-HCl, pH 7.4, 10% (v/v) glycerol, 2 mM DTT and 0.1 mM PMSF) and lysed using a French press. After centrifugation, soluble *Pf*ATPPase was precipitated with 60% (w/v) ammonium sulfate, and the pellet resuspended in lysis buffer and buffer exchanged into 20 mM Tris-HCl, pH 7.0, 10% (v/v) glycerol, 2 mM DTT and 0.1 mM PMSF. Buffer exchanged protein samples were subjected to cation-exchange chromatography using CM-Sepharose fast flow media (GE Healthcare). After verification by 12% SDS–PAGE pure protein fractions were pooled, dialysed against buffer containing 20 mM Tris-HCl, pH 7.4, 10% (v/v) glycerol, 1 mM DTT, 0.1 mM PMSF, flash frozen and stored at −80 °C.

### Analytical size-exclusion chromatography

Analytical gel filtration was performed using HR 10/30 column packed with Superdex 200 pg matrix attached to an ÄKTA Basic HPLC system. The column was equilibrated with a buffer containing 50 mM Tris-HCl, pH 7.4, 100 mM KCl and calibrated with the standards β-amylase, alcohol dehydrogenase, bovine serum albumin, carbonic anhydrase and cytochrome c (Sigma-Aldrich). A sample volume of 100 μl containing 20 μM of *Pf*GATase and *Pf*ATPPase was injected into the column individually and eluted at a flow rate of 0.5 ml min^−1^ and the eluted proteins were detected at 220 nm. Similar conditions were used for *Pf*GMPS and its mutants.

### Circular dichroism measurements

Proteins were concentrated to 5 μM in 7 mM Tris-HCl pH 7.4, 3.3% (v/v) glycerol, 0.3 mM EDTA and 0.6 mM DTT. Far-UV CD spectra were recorded on a Jasco J-810 spectropolarimeter using a quartz cell with 1 mm path length, from 200 to 260 nm with a bandwidth of 1 nm and a scan speed of 20 nm min^−1^. Each spectrum was an average of three scans.

### Enzyme assays and kinetics

*Assay for measuring GMP formation*. GMP synthesis activity was monitored at 25 °C using a continuous spectrophotometric assay as reported^8^. A Hitachi 2010 spectrophotometer was used to monitor the reaction rates as decrease in absorbance at 290 nm due to conversion of XMP into GMP and a Δ*ɛ* value of 1,500 M^−1^ cm^−1^ was used to calculate the amount of product formed^26^. The standard assay consisted of 90 mM Tris-HCl, pH 8.5, 150 μM XMP, 2 mM ATP, 5 mM glutamine, 20 mM MgCl_2_, 0.1 mM EDTA and 0.1 mM DTT in a total reaction volume of 0.25 ml. The reaction was initiated by adding 15 μg enzyme. The steady-state kinetic parameters were obtained by measuring initial velocities over a range of substrate concentrations. When the concentration of one substrate was varied, the concentration of the other two substrates was kept fixed and saturating. ATP was varied over the concentration range 30 μM to 4 mM, XMP from 5 to 250 μM, Gln from 0.25 to 20 mM and NH_4_Cl from 1 to 250 mM. The saturating concentration of ATP, XMP, Gln and NH_4_Cl were 2 mM, 150 μM, 5 mM and 100 mM, respectively. Initial velocity data were fitted to the Michaelis–Menten equation *v*=(*V*_max_S)/(*K*_m_+S) by nonlinear regression using GraphPad Prism version 5.00 (GraphPad Software). Initial velocity measurements at varying substrate concentrations were performed in duplicate and a minimum of 14 substrate concentrations were used in all cases.

*Assay for glutaminase activity*. Glutaminase activity was measured at 25 °C by estimating the concentration of glutamate formed using either glutamate dehydrogenase (Sigma-Aldrich, USA) or *P*. *falciparum* aspartate aminotransferase (*Pf*AAT, in-house generated) as the coupling enzyme. In the GDH-coupled assay, the glutamate generated is converted to α-ketoglutarate along with the concomitant reduction of NAD^+^ to NADH. The reaction was monitored in a continuous spectrophotometric assay at 340 nm and the Glu produced was estimated using a Δ*ɛ* value of 6,220 M^−1^ cm^−1^. A 250 μl reaction mix consisted of 100 mM Tris-HCl, pH 8.5, 5 mM Gln, 0.5 mM NAD^+^, 50 mM KCl, 0.1 mM EDTA and 0.1 mM DTT. The reaction was supplemented with 8 U of GDH and initiated with 25 μg of *Pf*GMPS/mutants to test the leaky activity. To determine GATase domain activation by ligands binding to ATPPase domain, the reaction mixture was supplemented with 150 μM XMP, 2 mM ATP, 20 mM MgCl_2_ and 5 μg *Pf*GMPS/mutants. The above GDH-coupled assay was used to compare the glutaminase activity of wild-type and mutant enzymes under unliganded and liganded conditions. These assays were repeated three times.

When the coupling enzyme is *Pf*AAT^27^, in the presence of oxaloacetate, the glutamate generated is converted into aspartate and α-ketoglutarate that can be monitored as decline in absorbance at 270 nm. A Δ*ɛ* value of 782 M^−1^ cm^−1^ was used to estimate the concentration of glutamate generated in the reaction. This assay was used to obtain the steady-state kinetic parameters for glutamine for *Pf*GATase and unliganded *Pf*GMPS. The assay mixture consisted of 90 mM Tris-HCl, pH 8.5, 5 mM glutamine, 0.1 mM EDTA, 0.1 mM DTT, 1 mM OAA, 20 μg ml^−1^ pyridoxal phosphate, 26 μg *Pf*AAT and 6.5 μg of *Pf*GATase or 10 μg of *Pf*GMPS in a total reaction volume of 0.3 ml. The concentration range of glutamine used was 5–150 mM. These assays were repeated 4–5 times and the activity values were reproducible.

*Stopped-flow assays*. Stopped-flow kinetics was performed at 25 °C using a SFM 300 stopped-flow device (BioLogic Science Instruments) equipped with MOS-200/M optics. Changes in absorbance were monitored at 290 nm using a 150 W Xe(Hg) lamp as light source and TC-100/10F cuvette (1 cm path length). The total flow rate was 8 ml s^−1^ resulting in a dead time of 3.8 ms. The instrument was controlled using the Biokine32 software, version 4.62, allowing accumulation of 8,000 data points for each trace. Data were acquired every ms, and traces from three or more shots were averaged and *A*_290_ was normalized (the offset was corrected to zero) and plotted against time. The resulting traces were fitted to equations^28^.


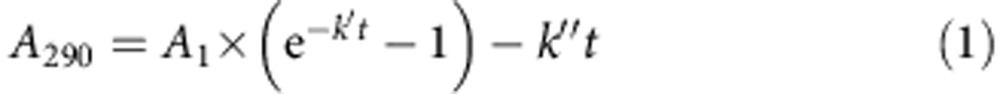


and


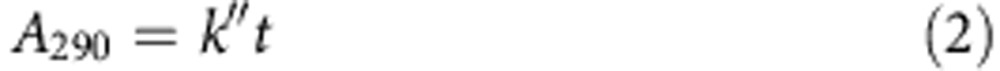


where *k*′ is the observed rate constant of the exponential phase (first faster phase), *k*′′ is the observed rate constant of the steady-state rate phase and *A*_1_ the amplitude of the exponential phase. The value of *k*_cat_ was obtained by dividing *k′′* with (Δ*ɛ* × [*E*_*t*_]) where the Δ*ɛ* value is 1,500 M^−1^ cm^−1^. For nucleotide-only stopped-flow kinetic measurements, a solution of 15 μM *Pf*GMPS wild-type or mutants in 20 mM Tris-HCl, pH 7.4, 10% (v/v) glycerol, 1 mM EDTA and 2 mM DTT was mixed in 1:1 ratio with a solution containing 180 mM Tris-HCl, pH 8.5, 300 μM XMP, 4 mM ATP, 0.2 mM EDTA, 0.2 mM DTT and 40 mM MgCl_2_. In a separate experiment, AMP-PNP (adenosine 5′-(β,γ-imido)triphosphate), a non-hydrolyzable analogue of ATP, at a final concentration of 2 mM was substituted for ATP. In assays with Gln, the syringe containing the substrates also contained 10 mM Gln. In another set of assays, 15 μM *Pf*GMPS was pre-incubated with 180 mM Tris-HCl, pH 8.5, 300 μM XMP, 4 mM ATP, 0.2 mM EDTA, 0.2 mM DTT and 40 mM MgCl_2_ at room temperature for 5 min before mixing at 1:1 ratio with 10 mM Gln in 20 mM Tris-HCl, pH 8.5 held in the second syringe. It should be noted that the concentration of the substrates used was saturating and at 10-times the respective *K*_m_ values. All experiments were repeated twice and found to be reproducible. The values of *k*′ and *k*′′, reported in the text are the mean along with the s.e.m. of the mean of two experiments.

### Reverse phase HPLC

Reverse phase HPLC was employed to detect the formation of adenyl-XMP intermediate and the product AMP. The protocol reported previously^29^ was followed with minor changes. Wild-type *Pf*GMPS or *Pf*GMPS_D371A (100 μM) were incubated in a 250 μl reaction mixture (20 mM Tris-HCl, pH 8.5, 250 μM ATP, 150 μM XMP and 5 mM MgCl_2_) for 10 min at 25 °C. The reaction was quenched by adding 70% (w/v) trichloroacetic acid to a final concentration of 2.5% (v/v) and precipitated protein was separated by centrifugation at 16,000*g* for 15 min at 4 °C. The supernatant was neutralized by addition of NaOH and 100 μl was injected into a 150 × 4.6 mm Genesis C18 reverse phase column with 4 μm beads (Grace Davison Discovery Sciences). Absorbance was monitored at 290 nm using an ÄKTA Basic HPLC equipped with a UV-900 detector at a flow rate of 0.45 ml min^−1^. The mobile phase consisted of Buffer A (50 mM potassium phosphate, pH 6.2, 4 mM tetrabutylammonium hydrogen sulfate as ion-pairing agent) and Buffer B (Buffer A supplemented with 50% (v/v) acetonitrile). After column equilibration with 10% buffer B, sample was injected and thereafter 5 ml of buffer B (10%) was passed through the column, which hereafter was washed with a linear gradient of buffer B from 10 to 44% over 4.5 ml and held at 44% buffer B for another 6.75 ml. The gradient was then ramped to 100% buffer B over 5 ml and held for further 8 ml. The identity of the eluted peaks was also established by recording the absorption spectrum.

### Crystallography

After re-suspension of lyophilized protein into 150 mM NaCl, 20 mM Tris-HCl, pH 7.4 buffer, crystallization screening was carried out at 292 K (vapour-diffusion in sitting-drops), with commercially available crystallization kits. For screening, a Mosquito crystallization robot from TTP Labtech was employed (150 nl+150 nl drops equilibrated against 70 μl). Once the crystallization conditions were established, a scale-up was performed (hanging drops mixing 2 μl protein solution (10 mg ml^−1^) with 2 μl reservoir solution and equilibrated against 500 μl reservoir solution).

*Pf*GATase crystals suitable for X-ray analysis grew within few days in a solution containing 0.2 M lithium nitrate and 20% (w/v) PEG3350. For cryoprotection, crystals were soaked in a reservoir solution supplemented with 10% (v/v) ethylene glycol during 2–5 min. X-ray diffraction data were collected at beamline ID14-EH4 at the ESRF (Grenoble, France), at a wavelength of 0.93928 Å. *Pf*GATase crystals diffracted X-rays to 2.60 Å resolution, and were indexed and scaled with the program XDS^30^.

The crystal structure of the *Pf*GATase domain was determined by the molecular replacement method with the program PHENIX^31^ using the refined structure of the GATase domain from *E. coli*^11^ as search model. The structure was refined with PHENIX^31^, and visualized with COOT^32^. The refined structure was validated with PROCHECK^33^ before depositing.

Crystals of *Pf*GMPS_C89A in complex with Glutamine were obtained by the hanging drop vapour diffusion method at 292 K, in 0.1 M Tris-HCl pH 8.5, 22.5% (w/v) PEG 1000 and 10 mM Gln. Ligand-free *Pf*GMPS crystallized in 20% (w/v) PEG 3350, 0.2 M Diammonium tartrate and 5 mM NaCl.

*Pf*GMPS_C89A/Gln and *Pf*GMPS crystals were flash-cooled in a stream of nitrogen gas after soaking for few minutes in their respective reservoir solutions supplemented with 10% (v/v) ethylene glycol before data collection. A complete data set was collected to 3.15 Å resolution for the *Pf*GMPS_C89A/Gln complex. Data on the ligand-free enzyme was collected to 3.6 Å resolution. All data were processed with programs from the XDS package^30^. Initial phases for *Pf*GMPS_C89A/Gln were obtained by molecular replacement with residues 6–233 and 235–555 from PDB 3UOW as independent search models, yielding an initial map of suitable quality for iterative, manual model building in COOT^32^ with interspersed cycles of automated refinement and phase improvement in PHENIX^31^. For *Pf*GMPS, molecular replacement was done using *Ec*GMPS^11^ as a template. Root mean square deviations calculated from Cα's with the program SuperPose (1.0)^34^.

### Small angle X-ray scattering

SAXS data (*Pf*GMPS and *Pf*GMPS_C89A) were collected at the ESRF BM29 beamline on samples concentrated between 1.1 and 11 mg ml^−1^ in 20 mM Tris pH 7.4, 50 mM NaCl. As no concentration dependence effects were observed, data at 11 mg ml^−1^ were processed for further analysis. Initial processing was done using PRIMUS^35^ and P(r) analysis carried out using GNOM^36^. *Ab initio* models of *Pf*GMPS or *Pf*GMPS_C89A were generated from the experimental data using DAMMIF^37^. Models were superimposed onto the crystal structures using SUPCOMB^38^.

### Characterization of GATase domain rotation

Two dynamic domains, which correspond to the GATase and ATPPase structural domains, were identified by the program DynDom^39^ from pdb files (*Pf*GMPS and *Pf*GMPS_C89A/Gln) containing the monomers of each structure. Hereafter the ‘SSM superimpose^40^' option in COOT^32^ was used to superimpose *Pf*GMPS and *Pf*GMPS_C89A/Gln structures, holding the ATPPase domain fixed. This is in agreement with the experimental SAXS and crystal structures which clearly shows that the dimerization takes place via the ATPPase domain. After superposing the ATPPase domains the change in orientation of the GATase domains in the *Pf*GMPS and *Pf*GMPS_C89A/Gln structures was established using the ‘SSM' option in COOT^32^, which gave the rotation matrix and translation vector from which the polar rotation angles and translation along the rotation axis (3 Å) were obtained (*ω*=34° (angle with the *z*-axis); *ϕ*=−158° (angle between the *x*- and the *y*-axis); *κ*=85° (rotation angle)). The rotation axis runs through Lys104 and Ile29.

### Molecular dynamics and minimization

Minimizations of each model (>10,000 steps with the conjugated gradient algorithm) were done using the Sybyl-X 2.0 software package, elaborated by Tripos (now CERTERA). The Tripos force field was applied with Gasteiger–Marsilli partial charges and a dielectric constant of 80 to simulate an implicit water phase (the dielectric constant of water is 20.10 at 20 °C). No restraints were applied to our models. This step principally refines and corrects the positions of amino acid side chains.

The resulting model was inserted into a parapipedic TIP3P solvent box by using the add solvation box module of the VMD 1.9.1 software. A distance of 15 Å was used for delimiting surfaces of the protein and that of the solvent box. Conditions for neutralizing charges were computed before the adding of sodium and chloride ions at concentrations similar to the salt concentrations used in the enzymatic assays. One thousand minimization steps of the model were done using the NaMD 2.8b software^41^ before molecular dynamics simulations. Two-dimensional root mean square calculations were performed to determine three key conformation frames resulting from each of the eight dynamics, and selected on the basis of its position along the rotation pathway. Alanine scanning was computed and ΔΔG variations for each residue were evaluated for each of the three selected key frames. Then the mean value of the ΔΔG of these latter was calculated for each of the eight conformation frames. The lowest energy conformation, frame number 8 (final conformation), was set to 0 kcal mol^−1^. Computing was performed on a 144 xeon core CPU cluster supercomputer (SGI Altix), and simulations were done at constant temperature (300 K) and pressure (1 atm) by implementing the widely used CHARMM 27 force fields. The time step was set to 1 fs and Langevin dynamics was performed with a target piston pressure of 1 atm and a damping coefficient of 5 ps^−1^. There is no coupling of the Langevin temperature with hydrogen. Particle Mesh Ewald (PME) algorithms were applied with a grid extended by 10 Å from the periodic boundary condition size^42^. The electrostatic cut-off was set to 14 Å. A conformation was sampled every 10 ps. As the solvent was described, the dielectric constant was set to 1. To identify steady conformations, Two-dimensional RMSD calculations were carried out on 100 conformations selected, with a stride of 10, from the 1,000 conformations produced during the 10 ns simulation. The equilibrium state was reached around 30 ps for all studied models.

Alanine scanning was calculated with the foldX 3.0b5.1 program for each steady conformation for all eight models with default values of parameters.

### Figure rendering

Figures were rendered using PyMOL^43^.

## Additional information

**Accession codes:** atomic coordinates and structure factors have been deposited in the Protein Data Bank, www.pdb.org (PDB-ID codes: 4WIN (*Pf*GATase), 4WIM (*Pf*GMPS) and 4WIO (*Pf*GMPS_C89A/Gln)).

**How to cite this article:** Ballut, L. *et al*. Active site coupling in *Plasmodium falciparum* GMP Synthetase is triggered by domain rotation. *Nat. Commun.* 6:8930 doi: 10.1038/ncomms9930 (2015).

## Supplementary Material

Supplementary Information Supplementary Figures 1-7 and Supplementary Tables 1-2

## Figures and Tables

**Table 1:** Data collection and refinement statistics.

	***Pf*****GMPS_C89A/Gln**	***Pf*****GMPS**	***Pf*****GATase**
PDB-ID	4WIO	4WIM	4WIN
*Data collection*			
Beamline	SOLEIL-PROXIMA1	ESRF-ID29	ESRF-ID14-EH4
Wavelength (Å)	1.07812	0.97239	0.93928
Temperature (K)	100	100	100
Space group	*P*4_3_2_1_2	*P*1	*P*2_1_2_1_2_1_
Cell dimensions			
*a*, *b*, *c* (Å)	76.9, 76.9, 204.8	43.5, 68.5, 102.1	70.8, 74.2, 91.6
*α*, *β*, *γ* (°)	90, 90, 90	101.1, 99.2, 93.7	90, 90, 90
Resolution (Å)	3.15	3.6	2.6
Unique reflections	11311	11730	15347
*R*_sym_ (%)*†	11.5 (37.6)	5.7 (64.1)	5.7 (37.8)
*I/σ*(*I*)*	10.13 (3.92)	7.18 (1.34)	22.42 (4.67)
Completeness (%)*	99.9 (100)	88.8 (84)	99.6 (99.8)
Redundancy*	7.5 (7.5)	1.7 (1.7)	5.1 (5)
N° mol. / asym. unit	1	2	2
			
*Model refinement*			
*R*_work_/*R*_free_ (%)‡	26.5/30.6	28.2/29.0	20.6/25.5
No. atoms			
Protein	4024	7293	3575
Ligand/ion	10	NA	12
Water	3	27	93
*B* factors (Å^2^)			
Protein	66.7	66.3	62.3
Ligand/ion	76.7	NA	48.7
Water	29.0	52.7	43.3
ESU (Å)§	0.44	0.35	0.33
r.m.s. deviations			
Bond lengths (Å)	0.003	0.035	0.002
Bond angles (°)	0.64	3.46	0.53

ESU, estimated overall coordinate error; r.m.s., root mean square.

All diffraction data were obtained from a single crystal.

^*^Highest resolution shell is shown in parentheses.

^†^*R*_sym_=Σ_hkl_Σ_i_|I_hkl_,_i_−<I>_hkl_|/Σ_hkl_Σ_i_|_Ihkl_,_i_|, where I_hkl_ is the intensity of a reflection and <I>_hkl_ is the average of all observations of the reflection.

^‡^*R*_free_, *R*_factor_ calculated from 5% of the data excluded from refinement.

^§^ESU based on maximum likelihood.

**Table 2:** Steady-state kinetic parameters for *Pf*GMPS and mutants*.

**Varied substrate**		***Pf*****GMPS**	**D172A**	**C89A**	**C89A C113A**	**Y212W**	**E374L**
Q	*K*_m_ (mM)	0.36±0.03	62.2±3.3	NA	NA	1.01±0.04	NA
	*k*_cat_ (min^−1^)	37±3	36.4±0.8			54±1	
	*k*_cat_/*K*_m_ (mM^−1^ min^−1^)	103	0.6			53	
NH_4_Cl	*K*_m_ (mM)	19±1	26.2±4.5	8±1	2.8±0.3	19±1	169±5
	*k*_cat_ (min^−1^)	55±1	66.1±4.5	63±2	49.5±0.9	58±1	6.4±0.4
	*k*_cat_/*K*_m_ (mM^−1^ min^−1^)	2.9	2.5	7.9	17.7	3.0	0.04
ATP	*K*_m_ (μM)	102±5	ND	240±17	297±17	ND	ND
	*k*_cat_ (min^−1^)	43±1		84±1	74±1		
	*k*_cat_/*K*_m_ (mM^−1^ min^−1^)	422		350	249		
XMP	*K*_m_ (μM)	10±2	ND	18±1	19±1.9	ND	ND
	*k*_cat_ (min^−1^)	44±1		90±2	101±4		
	*k*_cat_/*K*_m_ (mM^−1^ min^−1^)	4400		5000	5316		

NA, not applicable; ND, not determined.

The specific activity of *Pf*GMPS_D371A was 2 nmol min^−1^ mg^−1^ for NH_4_Cl-dependent GMP formation. The activity of *Pf*GMPS_D371A could be measured when the concentration of the enzyme in the assay was 18 μM that is 20-fold higher than the concentration of the wild-type enzyme.

All data are expressed as mean± s.e.m. of the mean of two independent measurements.

^*^In all cases, the assay involved monitoring GMP formation as drop in absorbance at 290 nm. The steady-state kinetic parameters for ATP and XMP for *Pf*GMPS are for Gln-dependent GMP formation while for the mutants the parameters are for NH_4_Cl-dependent activity. The concentration of the fixed substrates were 2 mM ATP, 150 μM XMP, 5mM Gln and 100mM NH_4_Cl. The reactions were carried out in 90 mM Tris-HCl, pH 8.5, 20 mM MgCl_2_, 0.1 mM EDTA and 0.1 mM DTT at 25 °C.

## References

[b1] Gardner M. J. . Genome sequence of the human malaria parasite *Plasmodium falciparum*. Nature 419, 498–511 (2002) . 12368864 10.1038/nature01097 PMC3836256

[b2] Downie M. J., Kirk K. & Mamoun C. B. Purine salvage pathways in the intraerythrocytic malaria parasite *Plasmodium falciparum*. Eukaryot. Cell 7, 1231–1237 (2008) . 18567789 10.1128/EC.00159-08 PMC2519781

[b3] Mehrotra S., Mylarappa B. N., Iyengar P. & Balaram H. Studies on active site mutants of *P. falciparum* adenylosuccinate synthetase: insights into enzyme catalysis and activation. Biochim. Biophys. Acta 1804, 1996–2002 (2010) . 20654742 10.1016/j.bbapap.2010.07.015

[b4] McConkey G. A. *Plasmodium falciparum*: isolation and characterisation of a gene encoding protozoan GMP synthase. Exp. Parasitol. 94, 23–32 (2000) . 10631077 10.1006/expr.1999.4467

[b5] Massiere F. & Badet-Denisot M. A. The mechanism of glutamine-dependent amidotransferases. Cell. Mol. Life Sci. 54, 205–222 (1998) . 9575335 10.1007/s000180050145 PMC11147313

[b6] Bhat J. Y. . Ammonia channeling in *Plasmodium falciparum* GMP synthetase: investigation by NMR spectroscopy and biochemical assays. Biochemistry 50, 3346–3356 (2011) . 21413787 10.1021/bi1017057

[b7] Mouilleron S. & Golinelli-Pimpaneau B. Conformational changes in ammonia-channeling glutamine amidotransferases. Curr. Opin. Struct. Biol. 17, 653–664 (2007) . 17951049 10.1016/j.sbi.2007.09.003

[b8] Bhat J. Y., Shastri B. G. & Balaram H. Kinetic and biochemical characterization of *Plasmodium falciparum* GMP synthetase. Biochem. J. 409, 263–273 (2008) . 17868038 10.1042/BJ20070996

[b9] Bhat J. Y., Venkatachala R. & Balaram H. Substrate-induced conformational changes in *Plasmodium falciparum* guanosine monophosphate synthetase. FEBS J. 278, 3756–3768 (2011) . 21827625 10.1111/j.1742-4658.2011.08296.x

[b10] Maruoka S., Horita S., Lee W. C., Nagata K. & Tanokura M. Crystal structure of the ATPPase subunit and its substrate-dependent association with the GATase subunit: a novel regulatory mechanism for a two-subunit-type GMP synthetase from *Pyrococcus horikoshii* OT3. J. Mol. Biol. 395, 417–429 (2010) . 19900465 10.1016/j.jmb.2009.10.053

[b11] Tesmer J. J., Klem T. J., Deras M. L., Davisson V. J. & Smith J. L. The crystal structure of GMP synthetase reveals a novel catalytic triad and is a structural paradigm for two enzyme families. Nat. Struct. Biol. 3, 74–86 (1996) . 8548458 10.1038/nsb0196-74

[b12] Welin M. . Substrate specificity and oligomerization of human GMP synthetase. J. Mol. Biol. 425, 4323–4333 (2013) . 23816837 10.1016/j.jmb.2013.06.032

[b13] Oliver J. C. . Conformational changes involving ammonia tunnel formation and allosteric control in GMP synthetase. Arch. Biochem. Biophys. 545, 22–32 (2014) . 24434004 10.1016/j.abb.2014.01.004 PMC3954777

[b14] Amuro N., Paluh J. L. & Zalkin H. Replacement by site-directed mutagenesis indicates a role for histidine 170 in the glutamine amide transfer function of anthranilate synthase. J. Biol. Chem. 260, 14844–14849 (1985) . 3902841

[b15] Miran S. G., Chang S. H. & Raushel F. M. Role of the four conserved histidine residues in the amidotransferase domain of carbamoyl phosphate synthetase. Biochemistry 30, 7901–7907 (1991) . 1868065 10.1021/bi00246a005

[b16] Franco T. M. . Biochemical characterization of recombinant guaA-encoded guanosine monophosphate synthetase (EC 6.3.5.2) from *Mycobacterium tuberculosis* H37Rv strain. Arch. Biochem. Biophys. 517, 1–11 (2012) . 22119138 10.1016/j.abb.2011.11.013

[b17] Chaudhuri B. N. . Crystal structure of imidazole glycerol phosphate synthase: a tunnel through a (beta/alpha)8 barrel joins two active sites. Structure 9, 987–997 (2001) . 11591353

[b18] Chaudhuri B. N., Lange S. C., Myers R. S., Davisson V. J. & Smith J. L. Toward understanding the mechanism of the complex cyclization reaction catalyzed by imidazole glycerolphosphate synthase: crystal structures of a ternary complex and the free enzyme. Biochemistry 42, 7003–7012 (2003) . 12795595 10.1021/bi034320h

[b19] List F. . Catalysis uncoupling in a glutamine amidotransferase bienzyme by unblocking the glutaminase active site. Chem. Biol. 19, 1589–1599 (2012) . 23261602 10.1016/j.chembiol.2012.10.012

[b20] Goto M., Omi R., Nakagawa N., Miyahara I. & Hirotsu K. Crystal structures of CTP synthetase reveal ATP, UTP, and glutamine binding sites. Structure 12, 1413–1423 (2004) . 15296735 10.1016/j.str.2004.05.013

[b21] Tanwar A. S., Goyal V. D., Choudhary D., Panjikar S. & Anand R. Importance of hydrophobic cavities in allosteric regulation of formylglycinamide synthetase: insight from xenon trapping and statistical coupling analysis. PLoS One 8, e77781 (2013) . 24223728 10.1371/journal.pone.0077781 PMC3815217

[b22] Chittur S. V., Klem T. J., Shafer C. M. & Davisson V. J. Mechanism for acivicin inactivation of triad glutamine amidotransferases. Biochemistry 40, 876–887 (2001) . 11170408 10.1021/bi0014047

[b23] Nakamura J., Straub K., Wu J. & Lou L. The glutamine hydrolysis function of human GMP synthetase. Identification of an essential active site cysteine. J. Biol. Chem. 270, 23450–23455 (1995) . 7559506 10.1074/jbc.270.40.23450

[b24] Mouilleron S., Badet-Denisot M. A., Badet B. & Golinelli-Pimpaneau B. Dynamics of glucosamine-6-phosphate synthase catalysis. Arch. Biochem. Biophys. 505, 1–12 (2011) . 20709015 10.1016/j.abb.2010.08.008

[b25] Shenoy A. R. & Visweswariah S. S. Site-directed mutagenesis using a single mutagenic oligonucleotide and DpnI digestion of template DNA. Anal. Biochem. 319, 335–336 (2003) . 12871732 10.1016/s0003-2697(03)00286-0

[b26] Moyed H. S. & Magasanik B. Enzymes essential for the biosynthesis of nucleic acid guanine; xanthosine 5′-phosphate aminase of *Aerobacter aerogenes*. J. Biol. Chem. 226, 351–363 (1957) . 13428768

[b27] Bulusu V., Jayaraman V. & Balaram H. Metabolic fate of fumarate, a side product of the purine salvage pathway in the intraerythrocytic stages of *Plasmodium falciparum*. J. Biol. Chem. 286, 9236–9245 (2011) . 21209090 10.1074/jbc.M110.173328 PMC3059058

[b28] Miles B. W. & Raushel F. M. Synchronization of the three reaction centers within carbamoyl phosphate synthetase. Biochemistry 39, 5051–5056 (2000) . 10819970 10.1021/bi992772h

[b29] Oliver J. C., Linger R. S., Chittur S. V. & Davisson V. J. Substrate activation and conformational dynamics of guanosine 5′-monophosphate synthetase. Biochemistry 52, 5225–5235 (2013) . 23841499 10.1021/bi3017075 PMC3859818

[b30] Kabsch W. XDS. Acta Crystallogr. D Biol. Crystallogr. 66, 125–132 (2010) . 20124692 10.1107/S0907444909047337 PMC2815665

[b31] Adams P. D. . PHENIX: a comprehensive Python-based system for macromolecular structure solution. Acta Crystallogr. D Biol. Crystallogr. 66, 213–221 (2010) . 20124702 10.1107/S0907444909052925 PMC2815670

[b32] Emsley P., Lohkamp B., Scott W. G. & Cowtan K. Features and development of Coot. Acta Crystallogr. D Biol. Crystallogr. 66, 486–501 (2010) . 20383002 10.1107/S0907444910007493 PMC2852313

[b33] Laskowski R. A., Macarthur M. W., Moss D. S. & Thornton J. M. PROCHECK: a program to check the stereochemical quality of protein structures. J. Appl. Crystallogr. 26, 283–291 (1993) .

[b34] Maiti R., Van Domselaar G. H., Zhang H. & Wishart D. S. SuperPose: a simple server for sophisticated structural superposition. Nucleic Acids Res. 32, W590–W594 (2004) . 15215457 10.1093/nar/gkh477 PMC441615

[b35] Konarev P. V., Volkov V. V., Sokolova A. V., Koch M. H. J. & Svergun D. I. PRIMUS: a Windows PC-based system for small-angle scattering data analysis. J. Appl. Crystallogr. 36, 1277–1282 (2003) .

[b36] Svergun D. Determination of the regularization parameter in indirect-transform methods using perceptual criteria. J. Appl. Crystallogr. 25, 495–503 (1992) .

[b37] Franke D. & Svergun D. I. DAMMIF a program for rapid ab-initio shape determination in small-angle scattering. J. Appl. Crystallogr. 42, 342–346 (2009) . 10.1107/S0021889809000338 PMC5023043 27630371

[b38] Kozin M. B. & Svergun D. I. Automated matching of high- and low-resolution structural models. J. Appl. Crystallogr. 34, 33–41 (2001) .

[b39] Hayward S. & Berendsen H. J. Systematic analysis of domain motions in proteins from conformational change: new results on citrate synthase and T4 lysozyme. Proteins 30, 144–154 (1998) . 9489922

[b40] Krissinel E. & Henrick K. Secondary-structure matching (SSM), a new tool for fast protein structure alignment in three dimensions. Acta Crystallogr. D. Biol. Crystallogr. 60, 2256–2268 (2004) . 15572779 10.1107/S0907444904026460

[b41] Phillips J. C. . Scalable molecular dynamics with NAMD. J. Comput. Chem. 26, 1781–1802 (2005) . 16222654 10.1002/jcc.20289 PMC2486339

[b42] Darden T., Perera L., Li L. & Pedersen L. New tricks for modelers from the crystallography toolkit: the particle mesh Ewald algorithm and its use in nucleic acid simulations. Structure 7, R55–R60 (1999) . 10368306 10.1016/s0969-2126(99)80033-1

[b43] DeLano W. L. PyMOL 0.99.rc6 Palo Alto, CA, USA (2002) .

