# Supplementary material for: Active site coupling in Plasmodium falciparum GMP synthetase is triggered by domain rotation
Source: Nat Commun. 2015 Nov 23;6:8930. doi: 10.1038/ncomms9930 (PMC4673825; doi:10.1038/ncomms9930)
Supplement: Supplementary Information — Supplementary Figures 1-7 and Supplementary Tables 1-2 [file ncomms9930-s1.pdf]

a

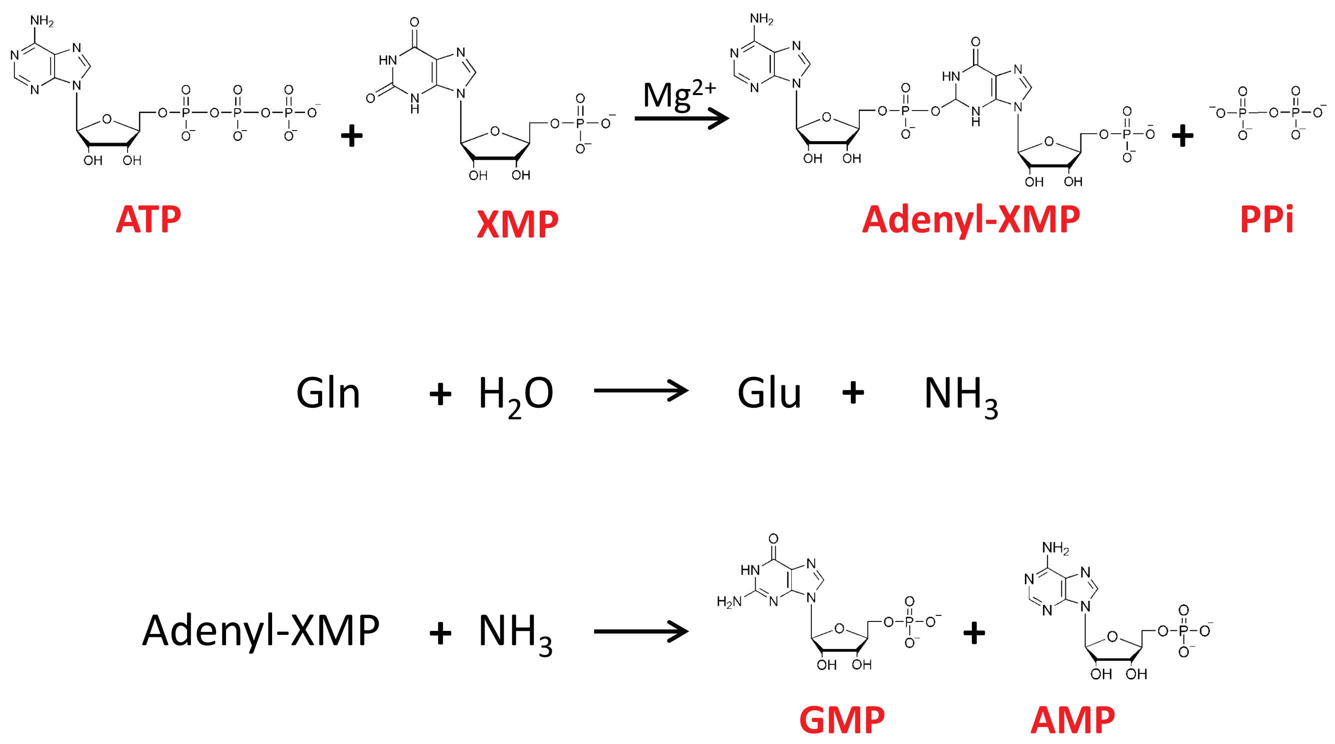

b

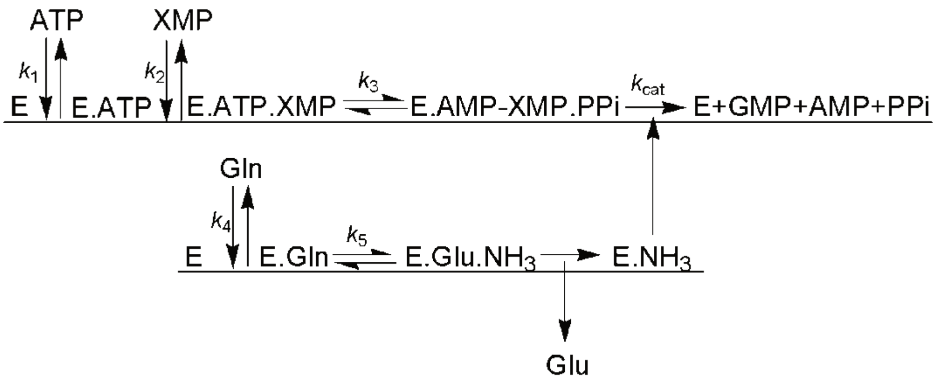

k_{cat}	$37 \pm 3 min^{-1}$	Steady-state k_{cat}
k_3	$156 \pm 5\ min^{-1}$	From nucleotide-only stopped-flow assay
	$111 \pm 2\ min^{-1}$	Stopped flow assay in presence of ATP+XMP+Gln
k_5	$60 \pm 13\ min^{-1}$	Gln hydrolysis in absence of ATP + XMP at 50 mM Gln
	$76 \pm 7\ min^{-1}$	Gln hydrolysis by D371A in presence of ATP + XMP at 5 mM Gln

Supplementary Figure 1. (**a**) *Pf*GMPS-catalyzed reactions (**b**) Scheme of enzymatic mechanism with associated k_{cat} values.

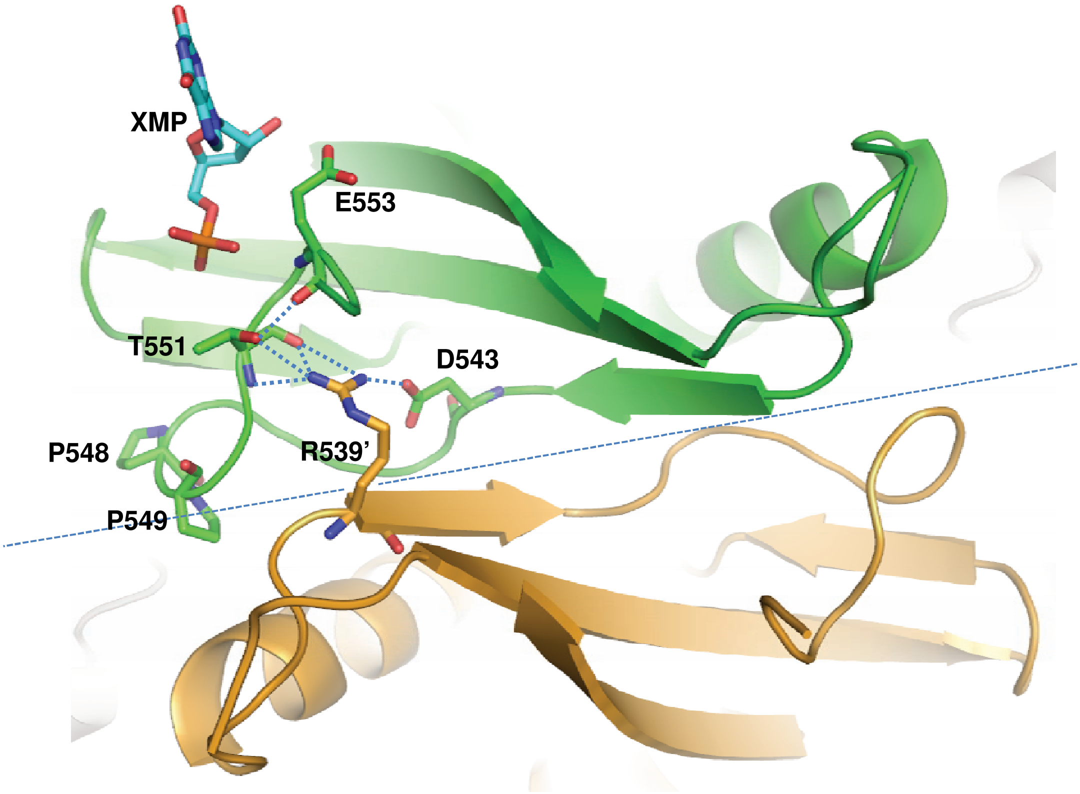

Supplementary Figure 2. *Pf*GMPS dimeric interface. Dimer interface formed by two ATPPase sub-domains in green and orange, respectively. The prime denotes a residue from the other subunit.

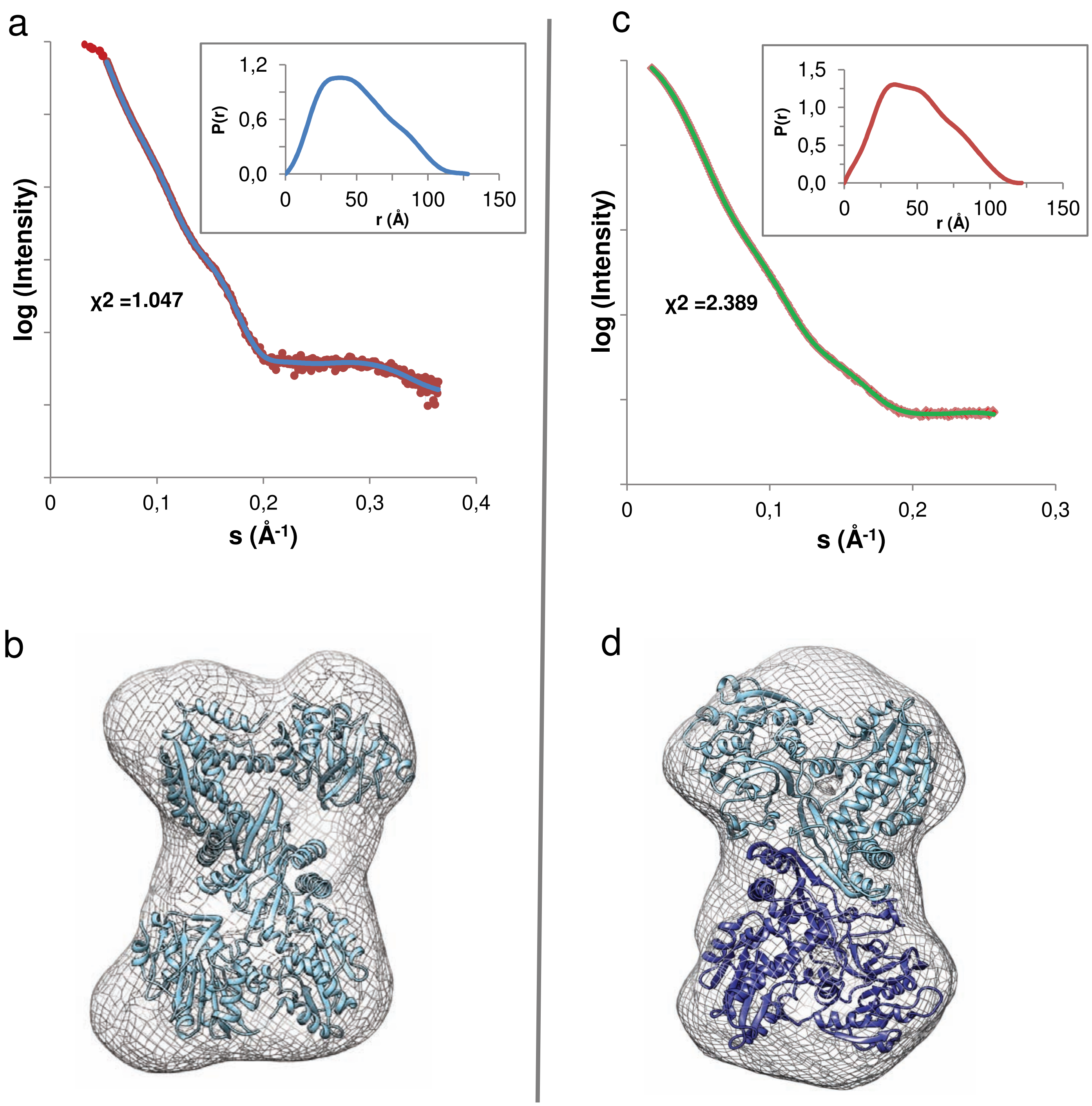

Supplementary Figure 3. Structure of *Pf*GMPS and *Pf*GMPS_C89A in solution. (**a**) Experimental SAXS data of *Pf*GMPS (red dots) fitted to the theoretical scattering curve calculated from the *Pf*GMPS crystal structure with the program DAMMIF (blue). Inset: the corresponding pair-distance distribution functions. Indirect Fourier transformation of the data indicated a Dmax of 128 Å and an Rg of 39.1 Å. (**b**) *Pf*GMPS_C89A/Gln crystal structure superimposed onto the averaged *ab initio* shape reconstructed with DAMMIF. (**c**) Experimental SAXS data of *Pf*GMPS_C89A (orange dots) fitted to the theoretical scattering curve calculated from the *Pf*GMPS_C89A/Gln crystal structure with the program DAMMIF (green). Inset: the corresponding pair-distance distribution functions. Indirect Fourier transformation of the data indicated a Dmax of 122 Å and an Rg of 39.1 Å. (**d**) *Pf*GMPS_C89A/Gln crystal structure superimposed onto the averaged *ab initio* shape reconstructed with DAMMIF. Monomers are depicted in blue and cyan, respectively.

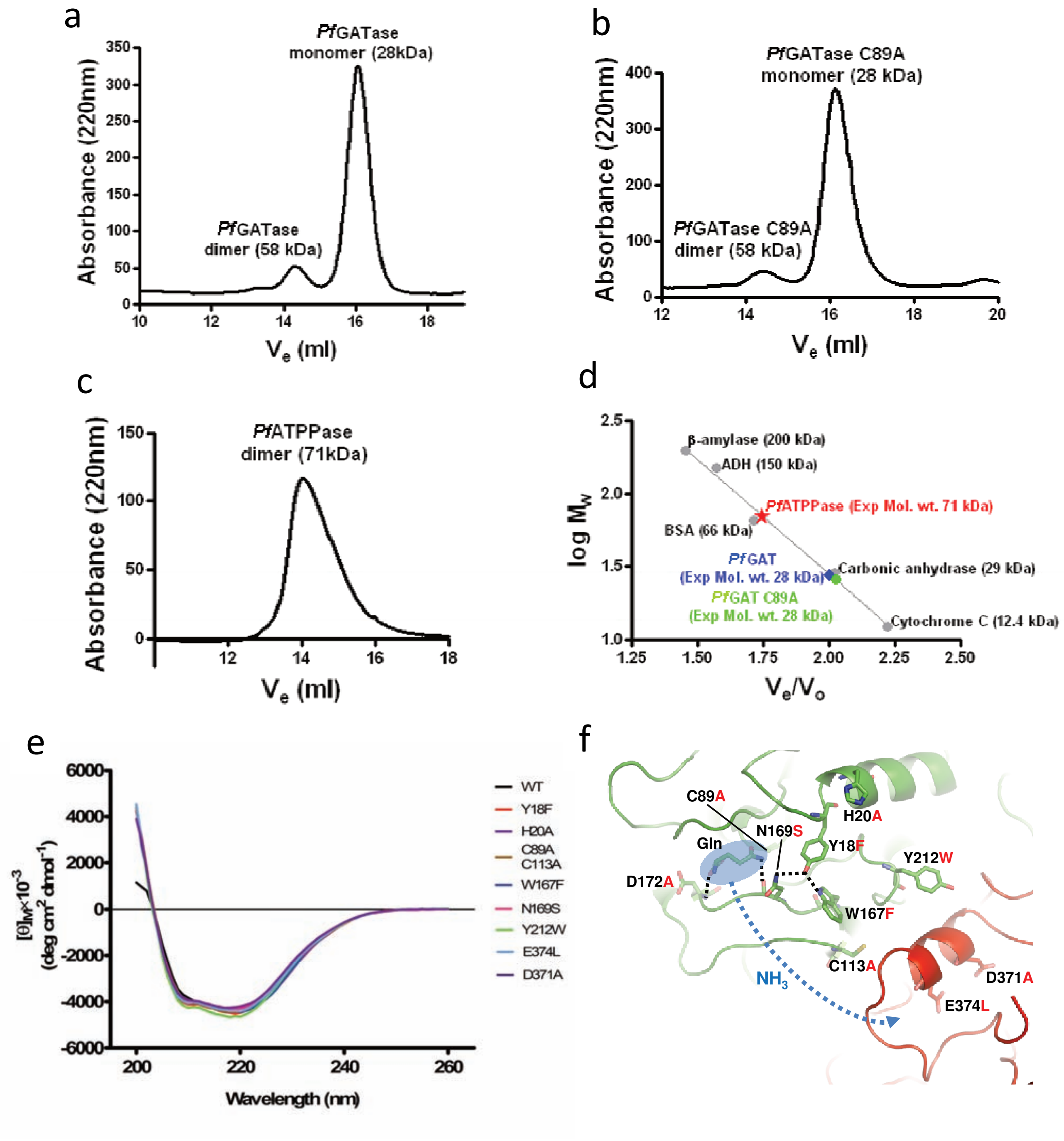

Supplementary Figure 4. Biophysical structural characterization of *Pf*GMPS full-length enzyme, domains and mutants. Determination of the oligomeric states of *Pf*GATase (**a**), *Pf*GATase_C89A mutant (**b**) and *Pf*ATPPase (**c**) by size-exclusion chromatography. Samples were analyzed using a HR 10/30 Superdex200 column. Buffer used was 50 mM Tris-HCl, pH 7.4 and 100 mM KCl. A sample volume of 100 µl containing 20 µM of *Pf*GATase and *Pf*ATPPase was injected into the column, eluted at a flow rate of 0.5 ml/min and detected at 220 nm. (**d**) The calibration curve of the column using molecular weight standards. Ve/Vo values for *Pf*GATase, *Pf*ATPPase and *Pf*GATase_C89A are also indicated on the plot. (**e**) Far-UV circular dichroism spectra of *Pf*GMPS wild-type and mutants. The spectra were recorded at a protein concentration of 5 µM in 7 mM Tris-HCl, pH 7.4, 3.3% (v/v) glycerol, 0.3 mM EDTA and 0.6 mM DTT. A cuvette of 1 mm path length was used. Each spectrum was an average of three scans. (**f**) Hydrogen bond network between the side-chains of Tyr18, Asn169 and Trp167 and the glutamine substrate. GATase and ATPPase domains are depicted in green and red, respectively.

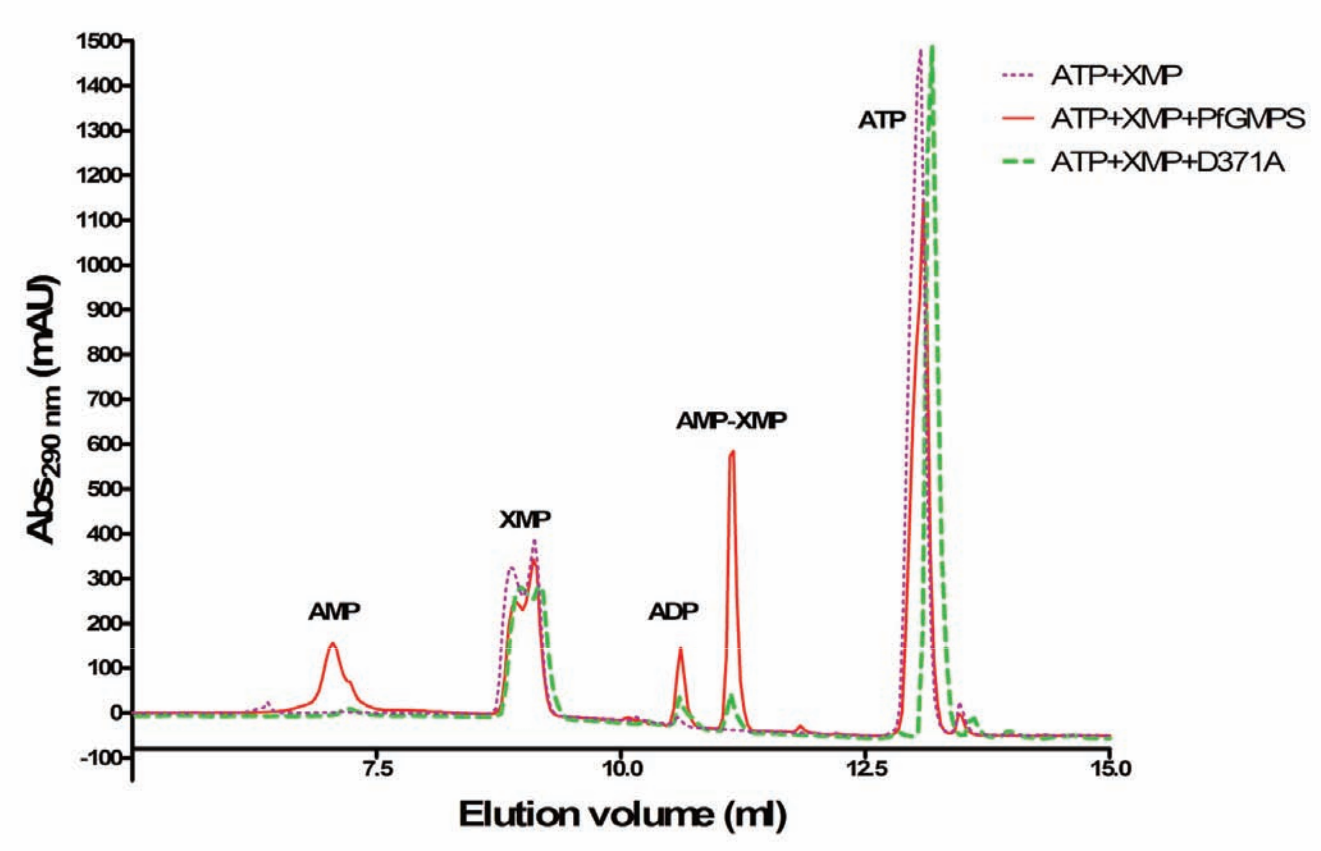

Supplementary Figure 5. Impaired ability of *Pf*GMPS_D371A to form the adenyl-XMP intermediate. 100 µM wild-type *Pf*GMPS or *Pf*GMPS_D371A were incubated in a 250 µl reaction mixture containing 20 mM Tris-HCl, pH 8.5, 250 µM ATP, 150 µM XMP and 5 mM $MgCl_2$ for 10 min at 25 °C. The samples were processed as described in methods and analyzed by reverse-phase HPLC on C18 column. The mobile-phase consisted of 50 mM potassium phosphate, pH 6.2 containing 4 mM tetrabutylammonium hydrogen sulphate as ion-pairing agent, as buffer A and buffer A containing 50 % (v/v) acetonitrile as buffer B.

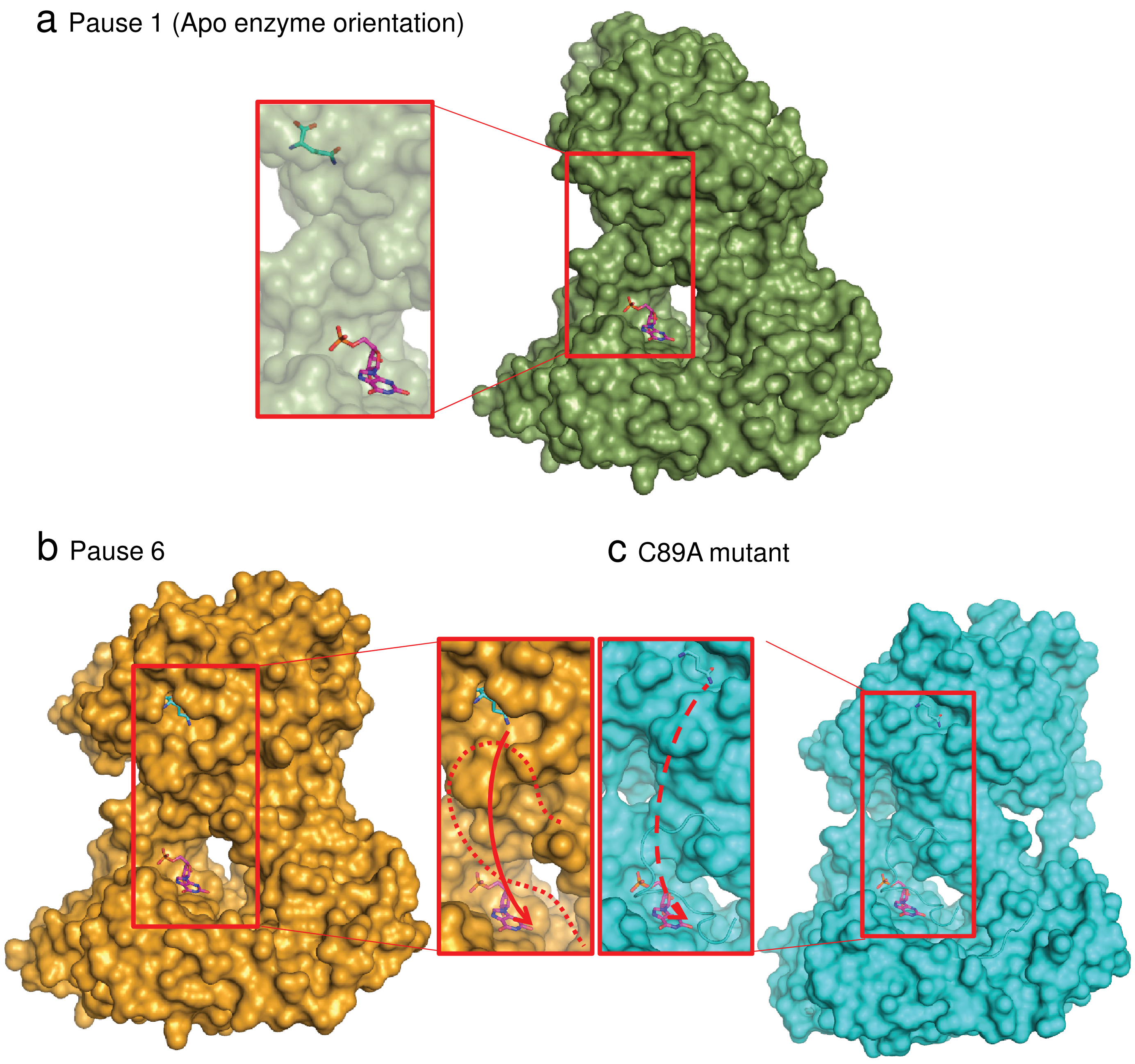

Supplementary Figure 6. Orientation of the two catalytic sites upon rotation of the GATase domain. Three possible orientations are represented: (**a**) the apo/XMP bound enzyme (0°/un-rotated enzyme), (**b**) an intermediate orientation suggested and obtained by molecular dynamics (60° rotated enzyme, corresponding to pause 6 of the dynamics study), and (**c**) the glutamine bound mutant enzyme (85° rotated enzyme). (**a**) Surface presentation showing that the two active sites are not properly oriented for channeling. (**b**) The plain arrow indicates the shortest distance between the two active sites observed for the 60° rotated enzyme, and illustrates a channel pathway. The dotted line is a suggestion of the loop position when in presence of substrates in the ATPPase domain (here only XMP has been shown). (**c**) The stippled arrow shows an increased distance between the GATase and ATPPase active sites compared to the previous orientation as described in (**b**).

```
                                                                                    1       10         20
GUAA_ECO55   ........................................................................MTENIHKHRILILDFGSQYTQL
GUAA_ECO45   ........................................................................MTENIHKHRILILDFGSQYTQL
GUAA_ECO57   ........................................................................MTENIHKHRILILDFGSQYTQL
GUAA_ECO7I   ........................................................................MTENIHKHRILILDFGSQYTQL
GUAA_ECO5E   ........................................................................MTENIHKHRILILDFGSQYTQL
GUAA_ECO81   ........................................................................MTENIHKHRILILDFGSQYTQL
GUAA_ECO8A   ........................................................................MTENIHKHRILILDFGSQYTQL
GUAA_ECOHS   ........................................................................MTENIHKHRILILDFGSQYTQL
GUAA_ECOK1   ........................................................................MTENIHKHRILILDFGSQYTQL
GUAA_ECOL5   ........................................................................MTENIHKHRILILDFGSQYTQL
GUAA_ECOL6   ........................................................................MTENIHKHRILILDFGSQYTQL
GUAA_ECOLC   ........................................................................MTENIHKHRILILDFGSQYTQL
GUAA_ECOSE   ........................................................................MTENIHKHRILILDFGSQYTQL
GUAA_ECOSM   ........................................................................MTENIHKHRILILDFGSQYTQL
GUAA_ECOUT   ........................................................................MTENIHKHRILILDFGSQYTQL
GUAA_ECO24   ........................................................................MTENIHKHRILILDFGSQYTQL
GUAA_SHISS   ........................................................................MTENIHKHRILILDFGSQYTQL
GUAA_SHIF8   ........................................................................MTENIHKHRILILDFGSQYTQL
GUAA_SHIFL   ........................................................................MTENIHKHRILILDFGSQYTQL
GUAA_SHIDS   ........................................................................MTENIHKHRILILDFGSQYTQL
GUAA_ECOLI   ........................................................................MTENIHKHRILILDFGSQYTQL
GUAA_ECODH   ........................................................................MTENIHKHRILILDFGSQYTQL
GUAA_ECOBW   ........................................................................MTENIHKHRILILDFGSQYTQL
GUAA_ESCF3   ........................................................................MTENIHKHRILILDFGSQYTQL
GUAA_ECO27   ........................................................................MTENIHKHRILILDFGSQYTQL
GUAA_SHIB3   ........................................................................MTENIHKHRILILDFGSQYTQL
GUAA_SHIBS   ........................................................................MTENIHKHRILILDFGSQYTQL
GUAA_ECOLU   ........................................................................MTENIHKHRILILDFGSQYTQL
GUAA_SALPK   ........................................................................MTENIHKHRILILDFGSQYTQL
GUAA_SALPC   ........................................................................MTENIHKHRILILDFGSQYTQL
GUAA_SALPB   ........................................................................MTENIHKHRILILDFGSQYTQL
GUAA_SALPA   ........................................................................MTENIHKHRILILDFGSQYTQL
GUAA_SALCH   ........................................................................MTENIHKHRILILDFGSQYTQL
GUAA_SALTY   ........................................................................MTENIHKHRILILDFGSQYTQL
GUAA_SALSV   ........................................................................MTENIHKHRILILDFGSQYTQL
GUAA_SALNS   ........................................................................MTENIHKHRILILDFGSQYTQL
GUAA_SALTI   ........................................................................MTENIHKHRILILDFGSQYTQL
GUAA_SALHS   ........................................................................MTENIHKHRILILDFGSQYTQL
GUAA_SALG2   ........................................................................MTENIHKHRILILDFGSQYTQL
GUAA_SALEP   ........................................................................MTENIHKHRILILDFGSQYTQL
GUAA_SALDC   ........................................................................MTENIHKHRILILDFGSQYTQL
GUAA_SALA4   ........................................................................MTENIHKHRILILDFGSQYTQL
GUAA_SALAR   ........................................................................MTDNIHKHRILILDFGSQYTQL
GUAA_KLEP7   ........................................................................MTENIHKHRILILDFGSQYTQL
GUAA_KLEP3   ........................................................................MTENIHKHRILILDFGSQYTQL
GUAA_ENT38   ........................................................................MTENIHKHRILILDFGSQYTQL
GUAA_CITK8   ........................................................................MTENIHKHRILILDFGSQYTQL
GUAA_CROS8   ........................................................................MTDNIHKHRILILDFGSQYTQL
GUAA_SODGM   ........................................................................MTENIHKHRILILDFGSQYTQL
GUAA_PECCP   ........................................................................MTQNIHQHRILILDFGSQYTQL
GUAA_PECAS   ........................................................................MTQNIHQHRILILDFGSQYTQL
GUAA_YERPY   ........................................................................MTKNIHKHRILILDFGSQYTQL
GUAA_YERPS   ........................................................................MTKNIHKHRILILDFGSQYTQL
GUAA_YERPB   ........................................................................MTKNIHKHRILILDFGSQYTQL
GUAA_YERP3   ........................................................................MTKNIHKHRILILDFGSQYTQL
GUAA_YERPP   ........................................................................MTKNIHKHRILILDFGSQYTQL
GUAA_YERPN   ........................................................................MTKNIHKHRILILDFGSQYTQL
GUAA_YERPG   ........................................................................MTKNIHKHRILILDFGSQYTQL
GUAA_YERPE   ........................................................................MTKNIHKHRILILDFGSQYTQL
GUAA_YERPA   ........................................................................MTKNIHKHRILILDFGSQYTQL
GUAA_YERE8   ........................................................................MTKNIHKHRILILDFGSQYTQL
GUAA_SERP5   ........................................................................MTKNIHKHRILILDFGSQYTQL
GUAA_PHOLL   ........................................................................MTTNIHQHRILILDFGSQYTQL
GUAA_PROMH   ........................................................................MTANIHNHRILILDFGSQYTQL
GUAA_EDWI9   ........................................................................MTQNIHQDRILILDFGSQYTQL
GUAA_AERHH   ........................................................................MTKDIHQHRILILDFGSQYTQL
GUAA_AERS4   ........................................................................MTKDIHQHRILILDFGSQYTQL
GUAA_TOLAT   ........................................................................MSKNIHKHRILILDFGSQYTQL
GUAA_VIBF1   ........................................................................MTTNIHDQRILILDFGSQYTQL
GUAA_VIBFM   ........................................................................MTTNIHDQRILILDFGSQYTQL
GUAA_ALISL   ........................................................................MTTNIHAQRILILDFGSQYTQL
GUAA_VIBSL   ........................................................................MTKNIHDQRILILDFGSQYTQL
GUAA_VIBCM   ........................................................................MTKNIHDQRILILDFGSQYTQL
GUAA_VIBCH   ........................................................................MTKNIHDQRILILDFGSQYTQL
GUAA_VIBC3   ........................................................................MTKNIHDQRILILDFGSQYTQL
GUAA_VIBVU   ........................................................................MTKNIHDQRILILDFGSQYTQL
GUAA_VIBVY   ........................................................................MTKNIHDQRILILDFGSQYTQL
GUAA_VIBPA   ........................................................................MTKNIHDQRILILDFGSQYTQL
GUAA_VIBCB   ........................................................................MTKNIHDQRILILDFGSQYTQL
GUAA_PHOPR   ......................................................................MTTSTNIHDQRILILDFGSQYTQL
GUAA_COLP3   ........................................................................MSKDIHDHRILILDFGSQYTQL
GUAA_PSYIN   ........................................................................MDKNIHEQRILILDFGSQYTQL
GUAA_PSEHT   ........................................................................MSKDIHDSRILILDFGSQYTQL
GUAA_PSEA6   ........................................................................MTTNIHDQRILILDFGSQYTQL
GUAA_ALTMD   ........................................................................MTTNIHDQRILILDFGSQYTQL
GUAA_SHESW   .........................................................................MSDIHEHKILILDFGSQYTQL
GUAA_SHEPC   .........................................................................MSDIHEHKILILDFGSQYTQL
GUAA_SHEB9   .........................................................................MSDIHEHKILILDFGSQYTQL
GUAA_SHEB8   .........................................................................MSDIHEHKILILDFGSQYTQL
GUAA_SHEB5   .........................................................................MSDIHEHKILILDFGSQYTQL
GUAA_SHEB2   .........................................................................MSDIHEHKILILDFGSQYTQL
GUAA_SHESM   .........................................................................MSDIHEHKILILDFGSQYTQL
GUAA_SHESA   .........................................................................MSDIHEHKILILDFGSQYTQL
GUAA_SHESR   .........................................................................MSDIHEHKILILDFGSQYTQL
GUAA_SHEON   .........................................................................MSDIHEHKILILDFGSQYTQL
GUAA_SHEDO   .........................................................................MSNIHEHKILILDFGSQYTQL
GUAA_SHEPW   .........................................................................MSNIHEHKILILDFGSQYTQL
GUAA_SHESH   .........................................................................MSNIHEHKILILDFGSQYTQL
GUAA_SHEWM   .........................................................................MNNIHDHKILILDFGSQYTQL
GUAA_SHEHH   .........................................................................MSNIHEHKILILDFGSQYTQL
GUAA_SHEPA   .........................................................................MSNIHEHKILILDFGSQYTQL
GUAA_SHEFN   .........................................................................MSNIHDHKILILDFGSQYTQL
GUAA_SHELP   .........................................................................MSNIHDHKILILDFGSQYTQL
GUAA_SHEAM   .........................................................................MSNIHEHKILILDFGSQYTQL
GUAA_IDILO   ........................................................................MTRDIHDHRILILDFGSQYTQL
GUAA_ACTSZ   .........................................................................MDNIHNHKILILDFGSQYTQL
GUAA_MANSM   .........................................................................MNNIHNHKILILDFGSQYTQL
GUAA_ACTPJ   .........................................................................MTNIHNHKILILDFGSQYTQL
GUAA_ACTP7   .........................................................................MTNIHNHKILILDFGSQYTQL
GUAA_ACTP2   .........................................................................MTNIHNHKILILDFGSQYTQL
GUAA_HAEPS   .........................................................................MTNIHNHKILILDFGSQYTQL
GUAA_PASMU   .........................................................................MNNIHNHKILILDFGSQYTQL
GUAA_HAEDU   .........................................................................MNNIHNHKILILDFGSQYTQL
GUAA_HISS1   .........................................................................MTNIHHHKILILDFGSQYTQL
GUAA_HISS2   .........................................................................MTNIHHHKILILDFGSQYTQL
GUAA_HAEI8   .........................................................................MTNIHNHKILILDFGSQYTQL
GUAA_HAEIG   .........................................................................MTNIHNHKILILDFGSQYTQL
GUAA_HAEIN   .........................................................................MTNIHYHKILILDFGSQYTQL
GUAA_HAEIE   .........................................................................MTNIHNHKILILDFGSQYTQL
GUAA_HAMD5   ........................................................................MIKNIDRHRILILDFGSQYTQL
GUAA_XANC5   ........................................................................MT.NIHTDKILILDFGAQYTQL
GUAA_XANAC   ........................................................................MT.NIHTDKILILDFGAQYTQL
GUAA_XANCP   ........................................................................MS.SLHNDKILILDFGAQYTQL
GUAA_XANCB   ........................................................................MS.SLHNDKILILDFGAQYTQL
GUAA_XANC8   ........................................................................MS.SLHNDKILILDFGAQYTQL
GUAA_XANOR   ........................................................................MT.NIHTDKILILDFGAQYTQL
GUAA_XANOM   ........................................................................MT.NIHTDKILILDFGAQYTQL
GUAA_XANOP   ........................................................................MT.NIHTDKILILDFGAQYTQL
GUAA_STRMK   ........................................................................MT.NIHNDKILILDFGAQYTQL
GUAA_STRM5   ........................................................................MT.NIHNDKILILDFGAQYTQL
GUAA_XYLFT   ........................................................................MTPNIHHDKILILDFGAQYTQL
GUAA_XYLF2   ........................................................................MTPNIHHDKILILDFGAQYTQL
GUAA_XYLFM   ........................................................................MTPNIHHDKILILDFGAQYTQL
GUAA_XYLFA   ........................................................................MTPNIHHDKILILDFGAQYTQL
GUAA_CELJU   ........................................................................MTHDIHAQRILILDFGSQYTQL
GUAA_TERTT   ........................................................................MTQNIHSQRVLILDFGSQYTQL
GUAA_HAHCH   ........................................................................MSENIHSHRILILDFGSQYTQL
GUAA_MARAV   ........................................................................MAQNIHDHRILILDFGSQYTQL
GUAA_PSESM   ........................................................................MALDIHAHRILILDFGSQYTQL
GUAA_PSE14   ........................................................................MALDIHAHRILILDFGSQYTQL
GUAA_PSEU2   ........................................................................MALDIHAHRILILDFGSQYTQL
GUAA_PSEFS   ........................................................................MALDIHAHRILILDFGSQYTQL
GUAA_PSEF5   ........................................................................MALDIHAHRILILDFGSQYTQL
GUAA_PSEPF   ........................................................................MALDIHAHRILILDFGSQYTQL
```

```
GUAA_PSEPK    ......................................................................MALDIHAHRILILDFGSQYTQL
GUAA_PSEP1    ......................................................................MALDIHAHRILILDFGSQYTQL
GUAA_PSEPG    ......................................................................MALDIHAHRILILDFGSQYTQL
GUAA_PSEE4    ......................................................................MALDIHAHRILILDFGSQYTQL
GUAA_PSEPW    ......................................................................MALDIHAHRILILDFGSQYTQL
GUAA_PSEMY    ......................................................................MAHDIHAHRILILDFGSQYTQL
GUAA_PSEAE    ......................................................................MSQDIHAHRILILDFGSQYTQL
GUAA_PSEA8    ......................................................................MSQDIHAHRILILDFGSQYTQL
GUAA_PSEAB    ......................................................................MSQDIHAHRILILDFGSQYTQL
GUAA_MARMS    ......................................................................MSQDIHAHKILILDFGSQYTQL
GUAA_ALCBS    .......................................................................MTDIHAQRILILDFGSQYTQL
GUAA_THICR    .....................................................................MSQNNIHEHRILILDFGSQYTQL
GUAA_ALKEH    ......................................................................MTQDIHAHRILILDFGSQYTQL
GUAA_METCA    .......................................................................MTDIHAQKILILDFGSQYTQL
GUAA_NITOC    .......................................................................MSDLYAHRILILDFGSQYTQL
GUAA_LEGPL    .......................................................................MNDLKKSPLLILDFGSQYTQL
GUAA_LEGPA    .......................................................................MNDLKKSPLLILDFGSQYTQL
GUAA_LEGPC    .......................................................................MNDLKKSPLLILDFGSQYTQL
GUAA_LEGPH    .......................................................................MNDLKKSPLLILDFGSQYTQL
GUAA_ACIAD    ........................................MLIERTCAILGKMLEYCASLVALHSCVIMTTNTHITDDRILILDFGSQYSQL
GUAA_PSYA2    ...........................................................MDTLFMTTVTAT.PAIKEDRILILDFGSQYSQL
GUAA_RUTMC    ...................................................................MMNN...IHLDKILILDFGSQYTQL
GUAA_FRAP2    .......................................................................MTDIHNHKILILDFGSQYTQL
GUAA_BURPP    ...........................................................................MHDKILILDFGSQVTQL
GUAA_BURXL    ...........................................................................MHDKILILDFGSQVTQL
GUAA_BURP8    ...........................................................................MHDKILILDFGSQVTQL
GUAA_POLNS    ...........................................................................MHDKILILDFGSQVTQL
GUAA_POLSQ    ...........................................................................MHDKILILDFGSQVTQL
GUAA_DECAR    ..........................................................................MSHQKILILDFGSQVTQL
GUAA_HERAR    ...........................................................................MHSKILILDFGSQVTQL
GUAA_JANMA    ...........................................................................MHSKILILDFGSQVTQL
GUAA_AROAE    ..........................................................................MAHQKILILDFGSQVTQL
GUAA_AZOSB    ..........................................................................MSHQKILILDFGSQVSQL
GUAA_METFK    ...........................................................................MQQKILILDFGSQYTQL
GUAA_THIDA    ...........................................................................MHQKILILDFGSQYTQL
GUAA_NITEC    ...........................................................................MHSAILILDFGSQYARL
GUAA_NITEU    ...........................................................................MHSAILILDFGSQYTRL
GUAA_NITMU    .........................................................................MVMHQKILILDFGSQYTQL
GUAA_NEIMA    ..........................................................................MTQDKILILDFGSQVTQL
GUAA_NEIMF    ..........................................................................MTQDKILILDFGSQVTQL
GUAA_NEIG1    ..........................................................................MTQDKILILDFGSQVTRL
GUAA_NEIG2    ..........................................................................MTQDKILILDFGSQVTRL
GUAA_NEIM0    ..........................................................................MTQDKILILDFGSQVTQL
GUAA_NEIMB    ..........................................................................MTQDKILILDFGSQVTQL
GUAA_GEOMG    ......................................................................MSSDIHSEKILILDFGSQYTQL
GUAA_GEOSL    ......................................................................MSTDIHTEKILILDFGSQYTQL
GUAA_GEODF    ......................................................................MSVDIHSEKILILDFGSQYTQL
GUAA_PELPD    ......................................................................MKTDIHSQKILILDFGSQYTQL
GUAA_PELCD    ......................................................................MSQDIHGEKILILDFGSQYTQL
GUAA_BARHE    .......................................................................MSISHSDTVLIIDFGSQVTQL
GUAA_BARQU    .......................................................................MSISHPDTVLIIDFGSQVTQL
GUAA_BARBK    .......................................................................MDTSHSDTVLIIDFGSQVTQL
GUAA_RHIE6    ......................................................................MTHTAHPDSVLIVDFGSQVTQL
GUAA_RHILW    ......................................................................MTQTAHPDSVLIVDFGSQVTQL
GUAA_RHIEC    ......................................................................MTQTAHPDSVLIVDFGSQVTQL
GUAA_RHIL3    ......................................................................MTQTAHPDSVLIVDFGSQVTQL
GUAA_AGRRK    ......................................................................MTQTAQPDTVLIVDFGSQVTQL
GUAA_RHISN    ......................................................................MTQTAHPDTVLIVDFGSQVTQL
GUAA_RHIME    ......................................................................MTQTAHPDTVLIVDFGSQVTQL
GUAA_SINMW    ......................................................................MTQTAHPDTVLIVDFGSQVTQL
GUAA_AGRVS    ......................................................................MTQTAHPDSVLIIDFGSQVTQL
GUAA_BRUSI    ......................................................................MSTTAYPDTILIIDFGSQVTQL
GUAA_BRUAB    ......................................................................MSTTAYPDTILIIDFGSQVTQL
GUAA_BRUA2    ......................................................................MSTTAYPDTILIIDFGSQVTQL
GUAA_BRUA1    ......................................................................MSTTAYPDTILIIDFGSQVTQL
GUAA_BRUO2    ......................................................................MSTTAYPDTILIIDFGSQVTQL
GUAA_BRUSU    ......................................................................MSTTAYPDTILIIDFGSQVTQL
GUAA_BRUC2    ......................................................................MSTTAYPDTILIIDFGSQVTQL
GUAA_BRUMB    ......................................................................MSTTAYPDTILIIDFGSQVTQL
GUAA_RHILO    ......................................................................MKTANHPDTVLIVDFGSQFTQL
GUAA_AGRT5    ......................................................................MTQIAHPDSILIIDFGSQVTQL
GUAA_BRADU    .......................................MGVQTCAVAGRGVVAYLLAMTAAQNDRSASTPSVASAHDKILIVDFGSQVTQL
GUAA_BRASO    .......................................................MTAPANNTPAAAATDTSVAALHDKILIVDFGSQVTQL
GUAA_OLICO    .......................................................MTAPRQS....TSATPDVAAAHDKILIVDFGSQVTQL
GUAA_RUEST    ......................................................................MTETAH.DRLLIIDFGSQVTQL
GUAA_RUEPO    ......................................................................MSQTSH.DRLLIIDFGSQVTQL
GUAA_CAUCR    ......................................................................MTQKTDHQRVLIVDFGSQVTQL
GUAA_CAUCN    ......................................................................MTQKTDHQRVLIVDFGSQVTQL
GUAA_PHEZH    ......................................................................MTDTADHEHVLIVDFGSQVTQL
GUAA_RHORT    .............................................................MTDRTSVS......ATDRILIIDFGSQVTQL
GUAA_GLUOX    .........................................................MTLTQQDASTVEKLDETLHEDRILILDFGSQVTQL
GUAA_RHOBA    MPATGITLPSPSGPPLPCSAAIFVLDHRSRLPWPFPVSPLLFYTAALAFPAISGKNTMTSSANTTSPAIAETTWLTDQRILVLDFGSQYAQL
GUAA_BACFR    ...........................................................................MQEKIIILDFGSQTTQL
GUAA_BACFN    ...........................................................................MQEKIIILDFGSQTTQL
GUAA1_BACTN   ...........................................................................MQEKIIILDFGSQTTQL
GUAA_CYTH3    ...........................................................................MTEKILILDFGSQYTQL
GUAA_PELLD    ..........................................................MQS..................VLVLDFGSQYTQL
GUAA_PROVI    ..........................................................MQS..................VLVLDFGSQYTQL
GUAA_CHLL2    ..........................................................MQS..................VLVLDFGSQYTQL
GUAA_CHLPD    ..........................................................MQS..................VTVLDFGSQYTQL
GUAA_PELPB    ..........................................................MQS..................VLVLDFGSQYTQL
GUAA_CHLTE    ......................................................MATSLQS..................VIVLDFGSQYTQL
GUAA_CHLCH    ..........................................................MQS..................VLVLDFGSQYTQL
GUAA_CHLPB    ..........................................................MHS..................VLVLDFGSQYTQL
GUAA_CHLT3    ..........................................................MNS..................VIVLDFGSQYTQL
GUAA_SALRD    .........................................................MHDK..................IVVLDFGSQYTQL
GUAA_GEMAT    .........................................................MNSR..................ILILDCGSQFTQL
GUAA_KLULA    ................................................MSPVE.VSNVFDT..................ILVLDFGSQYSHL
GUAA_ASHGO    ................................................MAAVEQVSSVFDT..................ILVLDFGSQYSHL
GUAA_YEAST    ................................................MAAGEQVSNMFDT..................ILVLDFGSQYSHL
GUAA_CANGA    ................................................MSSIEQVNEVFDT..................ILVLDFGSQYSHL
GUAA_DEBHA    ............................................MVNPADVPIEVSKVFDT..................ILVLDFGSQYSHL
GUAA_CANAL    ...........................................MSANIDDVPIEVSKVFDT..................ILVLDFGSQYSHL
GUAA_YARLI    ................................................MPAPVNIPSMFDT..................ILVLDFGSQYSHL
GUAA_PHANO    ............................................MATDGSDPIPPHKTFDT..................ILVLDFGSQYTHL
GUAA_EMENI    ...............................................MADT..IPHNTFDT..................ILVLDFGSQYTHL
GUAA_ASPOR    ..............................................MAETPELEPHNAFDT..................ILTLDFGSQYTHL
GUAA_ASPFU    ..............................................MAEEQ..NPSATFDT..................ILTLDFGSQYTHL
GUAA_USTMA    ..................................................MTEAIHSQYDS..................ILILDFGSQYSHL
GUAA_DEHM1    ......................................................MEIAKEKSGAKPEFIDNEDESLRESIVIFDFGSQYSLL
GUAA_DEHMC    ......................................................MEIAREKSGAKSECIDSGEETLRESIVIFDFGSQYSLL
GUAA_DESPS    ......................................................MDIHKEK..................IIILDFGSQTTQL
GUAA_BRAHW    ......................................................MQNNIDK..................VLILDFGSQFTQL
GUAA_DESVV    .......................................................MDAQTK..................VIIIDYGSQVTQL
GUAA_DESVH    .......................................................MDAQTK..................VIIIDYGSQVTQL
GUAA_DESVM    .......................................................MEAQTK..................VIIIDYGSQVTQL
GUAA_DESAG    .......................................................MEKQNK..................VIIVDYGSQVTQL
GUAA_DESMR    .......................................................MSQPDK..................VLILDFGSQYTQL
GUAA_DESAD    .......................................................MQHDNK..................VIILDFGSQFTQL
GUAA_CALBD    ..........................................................MQH................EIVIVLDFGGQYNQL
GUAA_CALS8    ..........................................................MQH................EIVIVLDFGGQYNQL
GUAA_CARHZ    ...........................................................MA................SKVIVLDFGGQYSQL
GUAA_CLOBL    ..........................................................MNK................ELVLVVDFGGQYNQL
GUAA_CLOBK    ..........................................................MNK................ELVLVVDFGGQYNQL
GUAA_CLOBM    ..........................................................MNK................ELVLVVDFGGQYNQL
GUAA_CLOBJ    ..........................................................MNK................ELVLVVDFGGQYNQL
GUAA_CLOB6    ..........................................................MNK................ELVLVVDFGGQYNQL
GUAA_CLOBH    ..........................................................MNK................ELVLVVDFGGQYNQL
GUAA_CLOB1    ..........................................................MNK................ELVLVVDFGGQYNQL
GUAA_CLONN    ........................................................MAIER................ELVLVVDFGGQYNQL
GUAA_CLOAB    ..........................................................MDK................QLVLVIDFGGQYNQL
GUAA_CLOTE    ..........................................................MKK................ELVFIIDFGGQYSQL
GUAA_CLOPE    ...........................................................MR................DLVLVVDFGGQYNQL
GUAA_CLOB8    ..........................................................MKR................DLVLVVDFGGQYNQL
GUAA_ALKMQ    ..........................................................MKQ................ELVLILDFGGQYNQL
GUAA_CLOTH    ..........................................................MNN................ELILVLDFGGQYNQL
GUAA_CLOCE    ..........................................................MNN................EMILVLDFGGQYNQL
GUAA_CLOBA    ..........................................................MKR................DLVLVIDFGGQYNQL
GUAA_PEPD6    ..........................................................MKH................ELVLVIDFGGQYNQL
GUAA_FINM2    ..........................................................MKH................QLVIVVDFGGQYNQL
GUAA_TREDE    ..........................................................MKKK...PLSAKIVFMKITEKILIVDFGGQYNQL
GUAA_BACCR    ......................................................MIILKKQ...............HDTIIVLDFGSQYNQL
GUAA_BACC4    .........................................................MKKQ...............HDTIIVLDFGSQYNQL
GUAA_BACC2    .........................................................MKKQ...............HDTIIVLDFGSQYNQL
```

```
GUAA_BACWK              .........................................................MKKQ...............HDTIIVLDFGSQYNQL
GUAA_BACCZ              ......................................................MIILKKQ...............HDTIIVLDFGSQYNQL
GUAA_BACC3              ......................................................MIILKKQ...............HDTIIVLDFGSQYNQL
GUAA_BACAH              ......................................................MIILKKQ...............HDTIIVLDFGSQYNQL
GUAA_BACAC              ......................................................MIILKKQ...............HDTIIVLDFGSQYNQL
GUAA_BACAA              ......................................................MIILKKQ...............HDTIIVLDFGSQYNQL
GUAA_BACHK              ......................................................MIILKKQ...............HDTIIVLDFGSQYNQL
GUAA_BACC0              .........................................................MKKQ...............HDTIIVLDFGSQYNQL
GUAA_BACAN              .........................................................MKKQ...............HDTIIVLDFGSQYNQL
GUAA_BACC1              .........................................................MKKQ...............HDTIIVLDFGSQYNQL
GUAA_BACCN              .........................................................MKKQ...............HDTIIVLDFGSQYNQL
GUAA_BACA2              ........................................................MTKLV...............NEMILVLDFGSQYNQL
GUAA_BACSU              ........................................................MTKLV...............NEMILVLDFGSQYNQL
GUAA_BACLD              ........................................................MTKLV...............NEMILVLDFGSQYNQL
GUAA_BACP2              ........................................................MTNLV...............NEMILVLDFGSQYNQL
GUAA_BACSK              ........................................................MQVTN...............HETIVVLDFGGQYNQL
GUAA_BACHD              ........................................................MEQLS...............EEMIVVLDFGGQYNQL
GUAA_GEOKA              ...........................................................MN...............QEMIVVLDFGSQYNQL
GUAA_LISMF              .......................................................MKDFTE...............QEKIIVLDFGSQYNQL
GUAA_LISMO              ...................................................MFKIMKDFTE...............QEKIIVLDFGSQYNQL
GUAA_LISW6              ...................................................MFKIMKDFTE...............QEKIIVLDFGSQYNQL
GUAA_LISIN              ...................................................MFKIMKDFTE...............QEKIIVLDFGSQYNQL
GUAA_EXIS2              .......................................................MEAHLD...............QEMILVLDFGGQYNQL
GUAA_STAES              .......................................................MEMAKE...............QELILVLDFGSQYNQL
GUAA_STAEQ              .......................................................MEMAKE...............QELILVLDFGSQYNQL
GUAA_MACCJ              .......................................................MEMAFE...............QELILVLDFGSQYNQL
GUAA_OCEIH              ..........................................................MEN...............NELVLVLDFGSQYNQL
GUAA_LACRJ              .....................................................MANINLDA...............FDKIIALDFGSQYNQL
GUAA_LACRD              .....................................................MANINLDA...............FDKIIALDFGSQYNQL
GUAA_LACJO              .....................................................MAKTNLND...............FDKIIVLDFGSQYNQL
GUAA_LACGA              .....................................................MAKTNLND...............FDKIIVLDFGSQYNQL
GUAA_LACAC              .....................................................MAK.NLED...............FDKIIVLDFGSQYNQL
GUAA_LACDB              .....................................................MAKIDLSN...............FDKIIVLDFGSQYNQL
GUAA_LACDA              .....................................................MAKIDLSN...............FDKIIVLDFGSQYNQL
GUAA_LACCB              .....................................................MANDQNKD...............YDKIIVLDYGSQYNQL
GUAA_LACC3              .....................................................MANDQNKD...............YDKIIVLDFGSQYNQL
GUAA_LACRH              .....................................................MANDQNKD...............YDKIIVLDYGSQYNQL
GUAA_LACS1              .....................................................MANVDMQT...............FDKIIVLDFGSQYNQL
GUAA_LACLM              ...................................................MSDTT....................LEKIIVLDYGSQYNQL
GUAA_LACLS              ...................................................MSDTT....................LEKIIVLDYGSQYNQL
GUAA_LACLA              ...................................................MSDTT....................LEKIIVLDYGSQYNQL
GUAA_STRZJ              ...................................................MSNISTDLQD...............VEKIIVLDYGSQYNQL
GUAA_STRPJ              ...................................................MSNISTDLQD...............VEKIIVLDYGSQYNQL
GUAA_STRZT              ...................................................MSNISTDLQD...............VEKIIVLDYGSQYNQL
GUAA_STRR6              ...................................................MSNISTDLQD...............VEKIIVLDYGSQYNQL
GUAA_STRPN              ...................................................MSNISTDLQD...............VEKIIVLDYGSQYNQL
GUAA_STRPI              ...................................................MSNISTDLQD...............VEKIIVLDYGSQYNQL
GUAA_STRP7              ...................................................MSNISTDLQD...............VEKIIVLDYGSQYNQL
GUAA_STRP4              ...................................................MSNISTDLQD...............VEKIIVLDYGSQYNQL
GUAA_STRP2              ...................................................MSNISTDLQD...............VEKIIVLDYGSQYNQL
GUAA_STRZP              ...................................................MSNISTDLQD...............VEKIIVLDYGSQYNQL
GUAA_STRPS              ...................................................MSNISTDLQD...............VEKIIVLDYGSQYNQL
GUAA_SYMTH              ........................................................................MSQQRDLIVVVDFGGQYSHL
GUAA_MOOTA              ....................................................................MIGENKQLAEIVLVLDFGGQYNQL
GUAA_HELMI              ........................................................................MAKPHETILVLDFGGQYNQL
GUAA_HALOH              ..........................................................................MKEDKILVLDFGGQYSQL
GUAA_THETN              ........................................................................MGIKRETILILDFGGQYTQL
GUAA_NATTJ              ..........................................................................MSRELVIVLDFGGQYSRL
GUAA_MYCTU              ............................................................MV...QPADIDVPETPARPVLVVDFGAQYAQL
GUAA_MYCTA              ............................................................MV...QPADIDVPETPARPVLVVDFGAQYAQL
GUAA_MYCBT              ............................................................MV...QPADIDVPETPARPVLVVDFGAQYAQL
GUAA_MYCBP              ............................................................MV...QPADIDVPETPARPVLVVDFGAQYAQL
GUAA_MYCBO              ............................................................MV...QPADIDVPETPARPVLVVDFGAQYAQL
GUAA_MYCTO              ............................................................MV...QPADIDVPETPARPVLVVDFGAQYAQL
GUAA_MYCMM              ............................................................MA...EPVDLDVAGPVGRPVLVVDFGAQYAQL
GUAA_MYCPA              ............................................................MA...ESPPTEVPEPPARPVLVVDFGAQYAQL
GUAA_NOSS1              ................................................MNTAVTLLTEQAP.....QPIEEFGQLERQIIIILDFGSQYSEL
GUAA_ANAVT              ................................................MNTAVTLLTEQAP.....QPIEEFGQLERQIIIILDFGSQYSEL
GUAA_NOSP7              ................................................MNTVVTLPTEQAP.....QTLENVGRFDRPLIIILDFGSQYSEL
GUAA_THEEB              ....................................................MTHSTLHQP.....DTATGQG.LQRQLIVILDFGSQYSEL
GUAA_CYAA5              ................................................MTTQTPSLPNKPET...LPTNTLDKSLDRQMIVILDFGSQYSEL
GUAA_CYAP8              ................................................MTTQTPPITNPSEK...LPTEPINKALNRQMLVILDFGSQYSEL
GUAA_CYAP7              ................................................MATQIPSKPSTSET...VPTESLKERINRQMIVILDFGSQYSEL
GUAA_SYNY3              ................................................MTTQIPVPPVVSDQ...ALPDRISDRLKGQIIVILDFGSQYSEL
GUAA_SYNP2              ................................................MLNVTTTPLQKP.....IDATNTGNSLQRQIIVILDFGSQYSEL
GUAA_SYNP6              ................................................MTLQTEM...........EAALEVAALNREMLIILDFGSQYSEL
GUAA_GLOVI              .......................................MTSSPTAAARTEGEAAPTVPTQV...ESGTAQRPGLAREMVAILDFGSQYSEL
GUAA_PROMP              ..................................................................MSKKLLKKERDPSILILDFGSQYSEL
GUAA_PROM5              ..................................................................MGKNLLKKERDPSILILDFGSQYSEL
GUAA_PROMT              ..................................................................MASMISAEKRNPAIVILDFGSQYSEL
GUAA_PROM1              ..................................................................MASMISAEKRNPAIVILDFGSQYSEL
GUAA_THERP              ................................................MSRLLPS...........TTNQHPTSPSFDSVVILDYGSQYAQL
GUAA_DEIGD              .............................................................................MSVVILDFGSQFTRL
GUAA_DEIDV              .............................................................................MSVVILDFGSQFTRL
GUAA_DEIRA              .............................................................................MSIVILDFGSQFTRL
GUAA_FUSNN              .........................................................................MKKG.GIVILDFGSQYNQL
GUAA_MESFL              .........................................................................MKNT.QILILDFGSQYTQL
GUAA_SPIKU              .........................................................................MKTTNKIIILDFGSQYTQL
GUAA_BORGA              .........................................................MKYALIRKLLFVLRTYMMNVRAILVLDFGSQYSQL
GUAA_SULDN              ..........................................................................MTNVSIIVLDFGSQYTQL
GUAA_WOLSU              .....................................................................MSIQLHDKVSLLILDFGSQYTQL
GUAA_SULNB              ..........................................................................MKQVPILVLDFGSQYTQL
GUAA_ARCB4              ..........................................................................MKHVPIVVLDFGSQYTQI
GUAA_NITSB              ..........................................................................MQEVPIVILDFGSQYTQL
GUAA_CAMHC              ..........................................................................MKNSDIIVLDFGSQYTQL
GUAA_THESQ              ..............................................................................MVLVVDYGSQYSRL
GUAA_THEMA              ..............................................................................MVLVVDYGSQYSRL
GUAA_THEP1              ..............................................................................MVLVVDYGSQYSRL
GUAA_PETMO              ............................................................................MEKILVIDYGSQYTQL
GUAA_AQUAE              ..........................................................................MQRRPILVVNFGSQYVQL
GUAA_THEAB              ............................................................................MNTVVVLDYGSQYTQL
GUAA_THEM4              ............................................................................MKTVVVLDYGSQYTQL
GUAA_PELUB              ......................................................................MSLSNNLSKILIIDFGSQFTQL
GUAA_MYCPE              ............................................................................MDKILILDFGGQYTQL
tr|Q7RIY7|Q7RIY7_PLAYO  .......................................................................MEGDNYDMILVLNFGSQYFHL
tr|Q4Z1X2|Q4Z1X2_PLABA  .......................................................................MEGDNYDIILVLNFGSQYFHL
tr|Q4Y2M7|Q4Y2M7_PLACH  ............................................................................................
tr|B3L3R6|B3L3R6_PLAKH  .......................................................................MEGGDYDKILVLNFGSQYFHL
tr|A5K7F1|A5K7F1_PLAVS  .......................................................................MEGGDYDKILVLNFGSQYFHL
tr|Q8IJR9|Q8IJR9_PLAF7  .......................................................................MEGEEYDKILVLNFGSQYFHL
```

	30 . 40 . 50 . 60 . 70 . 80 . 90 . 100 .
GUAA_ECO55	VARRVRELGVYCELWAWD.VTEAQIRDF....NPSGIILSGGPESTTEEN.SPRAPQYVFEAG......VPVFGVCYGMQTMAMQL.GGHVE
GUAA_ECO45	VARRVRELGVYCELWAWD.VTEAQIRDF....NPSGIILSGGPESTTEEN.SPRAPQYVFEAG......VPVFGVCYGMQTMAMQL.GGHVE
GUAA_ECO57	VARRVRELGVYCELWAWD.VTEAQIRDF....NPSGIILSGGPESTTEEN.SPRAPQYVFEAG......VPVFGVCYGMQTMAMQL.GGHVE
GUAA_ECO7I	VARRVRELGVYCELWAWD.VTEAQIRDF....NPSGIILSGGPESTTEEN.SPRAPQYVFEAG......VPVFGVCYGMQTMAMQL.GGHVE
GUAA_ECO5E	VARRVRELGVYCELWAWD.VTEAQIRDF....NPSGIILSGGPESTTEEN.SPRAPQYVFEAG......VPVFGVCYGMQTMAMQL.GGHVE
GUAA_ECO81	VARRVRELGVYCELWAWD.VTEAQIRDF....NPSGIILSGGPESTTEEN.SPRAPQYVFEAG......VPVFGVCYGMQTMAMQL.GGHVE
GUAA_ECO8A	VARRVRELGVYCELWAWD.VTEAQIRDF....NPSGIILSGGPESTTEEN.SPRAPQYVFEAG......VPVFGVCYGMQTMAMQL.GGHVE
GUAA_ECOHS	VARRVRELGVYCELWAWD.VTEAQIRDF....NPSGIILSGGPESTTEEN.SPRAPQYVFEAG......VPVFGVCYGMQTMAMQL.GGHVE
GUAA_ECOK1	VARRVRELGVYCELWAWD.VTEAQIRDF....NPSGIILSGGPESTTEEN.SPRAPQYVFEAG......VPVFGVCYGMQTMAMQL.GGHVE
GUAA_ECOL5	VARRVRELGVYCELWAWD.VTEAQIRDF....NPSGIILSGGPESTTEEN.SPRAPQYVFEAG......VPVFGVCYGMQTMAMQL.GGHVE
GUAA_ECOL6	VARRVRELGVYCELWAWD.VTEAQIRDF....NPSGIILSGGPESTTEEN.SPRAPQYVFEAG......VPVFGVCYGMQTMAMQL.GGHVE
GUAA_ECOLC	VARRVRELGVYCELWAWD.VTEAQIRDF....NPSGIILSGGPESTTEEN.SPRAPQYVFEAG......VPVFGVCYGMQTMAMQL.GGHVE
GUAA_ECOSE	VARRVRELGVYCELWAWD.VTEAQIRDF....NPSGIILSGGPESTTEEN.SPRAPQYVFEAG......VPVFGVCYGMQTMAMQL.GGHVE
GUAA_ECOSM	VARRVRELGVYCELWAWD.VTEAQIRDF....NPSGIILSGGPESTTEEN.SPRAPQYVFEAG......VPVFGVCYGMQTMAMQL.GGHVE
GUAA_ECOUT	VARRVRELGVYCELWAWD.VTEAQIRDF....NPSGIILSGGPESTTEEN.SPRAPQYVFEAG......VPVFGVCYGMQTMAMQL.GGHVE
GUAA_ECO24	VARRVRELGVYCELWAWD.VTEAQIRDF....NPSGIILSGGPESTTEEN.SPRAPQYVFEAG......VPVFGVCYGMQTMAMQL.GGHVE
GUAA_SHISS	VARRVRELGVYCELWAWD.VTEAQIRDF....NPSGIILSGGPESTTEEN.SPRAPQYVFEAG......VPVFGVCYGMQTMAMQL.GGHVE
GUAA_SHIF8	VARRVRELGVYCELWAWD.VTEAQIRDF....NPSGIILSGGPESTTEEN.SPRAPQYVFEAG......VPIFGVCYGMQTMAMQL.GGHVE
GUAA_SHIFL	VARRVRELGVYCELWAWD.VTEAQIRDF....NPSGIILSGGPESTTEEN.SPRAPQYVFEAG......VPIFGVCYGMQTMAMQL.GGHVE
GUAA_SHIDS	VARRVRELGVYCELWAWD.VTEAQIRDF....NPSGIILSGGPESTTEEN.SPRAPQYVFEAG......VPVFGVCYGMQTMAMQL.GGHVE
GUAA_ECOLI	VARRVRELGVYCELWAWD.VTEAQIRDF....NPSGIILSGGPESTTEEN.SPRAPQYVFEAG......VPVFGVCYGMQTMAMQL.GGHVE
GUAA_ECODH	VARRVRELGVYCELWAWD.VTEAQIRDF....NPSGIILSGGPESTTEEN.SPRAPQYVFEAG......VPVFGVCYGMQTMAMQL.GGHVE
GUAA_ECOBW	VARRVRELGVYCELWAWD.VTEAQIRDF....NPSGIILSGGPESTTEEN.SPRAPQYVFEAG......VPVFGVCYGMQTMAMQL.GGHVE
GUAA_ESCF3	VARRVRELGVYCELWAWD.VTEAQIRDF....NPSGIILSGGPESTTEEN.SPRAPQYVFEAG......VPVFGICYGMQTMAMQL.GGHVE
GUAA_ECO27	VARRVRELGVYCELWAWD.VTEAQIRDF....NPSGIILSGGPESTTEEN.SPRAPQYVFEAG......VPVFGICYGMQTMAMQL.GGHVE
GUAA_SHIB3	VARRVRELGVYCELWAWD.VTEAQIRDF....NPSGIILSGGPESTTEEN.SPRAPQYVFEAG......VPVFGVCYGMQTMAMQL.GGHVE
GUAA_SHIBS	VARRVRELGVYCELWAWD.VTEAQIRDF....NPSGIILSGGPESTTEEN.SPRAPQYVFEAG......VPVFGVCYGMQTMAMQL.GGHVE
GUAA_ECOLU	VARRVRELGVYCELWAWD.VTEAQIRDF....NPSGIILSGGPESTTEEN.SPRAPQYVFEAG......VPVFGVCYGMQTMAMQL.GGHVE
GUAA_SALPK	VARRVRELGVYCELWAWD.VTEAQIRDF....NPSGIILSGGPESTTEEN.SPRAPQYVFEAG......VPVFGVCYGMQTMAMQL.GGHVE
GUAA_SALPC	VARRVRELGVYCELWAWD.VTEAQIRDF....NPSGIILSGGPESTTEEN.SPRAPQYVFEAG......VPVFGVCYGMQTMAMQL.GGHVE
GUAA_SALPB	VARRVRELGVYCELWAWD.VTEAQIRDF....NPSGIILSGGPESTTEEN.SPRAPQYVFEAG......VPVFGVCYGMQTMAMQL.GGHVE
GUAA_SALPA	VARRVRELGVYCELWAWD.VTEAQIRDF....NPSGIILSGGPESTTEEN.SPRAPQYVFEAG......VPVFGVCYGMQTMAMQL.GGHVE
GUAA_SALCH	VARRVRELGVYCELWAWD.VTEAQIRDF....NPSGIILSGGPESTTEEN.SPRAPQYVFEAG......VPVFGVCYGMQTMAMQL.GGHVE
GUAA_SALTY	VARRVRELGVYCELWAWD.VTEAQIRDF....NPSGIILSGGPESTTEEN.SPRALQYVFEAG......VPVFGVCYGMQTMAMQL.GGHVE
GUAA_SALSV	VARRVRELGVYCELWAWD.VTEAQIRDF....NPSGIILSGGPESTTEEN.SPRAPQYVFEAG......VPVFGVCYGMQTMAMQL.GGHVE
GUAA_SALNS	VARRVRELGVYCELWAWD.VTEAQIRDF....NPSGIILSGGPESTTEEN.SPRAPQYVFEAG......VPVFGVCYGMQTMAMQL.GGHVE
GUAA_SALTI	VARRVRELGVYCELWAWD.VTEAQIRDF....NPSGIILSGGPESTTEEN.SPRAPQYVFEAG......VPVFGVCYGMQTMAMQL.GGHVE
GUAA_SALHS	VARRVRELGVYCELWAWD.VTEAQIRDF....NPSGIILSGGPESTTEEN.SPRAPQYVFEAG......VPVFGVCYGMQTMAMQL.GGHVE
GUAA_SALG2	VARRVRELGVYCELWAWD.VTEAQIRDF....NPSGIILSGGPESTTEEN.SPRAPQYVFEAG......VPVFGVCYGMQTMAMQL.GGHVE
GUAA_SALEP	VARRVRELGVYCELWAWD.VTEAQIRDF....NPSGIILSGGPESTTEEN.SPRAPQYVFEAG......VPVFGVCYGMQTMAMQL.GGHVE
GUAA_SALDC	VARRVRELGVYCELWAWD.VTEAQIRDF....NPSGIILSGGPESTTEEN.SPRAPQYVFEAG......VPVFGVCYGMQTMAMQL.GGHVE
GUAA_SALA4	VARRVRELGVYCELWAWD.VTEAQIRDF....NPSGIILSGGPESTTEEN.SPRAPQYVFEAG......VPVFGVCYGMQTMAMQL.GGHVE
GUAA_SALAR	VARRVRELGVYCELWAWD.VTEAQIREF....NPSGIILSGGPESTTEEN.SPRAPQYVFEAG......VPVFGVCYGMQTMAMQL.GGHVE
GUAA_KLEP7	VARRVRELGVYCELWAWD.VTEAQIREF....NPSGIILSGGPESTTEEN.SPRAPQYVFEAG......VPVFGVCYGMQTMAMQL.GGHVE
GUAA_KLEP3	VARRVRELGVYCELWAWD.VTEAQIREF....NPSGIILSGGPESTTEEN.SPRAPQYVFEAG......VPVFGVCYGMQTMAMQL.GGHVE
GUAA_ENT38	VARRVRELGVYCELWAWD.VTEAQIREF....NPSGIILSGGPESTTEDN.SPRAPQYVFEAG......VPVFGVCYGMQTMAMQL.GGHVE
GUAA_CITK8	VARRVRELGVYCELWAWD.VTEAQIREF....NPSGIILSGGPESTTEEN.SPRAPEYVFEAG......VPVFGVCYGMQTMAMQL.GGHVE
GUAA_CROS8	VARRVRELGVYCELWAWD.VTEEQIRGF....NPNGIILSGGPESTTEEN.SPRAPEYVFNAG......VPVLGVCYGMQTMAMQL.GGHVE
GUAA_SODGM	VARRVRELGVYCELWAWD.VTEAQIREF....NPSGIILSGGPESTTEQD.SPRAPDYVFQAG......VPVLGVCYGMQTMAMQL.GGKVQ
GUAA_PECCP	VARRVRELGVYCELWAWD.VTEAQIRGF....NPNGIILSGGPESTTEFG.SPRAPEYVFNAG......VPVLGVCYGMQTMAMQL.GGHVE
GUAA_PECAS	VARRVRELGVYCELWAWD.VTEAQIRGF....NPNGIILSGGPESTTEFG.SPRAPEYVFNAG......VPVLGVCYGMQTMAMQL.GGHVE
GUAA_YERPY	LARRVREIGVYCELWAWD.VTEAQIREF....NPSGIILSGSPESTIENG.SPRAPDYVFTAG......VPVLGVCYGMQTMAIQL.GGKVE
GUAA_YERPS	LARRVREIGVYCELWAWD.VTEAQIREF....NPSGIILSGSPESTIENG.SPRAPDYVFTAG......VPVLGVCYGMQTMAIQL.GGKVE
GUAA_YERPB	LARRVREIGVYCELWAWD.VTEAQIREF....NPSGIILSGSPESTIENG.SPRAPDYVFTAG......VPVLGVCYGMQTMAIQL.GGKVE
GUAA_YERP3	LARRVREIGVYCELWAWD.VTEAQIREF....NPSGIILSGSPESTIENG.SPRAPDYVFTAG......VPVLGVCYGMQTMAIQL.GGKVE
GUAA_YERPP	LARRVREIGVYCELWAWD.VTEAQIREF....NPSGIILSGSPESTIENG.SPRAPDYVFTAG......VPVLGVCYGMQTMAIQL.GGKVE
GUAA_YERPN	LARRVREIGVYCELWAWD.VTEAQIREF....NPSGIILSGSPESTIENG.SPRAPDYVFTAG......VPVLGVCYGMQTMAIQL.GGKVE
GUAA_YERPG	LARRVREIGVYCELWAWD.VTEAQIREF....NPSGIILSGSPESTIENG.SPRAPDYVFTAG......VPVLGVCYGMQTMAIQL.GGKVE
GUAA_YERPE	LARRVREIGVYCELWAWD.VTEAQIREF....NPSGIILSGSPESTIENG.SPRAPDYVFTAG......VPVLGVCYGMQTMAIQL.GGKVE
GUAA_YERPA	LARRVREIGVYCELWAWD.VTEAQIREF....NPSGIILSGSPESTIENG.SPRAPDYVFTAG......VPVLGVCYGMQTMAIQL.GGKVE
GUAA_YERE8	VARRVREIGVYCELWAWD.VTEDQIREF....NPSGIILSGGPESTTEHD.SPRAPDYVFTAG......VPVLGVCYGMQTMAMQL.GGKVE
GUAA_SERP5	VARRVREIGVYCELWAWD.VSEEQIREF....NPSGIILSGGPESTTEAN.SPRAPEYVFTAG......VPVLGVCYGMQTMAMQL.GGQVE
GUAA_PHOLL	IARRIREIGVYCELWAWD.VTEEQIREF....NPSGIILSGGPESTTAQG.SPRAPEYVFNAG......VPVLGVCYGMQTMSVQF.GGKVE
GUAA_PROMH	IARRIREIGVYCELWAWD.VTEEQIREF....NPNGIILSGGPESTTEDN.SPRAPEYVFNAG......VPVLGICYGMQTMSMQL.GGDVE
GUAA_EDWI9	LARRVREIGVYCELWAWD.VSEAQIRDF....NPSGIILSGGPESTTEAG.SPRAPEYVFQAG......VPVLGVCYGMQTMAMQL.GGRVE
GUAA_AERHH	IARRVREIGVYCELWAWD.VTEEQMREF....NPSGIILSGGPESVTEAG.SPRAPEYVFNAG......VPVFGICYGMQTMAEQL.GGKVQ
GUAA_AERS4	IARRVREIGVYCELWAWD.VTEEQIREF....NPSGIILSGGPESVTEAG.SPRAPEYVFNAG......VPVFGICYGMQTMAEQL.GGKVQ
GUAA_TOLAT	IARRVREIGVYCELWAWD.VTEEQIREF....NPDGIILSGGPESVTESD.SPRAPEYVFNAG......VPVLGVCYGMQTMAEQL.GGKVE
GUAA_VIBF1	VARRIREIGVYCELWSWD.VEESDIRDF....NPDGIILSGGPESVTEEN.SPRAPQYVFDSG......VPVFGVCYGMQTMAEQL.GGKVA
GUAA_VIBFM	VARRIREIGVYCELWSWD.VEESDIRDF....NPDGIILSGGPESVTEEN.SPRAPQYVFDSG......VPVFGVCYGMQTMAEQL.GGKVA
GUAA_ALISL	VARRIREIGVYCELWSWD.VEESDIRDF....NPDGIILSGGPESVTEAN.SPRAPQYVFDSG......VPVFGVCYGMQTMAEQL.GGKVA
GUAA_VIBSL	VARRIREIGVYCELWSWD.VEEADIREF....NPDGIILSGGPESVTEEN.SPRAPQYVFDSG......VPVFGICYGMQTMAEQL.GGKVA
GUAA_VIBCM	VARRVREIGVYCELWSWD.VEEADIREF....NPDGIILSGGPESVTEAN.SPRAPQYVFDSG......VPVFGVCYGMQTMAEQL.GGRVA
GUAA_VIBCH	VARRVREIGVYCELWSWD.VEEADIREF....NPDGIILSGGPESVTEAN.SPRAPQYVFDSG......VPVFGVCYGMQTMAEQL.GGRVA
GUAA_VIBC3	VARRVREIGVYCELWSWD.VEEADIREF....NPDGIILSGGPESVTEAN.SPRAPQYVFDSG......VPVFGVCYGMQTMAEQL.GGRVA
GUAA_VIBVU	VARRVREIGVYCELWSWD.VEEADIREF....NPDGIILSGGPESVTEDN.SPRAPQYVFDSG......VPVLGVCYGMQTMAEQL.GGKVS
GUAA_VIBVY	VARRVREIGVYCELWSWD.VEEADIREF....NPDGIILSGGPESVTEDN.SPRAPQYVFDSG......VPVLGVCYGMQTMAEQL.GGKVS
GUAA_VIBPA	VARRVREIGVYCELWSWD.VEEADIREF....NPDGIILSGGPESVTEEN.SPRAPQYVFDSG......VPVLGVCYGMQTMAEQL.GGKVA
GUAA_VIBCB	VARRVREIGVYCELWSWD.VEEADIREF....NPDGIILSGGPESVTEDN.SPRAPQYVFDSG......VPVLGVCYGMQTMAEQL.GGKVA
GUAA_PHOPR	IARRIREIGVYCELWSWD.VDEADIRDF....NPNGIILSGGPESVTGDE.SPRAPQYVFEAG......VPVFGVCYGMQTMAEQL.GGKVA
GUAA_COLP3	IARRVREIGVYCELWSWD.VTEEQIKGF....NPTGIILAGGPESVTEAN.SPRAPEYVYTAG......VPVLGICYGMQTMAEQL.GGGVE
GUAA_PSYIN	IARRIREIGVYCELWSWD.VDEQDIKDF...APNGIILAGGPESVTAEG.SPRAPEYVFTAG......VPVLGICYGMQTMSEQL.GGSVI
GUAA_PSEHT	IARRVREIGVYCELWAWD.VTEEQIREF....NPQGIILSGGPESTTLEN.SPRAPEYVFNAG......VPVLGICYGMQTMAAQL.GGSVH
GUAA_PSEA6	IARRVREIGVYCELWAWD.VTEEQIREF....NPNGIILSGGPESVHAKD.SPRAPQYVFDAG......VPVFGICYGMQTMAEQL.GGAVL
GUAA_ALTMD	IARRVREIGVYCELWAWD.VTEEQIREF....NPQGIILSGGPESVTEEG.SPRAPDYVFEAG......VPVLGVCYGMQTMAHQL.GGGVL
GUAA_SHESW	IARRIREIGVYCELWAWD.VTEAQIREF...APNGIILAGGPESVTAEN.SPRAPEYVFNAG......IPVLGICYGMQTMSEQL.GGKVI
GUAA_SHEPC	IARRIREIGVYCELWAWD.VTEAQIREF...APNGIILAGGPESVTAEN.SPRAPEYVFNAG......IPVLGICYGMQTMSEQL.GGKVI
GUAA_SHEB9	IARRIREIGVYCELWAWD.VTEAQIREF...APNGIILAGGPESVTAEN.SPRAPEYVFTAG......VPVLGICYGMQTMSEQL.GGKVI
GUAA_SHEB8	IARRIREIGVYCELWAWD.VTEAQIREF...APNGIILAGGPESVTAEN.SPRAPEYVFTAG......VPVLGICYGMQTMSEQL.GGKVI
GUAA_SHEB5	IARRIREIGVYCELWAWD.VTEAQIREF...APNGIILAGGPESVTAEN.SPRAPEYVFTAG......VPVLGICYGMQTMSEQL.GGKVI
GUAA_SHEB2	IARRIREIGVYCELWAWD.VTEAQIREF...APNGIILAGGPESVTAEN.SPRAPEYVFTAG......VPVLGICYGMQTMSEQL.GGKVI
GUAA_SHESM	IARRIREIGVYCELWAWD.VTEAQIREF...APNGIILAGGPESVTADN.SPRAPEYVFNAG......VPVLGICYGMQTMSEQL.GGKVI
GUAA_SHESA	IARRIREIGVYCELWAWD.VTEAQIREF...APNGIILAGGPESVTADN.SPRAPEYVFNAG......VPVLGICYGMQTMSEQL.GGKVI
GUAA_SHESR	IARRIREIGVYCELWAWD.VTEAQIREF...APNGIILAGGPESVTADN.SPRAPEYVFNAG......VPVLGICYGMQTMSEQL.GGKVI
GUAA_SHEON	IARRIREIGVYCELWAWD.VTEAQIREF...APNGIILAGGPESVTADN.SPRAPEYVFNAG......VPVLGICYGMQTMSEQL.GGKVI
GUAA_SHEDO	IARRIREIGVYCELWAWD.VSEAQIREF....NPQGIILAGGPESVTAEN.SPRAPEYVFNAG......VPVLGICYGMQTMSEQL.GGKVI
GUAA_SHEPW	IARRIREIGVYCELWAWD.VSEEQIRGF...APNGIILAGGPESVTADN.SPRAPEYVFNAG......VPVLGICYGMQTMSEQL.GGKVI
GUAA_SHESH	IARRIREIGVYCELWAWD.VSEAQIKGF...APNGIILAGGPESVTADE.SPRAPEYVFNAG......VPVLGICYGMQTMSEQL.GGKVI
GUAA_SHEWM	IARRIREIGVYCELWAWD.VSEEQIKEF...APNGIILAGGPESVTAKD.SPRAPEYVFTAG......VPVLGICYGMQTMSEQL.GGKVI
GUAA_SHEHH	IARRIREIGVYCELWAWD.VSEEQIREF...APNGIILAGGPESVTAEN.SPRAPEYVFNAG......VPVLGICYGMQTMSEQL.GGKVI
GUAA_SHEPA	IARRIREIGVYCELWAWD.VSEEQIREF...APNGIILAGGPESVTAEN.SPRAPEYVFNAG......VPVLGICYGMQTMSEQL.GGKVI
GUAA_SHEFN	IARRIREIGVYCELWAWD.VSEAQIREF...APNGIILAGGPESVTADN.SPRAPEYVFNAG......VPVLGICYGMQTMSEQL.GGKVI
GUAA_SHELP	IARRIREIGVYCELWAWD.VSEEQIKAF...APNGIILAGGPESVTADN.SPRAPEYVFNAG......VPVLGICYGMQTMSEQL.GGKVI
GUAA_SHEAM	IARRIREIGVYCELWAWD.VTEAQIREF...APNGIVLAGGPESVTEDN.SPRAPEYVFDAG......VPVLGICYGMQTMSEQL.GGKVI
GUAA_IDILO	IARRIREIGVYCELWAWD.VDEADIREF...APKGIILSGGPESVAEEG.SPRAPEYVFNAG......VPVFGICYGMQTMAHQL.GGSVQ
GUAA_ACTSZ	IARRVREIGVYCELWAWD.VTEQQIREF....NPTGIILSGGPESTTEND.SPRAPEYVFDAG......VPVLGVCYGMQTMAMQL.GGLTE
GUAA_MANSM	IARRVREIGVYCELWAWD.VTEQQIREF....NPTGIILSGGPESTTEDN.SPRAPEYVFNAG......VPVLGICYGMQTMAMQL.GGLTE
GUAA_ACTPJ	IARRVREIGVYCELWAWD.VTEEQIREF....NPTGIILSGGPESTTEEN.SPRAPEYVFNAG......VPVLGICYGMQTMAMQL.GGLTE
GUAA_ACTP7	IARRVREIGVYCELWAWD.VTEEQIREF....NPTGIILSGGPESTTEEN.SPRAQEYVFNAG......VPVLGICYGMQTMAMQL.GGLTE
GUAA_ACTP2	IARRVREIGVYCELWAWD.VTEEQIREF....NPTGIILSGGPESTTEEN.SPRAPEYVFNAG......VPVLGICYGMQTMAMQL.GGLTE
GUAA_HAEPS	IARRVREIGVYCELWAWD.VTEEQIREF....NPTGIILSGGPESTTEEN.SPRAPEYVFNAG......VPVLGICYGMQTMAMQL.GGLTE
GUAA_PASMU	IARRVREIGVYCELWAWD.VSEADIREF....NPTGIILSGGPESTTEEN.SPRAPEYVFNAG......VPVLGICYGMQTMAMQL.GGLTE
GUAA_HAEDU	IARRIREIGVYCELWAWD.VTEKQIREF....NPTGIILSGGPESTTETN.SPHAPEYVFKAG......VPVLGICYGMQTMAMQL.GGLTE
GUAA_HISS1	IARRVREIGVYCELWAWD.VTEEQIREF....NPTGIILSGGPESTTEAN.SPRAPEYVFNAG......VPVLGICYGMQTMAMQL.GGLTE
GUAA_HISS2	IARRVREIGVYCELWAWD.VTEEQIREF....NPTGIILSGGPESTTEAN.SPRATEYVFNAG......VPVLGICYGMQTMAMQL.GGLTE
GUAA_HAEI8	IARRVREIGVYCELWAWD.VTEQQIREF...APTGIILSGSPESTTEEN.SPRAPEYVFNAS......VPVLGICYGMQTMAMQL.GGLTE
GUAA_HAEIG	IARRVREIGVYCELWAWD.VTEQQIREF...APTGIILSGGPESTTEEN.SPRAPEYVFNAG......VPVLGVCYGMQTMAMQL.GGLTE
GUAA_HAEIN	IARRVREIGVYCELWAWD.VTEQQIREF...APTGIILSGSPESTTEEN.SPRAPEYVFNAG......VPVLGICYGMQTMAMQL.GGLTE
GUAA_HAEIE	IARRVREIGVYCELWAWD.VTEEQIREF...APTGIILSGSPESTTEEN.SPRAPEYVFNAG......VPVLGVCYGMQTMAMQL.GGLTE
GUAA_HAMD5	LARRIREMGVYSELWPWD.VAAERIQAF...HPQGIILSGGPESATEND.TPRAPDIIFTLG......VPVLGICYGMQTMAIQL.GGTVS
GUAA_XANC5	IARRIREIGVYCEIWAWD.HDPSEIAGF...GAKGIILSGGPESTTSPG.APVAPQEVFDSG......LPVFGICYGMQTLAAQL.GGATE
GUAA_XANAC	IARRIREIGVYCEIWAWD.HDPSEIAGF...GAKGIILSGGPESTTLPG.APVAPQEVFDSG......LPVFGICYGMQTLAAQL.GGATE
GUAA_XANCP	IARRIREIGVYCEIWAWD.HDPSEIAGF...GAKGIILSGGPESTTLPG.APVAPQEVFDSG......LPVFGICYGMQTLAAQL.GGATE
GUAA_XANCB	IARRIREIGVYCEIWAWD.HDPSEIAGF...GAKGIILSGGPESTTLPG.APVAPQEVFDSG......LPVFGICYGMQTLAAQL.GGATE
GUAA_XANC8	IARRIREIGVYCEIWAWD.HDPSEIAGF...GAKGIILSGGPESTTLPG.APVAPQEVFDSG......LPVFGICYGMQTLAAQL.GGATE
GUAA_XANOR	IARRIREIGVYCEIWAWD.HDPSEIAGF...GAKGIILSGGPESTTLPG.APVAPQEVFDSG......LPVFGICYGMQTLAAQL.GGATE
GUAA_XANOM	IARRIREIGVYCEIWAWD.HDPSEIAGF...GAKGIILSGGPESTTLPG.APVAPQEVFDSG......LPVFGICYGMQTLAAQL.GGATE
GUAA_XANOP	IARRIREIGVYCEIWAWD.HDPSEIAGF...GAKGIILSGGPESTTLPG.APVAPQEVFDSG......LPVFGICYGMQTLAAQL.GGATE
GUAA_STRMK	IARRIRELGVYCEIWAWD.HNPAEIAGF...GAKGIILSGGPESTTLPG.APAAPQEVFDSG......LPIFGICYGMQTLAAQL.GGATE
GUAA_STRM5	IARRIRELGVYCEIWAWD.HNPAEIAGF...GAKGIILSGGPESTTLPG.APAAPQEVFDSG......LPIFGICYGMQTLAAQL.GGATE
GUAA_XYLFT	IARRIREIGVYCEVWPWD.HSPEEILSF...GAKGIILSGGPESTTSPG.APAAPQHVFDSD......LPIFGICYGMQTMAVHL.GGATE
GUAA_XYLF2	IARRIREIGVYCEVWPWD.HSPEEILSF...GAKGIILSGGPESTTSPG.APAAPQHVFDSD......LPIFGICYGMQTMAVHL.GGATE
GUAA_XYLFM	IARRIREIGVYCEVWPWD.HSPEEILSF...GAKGIILSGGPESTTSPG.APAAPQHVFDSD......LPIFGICYGMQTMAVHL.GGATE
GUAA_XYLFA	IARRIREIGVYCEVWPWD.HSPEEILSF...GAKGIILSGGPESTTSPG.APAAPQHVFDSD......LPILGICYGMQTMAVHL.GGATE
GUAA_CELJU	IARRVREIGVFSEIRAWD.MSDDEIRAY...KPSGIILAGGPESTTEEE.APRAPEAVFALG......VPVLGICYGMQTMAMQM.GGWVE
GUAA_TERTT	IARRIREVGVFSEIRAFD.MTDEEIREY....NPKAIILSGGPESVTAGA.SPRAPEAVFELG......VPVLGICYGMQTMAEQL.GGRVQ
GUAA_HAHCH	IARRVREIGVYCEIYPFD.MSEADIREF....NPKGVILAGGPESVTELG.SPRAPECLFNMD......LPLLGICYGMQTMAEQM.GGKVA
GUAA_MARAV	IARRVREIGVYCEIKAFD.ITDDELNEF....NPKGIILAGGPESVTQLG.GPRAPEGLFDRG......IPVLGICYGMQTMAEQL.GGRVA
GUAA_PSESM	IARRVREIGVYCELHPFD.MDDEAIREF...APKGVILAGGPESVHEAN.SPRCPQAVFDLG......VPVFGICYGMQTMAEQL.GGKVA
GUAA_PSE14	IARRVREIGVYCELHPFD.MDDEAIREF...APKGVILAGGPESVHEAN.SPRCPQAVFDLG......VPVFGICYGMQTMAEQL.GGKVA
GUAA_PSEU2	IARRVREIGVYCELHPFD.MDDEAIREF...APKGVVLAGGPESVHEAD.SPRCPQAVFDLG......VPVFGICYGMQTMAEQL.GGKVA
GUAA_PSEFS	IARRVREIGVYCELHPFD.MDDEAIREF...APKGVILAGGPESVHEAN.SPRCPQAVFDLG......VPVFGICYGMQTMAEQL.GGKVE
GUAA_PSEF5	IARRVREIGVYCELHPFD.MDDEAIREF...APKGVILAGGPESVHEAN.SPRCPQAVFDLG......VPVFGICYGMQTMAEQL.GGKVE
GUAA_PSEPF	IARRVREIGVYCELHPFD.MDEDAIREF...APKGVILAGGPESVHEAN.SPRCPQAVFDLG......VPVFGICYGMQTMAEQL.GGKVE

```
GUAA_PSEPK    IARRVREIGVYCELHPFD.MDDEAIREF....NPRGIILAGGPESVHEAN.SPRAPQAVFDLN......VPVLGICYGMQTMAEQM.GGKVE
GUAA_PSEP1    IARRVREIGVYCELHPFD.MDDEAIREF....NPRGIILAGGPESVHEAN.SPRAPQAVFDLN......VPVLGICYGMQTMAEQM.GGKVE
GUAA_PSEPG    IARRVREIGVYCELHPFD.MDDEAIREF....NPRGIILAGGPESVHEAN.SPRAPQAVFDLN......VPVLGICYGMQTMAEQM.GGKVE
GUAA_PSEE4    IARRVREIGVYCELHPFD.MDDEAIREF....NPRGIILAGGPESVHEAN.SPRAPQAVFDLK......VPVLGICYGMQTMAEQM.GGKVE
GUAA_PSEPW    IARRVREIGVYCELHPFD.MDDEAIREF....NPRGIILAGGPESVHEAN.SPRAPQAVFDLN......VPLLGICYGMQTMAEQL.GGKVE
GUAA_PSEMY    IARRVREIGVYCELHPWD.MSDEDIRAF....APRGIILAGGPESVHEAN.SPRAPQAVFDLN......VPVLGICYGMQTMAEQL.GGKVE
GUAA_PSEAE    IARRVREIGVYCEIHPFD.MSNEAIIAF....APRGIILAGGPESVHEAD.SPRAPQAVFDLK......VPLFGICYGMQTMAEQM.GGKVQ
GUAA_PSEA8    IARRVREIGVYCEIHPFD.MSNEAIIAF....APRGIILAGGPESVHEAD.SPRAPQAVFDLK......VPLFGICYGMQTMAEQM.GGKVQ
GUAA_PSEAB    IARRVREIGVYCEIHPFD.MSNEAIIAF....APRGIILAGGPESVHEAD.SPRAPQAVFDLK......VPLFGICYGMQTMAEQM.GGKVQ
GUAA_MARMS    IARRVREIGVYCEVRAFD.MEDQDVIDF....DPKGIILAGGPESVPEPG.SPRAPEAVFTLG......VPVFGICYGMQTMAEQL.GGKVQ
GUAA_ALCBS    IARRVREIGVYCEIRAFD.MTAEELAEF....APKGIILSGGPESVTAAQ.TPRAPQAVFEAG......IPVLGICYGMQTMAEQL.GGKVI
GUAA_THICR    IARRIREIGVYCEVEPWD.IDVEDIVKF....GARGIILSGGPETVTGDN.APVAPVEVFELG......VPVLGICYGMQTMAEQL.GGKVI
GUAA_ALKEH    IARRVREAGVYCEIYAWD.APETAIRDF....GAAGFILSGGPESMTVDN.GPRVPEAVFQAG......VPVLGICYGMQAMAGQL.GGRVE
GUAA_METCA    IARRVRELGVYSEIHPYD.CGDDFITEF....APQGIILSGGPESVTGTG.TPRAPASVFTLG......VPVLGICYGMQTMAAQL.GGKVE
GUAA_NITOC    IARRVREAGVYCEIHPYD.MAESTLRDF....APRGIILSGGPASTVGET.APRLSPLIFELG......VPLLGICYGMQVMAAQL.GGRVE
GUAA_LEGPL    IARRVREMGVYCEIYPYN.INHEQFKKL....NPCGVILSGGPSTVTHDA.NPRAPQWLFESD......LPLLGICYGMQTMAVQL.GGQVH
GUAA_LEGPA    IARRVREMGVYCEIYPYN.INHEQFKKL....NPCGVILSGGPSTVTHDA.NPRAPQWLFESD......LPLLGICYGMQTMAVQL.GGQVH
GUAA_LEGPC    IARRVREMGVYCEIYPYH.INHEQFKKL....NPCGVILSGGPSTVTHDA.NPRAPQWLFESD......LPLLGICYGMQTMAVQL.GGQVH
GUAA_LEGPH    IARRVREMGVYCEIYPYH.INHEQFKKL....NPCGVILSGGPSTVTHDA.NPRAPQWLFDSG......LPLLGICYGMQTMAVQL.GGQVH
GUAA_ACIAD    IARRVREAGVYSEMYAFD.MSEEDIRAF....NPNGIILSGGPESVHEEG.SPRAPQVVFELG......VPVLGICYGFQTMSEQL.GGKVE
GUAA_PSYA2    IARRVRDSGVFCEMFPYD.IDTQRITDF....GAKGIILSGGPESVHAEN.SPRINDAVFELG......VPVLGICYGMQAMAERF.GGKVH
GUAA_RUTMC    IARRVREIGVYCELFPYD.VDSEFIKKL....NPKGIILSGGPDTVTLDN.AAKAPSIIFDLN......IPILGICYGMQTMAIQL.GGRAT
GUAA_FRAP2    IARRVREVGVFCEIFPHD.IADDFIKDY....HAKGIILSGGPESVYDS..DVKAPEIVFELG......VPVLGICYGMQTMVMQH.GGEVK
GUAA_BURPP    IARRVREANVYSEIHPYD.VDASFIRDF....APKGVILSGGPSSVTETD.TPRVPQAVFELG......VPVLGICYGMQAMAEQL.GGKVD
GUAA_BURXL    IARRVREANVYSEIHPYD.VDASFIRDF....APKGVILSGGPSSVTETD.TPRVPQAVFELG......VPVLGICYGMQAMAEQL.GGKVD
GUAA_BURP8    IARRIREAHVLSEIHPHD.VSDEFIRDF....KPTGIILSGGPNSVTETD.TPRAPQAVFEAG......VPVLGICYGMQTMAEQL.GGKVD
GUAA_POLNS    IARRVRDGRVYSEIHPYD.CDPELIRKFIQEQGGKGIILSGGPSSVTEEG.SPRAPQIVFELG......VPVLGICYGMQTMATQL.GGAVA
GUAA_POLSQ    IARRVRDARVYSEIHPYD.CDPEFIRKFIQEQGGKGIILSGGPNSVTEDG.SPRAPQIVFELG......VPVLGICYGMQTMATQL.GGAVA
GUAA_DECAR    IARRVREAKVFCEIHPYD.VSEEFIRNY....GAKGIILSGGPSSVTEGD.TPRAPEVVFQLG......VPVLGICYGMQTMAQQL.GGKVM
GUAA_HERAR    IARRVRDSGVFSEVYPYD.VSDEFVRNY....GAAGVILSGGPNSVIEGDESPRVPQAVFELG......VPVLGICYGMQAMAEQL.GGKVE
GUAA_JANMA    IARRVRDSGVFSEVFPYD.VSDEFVRNY....GAAGVILSGGPNSVIEGDESPRVPQAVFELG......VPVMGICYGMQAMAEQL.GGKVE
GUAA_AROAE    IARRVREQQVYCEIHPFD.VSDDFVREF....APAGVILSGGPNSVYEAV.GWHAPQSVFELG......VPVLGICYGMQTMAQQL.GGSVE
GUAA_AZOSB    IARRVREQHVYCELHPYD.VSEAFIRDF....KPQGIILSGGPNSVYEAV.DWRAPQVVFELG......VPVLGICYGMQTMAEQL.GGKVE
GUAA_METFK    IARRIREANVYCELHSWD.VSDAFIREF....NPAGVILSGGPNSVYEDE.TPRAPQSVFELG......VPVLGICYGMQTMAAQL.GGSVE
GUAA_THIDA    IARRVREAGVYCELHPND.VSEDFVREF....APQGIILSGGPNSVYEDE.TPRAPDVVFTLG......VPVLGICYGMQTMAAQL.GGAVE
GUAA_NITEC    IARRIRETSVYCELHPFD.VSPQFIREF....SPVGIILSGGPASIFTND.APRVPQIVFELG......VPVLGICYGMQAMAAQL.GGEVE
GUAA_NITEU    IARRIRETNVYCELHPFD.VSPQFIREF....APIGIILSGGPASTFTED.APRVPQIVFELG......VPVLGICYGMQAMAAQL.GGEVE
GUAA_NITMU    IARRVRESNAYCEIHPFD.ISSQFIGNF....APAGIILSGGPASVAENE.TPRAPQIVFELG......VPVMGICYGMQTMAAQL.GGSVE
GUAA_NEIMA    IARRVREAHVYCELHSFD.MPLDEIKAF....NPKGIILSGGPNSVYESD..YQADTGIFDLG......IPILGICYGMQFMAHHL.GGEVQ
GUAA_NEIMF    IARRVREAHVYCELHSFD.MPLDEIKAF....NPKGIILSGGPNSVYESD..YQADTGIFDLG......IPILGICYGMQFMAHHL.GGEVQ
GUAA_NEIG1    IARRVREAHVYCELHSFD.MPLDEIKAF....NPKGIILSGGPNSVYESD..YQADTGIFDLG......IPVLGICYGMQFMAHHL.GGEVQ
GUAA_NEIG2    IARRVREAHVYCELHSFD.MPLDEIKAF....NPKGIILSGGPNSVYESD..YQADTGIFDLG......IPVLGICYGMQFMAHHL.GGEVQ
GUAA_NEIM0    IARRVREAHVYCELHSFD.MPLDEIKAF....NPKGIILSGGPNSVYESD..YQADIGIFDLG......IPVLGICYGMQFMAHHL.GGEVT
GUAA_NEIMB    IARRVREAHVYCELHSFD.MPLDEIKAF....NPKGIILSGGPNSVYESD..YQADTGIFDLG......IPVLGICYGMQFMAHHL.GGEVQ
GUAA_GEOMG    IARRVREAHVYCELHPFD.MDLAAIRAF....APRGIILSGGPKSVYEEG.APAVEEALFELG......VPVLGICYGMQLMSRHF.GGQVV
GUAA_GEOSL    IARRVREAHVYCELHPFD.MGLEAIRAF....APKGIILSGGPKSVYEEG.APAVEEALFDLG......VPVLGICYGMQLMSRHF.GGEVV
GUAA_GEODF    IARRVREAHVYCELHPFD.LELAAIRSF....APSGIILSGGPKSVYEEG.APAVAEELFELG......VPVLGICYGMQLMSRHF.GGEVV
GUAA_PELPD    IARRVREAHVFCELHPFD.MEMAAIRAF....NPSGIILSGGPKSVYEPG.APAVAEELFELG......VPVLGICYGMQLMSRHF.GGEVV
GUAA_PELCD    IARRVREAHVYCELHPFD.MDLDAIRAF....GPKGIILSGGPKSVYEEG.APVVEEALFELG......IPVLGICYGMQLMSRHF.GGRVV
GUAA_BARHE    IARRVRAMGVYCEVVPFQ.LALEGMQR....IKPKAVILSGSPYSVIDEG.SPRAPMEIFEMD......IPVLGICYGQQIMCVQL.GGKVA
GUAA_BARQU    IARRVRAMGVYCEIVPFQ.LALEGVKR....LRPQAVILSGSPYSVIDDG.SPRAPMEIFEIG......VPVLGICYGEQVMCVQL.GGKVE
GUAA_BARBK    IARRVRAMGVYSEIVPFQ.SALGGINR....IKPKAVILSGSPYSTLDNG.SPRAPIEVFEAG......IPVLGICYGQQVMCVQL.GGKVE
GUAA_RHIE6    IARRVREAGVYCEIVPFQ.SAEEGFKR....LQPKAVILSGSPASTVDEG.SPRAPQIIFDSG......LPVFGICYGQQTMCMQL.GGKVE
GUAA_RHILW    IARRVREAGVYCEIVPFQ.SAEEGFNR....LQPKAVILSGSPASTVDEG.SPRAPQIVFDSG......LPVFGICYGQQTMCMQL.GGKVE
GUAA_RHIEC    IARRVREAGVYCEIVPFQ.SAEEGFKR....LQPKAVILSGSPASTVDEG.SPRAPQIIFDSG......LPVFGICYGQQTMCMQL.GGKVE
GUAA_RHIL3    IARRVREAGVYCEIVPFQ.SAEEGFHR....LQPKAVILSGSPASTVDEG.SPRAPDIIFDSG......LPVFGICYGQQTMCMQL.GGKVE
GUAA_AGRRK    IARRVREAGVYCEIVPFQ.SADAGFKR....LQPKAVILSGSPASTVDEG.SPRAPQIIFDSG......VPVFGICYGQQTMCMQL.GGKVE
GUAA_RHISN    IARRVREAGVYCEIVPFQ.SAEEGFKR....LKPKAVILSGSPASTLDIG.SPRAPSVIFESG......LPVFGICYGQQTMCAQL.GGKVE
GUAA_RHIME    IARRVRESGVYCEIVPFQ.SAEEGFHR....LKPKAVILSGSPASTLDTG.SPRAPSVIFDSG......LPVFGICYGQQTMCAQL.GGKVE
GUAA_SINMW    IARRVRESGVYCEIVPFQ.SAEDGFHR....LKPKAVILSGSPASTLDVG.SPRAPSVIFESG......LPVFGICYGQQTMCAQL.GGKVE
GUAA_AGRVS    IARRVRETGVYCEIVPFQ.SSEEGFAR....LKPKAVILSGSPASALDEG.SPRVPQVIFDSG......LPIFGICYGQQTICAQL.GGKVE
GUAA_BRUSI    IARRVREANVYCEIVPFQ.SADEAFKR....LQPKGVILSGSPHSTTDIG.SPRAPQAIFDAG......IPVLGICYGEQTMCAQL.GGNVE
GUAA_BRUAB    IARRVREANVYCEIVPFQ.SADEAFKR....LQPKGVILSGSPHSTTDIG.SPRAPQAIFDAG......IPVLGICYGEQTMCAQL.GGNVE
GUAA_BRUA2    IARRVREANVYCEIVPFQ.SADEAFKR....LQPKGVILSGSPHSTTDIG.SPRAPQAIFDAG......IPVLGICYGEQTMCAQL.GGNVE
GUAA_BRUA1    IARRVREANVYCEIVPFQ.SADEAFKR....LQPKGVILSGSPHSTTDIG.SPRAPQAIFDAG......IPVLGICYGEQTMCAQL.GGNVE
GUAA_BRUO2    IARRVREANVYCEIVPFQ.SADEAFKR....LQPKGVILSGSPHSTTDIG.SPRAPQAIFDAG......IPVLGICYGEQTMCAQL.GGNVE
GUAA_BRUSU    IARRVREANVYCEIVPFQ.SADEAFKR....LQPKGVILSGSPHSTTDIG.SPRAPQAIFDAG......IPVLGICYGEQTMCAQL.GGNVE
GUAA_BRUC2    IARRVREANVYCEIVPFQ.SADEAFKR....LQPKGVILSGSPHSTTDIG.SPRAPQAIFDAG......IPVLGICYGEQTMCAQL.GGNVE
GUAA_BRUMB    IARRVREANVYCEIVPFQ.SADEAFKR....LQPKGVILSGSPHSTTDIG.SPRAPQAIFDAG......IPVLGICYGEQTMCAQL.GGNVE
GUAA_RHILO    IARRIREAGVFSEIVPFQ.SAEAAFKR....INPKAVILSGGPASTSDIG.SPRAPQIVFDAG......VPVLGICYGQMAMCVQM.GGVAE
GUAA_AGRT5    IARRIREAGVYCEIHPFQ.NAAEAFEK....LQPKGVIFSGGPASVTAEG.SPRAPQAVFDSK......VPILGICYGQQTLCTQL.GGVVE
GUAA_BRADU    IARRVREDGVYCEIVPFN.KAEEAFKE....MKPKAVILSGGPESVHEAG.SPRAPQLIFASG......VPVMGICYGQMTMAAQL.GGEVE
GUAA_BRASO    IARRVREDGVYCEIVPFQ.KAEAAFRQ....MNPKAVILSGGPESVHEEG.SPRAPQLIFESG......VPVMGICYGQMTMAAQL.GGTVE
GUAA_OLICO    IARRVREEGVYSEIVPFQ.KAEQAFTA....MQPKAVILSGGPASVLEAG.APSAPMSIFTAG......VPVLGICYGEQTMAQQL.GGMVE
GUAA_RUEST    IARRLRELNVYCEIHPYQ.NVTMDFVRE...FAPKAVIFSGGPDSVTREG.SPRAPQEIFDYG......VPILGICYGQQVMMHQL.GGTVQ
GUAA_RUEPO    IARRLRELNVYCEIHPYQ.NVTMDFVRE...MAPRAVIFSGGPDSVTREG.SPRAPQEIFDYG......VPILGICYGQQTMMTQL.GGRVE
GUAA_CAUCR    IARRVREAGVYCEIHPFD.KAEAIVDE....YAPSAIILSGGPASVLEAD.SPRIGRKLFDLG......LPLLAICYGQQLLCDVL.SGKVE
GUAA_CAUCN    IARRVREAGVYCEIHPFD.KAEAIVDE....YAPSAIILSGGPASVLEAD.SPRIGRKLFDLG......LPLLAICYGQQLLCDVL.SGKVE
GUAA_PHEZH    IARRLRESGVYCEIHPYD.KVDAVLDS....FRPKAVILSGGPASVHEAE.SPAASKRLFELD......VPVLGICYGEQTMCDVL.GGKVE
GUAA_RHORT    IARRVRESGVYSEIQPFN.TVTAASIAA...FAPKGVILSGGPASVTAAD.TPRAPIELFTMG......LPVLGICYGQQTMVAQL.GGRVE
GUAA_GLUOX    IARRVRESGVYCEIWPF..TATEEKIRA...FNPRGIILSGSPASVHDEN.APPIPNVVFALN......RPVLGICYGQQAMCQML.GGKVE
GUAA_RHOBA    IARRVREQNVYCQIVRHD.ISAERIAEL....APKGIILSGGPNSVYEEG.APKCDEGLFDLG......IPVLGICYGMQLACQAL.GGKVD
GUAA_BACFR    IGRRVRELDTYCEIVPYN.KFPKGDETV......KGVILSGSPFSVYDES.AFKVDLSEIRGK......YPILGICYGAQFMAYTN.GGKVE
GUAA_BACFN    IGRRVRELDTYCEIVPYN.KFPKGDETV......KGVILSGSPFSVYDES.AFKVDLSEIRGK......YPILGICYGAQFMAYTN.GGKVE
GUAA1_BACTN   IGRRVRELDTYCEIVPYN.KFPKEDPTI......KGVILSGSPFSVYDKD.AFKVDLSEIRGK......YPILGICYGAQFMAYTN.NGKVE
GUAA_CYTH3    IARRVRELNVYCEIHPYN.KAPQIDGTV......KGVILSGSPCSVREEN.APDIDLSLYREK......LPLLGVCYGAQLIAHKS.GGTVQ
GUAA_PELLD    IARRIRELGIYSEILPYS.TTPEAIREH....DPKAIILSGGPTSVYGES.AILPHPGIFSLG......LPILGICYGLQAIANHFGG..AV
GUAA_PROVI    IARRIRELGIYSEILPYS.TTPETIREH....NPRAIILSGGPTSVYGDS.AILPHPGIFSLG......LPILGICYGLQAIANHFGG..AV
GUAA_CHLL2    IARRIRELGIYSEILPYN.TTPETIREH....APKAIILSGGPTSVYGES.AILPHEGIFSLG......LPILGICYGLQAIAKHFGG..EV
GUAA_CHLPD    IARRIRELGIYSEILPYN.ASPETIREH....DPKAIILSGGPTSVYGDS.ALLPDGGVFMLG......VPVLGICYGLQAIAKHFGG..EV
GUAA_PELPB    IARRIRELGIYSEILPYN.TTPESIREH....NPKAIILSGGPTSVYDAS.AILPDDGIFSLG......IPMLGICYGLQVIAKHFGG..EV
GUAA_CHLTE    IARRIREIGIYSEIFPYH.TKAETIRAH....QPKAIILSGGPNSVYDEK.AFMPDPEVFSLG......VPVLGICYGLQAIAKHFGG..NV
GUAA_CHLCH    IARRIRELNIYSEILPYN.TPADTIRTH....QPKAIILSGGPNSVYDQT.AFMPDPAIFSLG......IPVLGICYGLQAIAKHFGG..VV
GUAA_CHLPB    IARRIREIGIYSEIVPYN.TPLEKIREH....NPGAIILSGGPSSVYGES.AFHPDEGIFKLG......VPILGICYGLQVIATHFGG..SV
GUAA_CHLT3    IARRIRELGIYSEILPYH.TSAEALRER....NPKALILSGGPTSVYDAF.APMPDEKIYELD......IPILGICYGLQAIAKHFGG..EV
GUAA_SALRD    IARRVRETGVYSEIRPCT.VEADAIDAL....NPSGVILSGGPCSVYDEA.GPALDPALLELERPDGTPVPLLGICYGLQAMAHQFGG..EV
GUAA_GEMAT    IARRVREARVYSEIHPPT.KSLDWIRAW....KPTGIILSGGPSSVTDEG.APTIPPALLDVA.......PVLGVCYGMQLIAHLEGG..SV
GUAA_KLULA    ITRRLREFNVYAEMLPCT.QKIADLH.W....KPKGVILSGGPYSVYEKD.APHVDKAIFDLG......VPILGICYGLQEIAWINNT..EV
GUAA_ASHGO    ITRRLREFNVYAEMLPCT.QKISELG.W....KPKGVILSGGPYSVYAAD.APHVDRAVFELG......VPILGICYGLQELAWIAGA..EV
GUAA_YEAST    ITRRLREFNIYAEMLPCT.QKISELG.W....TPKGVILSGGPYSVYAED.APHVDHAIFDLN......VPILGICYGMQELAWINGK..QV
GUAA_CANGA    ITRRLREFNIYAEMLPCT.QKIADLH.F....KPKGVIMSGGPYSVYAED.APHVDHAIFDLG......VPILGICYGMQELAWINGK..QV
GUAA_DEBHA    ITRRLREFNVYAEMLPCT.QKISELT.W....KPKGIILSGGPYSVYEDG.SPHVDHDIFKLN......VPILGICYGMQELAWINGK..GV
GUAA_CANAL    ITRRLREFNVYAEMLPCT.QKIAELS.W....KPKGIILSGGPYSVYAED.APHVDHDIFKLG......VPILGICYGMQELAWINGK..GV
GUAA_YARLI    ITRRLREFNVYAEMLPCT.QKIAELP.W....KPKGVILSGGPYSVYAEG.SPHVDHAVFDLG......VPILGICYGMQELAWINGK..GV
GUAA_PHANO    ITRRLRELNVYSEMLPCN.TKLADLP.F....TPKGIILSGGPYSVYEEG.APHVDHAVFDLG......VPILGICYGLQEMAWHFGKNAGV
GUAA_EMENI    ITRRLREINVYSEMLPCT.QKLADLG.W....KPKGIILSGGPYSVYEEG.APHADPAFFELG......VPILGICYGLQELAHRLHAD.NV
GUAA_ASPOR    ITRRLRELNVYSEMLPCT.QKLADLK.F....KPAGVILSGGPYSVYEEG.APHVDPAYFDLG......VPILGICYGLQEIAYRLDST.NV
GUAA_ASPFU    ITRRLREIGVYSEMLPCT.QKLADLP.F....KPKGIILSGGPYSVYEDG.APHADPAVFELG......VPVLGICYGLQEIAYRLGKD.NV
GUAA_USTMA    ITRRCRELNVYCEMLPCT.QKIKDLD.W....KPKGIILSGSPYSVYDEV.APRVDPDVFTAG......VPVLGICYGLQEIAWNHGG..KV
GUAA_DEHM1    IARRIREMHVYCELVSHD.TPWEKIAHL....NPRGFILSGGPSSVYEAG.APLAPAYIFESK......LPVLGICYGMQAITHQL.GG.VV
GUAA_DEHMC    IARRIREMHVYCELVSHD.TPWEKIAHL....NPRGFILSGGPSSVYEPE.APLAPAYIFESK......LPVLGICYGMQAITQQL.GG.VV
GUAA_DESPS    IARRVREMKVYSEIHPFS.LPLEKLKEL....NPTGIILSGGPCSVYDDD.APHSDAGLFELG......VPVFGICYGAQLMIQQL.GG.SV
GUAA_BRAHW    ITRRIRELNVYSEIHPFH.VSIDFIKEF....KPKAIILSGGPSSVYEED.APKVDKQLFELG......MPILGICYGMQIIVYSM.GG.KV
GUAA_DESVV    IARRVREAGVYSEIHPCI.VTAEQVRAM....KPAAIILSGGPASVGEKD.APALEKGLLDLG......VPVLGICYGMQLLGQDL.GG.EL
GUAA_DESVH    IARRVREAGVYSEIHPCI.VTAEQVRAM....KPAAIILSGGPASVGEKD.APALEKGLLDLG......VPVLGICYGMQLLGQDL.GG.EL
GUAA_DESVM    IARRVREAGVYSEIHPCI.VTADQVRAM....KPAAVILSGGPASVGEAD.APTLEKGLLELG......VPVLAICYGMQLLGHNL.GG.QL
GUAA_DESAG    IARRIREAGVYSEIHPCI.VTAAEVAAM....SPSAVVLSGGPASVLGED.SPALDKGLLELG......VPVLGICYGMQLLAHNL.GG.EL
GUAA_DESMR    IARRVREAGVYSEIHPCT.VTAKEVAAM....APKAVILSGGPSSVADAD.APPFDPAVFDLG......LPMLCICYGMQLLAHHLPGG.QV
GUAA_DESAD    IARRIREAGVYSEIHPCN.VDPQKIKDL....NPGALILSGGPSSVLEDE.SPQLDPSLLELG......VPVLGICYGMQLMTNDL.GG.RV
GUAA_CALBD    IARRVRECGVYCEIWPYD.TPLEKIISK....NPKGIIFTGGPSSVYEPN.APMIDKELFEKG......IPILGICYGNQLIAHIM.GGKVS
GUAA_CALS8    IARRVRECGVYCEIWPYD.TPLEKIIEK....NPKGIIFTGGPSSVYEEN.APVVDKKIFEIG......VPILGICYGNQLIAYFL.GGKVS
GUAA_CARHZ    IARRIRELKVYCEMLPYN.TPLEKIVQE....NPGGIVFSGGPSSVYGEG.APTVDPEIYRLN......IPILGICYGMQLMAHQL.GGIVR
GUAA_CLOBL    IARRVRENRVYCEIVPYT.TSIEDIKEK....APKGIIFTGGPNSVYGEN.APRVQKELFDLG......IPVLGICYGDQLMAHSL.EGEVT
GUAA_CLOBK    IARRVRENRVYCEIVPYT.TSIEDIKEK....APKGIIFTGGPNSVYGEN.APRVQKELFDLG......IPVLGICYGDQLMAHSL.EGEVT
GUAA_CLOBM    IARRVRENRVYCEIVPYT.TSIEDIKEK....APKGIIFTGGPNSVYGEN.APRVRKELFDLG......IPVLGICYGDQLMAHSL.EGEVT
GUAA_CLOBJ    IARRVRENRVYCEIVPYT.TSIEDIKEK....APKGIIFTGGPNSVYGEN.APRVQKELFDLG......IPVLGICYGDQLMAHSL.EGEVT
GUAA_CLOB6    IARRVRENRVYCEIVPYT.TSIEDIKEK....APKGIIFTGGPNSVYGEN.APRVQKELFDLG......IPVLGICYGDQLMAHSL.EGEVT
GUAA_CLOBH    IARRVRENRVYCEIVPYT.TSIEDIKEK....APKGIIFTGGPNSVYGEN.APRVQKELFDLG......IPVLGICYGDQLMAHSL.EGEVT
GUAA_CLOB1    IARRVRENRVYCEIVPYT.TSIEDIKEK....APKGIIFTGGPNSVYGEN.APRVQKELFDLG......IPVLGICYGDQLMAHSL.EGEVT
GUAA_CLONN    IARRVREHNVYCEIIPYT.TSVEDIKKK....NPKAIIFTGGPNSVYGEG.TPRVEKEIFELG......VPVLGICYGHQLTAYTL.GGKVE
GUAA_CLOAB    IARRVREHNVYCEIVPYT.YSIDKIKEK....KPSAVIFTGGQNSVYGED.SPRMDKEIFDLG......VPVLGICYGHQLITYTL.GGEVM
GUAA_CLOTE    IARRVRENNVYCEIIPYS.TSIEKIKEK....NPKGIIFSGGPNSVYGEN.SPRISKEIFEID......VPVLGICYGQQLASFIL.GGKVE
GUAA_CLOPE    IARRVRECGVYCEIIPYT.YSIEKIKEK....NPKGIIFTGGPNSVYGEN.TPTLDKEIFNLN......VPVLGICYGDQLMAHLL.GGKVD
GUAA_CLOB8    IARRVRECGVYCEIIPYD.YTIEKIKAK....NPKGIIFTGGPNSVYGED.TPTVEKEVFELG......VPVLGICYGDQLMAHLL.GGKVA
GUAA_ALKMQ    IARRVREHNIYCEVVPYK.ITAEEIKLK....NPKGLILTGGPSSVYGES.TPRCEEEIFQLD......IPILGICYGGQLMAHTL.GGKVN
GUAA_CLOTH    IARRVREANVYCEVLPYN.SSIDKIKSK....NPKGIIFTGGPASVLDPK.APICDREVFELG......IPILGICYGMQLMSHML.GGTVE
GUAA_CLOCE    IARRVREANVYCEVIPYN.ASLERIKSY....NAKGIIFTGGPNSVLDEG.APKCDPGVFELG......IPVLGICYGMQLMSVML.GGSVT
GUAA_CLOBA    IARRVRECNVYCEVHPYN.LSVDEIKQM....NPKGIIFTGGPNSVYGEN.SPLCDKAIFELG......VPIFGICYGSQLMSHML.GGKVA
GUAA_PEPD6    IARRVRENNVYCEILPCT.ASIERIKEK....NPKGIIFTGGPNSAYLED.SPTISKEIFELG......VPILGICYGIQIMSHVL.GGVVR
GUAA_FINM2    IARRVRDLNVYCEVVPYK.KALDVIKEK....QPIGIIFTGGPNSVYEEN.SPQIEKEIFELN......IPILGMCYGMQLISKDF.GGVVE
GUAA_TREDE    IARRVRDLNVYSDIVPAS.KALDYIREN....KPIGIIFTGGPNSVYEEK.APLPPKEIFNLN......IPILGICYGMQAMAHCL.GGKVE
GUAA_BACCR    IARRIREFGVYSELHPHT.ITAEEIKAM....NPKGIIFSGGPNSVYGEG.ALHCDEKIFELG......LPIFGICYGMQLMTQQF.GGKVE
GUAA_BACC4    IARRIREFGVYSELHPHT.ITAEEIKAM....NPKGIIFSGGPNSVYGEG.ALHCDEKIFELG......LPIFGICYGMQLMTQHF.GGKVE
GUAA_BACC2    IARRIREFGVYSELHPHT.ITAEEIKAM....NPKGIIFSGGPNSVYGEG.ALHCDEKIFELG......LPIFGICYGMQLMTQHF.GGKVE
```

```
GUAA_BACWK             IARRIREFGVYSELHPHT.ITAEEIKAM....NPKGIIFSGGPNSVYGEG.ALHCDEKIFELG......LPIFGICYGMQLMTQHF.GGKVE
GUAA_BACCZ             IARRIREFGVYSELHPHT.ITAEEIKAM....NPKGIIFSGGPNSVYGEG.ALHCDEKIFDLG......LPIFGICYGMQLMTQQF.GGTVE
GUAA_BACC3             IARRIREFGVYSELHPHT.ITAEEIKAM....NPKGIIFSGGPNSVYGEG.ALHCDEKIFDLG......LPIFGICYGMQLMTQQF.GGTVE
GUAA_BACAH             IARRIREFGVYSELHPHT.ITAEEIKAM....NPKGIIFSGGPNSVYGEG.ALHCDEKIFDLG......LPIFGICYGMQLMTQQF.GGTVE
GUAA_BACAC             IARRIREFGVYSELHPHT.ITAEEIKAM....NPKGIIFSGGPNSVYGEG.ALHCDEKIFDLG......LPIFGICYGMQLMTQQF.GGTVE
GUAA_BACAA             IARRIREFGVYSELHPHT.ITAEEIKAM....NPKGIIFSGGPNSVYGEG.ALHCDEKIFDLG......LPIFGICYGMQLMTQQF.GGTVE
GUAA_BACHK             IARRIREFGVYSELHPHT.ITAEEIKAM....NPKGIIFSGGPNSVYGEG.ALHCDEKIFDLG......LPIFGICYGMQLMTQQF.GGTVE
GUAA_BACC0             IARRIREFGVYSELHPHT.ITAEEIKAM....NPKGIIFSGGPNSVYGEG.ALHCDEKIFDLG......LPIFGICYGMQLMTQQF.GGTVE
GUAA_BACAN             IARRIREFGVYSELHPHT.ITAEEIKAM....NPKGIIFSGGPNSVYGEG.ALHCDEKIFDLG......LPIFGICYGMQLMTQQF.GGTVE
GUAA_BACC1             IARRIREFGVYSELHPHT.ITAEEIKEI....NPKGIIFSGGPNSVYGEG.ALHCDEKIFDLG......LPIFGICYGMQLMTQKF.GGKVE
GUAA_BACCN             IARRIREFGVYSELHPHT.ITAEEIKAM....NPKGIIFSGGPNSVYGEN.AFHCDEKIFELG......LPIFGICYGMQLMTKHF.GGKVE
GUAA_BACA2             ITRRIREFGVYSELHPHT.LTAEEIKEM....NPKGIILSGGPNSVYDEG.SFRCDEKIFELD......IPVLGICYGMQLMTHYL.GGKVE
GUAA_BACSU             ITRRIREFGVYSELHPHT.LTAEEIKKM....NPKGIILSGGPNSVYDEN.SFRCDEKIFELD......IPVLGICYGMQLMTHYL.GGKVE
GUAA_BACLD             ITRRIREFGVYSELHPHT.LTAEEIKKM....NPKGIILSGGPNSVYDEG.SFRCDEKIFELD......IPVLGICYGMQLMTHYL.GGKVE
GUAA_BACP2             ITRRIREFGVYSELHPHT.LTAEEIKEM....APKGIILSGGPNSVYDEG.SFRCDEKIFDLD......IPVLGICYGMQLMTHYL.GGKVE
GUAA_BACSK             ITRRIRDLGVYSELHSHK.LTADELKEM....NPIGIIFSGGPNSAYVEG.SPKCDPAIYDLG......IPILGICYGVQLMTHHF.GGKVD
GUAA_BACHD             ITRRIRDLGVYSELHPNT.ITAEQLKEM....KPKGIIFSGGPNSAYAEG.APKCDPAIFDLG......VPILGICYGMQLMTQHF.GGKVD
GUAA_GEOKA             ITRRIREFGVYSELHPHT.IRAEEIRAL....NAKGIIFSGGPNSVYDEQ.AFTCDPAIFELG......LPILGICYGMQLMAHHL.GGKVE
GUAA_LISMF             ITRRIREFGVYSELHPHT.ITVEEMKAL....NPTGIIFSGGPNSVYDED.AFRADERIFDMG......IPILGICYGMQLMTTHF.GGKVE
GUAA_LISMO             ITRRIREFGVYSELHPHT.ITVEEMKAL....NPTGIIFSGGPNSVYDED.AFRADERIFDMG......IPILGICYGMQLMTTHF.GGKVE
GUAA_LISW6             ITRRIREFGVYSELHPHT.ITVEEMKAL....NPTGIIFSGGPNSVYDKD.AFRADERIFDMG......IPILGICYGMQLMTTHF.DGKVE
GUAA_LISIN             ITRRIREFGVYSELHPHT.ITVEEMKAL....NPTGIIFSGGPNSVYDED.AFRADERIFDMG......IPILGICYGMQLMTTHF.GGKVE
GUAA_EXIS2             ITRRIRDLGVYSELHSHK.ITAAEVKDI....APAGIIFSGGPRSVYAED.AYRCDPEIFDLG......IPIFGICYGMQLMSQHF.GGTVE
GUAA_STAES             ITRRIREMGVYSELHDHE.ISIEEIKRM....NPKGIILSGGPNSVYEEG.SFTIDPEIYNLG......IPVLGICYGMQLTTKLL.GGKVE
GUAA_STAEQ             ITRRIREMGVYSELHDHE.ISIEEIKRM....NPKGIILSGGPNSVYEEG.SFTIDPEIYNLG......IPVLGICYGMQLTTKLL.GGKVE
GUAA_MACCJ             ITRRIREMGVYSELHDHE.ISIEEIKKM....NPKGIILSGGPNSVYEEG.SFTVDPEIFNLG......VPVMGICYGMQLMTKLL.GGSVE
GUAA_OCEIH             ITRRIREFGVYSELHSHK.LTAEEIKEM....NPKGIILSGGPHSVYDEN.SFRCDEGIFELG......IPVLGICYGMQLMSLHY.DGKVE
GUAA_LACRJ             ITRRLRDFGIYSELLSHK.LTADEIKEI....NPKGIIFSGGPNSVYDPN.AFKVDPEIFKLG......IPILGICYGMQLMSYDL.GGKVE
GUAA_LACRD             ITRRLRDFGIYSELLSHK.LTADEIKEI....NPKGIIFSGGPNSVYDPN.AFKVDPEIFKLG......IPILGICYGMQLMSYDL.GGKVE
GUAA_LACJO             ITRRIRDFGIYSELLPHD.LSIEKIKEM....APKGIIFSGGPNSVYDKG.ALKVDPEIFKLG......IPILGICYGMQLMSYDL.GGKVE
GUAA_LACGA             ITRRIRDFGIYSELLPHD.LSIEKIKKM....APKGIIFSGGPNSVYDEG.ALKVDPEIFKLG......IPILGICYGMQLMSYDL.GGKVE
GUAA_LACAC             ITRRIRDFGIYSELLPHD.LSIEKIKEM....NPKGIIFSGGPNSVYDDG.ALKVDPEIFKLG......IPILGICYGMQLMSYDL.GGKVE
GUAA_LACDB             ITRRIRDFGIYSELLPHT.ITAEEVKKI....APKGIIFSGGPNSVYDEN.ALDVDPEIFELG......IPILGICYGMQLMTQKL.GGTVE
GUAA_LACDA             ITRRIRDFGIYSELLPHT.ITAEEVKKI....APKGIIFSGGPNSVYDEN.ALDVDPEIFELG......IPILGICYGMQLMTQKL.GGTVE
GUAA_LACCB             ITRRIREFGIYSELKPHT.ITAAEVKKI....APKGIIFSGGPNSVYDEG.ALGVDEDLFKLG......IPILGVCYGMQLMAQRL.GGDVE
GUAA_LACC3             ITRRIREFGIYSELKPHT.ITAAEVKKI....APKGIIFSGGPNSVYDEG.ALGVDEDLFKLG......IPILGVCYGMQLMAQRL.GGDVE
GUAA_LACRH             ITRRIREFGIYSELKPHT.ITAAEVKKI....APKGIIFSGGPNSVYDEG.ALGVDEDIFKLG......IPILGVCYGMQLMAQRL.GGDVE
GUAA_LACS1             ITRRIREFGVYSELLSHK.LTAAQIKEM....NPKGIIFSGGPNSVYDDG.AFQIDPEIFELG......IPILGICYGMQLMAHNLSGGKVE
GUAA_LACLM             IARRIREIGVFSELMSHK.VTAKEIREI....NPIGIILSGGPNSVYDEG.SFDIDPEIFELG......LPVLGICYGMQLMSYKL.GGMVE
GUAA_LACLS             IARRIREIGVFSELMSHK.VTAKEIREI....NPIGIILSGGPNSVYDEG.SFDIDPEIFELG......LPVLGICYGMQLMSYKL.GGMVE
GUAA_LACLA             IARRIREIGVFSELMSHK.VTAKEIREI....NPIGIILSGGPNSVYDEG.SFDIDPEIFELG......LPVLGICYGMQLLSYKL.GGMVE
GUAA_STRZJ             ISRRIREIGVFSELKSHK.ISAAEVREV....NPVGIILSGGPNSVYEDG.SFDIDPEIFELG......IPILGICYGMQLLTHKL.GGKVV
GUAA_STRPJ             ISRRIREIGVFSELKSHK.ISAAEVREV....NPVGIILSGGPNSVYEDG.SFDIDPEIFELG......IPILGICYGMQLLTHKL.GGKVV
GUAA_STRZT             ISRRIREIGVFSELKSHK.ISAAEVREV....NPVGIILSGGPNSVYEDG.SFDIDPEIFELG......IPILGICYGMQLLTHKL.GGKVV
GUAA_STRR6             ISRRIREIGVFSELKSHK.ISAAEVREV....NPVGIILSGGPNSVYEDG.SFDIDPEIFELG......IPILGICYGMQLLTHKL.GGKVV
GUAA_STRPN             ISRRIREIGVFSELKSHK.ISAAEVREV....NPVGIILSGGPNSVYEDG.SFDIDPEIFELG......IPILGICYGMQLLTHKL.GGKVV
GUAA_STRPI             ISRRIREIGVFSELKSHK.ISAAEVREV....NPVGIILSGGPNSVYEDG.SFDIDPEIFELG......IPILGICYGMQLLTHKL.GGKVV
GUAA_STRP7             ISRRIREIGVFSELKSHK.ISAAEVREV....NPVGIILSGGPNSVYEDG.SFDIDPEIFELG......IPILGICYGMQLLTHKL.GGKVV
GUAA_STRP4             ISRRIREIGVFSELKSHK.ISAAEVREV....NPVGIILSGGPNSVYEDG.SFDIDPEIFELG......IPILGICYGMQLLTHKL.GGKVV
GUAA_STRP2             ISRRIREIGVFSELKSHK.ISAAEVREV....NPVGIILSGGPNSVYEDG.SFDIDPEIFELG......IPILGICYGMQLLTHKL.GGKVV
GUAA_STRZP             ISRRIRDIGVFSELKSHK.ISAAEVREV....NPVGIILSGGPNSVYEDG.SFDIDPEIFELG......IPILGICYGMQLLTHKL.GGKVV
GUAA_STRPS             ISRRIREIGVFSELKSHK.ISAAEVREV....NPVGIILSGGPNSVYEDG.SFDIDPEIFELG......IPILGICYGMQLLTHKL.GGKVV
GUAA_SYMTH             IARRVREAQVYCEIWPFT.APVQRYREA....GVKGIIFSGGPASVEQAD.APRIDPAIYELG......IPILGICYGMQLMALQL.GGEVR
GUAA_MOOTA             IARRVREAGVYSEMIPYN.TPLEKILAR....RPRGIIFSGGPASVYSLG.APRIDRALYESG......IPILGICYGMQLMAHDL.GGRVE
GUAA_HELMI             IARRVRELHVYCEMHPYT.ISVDAIREM....NPKGIIFTGGPASVYEEK.APAVDPAIYDLG......IPILGICYGMQLMVNQL.GGKVG
GUAA_HALOH             IARRVRECNVYSIIKPYN.ISIDQIKDI....NPAGIIFSGGPKSVTGPE.APMIDKEVFKLN......IPILGICYGMQLMTALLPGGAVE
GUAA_THETN             IARRIREANVYCEIVPYD.ISPEEIKKI....DPKGIVLSGGPASVYVKN.APKCDKEIFELG......YPVLGICYGVQLMTELL.GGKVA
GUAA_NATTJ             IARRIREHNVYCEIYPYN.ISINKLKQL....SPAAIVLSGGPSSVYQEG.SPDLDTEIFEIG......IPVLGICYGMQLMAKKL.GGTVT
GUAA_MYCTU             IARRVREARVFSEVIPHT.ASIEEIRAR....QPVALVLSGGPASVYADG.APKLDPALLDLG......VPVLGICYGFQAMAQAL.GGIVA
GUAA_MYCTA             IARRVREARVFSEVIPHT.ASIEEIRAR....QPVALVLSGGPASVYADG.APKLDPALLDLG......VPVLGICYGFQAMAQAL.GGIVA
GUAA_MYCBT             IARRVREARVFSEVIPHT.ASIEEIRAR....QPVALVLSGGPASVYADG.APKLDPALLDLG......VPVLGICYGFQAMAQAL.GGIVA
GUAA_MYCBP             IARRVREARVFSEVIPHT.ASIEEIRAR....QPVALVLSGGPASVYADG.APKLDPALLDLG......VPVLGICYGFQAMAQAL.GGIVA
GUAA_MYCBO             IARRVREARVFSEVIPHT.ASIEEIRAR....QPVALVLSGGPASVYADG.APKLDPALLDLG......VPVLGICYGFQAMAQAL.GGIVA
GUAA_MYCTO             IARRVREARVFSEVIPHT.ASIEEIRAR....QPVALVLSGGPASVYADG.APKLDPALLDLG......VPVLGICYGFQAMAQAL.GGIVA
GUAA_MYCMM             IARRVREARVFSEVIPHT.TSIEEIKAR....DPLALVLSGGPASVYAPG.APQLDPALFDLG......LPVFGICYGFQVMAQAL.GGTVA
GUAA_MYCPA             IARRVREARVFSEVIPHT.ATIEEIKAR....QPRALVLSGGPSSVYEPG.APQLDPAVFDLG......VPVFGICYGFQAMAQAL.GGTVA
GUAA_NOSS1             IARRIRETQVYSEVLSYR.TPAEHLRQL....NPKGIILSGGPSSVYSDR.APHCDPEIWNLG......VPILGVCYGMQLMVNQL.GGEVA
GUAA_ANAVT             IARRIRETQVYSEVLSYR.TSAEHLRQL....NPKGIILSGGPSSVYSDR.APHCDPEIWNLG......VPILGVCYGMQLMVNQL.GGEVA
GUAA_NOSP7             IARRIRETQVYSEVLSYR.TTSEQLRQL....NPKGIILSGGPSSVYGDN.APHCDPEIWNLG......IPVLGVCYGMQLMVNQL.GGEVA
GUAA_THEEB             IARRIRETQVYSEVISYR.TTAEQLAQL....APKGIILSGGPNSVYDEN.APQCDPEIWNLG......IPILGVCYGMQLMVQQL.GGTVE
GUAA_CYAA5             IARRIRETQVYSEVLSYR.TTAQKLRQL....NPKGIILSGGPNSVYDDR.APQCDPDIWQLG......IPVLGVCYGMQLMVKQL.GGQVE
GUAA_CYAP8             IARRIRETQVYSEVLSYR.TTIEQIRQL....NPRGIILSGGPNSVYDNR.APQCDPEIFHLG......VPILGVCYGMQLMVQQL.GGQVE
GUAA_CYAP7             IARRIRETNVYSEVLSYR.TSAEQLRQL....APKGIILSGGPNSVYDKN.APQCTQEIWDLG......IPILGVCYGMQLMVQQL.GGKVE
GUAA_SYNY3             IARRIRETEVYSEVLSYR.TTAQQLREI....KPKGIILSGGPNSVYDQG.APECDPEIFQLG......VPVLGVCYGMQLMVKQL.GGRVE
GUAA_SYNP2             IARRIRETEVYSEVISYR.TSAEQLRQL....NPSGIILSGGPSSVYDDY.APACDPEIWNLG......IPVLGVCYGMQLMVKQL.GGTVE
GUAA_SYNP6             IARRIRETQVYSEVLSYR.TTADQIRQL....SPKGIILSGGPNSVYDDY.APVCDPEIWNLG......IPVLGVCYGMQLMVQQL.GGQVD
GUAA_GLOVI             IARRIRETKVYSEVLSYQ.TPIAEIRRL....APKGIILSGGPNSVYEAY.APQCDPALWELG......IPILGVCYGMQLMVQQL.GGAVE
GUAA_PROMP             IARRIREADVFSLVVSNF.TSVKEIQDI....NPKGIILSGGPSSVYEDN.APKCDAKIFDLG......IPILGICYGMQLMVKEL.GGEVT
GUAA_PROM5             IARRIREANVFSLVVSNF.ISTKEIQDI....NPQGIILSGGPNSVYEDN.APKCDQNIFNLG......IPVLGICYGMQLMVKEL.GGTVT
GUAA_PROMT             IARRIRETEVYSLVMSYT.TSADQLRSL....KPKGIILSGGPGSVYEEG.APYCDPEIFNLG......IPVLGVCYGMQLMVHEL.GGSVK
GUAA_PROM1             IARRIRETEVYSLVMSYT.TSADKLRSL....KPKGIILSGGPGSVYEEG.APYCDPEIFNLG......IPVLGVCYGMQLMVHEL.GGSVK
GUAA_THERP             IARRVRDAHVYCELVPYD.TDWSALKHL....SPKGIILSGGPASVYEPD.APQLPSWVLESG......LPVLGICYGMQLLAHTL.GGRVA
GUAA_DEIGD             IARRLRELGAYSVILPGT.ASLERIAQE....NPQGIVLSGGPSSVYDAN.APRPAPGVLDLD......VPILGVCYGMQFLAHEA.GGAVQ
GUAA_DEIDV             IARRFRELGAYSVILPGT.ASLERIGQE....NPQGIVLSGGPSSVYDER.APRPAPGVLELD......VPILGVCYGMQFLAHEA.GGDVK
GUAA_DEIRA             ITRRFRELGAYSVILPGT.ASLERIQQE....NPQGIVLSGGPSSVYDEG.APRPAPGVLDLN......VPILGVCYGMQYLAHEA.GGDVK
GUAA_FUSNN             IARRVREMGVYAEVPFH.EDVDKILAR....EPKGIILSGGPASVYAEG.APSLDIKLFQNN......IPILGLCYGMQLITHLH.GGKVA
GUAA_MESFL             LARRVREANIYTEVLPFD.TSIEKIKEYP...LLKGIILSGGPSSVYLQN.AYKIDSEILNLD......IAILGVCYGMQLLTQYF.QGAVE
GUAA_SPIKU             IARRIRDLEVYCEVWPYN.TALEKIKTT....SMKGIILSGGPASVYEED.AFLIDKQLFELK......VPVLGICYGMQIISHLH.EGTVQ
GUAA_BORGA             IARRIREIGVYTKVIPYY.TPLKEIKNM....NIAGIILSGGPASVYAKD.APTLNMEIFNLK......IPVLGICYGMQLIVKLF.GGLVS
GUAA_SULDN             IARRLREDKIYCEILPYH.TKVADIKAK....NPQGIILSGGPSSVYNKD.AYEVDQGVYKMD......IPVLGICYGMQRIAADF.GGSVV
GUAA_WOLSU             IARRLREYGIYCEIVPYH.EKLEAIKAR....EPKGIILSGGPASVYEED.AFLIDKAVFELG......IPVLGICYGMQLMTHYF.GGVVA
GUAA_SULNB             IARKLRESGVYTEVVPYR.ESIEDIKAR....KPQGIILSGGPASVYAED.AYKPDEGIWDLG......LPILGICYGMQLITQHF.GGSVI
GUAA_ARCB4             IARKLREAGVYSEIVPYN.EPIGDIVAR....TPKGIILSGGPASVYAVD.AYHPDTTIFELG......LPILGICYGMQLISQHF.GGSVI
GUAA_NITSB             IARRLREVGVYCEIYPFF.EPIENIRAK....KPQGIILSGGPASVYEPD.APRVDKAIYELG......IPVLGICYGMQLITVDF.GGQVV
GUAA_CAMHC             IARRLREQGVYTEILPFN.AKISQIKEK....NPKGLILSGGPASVYAKD.AYFPNDEIFNLN......LPILGICYGMQLIAYHF.GASVT
GUAA_THESQ             ITRRIRENEVYSEVVFPD....DKVDLS....KVDAVILSGGPRSVYEED.APKLPEWFQEYK......GPVLAICYGMQLIVKEL.GGEVR
GUAA_THEMA             ITRRIRENEVYSEVVFPD....DKVDLS....KVDAVILSGGPRSVYEED.APKLPEWFQEYK......GPVLAICYGMQLIVKEL.GGEVR
GUAA_THEP1             ITRRIRENEVYSEVVFPD....DKVDLS....KVDTVILSGGPRSVYEED.APKLPEWFQEYK......GPVLAICYGMQLIVKEL.GGEVR
GUAA_PETMO             LAKRIRDLGVFSEVIQYD....DNISLS....NVKGIILSGGPDSVYNID.APDISDEILNAE......LPILGICYGMQLIAKKL.GGKVE
GUAA_AQUAE             IARRVRELGVYSEIVHWD.TPVEEIKKK....NPYGIIFSGGPASVYAEG.APLPDKRIYELG......VPILGICYGLQVITHQL.GGKVV
GUAA_THEAB             IVRRVREKGYYAELLPWD.ASKEEVQNL....NPAAIILSGGPASVFEKD.APFVPDYILELN......IPILGICYGLQSLVHKF.GGIVE
GUAA_THEM4             IVRRVREKGYYAELLPWD.AKEEEVKRL....NPTAIILSGGPSSVYEKD.APFVPEYVLHLN......IPILGICYGLQALVHKF.GGRVE
GUAA_PELUB             IARRVRELGVFSEIVSHKKIKIAQITKD....NIAGIILSGGPLNVYEND.KFKFDKKILKLG......IPILGICFGHQILSKLL.GGKVK
GUAA_MYCPE             IARRIRDLNVYSEIVDYD.ISIEEIIKK....NPKGIILSGGYDSVYGDR.SIKPNKGIWDLN......IPILGICYGFQVMMQEN.GGQVE
tr|Q7RIY7|Q7RIY7_PLAYO IVKRLNNIKIYSETKDYN.VDLKDIKNL....NIKGVILSGSPHSVNAEN.GPHIKKEVLNYFIEN..KIPIFGICYGMQEIAFHM.NGKVG
tr|Q4Z1X2|Q4Z1X2_PLABA IVKRLNNIKIYSETKDYN.VDLKDIKNL....NIKGVILSGSPHSVNAKN.GPHIKKEVLNYFIEN..KIPIFGICYGMQEIAFHM.NGQVG
tr|Q4Y2M7|Q4Y2M7_PLACH ............................................................................................
tr|B3L3R6|B3L3R6_PLAKH IVKRLNNIKIFSETRDYG.IELKEVKEL....NIKGVILSGGPHSVTEEN.APHLNKEVLEYFLEK..KIPIFAICYGMQEIAVQM.NGEVN
tr|A5K7F1|A5K7F1_PLAVS IVKRLNNIKIFSETRDYG.IDVKEVEEL....NIKGVILSGGPHSVTEEN.APHLKKEVLEYFLEK..KIPIFAICYGMQEIAVQM.NGEVK
tr|Q8IJR9|Q8IJR9_PLAF7 IVKRLNNIKIFSETKDYG.VELKDIKDM....NIKGVILSGGPYSVTEAG.SPHLKKEVFEYFLEK..KIPIFGICYGMQEIAVQM.NGEVK
```

```
                               110                                                      120        130        140        150
GUAA_ECO55    ......ASNEREFGYAQVEVVN....................................DSALVRGIEDALTADGKPLLDVWMSHGDKVTAIP
GUAA_ECO45    ......ASNEREFGYAQVEVVN....................................DSALVRGIEDALTADGKPLLDVWMSHGDKVTAIP
GUAA_ECO57    ......ASNEREFGYAQVEVVN....................................DSALVRGIEDALTADGKPLLDVWMSHGDKVTAIP
GUAA_ECO7I    ......ASNEREFGYAQVEVVN....................................DSALVRGIEDALTADGKPLLDVWMSHGDKVTAIP
GUAA_ECO5E    ......ASNEREFGYAQVEVVN....................................DSALVRGIEDALTADGKPLLDVWMSHGDKVTAIP
GUAA_ECO81    ......ASNEREFGYAQVEVVN....................................DSALVRGIEDALTADGKPLLDVWMSHGDKVTAIP
GUAA_ECO8A    ......ASNEREFGYAQVEVVN....................................DSALVRGIEDALTADGKPLLDVWMSHGDKVTAIP
GUAA_ECOHS    ......ASNEREFGYAQVEVVN....................................DSALVRGIEDALTADGKPLLDVWMSHGDKVTAIP
GUAA_ECOK1    ......ASNEREFGYAQVEVVN....................................DSALVRGIEDALTADGKPLLDVWMSHGDKVTAIP
GUAA_ECOL5    ......ASNEREFGYAQVEVVN....................................DSALVRGIEDALTADGKPLLDVWMSHGDKVTAIP
GUAA_ECOL6    ......ASNEREFGYAQVEVVN....................................DSALVRGIEDALTADGKPLLDVWMSHGDKVTAIP
GUAA_ECOLC    ......ASNEREFGYAQVEVVN....................................DSALVRGIEDALTADGKPLLDVWMSHGDKVTAIP
GUAA_ECOSE    ......ASNEREFGYAQVEVVN....................................DSALVRGIEDALTADGKPLLDVWMSHGDKVTAIP
GUAA_ECOSM    ......ASNEREFGYAQVEVVN....................................DSALVRGIEDALTADGKPLLDVWMSHGDKVTAIP
GUAA_ECOUT    ......ASNEREFGYAQVEVVN....................................DSALVRGIEDALTADGKPLLDVWMSHGDKVTAIP
GUAA_ECO24    ......ASNEREFGYAQVEVVN....................................DSALVRGIEDALTADGKPLLDVWMSHGDKVTAIP
GUAA_SHISS    ......ASNEREFGYAQVEVVN....................................DSALVRGIEDALTADGKPLLDVWMSHGDKVTAIP
GUAA_SHIF8    ......ASNEREFGYAQVEVVN....................................DSALVRGIEDALTADGKPLLDVWMSHGDKVTAIP
GUAA_SHIFL    ......ASNEREFGYAQVEVVN....................................DSALVRGIEDALTADGKPLLDVWMSHGDKVTAIP
GUAA_SHIDS    ......ASNEREFGYAQVEVVN....................................DSALVRGIEDALTADGKPLLDVWMSHGDKVTAIP
GUAA_ECOLI    ......ASNEREFGYAQVEVVN....................................DSALVRGIEDALTADGKPLLDVWMSHGDKVTAIP
GUAA_ECODH    ......ASNEREFGYAQVEVVN....................................DSALVRGIEDALTADGKPLLDVWMSHGDKVTAIP
GUAA_ECOBW    ......ASNEREFGYAQVEVVN....................................DSALVRGIEDALTADGKPLLDVWMSHGDKVTAIP
GUAA_ESCF3    ......ASNEREFGYAQVEVVN....................................DSALVRGIEDALTADGKPLLDVWMSHGDKVTAIP
GUAA_ECO27    ......ASNEREFGYAQVEVVN....................................DSALVRGIEDALTADGKPLLDVWMSHGDKVTAIP
GUAA_SHIB3    ......ASNEREFGYAQVEVVN....................................DSALVRGIEDALTADGKPLLDVWMSHGDKVTAIP
GUAA_SHIBS    ......ASNEREFGYAQVEVVN....................................DSALVRGIEDALTADGKPLLDVWMSHGDKVTAIP
GUAA_ECOLU    ......ASNEREFGYAQVEVVN....................................DSALVRGIEDALTADGKPLLDVWMSHGDKVTAIP
GUAA_SALPK    ......GSNEREFGYAQVEVLT....................................DSALVRGIEDSLTADGKPLLDVWMSHGDKVTAIP
GUAA_SALPC    ......GSNEREFGYAQVEVLT....................................DSALVRGIEDSLTADGKPLLDVWMSHGDKVTAIP
GUAA_SALPB    ......GSNEREFGYAQVEVLT....................................DSALVRGIEDSLTADGKPLLDVWMSHGDKVTAIP
GUAA_SALPA    ......GSNEREFGYAQVEVLT....................................DSALVRGIEDSLTADGKPLLDVWMSHGDKVTAIP
GUAA_SALCH    ......GSNEREFGYAQVEVLT....................................DSALVRGIEDSLTADGKPLLDVWMSHGDKVTAIP
GUAA_SALTY    ......GSNEREFGYAQVEVLT....................................DSALVRGIEDSLTADGKPLLDVWMSHGDKVTAIP
GUAA_SALSV    ......GSNEREFGYAQVEVLT....................................DSALVRGIEDSLTADGKPLLDVWMSHGDKVTAIP
GUAA_SALNS    ......GSNEREFGYAQVEVLT....................................DSALVRGIEDSLTADGKPLLDVWMSHGDKVTAIP
GUAA_SALTI    ......GSNEREFGYAQVEVLT....................................DSALVRGIEDSLTADGKPLLDVWMSHGDKVTAIP
GUAA_SALHS    ......GSNEREFGYAQVEVLT....................................DSALVRGIEDSLTADGKPLLDVWMSHGDKVTAIP
GUAA_SALG2    ......GSNEREFGYAQVEVLT....................................DSALVRGIEDSLTADGKPLLDVWMSHGDKVTAIP
GUAA_SALEP    ......GSNEREFGYAQVEVLT....................................DSALVRGIEDSLTADGKPLLDVWMSHGDKVTAIP
GUAA_SALDC    ......GSNEREFGYAQVEVLT....................................DSALVRGIEDSLTADGKPLLDVWMSHGDKVTAIP
GUAA_SALA4    ......GSNEREFGYAQVEVLT....................................DSALVRGIEDSLTADGKPLLDVWMSHGDKVTAIP
GUAA_SALAR    ......GSNEREFGYAQVEVLT....................................DSALIRGIEDSLTADGKPLLDVWMSHGDKVTAIP
GUAA_KLEP7    ......GSNEREFGYAQVEVVN....................................DSALVRGIEDSLTADGKPLLDVWMSHGDKVTAIP
GUAA_KLEP3    ......GSNEREFGYAQVEVVN....................................DSALVRGIEDSLTADGKPLLDVWMSHGDKVTAIP
GUAA_ENT38    ......ASNEREFGYAQVEVLT....................................DSALVRGIEDSLTADGKPLLDVWMSHGDKVTAIP
GUAA_CITK8    ......ASNEREFGYAQVEVRT....................................NSALIRGIEDSLTADGKPLLDVWMSHGDKVTAIP
GUAA_CROS8    ......GSNEREFGYAQVEVKT....................................DSALVRGIEDALSADGNPLLDVWMSHGDKVTAIP
GUAA_SODGM    ......GSTQREFGYAQVEVLT....................................DSLLVRDIQDDIGTGGAPLLDVWMSHGDKVTAIP
GUAA_PECCP    ......GSNEREFGYAQVEVKT....................................DSALVRDIQDALSATGAPLLDVWMSHGDKVTAIP
GUAA_PECAS    ......GSNEREFGYAQVEVKT....................................NSALVRDIQDALSATGAPLLDVWMSHGDKVTAIP
GUAA_YERPY    ......SSNQREFGYAQVEIKA....................................DSALIRDIKDAINPAGEAVLDVWMSHGDKVAEIP
GUAA_YERPS    ......SSNQREFGYAQVEIKA....................................DSALIRDIKDAINPAGEAVLDVWMSHGDKVAEIP
GUAA_YERPB    ......SSNQREFGYAQVEIKA....................................DSALIRDIKDAINPAGEAVLDVWMSHGDKVAEIP
GUAA_YERP3    ......SSNQREFGYAQVEIKA....................................DSALIRDIKDAINPAGEAVLDVWMSHGDKVAEIP
GUAA_YERPP    ......SSNQREFGYAQVEIKA....................................DSALIRDIKDAINPAGEAVLDVWMSHGDKVAEIP
GUAA_YERPN    ......SSNQREFGYAQVEIKA....................................DSALIRDIKDAINPAGEAVLDVWMSHGDKVAEIP
GUAA_YERPG    ......SSNQREFGYAQVEIKA....................................DSALIRDIKDAINPAGEAVLDVWMSHGDKVAEIP
GUAA_YERPE    ......SSNQREFGYAQVEIKA....................................DSALIRDIKDAINPAGEAVLDVWMSHGDKVAEIP
GUAA_YERPA    ......SSNQREFGYAQVEIKA....................................DSALIRDIKDAINPAGEAVLDVWMSHGDKVAEIP
GUAA_YERE8    ......GSNQREFGYAQVEIKT....................................DSALIRDIKDAINPAGEAVLDVWMSHGDKVTAIP
GUAA_SERP5    ......GSNEREFGYAQVEITT....................................ESALVRDIEDALSPAGKPLLDVWMSHGDKVTAIP
GUAA_PHOLL    ......DSTEREFGYAQVEIKA....................................ESALFRGIQDSLNEQGKPSLDVWMSHGDKVTAIP
GUAA_PROMH    ......VSGEREFGYSQVEIRE....................................TCELFRDIQDSVSEDGKPLLDVWMSHGDKVTAIP
GUAA_EDWI9    ......SSTEREFGYAKVEVTA....................................PGRLFDGIQDALSADGNAVLDVWMSHGDKVTAIP
GUAA_AERHH    ......SSTEREFGYAKVEVLGNSG................................KTSALLRNIEDAIADNGNALLDVWMSHGDKVTTIP
GUAA_AERS4    ......SSTEREFGYAQVEVLG...................................QTNALLRSIEDAIAPNGNALLDVWMSHGDKVTAIP
GUAA_TOLAT    ......SSDKREFGYAKVKLVK....................................DSELFHNIEDAITEDGRPELDVWMSHGDKVMEIP
GUAA_VIBF1    ......TSTEREFGYAAVQVTG....................................ESALFKDLEAT........QDVWMSHGDKVVEIP
GUAA_VIBFM    ......TSTEREFGYAAVQVTG....................................ESALFKDLEAT........QDVWMSHGDKVVEIP
GUAA_ALISL    ......TSTEREFGYAAVQVTG....................................ESALFKDLEAT........QDVWMSHGDKVVEIP
GUAA_VIBSL    ......TSTEREFGYAAVQVTG....................................ESALFADLETT........QDVWMSHGDKVVEIP
GUAA_VIBCM    ......TSDEREFGYAQVKISG....................................ESALFKDLDLT........QDVWMSHGDKVVEIP
GUAA_VIBCH    ......TSDEREFGYAQVKISG....................................ESALFKDLDLT........QDVWMSHGDKVVEIP
GUAA_VIBC3    ......TSDEREFGYAQVKISG....................................ESALFKDLDLT........QDVWMSHGDKVVEIP
GUAA_VIBVU    ......TSDEREFGYAAVKVSG....................................ESAIFKDLEAT........QDVWMSHGDKVVEIP
GUAA_VIBVY    ......TSDEREFGYAAVKVSG....................................ESAIFKDLEAT........QDVWMSHGDKVVEIP
GUAA_VIBPA    ......GSNEREFGYAQVKVSG....................................ESAIFKDLEAT........QDVWMSHGDKVVEIP
GUAA_VIBCB    ......GSTEREFGYAQVKVSG....................................ESALFKDLELT........QDVWMSHGDKVVEIP
GUAA_PHOPR    ......GSSMREFGYAQVEIVE....................................PTSFFKNIEDAVAEDGNGLLDVWMSHGDKVVEIP
GUAA_COLP3    ......SSSHKEFGYAAVELIA....................................QSALFNKVEDSIGDNGNALLDVWMSHGDKVSAIP
GUAA_PSYIN    K.....GEGEGEFGYAQIEVKG....................................ASELFKLIEDAVAQNGNALLDVWMSHGDKVSAIP
GUAA_PSEHT    ......SSDKKEFGYAQVEKVG....................................NCALFDAIEDHITDGGNGVLDVWMSHGDKVMDIP
GUAA_PSEA6    ......GSDMREFGYAQVEVVE....................................QMALLNNIEDHVSPNGNALLDVWMSHGDKVAAVP
GUAA_ALTMD    ......GSDKREFGYAQVEVIK....................................PSPLLDKIEDAIGDNGAALLDVWMSHGDKVAAIP
GUAA_SHESW    Q.....GVGEGEFGYAQIEMLT....................................KSALFKDIEDAVNSEGKPLLDVWMSHGDKVSAIP
GUAA_SHEPC    Q.....GVGEGEFGYAQIEMLT....................................KSALFKDIEDAVNSEGKPLLDVWMSHGDKVSAIP
GUAA_SHEB9    Q.....GVGEGEFGYAQIEMLT....................................DSLLFKGIEDAVNSEGKPLLDVWMSHGDKVSAIP
GUAA_SHEB8    Q.....GVGEGEFGYAQIEMLT....................................DSLLFKGIEDAVNSEGKPLLDVWMSHGDKVSAIP
GUAA_SHEB5    Q.....GVGEGEFGYAQIEMLT....................................DSLLFKGIEDAVNSEGKPLLDVWMSHGDKVSAIP
GUAA_SHEB2    Q.....GVGEGEFGYAQIEMLT....................................DSLLFKGIEDAVNSEGKPLLDVWMSHGDKVSAIP
GUAA_SHESM    Q.....GVGEGEFGYAQIEMLA....................................QSALFKDIEDAVSADGKSLLDVWMSHGDKVSAIP
GUAA_SHESA    Q.....GVGEGEFGYAQIEMLA....................................QSALFKDIEDAVSADGKSLLDVWMSHGDKVSAIP
GUAA_SHESR    Q.....GVGEGEFGYAQIEMLA....................................QSALFKDIEDAVNSDGKSLLDVWMSHGDKVSAIP
GUAA_SHEON    Q.....GVGEGEFGYAQIEMLA....................................ESALFKGIEDAVSSDGKSLLDVWMSHGDKVSAIP
GUAA_SHEDO    Q.....GVGEGEFGYAQVEMLL....................................ESALFKGIEDAISPAGRPLLDVWMSHGDKVSAIP
GUAA_SHEPW    Q.....GVGEGEFGYAQVEVQT....................................ESALFKAIEDAVSETGKPLLDVWMSHGDKVSEIP
GUAA_SHESH    E.....GIGEGEFGYAQIEIQE....................................PSELFKSIEDAISESGKPLLDVWMSHGDKVLDIP
GUAA_SHEWM    Q.....GVGEGEFGYAQVELQT....................................QSELFKSIEDAISDSGKPLLDVWMSHGDKVSEIP
GUAA_SHEHH    Q.....GVGEGEFGYAQVEVQT....................................ESALFKAIEDAVSETGKPLLDVWMSHGDKVSAIP
GUAA_SHEPA    Q.....GVGEGEFGYAQVEVQT....................................ESALFKAIEDAVSETGKPLLDVWMSHGDKVSAIP
GUAA_SHEFN    Q.....GVGEGEFGYAQIELQT....................................TSALFKSIEDAVSAEGKPLLDVWMSHGDKVSAIP
GUAA_SHELP    Q.....GVGEGEFGYAQVEVQA....................................ASELFKSIEDAVSDSGKALLDVWMSHGDKVSEIP
GUAA_SHEAM    Q.....GVGEREFGYAQVEVLT....................................DSALLRHIEDGISDAGKPVLDVWMSHGDKVSAIP
GUAA_IDILO    ......CSLEREFGYAQIELVE....................................QSPLFKAIEDAVSESGAPLLDVWMSHGDKVEMIP
GUAA_ACTSZ    ......GSAHREFGYAQVDLQA....................................TDSLFAQLNDDLDSA.QPKLDVWMSHGDKVTRLP
GUAA_MANSM    ......PSSHREFGYASVSLEN....................................STALFAQLNDDLNSS.LPKLDVWMSHGDKVTRLP
GUAA_ACTPJ    ......TSDHREFGYASVDLQA....................................TDALFAKLNDNLTAS.EPKLDVWMSHGDKVTRLP
GUAA_ACTP7    ......TSDHREFGYASVDLQA....................................TDALFAKLNDNLTAS.EPKLDVWMSHGDKVTRLA
GUAA_ACTP2    ......TSDHREFGYASVDLQA....................................TDALFAKLNDNLTAS.EPKLDVWMSHGDKVTRLP
GUAA_HAEPS    ......TSDHREFGYASVELKA....................................TDSLFAKLNDDLTAC.NPKLDVWMSHGDKVTRLP
GUAA_PASMU    ......TSTHREFGYASVNLKA....................................ADALFAQLNDDVTSS.QPKLDVWMSHGDKVTRLP
GUAA_HAEDU    ......ASEQREFGYAAVELQH....................................ADQLFAKLNDDLTAS.QPKLDVWMSHGDKVTRLP
GUAA_HISS1    ......TSTHREFGYAEVSLRN....................................PTALFDHLNDDATTS.QTTLDVWMSHGDKVTRLP
GUAA_HISS2    ......TSTHREFGYAEVSLQN....................................PTALFADLNDDLTAC.DPKLDVWMSHGDKVTRLP
GUAA_HAEI8    ......TSDHREFGYASVSLEN....................................STALFANLNDNSTAS.EPKLDVWMSHGDKVTRLP
GUAA_HAEIG    ......TSDHREFGYASVSLEN....................................STALFANLNDNSTAS.EPKLDVWMSHGDKVTRLP
GUAA_HAEIN    ......TSDHREFGYASVSLEN....................................STALFANLNDNLTAS.EPKLDVWMSHGDKVTRLP
GUAA_HAEIE    ......TSDHREFGYASVSLEN....................................STALFANLNDNLTAS.EPKLDVWMSHGDKVTRLP
GUAA_HAMD5    ......NTHQGEFGYAPLALKN....................................KNALIDGLEDFVNANNEPCLKVWMSHADKVTKLP
GUAA_XANC5    ......AADQREFGHAEVDVVA....................................ADALFSGLTDHA...GASRLNVWMSHGDHVSQVP
GUAA_XANAC    ......AADQREFGHAEVDVVA....................................ADALFAGLTDHA...GASRLNVWMSHGDHVSQVP
GUAA_XANCP    ......AADQREFGHAEVDVVA....................................ADALFSGLTDHA...GASRLNVWMSHGDHVSQVP
GUAA_XANCB    ......AADQREFGHAEVDVVA....................................ADALFSGLTDHA...GASRLNVWMSHGDHVSQVP
GUAA_XANC8    ......AADQREFGHAEVDVVA....................................ADALFSGLTDHA...GASRLNVWMSHGDHVSQVP
GUAA_XANOR    ......AADQREFGHAEVDVIA....................................ADALFAGLTDHA...GAPRLNVWMSHGDHVSQVP
GUAA_XANOM    ......AADQREFGHAEVDVIA....................................ADALFAGLTDHA...GAPRLNVWMSHGDHVSQVP
GUAA_XANOP    ......AADQREFGHAEVDVIA....................................ADALFAGLTDHA...GAPRLNVWMSHGDHVSQVP
GUAA_STRMK    ......AADQREFGHAEVNVIN....................................PDALFKGLSDHG...GEPKLNVWMSHGDHVSVAP
GUAA_STRM5    ......AADQREFGHAEVNVIN....................................PDALFKGLSDHG...GEPKLNVWMSHGDHVSVAP
GUAA_XYLFT    ......AADKREFGHASVQVIY....................................PDTLFSGLSDHP...SEFRLDVWMSHGDHVSRVP
GUAA_XYLF2    ......AADKREFGHASVQVIY....................................PDTLFSGLSDHP...SEFRLDVWMSHGDHVSRVP
GUAA_XYLFM    ......AADKREFGHASVQVIY....................................PDTLFSGLSDHP...SEFRLDVWMSHGDHVSRVP
GUAA_XYLFA    ......AADKREFGHASVQVIY....................................PDTLFSGLSDHP...SEFRLDVWMSHGDHVSRVP
GUAA_CELJU    ......SSAIREFGYAQIKA.HG...................................QSSLLQDIKDHVDQDGSALLDVWMSHGDKVTKMP
GUAA_TERTT    ......SSEVREFGYAQVRV.AG...................................ESALFRDIKDHVDSDGGALLDVWMSHGDKVIAMP
GUAA_HAHCH    ......GSNVREFGYAQVKIERS...................................EDGLFENIKDHVAEDGKPLLDVWMSHGDKVVALP
GUAA_MARAV    ......SSEKREFGYAQVKV.RA...................................KGPLLADITDHLTPAGDSLLDVWMSHGDKVVAMP
GUAA_PSESM    ......GSELREFGYARVDV.VG...................................KSRLLDGIEDHIDADGLFGLDVWMSHGDKVTKLP
GUAA_PSE14    ......GSELREFGYARVDV.VG...................................KSRLLDGIEDHIDADGLFGLDVWMSHGDKVTKLP
GUAA_PSEU2    ......GSELREFGYARVDV.VG...................................KSRLLDGIEDHIDADGLFGLDVWMSHGDKVTKLP
GUAA_PSEFS    ......GSELREFGYARVDV.VG...................................KSRLLDGIEDHIDADGLFGLDVWMSHGDKVTKMP
GUAA_PSEF5    ......GSELREFGYARVDV.VG...................................KSRLLDGIEDHVDADGLFGLDVWMSHGDKVTKMP
GUAA_PSEPF    ......GSELREFGYARVDV.VG...................................KSRLLDGIEDHIDADGLFGLDVWMSHGDKVTKMP
```

```
GUAA_PSEPK    ......GSDLREFGYARVDV.VG...................................KSRLLDGIEDHVDDDGVLGLDVWMSHGDKVTQMP
GUAA_PSEP1    ......GSDLREFGYARVDV.VG...................................KSRLLDGIEDHVDDDGVLGLDVWMSHGDKVTQMP
GUAA_PSEPG    ......GSDLREFGYARVDV.VG...................................KSRLLDGIEDHVDDDGVLGLDVWMSHGDKVTQMP
GUAA_PSEE4    ......GSDLREFGYARVDV.VG...................................KSRLLDGIEDHVDDDGVLGLDVWMSHGDKVTQMP
GUAA_PSEPW    ......GSDLREFGYARVDV.VG...................................KSRLLDGIEDHVDGDGVLGLDVWMSHGDKVTQMP
GUAA_PSEMY    ......GSDLREFGYARVDV.VG...................................KSQLLAGIEDHVDADGVFGLDVWMSHGDKVTRLP
GUAA_PSEAE    ......GSDLREFGYARVDV.VG...................................KARLLDGIEDHVDDDGVLGLDVWMSHGDKVTEMP
GUAA_PSEA8    ......GSDLREFGYARVDV.VG...................................KARLLDGIEDHVDDDGVLGLDVWMSHGDKVTEMP
GUAA_PSEAB    ......GSDVREFGYARVDV.VG...................................KARLLDGIEDHVDDDGVLGLDVWMSHGDKVTEMP
GUAA_MARMS    ......GSEIREFGYAQIRK.HE...................................GPALFDDIQDHIANNGVALLDVWMSHGDKVITMP
GUAA_ALCBS    ......AGEKHEFGYARVEK.AE...................................PNALLDGIVDHEE.QGKEFLDVWMSHGDRVGEMP
GUAA_THICR    ......NATEHEYGYAQVRA.HG...................................HTKLLNDIEDHVTPEGYGMLDVWMSHGDRVDKMP
GUAA_ALKEH    ......ASDHKEFGYARVRARG....................................HSRLLRDIEDHASPEGWGLLDVWMSHGDRVTGLP
GUAA_METCA    ......AVEHREFGYAQVRAHG....................................HSQLLNGIEDHTTPEGHGLLDVWMSHGDQVVGLP
GUAA_NITOC    ......VSAQREYGYAQVYVHG....................................HSRLLQNIEDHTTAGGEALLDVWMSHGDRVIGLP
GUAA_LEGPL    ......SSTLREFGYAELRLHG....................................HSQLLSNIEDRTAMDGSALLDVWMSHGDKVTELP
GUAA_LEGPA    ......SSTLREFGYAELRLHG....................................HSQLLSNIEDRTAMDGSALLDVWMSHGDKVTELP
GUAA_LEGPC    ......SSALREFGYAELRLHG....................................HSQLLSNIEDRTAVDGSALLDVWMSHGDKVTELP
GUAA_LEGPH    ......SSALREFGYAELRLHG....................................HSQLLNNIEDRILADGSALLDVWMSHGDKVTELP
GUAA_ACIAD    ......PGTVHEFGYAEVDIQQ....................................RDQLVGNLQDREN.....QLHVWMSHGDKVSRLP
GUAA_PSYA2    ......ASDIHEFGAATINIDG....................................KSTLTNGIEDAAA.....KLNVWMSHGDKVVDAP
GUAA_RUTMC    ......SANKHEYGFAKVRVRN....................................HSPLLSDISDEGHG....LLDVWMSHGIEVEQLP
GUAA_FRAP2    ......GADQSEFGKAIVNILKS...................................SENIFSNLKTEQS........VWMSHSDKVTQTG
GUAA_BURPP    ......IGHLREFGYAEVRARN....................................HTSLLEGIKDFTTPEGHGMLKVWMSHGDKVLEMP
GUAA_BURXL    ......IGHLREFGYAEVRARN....................................HTSLLEGISDFTTPEGHGMLKVWMSHGDKVLEMP
GUAA_BURP8    ......SGHLREFGYAEVRARN....................................HTSFLDGIQDFATPEGHGMLKVWMSHGDKVLEMP
GUAA_POLNS    SAE..SLGKAREFGYSEVRAHG....................................HTNLLKGIQDFSTSEGHGILKVWMSHGDSVTTLP
GUAA_POLSQ    SAE..SLGKAREFGYSEVRAHG....................................HTNLLKGIQDFSTSEGHGILKVWMSHGDSVTALP
GUAA_DECAR    TAE..AAGKSREFGYSEVRAHG....................................HTALLNDIADFYSPEGHGMLKVWMSHGDSVMELP
GUAA_HERAR    ......NGTVREFGYAEVRAHG....................................HTALLKDISDFTTPEGHGMLKVWMSHGDKVNEMP
GUAA_JANMA    ......NGKVREFGYAEVRAHG....................................HTALLKDISDFTTPEGHGMLKVWMSHGDKVNEMP
GUAA_AROAE    ......SSGKREFGYAEIRARG....................................HSELFRSIEDRTNDEGHGLLDVWMSHGDKVTALP
GUAA_AZOSB    ......SSAKREFGYAELRARG....................................HSKLFQGIEDRTNAEGHGLLDVWMSHGDKVTELP
GUAA_METFK    ......NAAKREFGYAEIRAQG....................................HSALLKGIQDKSNAEGHGLLDVWMSHGDKVTALP
GUAA_THIDA    ......SAAKREFGYAEIRARG....................................HTQLLRDIQDRVTGEGHGMLDVWMSHGDKVTALP
GUAA_NITEC    ......NAQTREFGYAELLTEP....................................CR.LFQDIKDRINSDGKPALDVWMSHGDRVNKLP
GUAA_NITEU    ......DAQTREFGYAELLTAP....................................CK.LFHGIQDRINSDGKPALDVWMSHGDRVNKLP
GUAA_NITMU    ......NSKVREFGYAELQVKN....................................LAGLLQDIHDRTDASRQKLLDVWMSHGDSVNTLP
GUAA_NEIMA    ......PGNQREFGYAQVKTID....................................SE.LTRGIQD....DTPNTLDVWMSHGDKVSKLP
GUAA_NEIMF    ......PGNQREFGYAQVKTID....................................SE.LTRGIQD....DTPNTLDVWMSHGDKVSKLP
GUAA_NEIG1    ......PGNQREFGYAQVKTID....................................SG.LTRGIQD....DAPNTLDVWMSHGDKVSKLP
GUAA_NEIG2    ......PGNQREFGYAQVKTID....................................SG.LTRGIQD....DAPNTLDVWMSHGDKVSKLP
GUAA_NEIM0    ......PGNQREFGYAQVKTID....................................SG.LTRDIYD....DAPNTLDVWMSHGDKVSKLP
GUAA_NEIMB    ......PGNQREFGYAQVKTID....................................SE.LTRGIQD....GEPNTLDVWMSHGDKVSKLP
GUAA_GEOMG    P......AGKREFGHADLLAVGT...................................PGPLFDGFFVEGKS......PVWMSHGDHVSLVP
GUAA_GEOSL    P......AGKREFGHADLLAAGT...................................PGPLFEGFFVEGKS......PVWMSHGDHVSRMP
GUAA_GEODF    P......AGKREFGHAELLAQGT...................................PGPLFDGFFLDGKS......PVWMSHGDHVSQVP
GUAA_PELPD    P......AGKREFGHAELLCSGQ...................................PGQLFDGFFVGGAS......PVWMSHGDHVSRVP
GUAA_PELCD    P......AGKREYGHATLLPCGS...................................PGPLFDEFFVDGKS......PVWMSHGDHVEQVP
GUAA_BARHE    SGH......EREFGRAFLEVQE....................................NSALFEGVWEKGSCY.....QVWMSHGDRVTALP
GUAA_BARQU    SGH......RREFGRAFLEVQE....................................KSALFDGVWEKGSCY.....QVWMSHGDHVAALP
GUAA_BARBK    AGH......EREFGRAFLEIKE....................................ESALFDGVWEKGSSQ.....QVWMSHGDRVTALP
GUAA_RHIE6    SGH......HREFGRAFLDVDK....................................DCQLFEGLWSSGSRH.....QVWMSHGDRVTALP
GUAA_RHILW    SGH......HREFGRAFLDVDK....................................DCQLFEGLWSSGSRH.....QVWMSHGDRVTALP
GUAA_RHIEC    SGH......HREFGRAFLDVDK....................................DCELFEGLWSSGSRH.....QVWMSHGDRVTALP
GUAA_RHIL3    SGH......HREFGRAFLEVDR....................................DCQLFEGLWSSGSRH.....QVWMSHGDRVTALP
GUAA_AGRRK    SGH......HREFGRAFLDVNK....................................DCALFEGLWSSGSRH.....QVWMSHGDRVTALP
GUAA_RHISN    SGH......HREFGRAFLEVEK....................................DCALFDGLWSLGSRH.....QVWMSHGDRVTALP
GUAA_RHIME    SGH......HREFGRAFLEVEK....................................DCPLFDGLWSLGSRH.....QVWMSHGDRVTALP
GUAA_SINMW    SGH......HREFGRAFLEVEK....................................DCPLFEGLWSLGSRH.....QVWMSHGDRVTALP
GUAA_AGRVS    AGH......HREFGRAFLEIEQ....................................DCKLFEGLWSVGSRH.....QVWMSHGDRVTAIP
GUAA_BRUSI    SGH......DREFGRAFLDVQE....................................DSPLFAGIWAKGTRH.....QVWMSHGDRVTSLP
GUAA_BRUAB    SGH......DREFGRAFLDVQE....................................DSPLFAGIWAKGTRH.....QVWMSHGDRVTSLP
GUAA_BRUA2    SGH......DREFGRAFLDVQE....................................DSPLFAGIWAKGTRH.....QVWMSHGDRVTSLP
GUAA_BRUA1    SGH......DREFGRAFLDVQE....................................DSPLFAGIWAKGTRH.....QVWMSHGDRVTSLP
GUAA_BRUO2    SGH......DREFGRAFLDVQE....................................DSPLFAGIWAKGTRH.....QVWMSHGDRVTSLP
GUAA_BRUSU    SGH......DREFGRAFLDVQE....................................DSPLFAGIWAKGTRH.....QVWMSHGDRVTSLP
GUAA_BRUC2    SGH......DREFGRAFLDVQE....................................DSPLFAGIWAKGTRH.....QVWMSHGDRVTSLP
GUAA_BRUMB    SGH......DREFGRAFLDVQE....................................DSPLFAGIWAKGTRH.....QVWMSHGDRVTSLP
GUAA_RHILO    SSN......HREFGRAFVEIEK....................................DSPLFEGLWATGQRH.....QVWMSHGDRVIALP
GUAA_AGRT5    GGH......AAEFGRADIDIKK....................................ASPLFEGFWEQGKSY.....PVWMSHGDRVTKLP
GUAA_BRADU    GGH......HREFGRADVEVKA....................................QSKLFEDVWSSGSKN.....QVWMSHGDRITKMP
GUAA_BRASO    GGH......HREFGRADVEVKT....................................NSLLFDGVWQPGEQH.....QVWMSHGDRITQMP
GUAA_OLICO    GGH......HREFGRAAIEITD....................................DCALFEGVWKKGEQH.....DVWMSHGDRVTKLP
GUAA_RUEST    SGHG.....TAEFGRAYVTPTE....................................ERIDMLSGWFLDQTE.....QVWMSHGDHVSEIA
GUAA_RUEPO    SGHG.....TAEFGRAYVTP.K....................................GTLDILDGWFTDGRE.....QVWMSHGDHVAEIA
GUAA_CAUCR    GGH......AGEFGRAELTIGK....................................DSPMFQGLAGVGGVE.....TVWMSHGDRVTAIP
GUAA_CAUCN    GGH......AGEFGRAELTIGK....................................DSPMFQGLAGVGGVE.....TVWMSHGDRVTAIP
GUAA_PHEZH    PGT......TREFGRAEIEIVR....................................ESPLLDGLGGAGHFE.....PVWMSHGDKVTALP
GUAA_RHORT    APD......HREFGRAFVEVTA....................................GCALFDGVWTPGERD.....QVWMSHGDRVDAIP
GUAA_GLUOX    SHE......HREFGRAYIDIAE....................................DCSLFRGVWARGKRE.....QVWMSHGDRVTQLP
GUAA_RHOBA    N......TPSREYGRAMCEFTD....................................RDSIFRGMQESE........QVWMSHGDQVSQIA
GUAA_BACFR    ......PAGTREYGRAHLTSFCK...................................DNVLFKGVREGT........QVWMSHGDTITAIP
GUAA_BACFN    ......PAGTREYGRAHLTSFCK...................................DNVLFKGVREGT........QVWMSHGDTITAIP
GUAA1_BACTN   ......PAGTREYGRAHLTSFCK...................................DNVLFKGVRENT........QVWMSHGDTITAIP
GUAA_CYTH3    ......PSSIREYGRARLNNVHT...................................SDDLFKEIVPDS........QVWMSHGDTIVEIP
GUAA_PELLD    ESSS.....KQEFGRAKILVDRTE................................GHESMLFKDIPD.SD........VWMSHGDKVTRMP
GUAA_PROVI    ESSS.....KQEFGRAKILVDRSA................................DHESLLFEGFPD.SD........VWMSHGDKVTRMP
GUAA_CHLL2    ASSA.....KQEFGRAKMLVSRDG................................EPESMLFRNIPD.SD........VWMSHGDKVVNLP
GUAA_CHLPD    AGSS.....KQEFGRAKMLVSHNE................................ESESPLFRNIPD.SD........VWMSHGDKVVRLP
GUAA_PELPB    ASSS.....KHEFGRAKIMVAQDN................................EPESPLFHDIPD.SD........VWMSHGDKVMQLP
GUAA_CHLTE    ESSS.....KQEFGRAKMLVNHD.................................ESESLLFRDIPD.SD........VWMSHGDKVTQLP
GUAA_CHLCH    ASSN.....KHEFGRSKILVEQQ..................................GDNPLFQNIPN.SD........VWMSHGDKVMQLP
GUAA_CHLPB    DSSA.....KQEFGRAEIQVERHDG...............................NPQSMLFKDIPD.SH........VWMSHGDKVVRMP
GUAA_CHLT3    AASP.....KREFGRAQMNIVRQNE...............................LAESLLFKNIPD.ST........VWMSHSDKLTCLP
GUAA_SALRD    ARAD.....RREFGRAQLQVPPDG................................QEKSLLFEGVPSGST........VWMSHSDHLTDLP
GUAA_GEMAT    VGGG.....RREYGRAEVTVDEAS.................................GMFAGFTAGEK.TT........VWMSHGDHVEQVP
GUAA_KLULA    GRGE.....KREYGPATLRVEDKS...................................CPLFANVD.HST........VWMSHHDKVHNLP
GUAA_ASHGO    GRGE.....KREYGRATLHVEDSA...................................CPLFNNVD.SST........VWMSHGDKLHALP
GUAA_YEAST    GRGD.....KREYGPATLKVIDDS...................................NSLFKGMN.DST........VWMSHGDKLHGLP
GUAA_CANGA    ARGE.....KREYGPATLNVLDKE...................................DALFKNVD.HST........VWMSHGDKLHGLP
GUAA_DEBHA    ARGD.....KREYGPATLNVEDSS...................................CSLFKGVD.HSQ........VWMSHGDKLHALP
GUAA_CANAL    ARGD.....KREYGPATLNVEDPE...................................CALFKGVD.HSQ........VWMSHGDKLHALP
GUAA_YARLI    HAGE.....KKEFGHATIHVEKPS...................................DPLFHGID.NFQ........VWMSHGDKLNALP
GUAA_PHANO    AAGE.....KREYGHANLKVESHGG................................HMDELFKDVGNELE........VWMSHGDKLSNMP
GUAA_EMENI    VAGT.....AREYGHADLKATKFG................................GHVDKLFENIEGDMT........VWMSHGDKLRNLP
GUAA_ASPOR    IAGT.....SREYGLAQLKAKKVG................................GHVDHLFDGLEDEFN........VWMSHGDKLGKLP
GUAA_ASPFU    VAGT.....AREYGHADLNAQRLDNQ..............................GHVDKLFAGLEEHVK........VWMSHGDKLVKLP
GUAA_USTMA    DPHD.....KREYGHAMVEVVKHG.V..............................PHLDALFKNWEGSVQ........VWMSHGDQLSKAP
GUAA_DEHM1    EHSE.....KREYGHALLHSSVANS.................................DLLS..DMPEPSP........VWMSHGDRIEKMP
GUAA_DEHMC    EHSD.....KREYGHALLHSNVINT.................................ELLA..DMPEPSP........VWMSHGDRIEKLP
GUAA_DESPS    EKAE.....KREFGKAEIQ.ILNDS.................................DLFAGLDVARSHQ........VWMSHGDRVEVIP
GUAA_BRAHW    ESAD.....KREYGKAEIEITNHES.................................IFKS...FGKSNI........VWMSHGDSIKSIP
GUAA_DESVV    ATSE.....TREYGPADLT.LTAPC.................................PLWDGLDLGTTSR........VWMSHGDKVKTPP
GUAA_DESVH    ATSE.....TREYGPADLT.LTAPC.................................PLWDGLDLGTTSR........VWMSHGDKVKTPP
GUAA_DESVM    ATSE.....TREYGPADLT.LLGDC.................................PLWDGIDKSATTR........VWMSHGDKVKTPP
GUAA_DESAG    SCSE.....TREYGPADLK.FCAGS.................................PLWDGLKTDESSR........VWMSHGDRVKKVP
GUAA_DESMR    AAST.....DREYGRADLQ.LLADT.................................PLFAGLPQKDGHI........VWMSHGDKVMAAP
GUAA_DESAD    VSSE.....DREYGRAEFK.GDSSC.................................TIFEGIEDIEKLT........VWMSHGDRVEAIP
GUAA_CALBD    RA......LFREYGKTHIKYDI.SS...................................PLFSGIPEHSV........CWMSHTDFVEKLP
GUAA_CALS8    TA......LFREYGKTHIKYNT.NS...................................PLFTGLPESSI........CWMSHTDFVEELP
GUAA_CARHZ    PA......EGREYGKTPLFILN.RD...................................RLFAGLNETEI........CWMSHGDFVAKAP
GUAA_CLOBL    SP......EKREYGKTDVNLDN.SS...................................LLFKDMKEKDQ........CWMSHTDYISKVP
GUAA_CLOBK    SP......EKREYGKTDVNLDN.SS...................................LLFKDMKEKDQ........CWMSHTDYISKVP
GUAA_CLOBM    SP......EKREYGKTDVNLDN.SS...................................LLFKDMKEKDQ........CWMSHTDYISKVP
GUAA_CLOBJ    SP......EKREYGKTDVNLDN.SS...................................LLFKDMKEKDQ........CWMSHTDYISKVP
GUAA_CLOB6    SP......EKREYGKTDVNLDN.SS...................................LLFKDMKEKDQ........CWMSHTDYISKVP
GUAA_CLOBH    SP......EKREYGKTDVNLDN.SS...................................LLFKDMKEKDQ........CWMSHTDYISKVP
GUAA_CLOB1    SP......EKREYGKTDVNLDN.SS...................................LLFKDMKEKDQ........CWMSHTDYISKVP
GUAA_CLONN    SP......DIREYGKTEVKIDN.KS...................................PLFDGIKEADQ........SWMSHTDYVSEIP
GUAA_CLOAB    GS......EIREYGKTDVTLDS.VC...................................ELFEGIDIENS........CWMSHTDRVAKVP
GUAA_CLOTE    SA......KVREYGKTAVNLDN.KC...................................ALFEGIDKVQE........CWMSHTDYVSEIP
GUAA_CLOPE    TA......PVREYGKTNVTLDN.SS...................................KLFAGIEADET........CWMSHTDYIAEAP
GUAA_CLOB8    TA......PVREYGKTNVKVDK.SS...................................KLFEGIETDGI........AWMSHTDYIEEAP
GUAA_ALKMQ    RA......KNREYGKTALQVDG.NS...................................KLFKEVAQDSI........CWMSHTDFIETAP
GUAA_CLOTH    KA......EQREYGKVNITFDT.SS...................................MLFEGIEKEST........CWMSHTYYVNNLP
GUAA_CLOCE    AA......NQREYGKVEICVDK.SQ...................................PLFRDVDENTI........CWMSHTYYVDTPP
GUAA_CLOBA    TA......PVSEYGKTKVDVNI.ES...................................KLFEGVSSSTI........CWMSHTDYIEKAP
GUAA_PEPD6    KGNK....QEKEYGKTAITYGK.SS....................................LFEGITTNS.........VWMSHTDLIEKVP
GUAA_FINM2    KA......KNREFGKTNAKISN.QS...................................SILKGMSDESI........VWMSHTDFVSEKP
GUAA_TREDE    KS......LKREFGKTLTKFDT.GI...................................PLFKNIKDKSS........VWMSHVDCVSRLP
GUAA_BACCR    RANH......REYGKAVLKVEN.ES...................................KLYANLPEEQV........VWMSHGDLVTGLP
GUAA_BACC4    RANH......REYGKAVLKVEN.ES...................................KLYANLPEEQV........VWMSHGDLVTGLP
GUAA_BACC2    RANH......REYGKAVLKVEN.ES...................................KLYANLPEEQV........VWMSHGDLVTGLP
```

```
GUAA_BACWK                RANH......REYGKAVLKVEN.ES...................................KLYANLPEEQV........VWMSHGDLVTGLP
GUAA_BACCZ                RANH......REYGKAVLKVEN.ES...................................KLYANLPEEQV........VWMSHGDLVTGLP
GUAA_BACC3                RANH......REYGKAVLKVEN.ES...................................KLYANLPEEQV........VWMSHGDLVTGLP
GUAA_BACAH                RANH......REYGKAVLKVEN.ES...................................KLYANLPEEQV........VWMSHGDLVTGLP
GUAA_BACAC                RANH......REYGKAVLKVEN.ES...................................KLYANLPEEQV........VWMSHGDLVTGLP
GUAA_BACAA                RANH......REYGKAVLKVEN.ES...................................KLYANLPEEQV........VWMSHGDLVTGLP
GUAA_BACHK                RANH......REYGKAVLKVEN.ES...................................KLYANLPEEQV........VWMSHGDLVTGLP
GUAA_BACC0                RANH......REYGKAVLKVEN.ES...................................KLYANLPEEQV........VWMSHGDLVTGLP
GUAA_BACAN                RANH......REYGKAVLKVEN.ES...................................KLYANLPEEQV........VWMSHGDLVTGLP
GUAA_BACC1                RANH......REYGKAVLKVEN.ES...................................KLYANLPEEQV........VWMSHGDLVTGLP
GUAA_BACCN                RANH......REYGKAVLKVEN.ES...................................KLYTNLPEEQV........VWMSHGDLVTGLP
GUAA_BACA2                AASQ......REYGKANIQIQG.TP...................................DLFKDLPEEQV........VWMSHGDLVVEVP
GUAA_BACSU                AASQ......REYGKANIRIEG.TP...................................DLFRDLPNEQV........VWMSHGDLVVEVP
GUAA_BACLD                AASQ......REYGKANIHIEG.EP...................................DLFKDLPNEQV........VWMSHGDLVVEVP
GUAA_BACP2                AASQ......REYGKADIHING.TP...................................ALFKDLPTDQV........VWMSHGDLVVEVP
GUAA_BACSK                KASH......REYGKALLNVEN.QS...................................PLFKGLPIEQT........VWMSHGDKIVAPP
GUAA_BACHD                AAEH......REYGKATITVEN.QS...................................KLFQGLPVEQT........VWMSHGDLIVAPP
GUAA_GEOKA                KATH......REYGKALIQVKN.DS...................................LLFHGLPDEQV........VWMSHGDLVTAPP
GUAA_LISMF                RAKD......REYGKADIHVEK.PN...................................RLFAGLPTDQV........VWMSHGDLVVEEP
GUAA_LISMO                RAKD......REYGKADIHVEK.PN...................................RLFAGLPTDQV........VWMSHGDLVVEEP
GUAA_LISW6                RAKD......REYGKADIHVEN.PS...................................RLFAGLPTDQV........VWMSHGDLVVEEP
GUAA_LISIN                RAKD......REYGKADIHVEN.PS...................................RLFAGLPTDQV........VWMSHGDLVVEEP
GUAA_EXIS2                RAGH......REYGKATLTLND.PS...................................PMYANLPLEQT........VWMSHSDLVTSVP
GUAA_STAES                RANE......REYGKATINAKS.DE....................................LFFGLPSEQT........VWMSHSDKVIEIP
GUAA_STAEQ                RANE......REYGKATINAKS.DE....................................LFFGLPSEQT........VWMSHSDKVIEIP
GUAA_MACCJ                RANE......REYGKAVIKAET.HS....................................LFTKLPEEQT........VWMSHSDKVINLP
GUAA_OCEIH                RSKN......REYGKALIELDG.EP...................................VLFKDTPKGQT........VWMSHGDKVTAAP
GUAA_LACRJ                KADN......SEYGRADIEVLDDEA...................................VLLKGLPKKQY........VWMSHGDLVTQAP
GUAA_LACRD                KADN......SEYGRADIEVLDDEA...................................VLLKGLPKKQY........VWMSHGDLVTQAP
GUAA_LACJO                KADN......SEYGRADIEVIDPNA...................................VLFEGLPREQY........VWMSHGDLVTKAP
GUAA_LACGA                KADN......SEYGRADIEVTDPNA...................................VLFEGLPKEQY........VWMSHGDLVTKAP
GUAA_LACAC                RAEN......KEYGRANITVEDPDS...................................ALFKGLPTKQY........VWMSHGDLVTQAP
GUAA_LACDB                KAGE......AEYGSAHIEVKEADS...................................PLYKGLPEKQV........VWMSHGDLVTKVP
GUAA_LACDA                KAGE......AEYGSAHIEVKEADS...................................PLYKGLPEKQV........VWMSHGDLVTKVP
GUAA_LACCB                PADN......REYGKADIEVTDDSA...................................KLFRDLPKDQT........VWMSHGDLVTRVP
GUAA_LACC3                PADN......REYGKADIEVTDDSA...................................KLFRDLPKDQT........VWMSHGDLVTRVP
GUAA_LACRH                PADN......REYGKADIEVTDASA...................................KLFHDLPKDQT........VWMSHGDLVTRVP
GUAA_LACS1                SADN......KEYGKAIITVKNQEN...................................VMFKDLPETQT........VWMSHGDLVTQVP
GUAA_LACLM                AAGE......REYGVAPLQLTEK.S...................................ALFAGTPEVQD........VLMSHGDRVTAIP
GUAA_LACLS                AAGE......REYGVAPLQLTEK.S...................................ALFAGTPEVQD........VLMSHGDRVTAIP
GUAA_LACLA                AAGE......REYGVAPLQLTGK.S...................................ALFAGTPEVQD........VLMSHGDRVTAIP
GUAA_STRZJ                PAGDA...GNREYGQSTLTHTP..S...................................ALFESTPDEQT........VLMSHGDAVTEIP
GUAA_STRPJ                PAGDA...GNREYGQSTLTHTP..S...................................ALFESTPDEQT........VLMSHGDAVTEIP
GUAA_STRZT                PAGDA...GNREYGQSTLTHTP..S...................................ALFESTPDEQT........VLMSHGDAVTEIP
GUAA_STRR6                PAGDA...GNREYGQSTLTHTP..S...................................ALFESTPDEQT........VLMSHGDAVTEIP
GUAA_STRPN                PAGDA...GNREYGQSTLTHTP..S...................................ALFESTPDEQT........VLMSHGDAVTEIP
GUAA_STRPI                PAGDA...GNREYGQSTLTHTP..S...................................ALFESTPDEQT........VLMSHGDAVTEIP
GUAA_STRP7                PAGDA...GNREYGQSTLTHTP..S...................................ALFESTPDEQT........VLMSHGDAVTEIP
GUAA_STRP4                PAGDA...GNREYGQSTLTHTP..S...................................ALFESTPDEQT........VLMSHGDAVTEIP
GUAA_STRP2                PAGDA...GNREYGQSTLTHTP..S...................................ALFESTPDEQT........VLMSHGDAVTEIP
GUAA_STRZP                PAGDA...GNREYGQSTLTHTP..S...................................ALFESTPDEQT........VLMSHGDAVTEIP
GUAA_STRPS                PAGDA...GNREYGQSTLTHTP..S...................................ALFESTPDEQT........VLMSHGDAVTEIP
GUAA_SYMTH                RGQ......QGEYGRATLRLADAEH...................................PLLRGLGRESL........VWMSHFDSVTRVP
GUAA_MOOTA                GAS......GREYGKTQLEITAAD....................................PLFAGLPESIQ........CWMSHGDYISAPP
GUAA_HELMI                RAE......SREYGKASLTITASE....................................GPFAGMEGDVQ........CWMSHGDKVEVLP
GUAA_HALOH                KAR......HGEYGKARLHITEKD....................................KLFEGIS.DSQ........VWMSHWDRVKKVP
GUAA_THETN                PAP......VREYGKTEVVINNTI....................................PLFKGIERDTI........VWMSHTDQIELPP
GUAA_NATTJ                GST......KREYGKALLEIENPN....................................LLTEGVSSDLL........VWMSHGDSVEQVP
GUAA_MYCTU                HTG......TREYGRTELKVLGGK.....................................LHSDLPEVQP........VWMSHGDAVTAAP
GUAA_MYCTA                HTG......TREYGRTELKVLGGK.....................................LHSDLPEVQP........VWMSHGDAVTAAP
GUAA_MYCBT                HTG......TREYGRTELKVLGGK.....................................LHSDLPEVQP........VWMSHGDAVTAAP
GUAA_MYCBP                HTG......TREYGRTELKVLGGK.....................................LHSDLPEVQP........VWMSHGDAVTAAP
GUAA_MYCBO                HTG......TREYGRTELKVLGGK.....................................LHSDLPEVQP........VWMSHGDAVTAAP
GUAA_MYCTO                HTG......TREYGRTELKVLGGK.....................................LHSDLPEVQP........VWMSHGDAVTAAP
GUAA_MYCMM                HTG......TSEYGRTELKVLGGD.....................................LHSDLPDIQP........VWMSHGDAVTAAP
GUAA_MYCPA                HTG......TSEYGRTELKVLGGE.....................................LHSGLPGVQP........VWMSHGDAVTAAP
GUAA_NOSS1                KAD......RGEYGKASLHIDDPT....................................DLLTNVEDGTT........MWMSHGDSVTKMP
GUAA_ANAVT                KAD......RGEYGKASLHIDDPT....................................DLLTNVEDGTT........MWMSHGDSVTKMP
GUAA_NOSP7                KAD......RGEYGKASLYIDDPT....................................DLLTNVEDGTT........MWMSHGDSVTQMP
GUAA_THEEB                RAI......AGEYGKAALFIDDPT....................................DLLTNVEQETI........MWMSHGDSVTELP
GUAA_CYAA5                KAL......RGEYGKASLRIDDPT....................................DLLTNVEDGST........MWMSHGDSCTRLP
GUAA_CYAP8                RAK......RGEYGKASLFIDDPT....................................DLLTNVEDGST........MWMSHGDSCTQLP
GUAA_CYAP7                RAK......RAEYGKASLHIDDPT....................................DLLTNVEDNSI........AWMSHGDSCIELP
GUAA_SYNY3                RAK......RGEYGKASLHIDDPT....................................DLLTNVENDST........MWMSHGDSCVDLP
GUAA_SYNP2                RAK......HAEYGKASIFIDDPT....................................DLLTNVEEGST........MWMSHGDSCVHLP
GUAA_SYNP6                RAE......RGEYGKAALVIDDPT....................................DLLTNVEPGST........MWMSHGDSVKTMP
GUAA_GLOVI                RAE......RAEYGKASLFINDPT....................................DLFTNVEDGTT........MWMSHADSVLRMP
GUAA_PROMP                PAIN.....KAEYGRAPINIDCES....................................DLLSNVQDKSI........MWMSHGDSINNLP
GUAA_PROM5                PANN.....RSEYGRAPIKIDFES....................................DLLSSVKDKSI........MWMSHGDSINHLP
GUAA_PROMT                PAAG.....KAEYGKAPLEVDDPT....................................ALLTNVISGST........MWMSHGDSVQKLP
GUAA_PROM1                PATG.....KAEYGKAPLEVDDPT....................................ALLTNVISGST........MWMSHGDSVQKLP
GUAA_THERP                PAQ......RREYGPAVVERVADH....................................PIFAGLPARFD........VWMSHGDRIDALP
GUAA_DEIGD                RAGK......REYGKADLTRYG..G...................................QLFAGIQGEFV........AWMSHSDSVTQLP
GUAA_DEIDV                RAGK......REYGKADLTRYG..G...................................QLFEGIQGEFV........AWMSHSDSVTQLP
GUAA_DEIRA                RAGK......REYGKADLTEYG..G...................................RLFEGIQGEFV........AWMSHSDSVTQLP
GUAA_FUSNN                RAD......KQEFGKAELELDDKNHI...................................LYKNIPNKTT........VWMSHGDHVTEMA
GUAA_MESFL                LAD......EQEFGKAIITIDDKKNP...................................LFKNVTTESQ........VWMSHADHVTELP
GUAA_SPIKU                RAT......KQEFGFSELIIDNQED....................................LFANIPVKSQ........VWMSHADYIEGMP
GUAA_BORGA                KDY......KQEYGSSEIFLKNEKSL...................................LFSELPNKFQ........IIMSHGDSIEKIP
GUAA_SULDN                RAS......HHEYGKAELKILNLETN................................PSPLFKDCDDERI........VWMSHSDKVDTLP
GUAA_WOLSU                KAE......HHEYGKAEVIFDALEGS................................ESKLFEGCHSGKV........VWMSHGDKVEKLP
GUAA_SULNB                AAD......HHEYGKAKLHIENENS...................................PIFKDVAQDSI........VWMSHGDRAERLP
GUAA_ARCB4                PAS......HHEYGKAKLQFEVEN....................................PIFKDTQDGQI........VWMSHGDRVENIP
GUAA_NITSB                RAL......EHEYGKAKLYFDPIHGK................................VCPLFEGTKDGQI........VWMSHGDKVTELP
GUAA_CAMHC                PTN......KKEYGKSNLKFVSEN....................................ALLKDTKDGQI........VWMSHSDKVENLP
GUAA_THESQ                .......RGRGEYGRTLVELS.....................................RDPIFEGIPEKVH........VWMSHGDEVVRLP
GUAA_THEMA                .......RGRGEYGRTLVELS.....................................RDPIFEGIPEKVH........VWMSHGDEVVRLP
GUAA_THEP1                .......RGRGEYGRTLVELS.....................................RDPIFEGIPEKVH........VWMSHGDEVVRLP
GUAA_PETMO                Q......RGIAEYGKTKINITD....................................QSLLFKKIPSTFN........VWMSHKDMVTKVP
GUAA_AQUAE                R......SEKQEYGRARLRIIK....................................EDVIFEGIPKESD........VWMSHADKVVELP
GUAA_THEAB                K......SPKREFGHAVLKVK.....................................DDPLFEGLPKEFD........VWMSHSDRVEKLP
GUAA_THEM4                K......SQKREFGHAILEVE.....................................EDPLFEGLPKKFD........VWMSHSDRVEKLP
GUAA_PELUB                KSK......HREFGLATINKVS....................................NSTLTKNFFNKNNRS.....DVWMSHADQVSKMP
GUAA_MYCPE                NDKD.....SEEYGSTDIRLTT.....................................NELFENIELDNN........CWMSHSDSVIFLP
tr|Q7RIY7|Q7RIY7_PLAYO    KSK......NSEHGSTEVTLISNDYK..............NNESYKNYKLLE...KDSNCLLFDGIKNTNNMN......VWMNHTEEVIEIP
tr|Q4Z1X2|Q4Z1X2_PLABA    KSK......NSEHGSTEVTLISNDYK..............DNELYKNYKLLE...KDSNCLLFDDIKNTNNMN......VWMNHTEEVIEIP
tr|Q4Y2M7|Q4Y2M7_PLACH    ............................................................................................
tr|B3L3R6|B3L3R6_PLAKH    KSK......NSEYGCTDVNIITSKNK..............SEEKYKNYNLVDGDGKGKKCLLFDGIKDPEKST......VWMNHTDEVTKIP
tr|A5K7F1|A5K7F1_PLAVS    KSK......NSEYGCTDVNIITSKNG..............GEEKYKNYKLVDGDSKGSKCILFDGIKNAEKST......VWMNHTDEVTKIP
tr|Q8IJR9|Q8IJR9_PLAF7    KSK......TSEYGCTDVNILRNDNINNITYCRNFGDSSSAMDLYSNYKLMN.....ETCCLFENIKS.DITT......VWMNHNDEVTKIP
```

```
                       160       170          180       190        200       210       220        230
GUAA_ECO55      SDFVTVASTESCPFAIMANEEKR....FYGVQFHPEVTHTRQGMRMLERFVRDICQCEALWTPAKIIDDAVARIREQVG.DDKVILGLSGGV
GUAA_ECO45      SDFVTVASTESCPFAIMANEEKR....FYGVQFHPEVTHTRQGMRMLERFVRDICQCEALWTPAKIIDDAVARIREQVG.DDKVILGLSGGV
GUAA_ECO57      SDFVTVASTESCPFAIMANEEKR....FYGVQFHPEVTHTRQGMRMLERFVRDICQCEALWTPAKIIDDAVARIREQVG.DDKVILGLSGGV
GUAA_ECO7I      SDFVTVASTESCPFAIMANEEKR....FYGVQFHPEVTHTRQGMRMLERFVRDICQCEALWTPAKIIDDAVARIREQVG.DDKVILGLSGGV
GUAA_ECO5E      SDFVTVASTESCPFAIMANEEKR....FYGVQFHPEVTHTRQGMRMLERFVRDICQCEALWTPAKIIDDAVARIREQVG.DDKVILGLSGGV
GUAA_ECO81      SDFVTVASTESCPFAIMANEEKR....FYGVQFHPEVTHTRQGMRMLERFVRDICQCEALWTPAKIIDDAVARIREQVG.DDKVILGLSGGV
GUAA_ECO8A      SDFVTVASTESCPFAIMANEEKR....FYGVQFHPEVTHTRQGMRMLERFVRDICQCEALWTPAKIIDDAVARIREQVG.DDKVILGLSGGV
GUAA_ECOHS      SDFVTVASTESCPFAIMANEEKR....FYGVQFHPEVTHTRQGMRMLERFVRDICQCEALWTPAKIIDDAVARIREQVG.DDKVILGLSGGV
GUAA_ECOK1      SDFVTVASTESCPFAIMANEEKR....FYGVQFHPEVTHTRQGMRMLERFVRDICQCEALWTPAKIIDDAVARIREQVG.DDKVILGLSGGV
GUAA_ECOL5      SDFVTVASTESCPFAIMANEEKR....FYGVQFHPEVTHTRQGMRMLERFVRDICQCEALWTPAKIIDDAVARIREQVG.DDKVILGLSGGV
GUAA_ECOL6      SDFVTVASTESCPFAIMANEEKR....FYGVQFHPEVTHTRQGMRMLERFVRDICQCEALWTPAKIIDDAVARIREQVG.DDKVILGLSGGV
GUAA_ECOLC      SDFVTVASTESCPFAIMANEEKR....FYGVQFHPEVTHTRQGMRMLERFVRDICQCEALWTPAKIIDDAVARIREQVG.DDKVILGLSGGV
GUAA_ECOSE      SDFVTVASTESCPFAIMANEEKR....FYGVQFHPEVTHTRQGMRMLERFVRDICQCEALWTPAKIIDDAVARIREQVG.DDKVILGLSGGV
GUAA_ECOSM      SDFVTVASTESCPFAIMANEEKR....FYGVQFHPEVTHTRQGMRMLERFVRDICQCEALWTPAKIIDDAVARIREQVG.DDKVILGLSGGV
GUAA_ECOUT      SDFVTVASTESCPFAIMANEEKR....FYGVQFHPEVTHTRQGMRMLERFVRDICQCEALWTPAKIIDDAVARIREQVG.DDKVILGLSGGV
GUAA_ECO24      SDFVTVASTESCPFAIMANEEKR....FYGVQFHPEVTHTRQGMRMLERFVRDICQCEALWTPAKIIDDAVARIREQVG.DDKVILGLSGGV
GUAA_SHISS      SDFVTVASTESCPFAIMANEEKR....FYGVQFHPEVTHTRQGMRMLERFVRDICQCEALWTPAKIIDDAVARIREQVG.DDKVILGLSGGV
GUAA_SHIF8      SDFVTVASTESCPFAIMANEEKR....FYGVQFHPEVTHTRQGMRMLERFVRDICQCEALWTPAKIIDDAVARIREQVG.DDKVILGLSGGV
GUAA_SHIFL      SDFVTVASTESCPFAIMANEEKR....FYGVQFHPEVTHTRQGMRMLERFVRDICQCEALWTPAKIIDDAVARIREQVG.DDKVILGLSGGV
GUAA_SHIDS      SDFITVASTESCPFAIMANEEKR....FYGVQFHPEVTHTRQGMRMLERFVRDICQCEALWTPAKIIDDAVARIREQVG.DDKVILGLSGGV
GUAA_ECOLI      SDFITVASTESCPFAIMANEEKR....FYGVQFHPEVTHTRQGMRMLERFVRDICQCEALWTPAKIIDDAVARIREQVG.DDKVILGLSGGV
GUAA_ECODH      SDFITVASTESCPFAIMANEEKR....FYGVQFHPEVTHTRQGMRMLERFVRDICQCEALWTPAKIIDDAVARIREQVG.DDKVILGLSGGV
GUAA_ECOBW      SDFITVASTESCPFAIMANEEKR....FYGVQFHPEVTHTRQGMRMLERFVRDICQCEALWTPAKIIDDAVARIREQVG.DDKVILGLSGGV
GUAA_ESCF3      SDFITVASTESCPFAIMANEEKR....FYGVQFHPEVTHTRQGMRMLERFVRDICQCEALWTPAKIIDDAVARIREQVG.DDKVILGLSGGV
GUAA_ECO27      SDFVTVASTESCPFAIMANEEKR....FYGVQFHPEVTHTRQGMRMLERFVRDICQCEALWTPAKIIDDAVARIREQVG.DDKVILGLSGGV
GUAA_SHIB3      SDFVTVASTESCPFAIMANEEKR....FYGVQFHPEVTHTRQGMHMLERFVRDICQCEALWTPAKIIDDAVARIREQVG.DDKVILGLSGGV
GUAA_SHIBS      SDFVTVASTESCPFAIMANEEKR....FYGVQFHPEVTHTRQGMHMLERFVRDICQCEALWTPAKIIDDAVARIREQVG.DDKVILGLSGGV
GUAA_ECOLU      SDFVTVASTESCPFAIMANEEKR....FYGVQFHPEVTHTRQGMRMLERFVRDICQCEALWTPAKIIDDAVARIREQVG.DDKVILGLSGGV
GUAA_SALPK      SDFVTVASTESCPFAIMANEEKR....FYGVQFHPEVTHTRQGMRMLERFVRDICQCEALWTPAKIIDDAVARIREQVG.DDKVILGLSGGV
GUAA_SALPC      SDFVTVASTESCPFAIMANEEKR....FYGVQFHPEVTHTRQGMRMLERFVRDICQCEALWTPAKIIDDAVARIREQVG.DDKVILGLSGGV
GUAA_SALPB      SDFVTVASTESCPFAIMANEEKR....FYGVQFHPEVTHTRQGMRMLERFVRDICQCEALWTPAKIIDDAVARIREQVG.DDKVILGLSGGV
GUAA_SALPA      SDFVTVASTESCPFAIMANEEKR....FYGVQFHPEVTHTRQGMRMLERFVRDICQCEALWTPAKIIDDAVARIREQVG.DDKVILGLSGGV
GUAA_SALCH      SDFVTVASTESCPFAIMANEEKR....FYGVQFHPEVTHTRQGMRMLERFVRDICQCEALWTPAKIIDDAVARIREQVG.DDKVILGLSGGV
GUAA_SALTY      SDFVTVASTESCPFAIMANEEKR....FYGVQFHPEVTHTRQGMRMLERFVRDICQCEALWTPAKIIDDAVARIREQVG.DDKVILGLSGGV
GUAA_SALSV      SDFVTVASTESCPFAIMANEEKR....FYGVQFHPEVTHTRQGMRMLERFVRDICQCEALWTPAKIIDDAVARIREQVG.DDKVILGLSGGV
GUAA_SALNS      SDFVAVASTESCPFAIMANEEKR....FYGVQFHPEVTHTRQGMRMLERFVRDICQCEALWTPAKIIDDAVARIREQVG.DDKVILGLSGGV
GUAA_SALTI      SDFVTVASTESCPFAIMANEEKR....FYGVQFHPEVTHTRQGMRMLERFVRDICQCEALWTPAKIIDDAVARIREQVG.DDKVILGLSGGV
GUAA_SALHS      SDFVTVASTESCPFAIMANEEKR....FYGVQFHPEVTHTRQGMRMLERFVRDICQCEALWTPAKIIDDAVARIREQVG.DDKVILGLSGGV
GUAA_SALG2      SDFVTVASTESCPFAIMANEEKR....FYGVQFHPEVTHTRQGMRMLERFVRDICQCEALWTPAKIIDDAVARIREQVG.DDKVILGLSGGV
GUAA_SALEP      SDFVTVASTESCPFAIMANEEKR....FYGVQFHPEVTHTRQGMRMLERFVRDICQCEALWTPAKIIDDAVARIREQVG.DDKVILGLSGGV
GUAA_SALDC      SDFVTVASTESCPFAIMANEEKR....FYGVQFHPEVTHTRQGMRMLERFVRDICQCEALWTPAKIIDDAVARIREQVG.DDKVILGLSGGV
GUAA_SALA4      SDFVTVASTESCPFAIMANEEKR....FYGVQFHPEVTHTRQGMRMLERFVRDICQCEALWTPAKIIDDAVARIREQVG.DDKVILGLSGGV
GUAA_SALAR      SDFVTVASTESCPFAIMANEEKR....FYGVQFHPEVTHTRQGMRMLERFVRDICQCEALWTPAKIIDDAVARIREQVG.DDKVILGLSGGV
GUAA_KLEP7      ADFVTVASTDNCPFAIMANEEKR....FYGVQFHPEVTHTRQGMRMLERFVRDICQCEALWTPAKIIDDAVERIRQQVG.DDKVILGLSGGV
GUAA_KLEP3      SDFVTVASTDNCPFAIMANEEKR....FYGVQFHPEVTHTRQGMRMLERFVRDICQCEALWTPAKIIDDAVERIRQQVG.DDKVILGLSGGV
GUAA_ENT38      SDFVTVASTETCPFAIMANEEKR....FYGVQFHPEVTHTRQGMRMLDRFVRDICQCEALWTPAKIIDDAIARIRQQVG.DDKVILGLSGGV
GUAA_CITK8      SDFVTVASTETCPFAIMANEEKR....FYGVQFHPEVTHTRQGMRMLERFVRDICQCEALWTPAKIIDDAVNRIREQVG.DDKVILGLSGGV
GUAA_CROS8      SDFVTVASTETCPFAIMANEEKR....FYGVQFHPEVTHTRQGLRMLERFVRDICECEALWTPAKIIDDAVERIRQQVG.NDRVILGLSGGV
GUAA_SODGM      ADFVTVASTETCHFAIMANEEKR....FYGVQFHPEVTHTRQGQRMLERFVLDICRCTPLWTPAKIIEDAVARIREQVG.NDRVILGLSGGV
GUAA_PECCP      EGFETVASTDTCPFAIMANEEKR....FYGVQFHPEVTHTRQGQRMLERFVRDICQCEALWTPAKIIDDAVTRIREQVG.NDQVILGLSGGV
GUAA_PECAS      EGFETVASTDTCPFAIMANEEKR....FYGVQFHPEVTHTRQGQRMLERFVLDICQCEALWTPAKIIDDAVNRIREQVG.NDSVILGLSGGV
GUAA_YERPY      ADFVTVASTDTCPFAIMANEEKR....FYGVQFHPEVTHTKQGLRLLERFVLGICGCEALWTSATIIEDAIVRLREQIG.DDHVILGLSGGV
GUAA_YERPS      ADFVTVASTDTCPFAIMANEEKR....FYGVQFHPEVTHTKQGLRLLERFVLGICGCEALWTSATIIEDAIVRLREQIG.DDHVILGLSGGV
GUAA_YERPB      ADFVTVASTDTCPFAIMANEEKR....FYGVQFHPEVTHTKQGLRLLERFVLGICGCEALWTSATIIEDAIVRLREQIG.DDHVILGLSGGV
GUAA_YERP3      ADFVTVASTDTCPFAIMANEEKR....FYGVQFHPEVTHTKQGLRLLERFVLGICGCEALWTSATIIEDAIVRLREQIG.DDHVILGLSGGV
GUAA_YERPP      ADFVTVASTDTCPFAIMANEEKR....FYGVQFHPEVTHTKQGLRLLERFVLGICGCEALWTSATIIEDAIVRLREQIG.DDHVILGLSGGV
GUAA_YERPN      ADFVTVASTDTCPFAIMANEEKR....FYGVQFHPEVTHTKQGLRLLERFVLGICGCEALWTSATIIEDAIVRLREQIG.DDHVILGLSGGV
GUAA_YERPG      ADFVTVASTDTCPFAIMANEEKR....FYGVQFHPEVTHTKQGLRLLERFVLGICGCEALWTSATIIEDAIVRLREQIG.DDHVILGLSGGV
GUAA_YERPE      ADFVTVASTDTCPFAIMANEEKR....FYGVQFHPEVTHTKQGLRLLERFVLGICGCEALWTSATIIEDAIVRLREQIG.DDHVILGLSGGV
GUAA_YERPA      ADFVTVASTDTCPFAIMANEEKR....FYGVQFHPEVTHTKQGLRLLERFVLGICGCEALWTSATIIEDAIVRLREQIG.DDHVILGLSGGV
GUAA_YERE8      SDFVTVASTDTCPFAIMANEEKR....FYGVQFHPEVTHTKQGQRLLERFVLDICACEALWTPATIIEDAIVRLREQIG.EDHVILGLSGGV
GUAA_SERP5      ADFVTVASTDTCPFAIMANEEKR....FYGVQFHPEVTHTRQGQRMLERFVLDICQCEALWTPATIIEDAVERIRQQVG.EDHVILGLSGGV
GUAA_PHOLL      EDFITIASTDTCPFAIIANEEKR....FYGVQFHPEVTHTHQGQRILERFVLDVCQCEALWTPASIIEDTVVRLREQVG.EDHVILGLSGGV
GUAA_PROMH      ADFVTIASTETCPFAIMANEEKR....FYGVQFHPEVTHTHQGLAILKRFVLDICGCDALWTSAAIIEDTVARLKQQIG.DDHVILALSGGV
GUAA_EDWI9      SDFTTVASTETCPYAIMANEAKR....FYGVQFHPEVTHTHQGQHMLQRFVLEICGCAALWTPATIIEDAVVRMREQIG.DDEVILGLSGGV
GUAA_AERHH      ADFTTIAQTATCPHAAMANEAKR....FYGVQFHPEVTHTRQGARMLEHFVKDICGCECLWTPATIIDDAVARIREQVG.DDEVILGLSGGV
GUAA_AERS4      ADFTTIAQTATCPHAAMACESKH....FYGVQFHPEVTHTRQGARMLEHFVKDICGCECLWTPATIIDDAVARIREQVG.DDEVILGLSGGV
GUAA_TOLAT      VDFVTIAVTPTCPHAAMANEAKR....FYGVQFHPEVTHTVQGARMLERFVKEICHCEALWTPATIIEDAVARIREQVG.TDEVILGLSGGV
GUAA_VIBF1      SDFVKIAETETCPYAAMANEEKK....YYGVQFHPEVTHTKNGMKMLENFVLNVCGCEGLWTSASIIEDAVARIKEQVG.DDEVILGLSGGV
GUAA_VIBFM      SDFVKIAETETCPYAVMANEEKK....YYGVQFHPEVTHTKNGMKMLENFVLNVCGCEGLWTSASIIEDAVARIKEQVG.DDEVILGLSGGV
GUAA_ALISL      SDFVKIAETETCPYAAMANEEKK....YYGVQFHPEVTHTKNGLKMLENFVLNVCGCEGLWTSASIIEDAIARIKEQVG.DDEVILGLSGGV
GUAA_VIBSL      ADFTKIAETDTCPYAAMANEEKK....YYGVQFHPEVTHTKNGLKMLENFVLNACGCEGLWTSASIIEDAVARIKEQVG.DDEVILGLSGGV
GUAA_VIBCM      ADFVKIGETDTCPYAAMANEEKK....YYGVQFHPEVTHTKNGLQMLENFVLGVCGCERLWTSESIIEDAVARIKEQVG.NDEVILGLSGGV
GUAA_VIBCH      ADFVKIGETDTCPYAAMANEEKK....YYGVQFHPEVTHTKNGLQMLENFVLGVCGCERLWTSESIIEDAVARIKEQVG.NDEVILGLSGGV
GUAA_VIBC3      ADFVKIGETDTCPYAAMANEEKK....YYGVQFHPEVTHTKNGLQMLENFVLGVCGCERLWTSESIIEDAVARIKEQVG.NDEVILGLSGGV
GUAA_VIBVU      AGFTKVGETDTCPYAAMANEEKK....YYGVQFHPEVTHTKNGLQMLENFVLGICGCERLWTSESIIEDAVARIKEQVG.DDEVILGLSGGV
GUAA_VIBVY      AGFTKVGETDTCPYAAMANEEKK....YYGVQFHPEVTHTKNGLQMLENFVLGVCGCERLWTSESIIEDAVARIKEQVG.DDEVILGLSGGV
GUAA_VIBPA      ADFVKVGETDTCPYAAMANEEKK....YYGVQFHPEVTHTKGGLQMLENFVLGVCGCERLWTSESIIEDAVARIKEQVG.DDEVILGLSGGV
GUAA_VIBCB      ADFVKVGETDTCPYAAMANEEKK....YYGVQFHPEVTHTKGGLQMLENFVLGVCGCERLWTSESIIEDAVARIKEQVG.DDEVILGLSGGV
GUAA_PHOPR      SDFVKVAQTDTCPFAAIANEEKH....FYGVQFHPEVTHTRQGMRIIENFVLNICGCENLWTSANIIEDAIANIKEQVG.DDEVILGLSGGV
GUAA_COLP3      EGFVTVAQTASCAYGAMANEEKQ....FYGVQFHPEVTHTKQGSRILENFVVDICKCEKLWTSASIIDDAIAKMKAQVG.DDEVILGLSGGV
GUAA_PSYIN      AGFTTIANTATCQFAAIANEEKR....FYGVQFHPEVTHTRQGERMLRHFVMDICGCETLWTSSSIIEDAVARMKAQIG.DDEVILGLSGGV
GUAA_PSEHT      QSFKTTAKTSTCPHAAMSNEEKR....FYGVQFHPEVTHTHQGQRLLERFAIDICGCEKLWTAAKIIDNAIERIKETVG.DDEVILGLSGGV
GUAA_PSEA6      DGFITAAKTDSCPFAAIVHPEKQ....FYGVQFHPEVTHTRQGQRMLSHFVLDICQCEKLWTPDAIIEDAVARMKKQIG.DDEVILGLSGGV
GUAA_ALTMD      EGFETIAQTASCPHAAMYNEEKQ....FYGVQFHPEVTHTRQGMRLLTQFVMDICKCEKLWTAGAIIEDAIERIKEKVG.DERVILGLSGGV
GUAA_SHESW      EGFVAVAKTDTCPFAAMANEEKR....FYGVQFHPEVTHTRQGMRMLSHFALDICGCAANWKPSSIIEDAIERLKKQIG.DDEVILGLSGGV
GUAA_SHEPC      EGFVAVAKTDTCPFAAMANEEKR....FYGVQFHPEVTHTRQGMRMLSHFALDICGCAANWKPSSIIEDAIERLKKQIG.DDEVILGLSGGV
GUAA_SHEB9      EGFVAVAKTDTCPFAAMANEEKQ....FFGVQFHPEVTHTRQGMRMLSHFALDICGCAANWKPSSIIEDAIERLKKQIG.DDEVILGLSGGV
GUAA_SHEB8      EGFVAVAKTDTCPFAAMANEEKQ....FFGVQFHPEVTHTRQGMRMLSHFALDICGCAANWKPSSIIEDAIERLKKQIG.DDEVILGLSGGV
GUAA_SHEB5      EGFVAVAKTDTCPFAAMANEEKQ....FFGVQFHPEVTHTRQGMRMLSHFALDICGCAANWKPSSIIEDAIERLKKQIG.DDEVILGLSGGV
GUAA_SHEB2      EGFVAVAKTDTCPFAAMANEEKQ....FFGVQFHPEVTHTRQGMRMLSHFALDICGCAANWKPSSIIEDAIERLKKQIG.DDEVILGLSGGV
GUAA_SHESM      EGFVAVAKTDTCPFAAMSCEEKR....FYGVQFHPEVTHTRQGMRMLSHFALDICGCAANWKPSSIIEDAIERLKKQVG.DDEVILGLSGGV
GUAA_SHESA      EGFVAVAKTDTCPFAAMSCEEKR....FYGVQFHPEVTHTRQGMRMLSHFALDICGCAANWKPSSIIEDAIERLKKQVG.DDEVILGLSGGV
GUAA_SHESR      EGFVAVAKTDTCPFAAMSCEEKR....FYGVQFHPEVTHTRQGMRMLSHFALDICGCAANWKPSSIIEDAIERLKKQVG.DDEVILGLSGGV
GUAA_SHEON      AGFVAVAKTDTCPFAAMSCEEKR....FYGVQFHPEVTHTRQGMRMLSHFALDICGCAANWKPSSIIEDAIERLKKQVG.DDEVILGLSGGV
GUAA_SHEDO      EGFKAVAKTDTCPFAAMANEEKR....FYGVQFHPEVTHTRQGLRMLEHFAVAICGCGTNWKPSSIIEDAIERIKAQVG.DDEVILGLSGGV
GUAA_SHEPW      DGFVTVANTETCPFAAMANEDKK....FYGVQFHPEVTHTRQGKRMLEHFALDICGCDANWKPSSIIEDAVERLKKQIG.DDEVILGLSGGV
GUAA_SHESH      EGFVTVANTETCPHAAMANEDKK....FYGVQFHPEVTHTRQGKRMLEHFALDICECEANWKPTSIIEDAVERLKKQIG.DDEVILGLSGGV
GUAA_SHEWM      EGFVTVANTETCPYAAMANEEKK....FYGVQFHPEVTHTRQGKRMLEHFALDICQCDANWKPASIIEDAIERLKQQIG.DDEVILGLSGGV
GUAA_SHEHH      EGFVAVAKTETCPFAAMANEDKQ....FYGVQFHPEVTHTRQGKRMLEHFALEICACAANWKPASIIEDAIERLKAQIG.DDEVILGLSGGV
GUAA_SHEPA      EGFVAVAKTETCPFAAMANEDKQ....FYGVQFHPEVTHTRQGKRMLEHFALEICGCPANWKPSSIIEDAIERLKEQIG.DDEVILGLSGGV
GUAA_SHEFN      DGFVTVAKTATCPFAAMANEEKG....FYGVQFHPEVTHTRQGQRMLEHFALDICGCKANWKPSSIIEDAIERLKKQIG.DDEVILGLSGGV
GUAA_SHELP      EGFVTVAKTDTCPYAAMANEEKR....FYGVQFHPEVTHTRQGKRMLEHFALDICQCEANWKPSSIIEDAVARIKEQVG.DDEVILGLSGGV
GUAA_SHEAM      EGFVAVAKTETCPFAVMANEDKK....FYGVQFHPEVTHTRQGKRMLEHFVMDICGCDNKWQPASIIEDAVERIKAQVG.DDEVILGLSGGV
GUAA_IDILO      EGFHTVAKTSYCPYAAMADEERQ....FYGVQFHPEVTHTRQGQRMLEHFVSDICGCEKQWTPAKIIDDAIERIREQVG.SDKVILGLSGGV
GUAA_ACTSZ      EGFQVTGTTPTCPIAAMSDEKRH....FYGVQFHPEVTHTKSGLALLTNFVVNICGCATNWTPENIIEDAVARIKAQVG.DDEVILGLSGGV
GUAA_MANSM      EGFQLTGTTSTCPIAAMSDESRH....FYGVQFHPEVTHTKSGLALLTNFVVNICGCTTNWTPENIIEDAVARIKAQVG.DDEVILGLSGGV
GUAA_ACTPJ      QGFQVTGITPTCPIAAMSDESRR....FYGVQFHPEVTHTKSGLELLTNFVVGICGCECKWTAENIIEDAVARIKEQVG.DDEVILGLSGGV
GUAA_ACTP7      QGFQVTGITPTCPIAAMSDESRR....FYGVQFHPEVTHTKSGLELLTNFVVGICGCECKWTAENIIEDAVARIKEQVG.DDEVILGLSGGV
GUAA_ACTP2      QGFQVTGITPTCPIAAMSDESRR....FYGVQFHPEVTHTKSGLELLTNFVVGICGCECKWTAENIIERRVARIKEQVG.DDEVILGLSGGV
GUAA_HAEPS      SNFQITGVTPTCPIAAMSDESRR....FYGVQFHPEVTHTKSGLALLTNFVVNICGCETKWTAENIIEDAVARIKAQVG.DDEVILGLSGGV
GUAA_PASMU      DHFQVTAMTSTCPIAAMSDERRR....FYGVQFHPEVTHTKSGLELLTNFVVKICGCERNWTPENIIEDAVARLKAQVG.DDEVILGLSGGV
GUAA_HAEDU      AGFQTTAVTPTCPIAAMSDEKRR....FYGVQFHPEVTHTKSGLALLTNFVENICGCARSWTPENIIEDAVAKIKQKVG.NDQVILGLSGGV
GUAA_HISS1      DNFQITGMTSTCPIAAMSDESRR....FYGVQFHPEVTHTKCGQKLLQNFVVDICGCETNWTAENIIEDAVARIKAQVG.GDEVILGLSGGV
GUAA_HISS2      DNFQITGMTSTCPIAAMSDESRR....FYCVQFHPEVTHTKCGQKLLQNFVVDICGCETNWTAENIIEDAVARIKAQVG.DDEVILGLSGGV
GUAA_HAEI8      ENFKVTGTTPTCPIAAMSDENRR....FYGVQFHPEVTHTKKGLELLTNFVVNICGCETKWTAENIIEDAVARIKEQVG.DDEVILGLSGGV
GUAA_HAEIG      ENFKVTGTTLTCPIAAMSDENRR....FYGVQFHPEVTHTKKGLELLTNFVVNICGCETKWTAENIIEDAVARIKEQVG.NDEVILGLSGGV
GUAA_HAEIN      ENFKVTGTTLTCPIAAMSDESRR....FYGVQFHPEVTHTKKGLELLTNFVVNICGCETKWTAENIIEDAVARIKEQVG.DDEVILGLSGGV
GUAA_HAEIE      QNFQVTGTTPTCPIAAMSDESRR....FYGVQFHPEVTHTKKGLELLTNFVVNICGCETKWTAENIIEDAVARIKEQVG.DDEVILGLSGGV
GUAA_HAMD5      EHFTCIASTETCPIAIMAHPEKH....FYGVQFHPEVTHTLQGQSLLKRFVLQICQCEPLWTPRNIIDQTLSELKDQIG.DDQVILGLSGGV
GUAA_XANC5      PGFTITATTDRIPVAAMSNEAKR....WYGVQFHPEVTHTLQGQTLLRRFVVDVCGCQTLWTAANIIEDQIARVREQVG.DDEVILGLSGGV
GUAA_XANAC      PGFTITATTDRIPVAAMSNEAKR....WYGVQFHPEVTHTLQGQTLLRRFVVDVCGCQTLWTAANIIEDQIARVREQVG.DDEVILGLSGGV
GUAA_XANCP      PGFTITAVTDRIPVAAMSNEDKR....WYGVQFHPEVTHTLQGQTLLRRFVVDVCGCQTLWTAANIIDDQIARVREQVG.DDEVILGLSGGV
GUAA_XANCB      PGFTITAVTDRIPVAAMSNEDKR....WYGVQFHPEVTHTLQGQTLLRRFVVDVCGCQTLWTAANIIDDQIARVREQVG.DDEVILGLSGGV
GUAA_XANC8      PGFTITAVTDRIPVAAMSNEDKR....WYGVQFHPEVTHTLQGQTLLRRFVVDVCGCQTLWTAANIIDDQIARVREQVG.DDEVILGLSGGV
GUAA_XANOR      PGFTITATTDRIPVAAMSNEDKR....WYGVQFHPEVTHTLQGQTLLRRFVVDICGCQTLWTAANIIDDQIARVREQVG.DDDVILGLSGGV
GUAA_XANOM      PGFTITATTDRIPVAAMSNEDKR....WYGVQFHPEVTHTLQGQTLLRRFVVDICGCQTLWTAANIIDDQIARVREQVG.DDDVILGLSGGV
GUAA_XANOP      PGFTITATTDRIPVAAMSNEDKR....WYGVQFHPEVTHTLQGQTLLRRFVVDICGCQTLWTAANIIDDQIARVREQVG.DDDVILGLSGGV
GUAA_STRMK      PGFTITATTDRIPVAAMANEEKR....WYGVQFHPEVTHTLQGQALLRRFVVDVCGCQTLWTAANIIDDQIARVREQVG.DDEVILGLSGGV
GUAA_STRM5      PGFTITATTDRIPVAAMANEEKR....WYGVQFHPEVTHTLQGQALLRRFVVDVCGCQTLWTAANIIDDQITRVREQVG.DDEVILGLSGGV
GUAA_XYLFT      PCFTITAATDRIPIAAMSREDKR....WYGVQFHPEVTHTLQGQALLRRFVVDICGCQTLWTSANIIEDQIARVRERVG.CDEVILGLSGGV
GUAA_XYLF2      PCFTITAATDRIPIAAMSREDKR....WYGVQFHPEVTHTLQGQALLRRFVVDICGCQTLWTSANIIEDQIARVRERVG.CDEVILGLSGGV
GUAA_XYLFM      PGFTITAATDRIPIAAMSREDKR....WYGVQFHPEVTHTLQGQALLRRFVVDICGCQTLWTAANIIEDQIARVRERVG.RDEVILGLSGGV
GUAA_XYLFA      PGFTITAATDRIPIAAMSREDKR....WYGVQFHPEVTHTLQGQALLRRFVVDICGCQMLWTAANIIEDQIAHVRERVG.CDEVILGLSGGV
GUAA_CELJU      EGFELMASTPSCPIAGMYNEAKK....FYGVQFHPEVTHTLQGKRLFEHFVLQLCACEKLWTPANIVEDAIKKVREQVG.TDKVLLGLSGGV
GUAA_TERTT      DTFSLLASTPSCPIAGMSWEEKR....FYGVQFHPEVTHTLQGKRIFEHFVLEIAGCEPLWTPANIVEDAIERVREKVG.SDKVLLGLSGGV
GUAA_HAHCH      EGFVISASTPSAPIAAMQHESKP....WFGVQFHPEVTHTLQGRRMLERFLVNICGCKQLWTPGRIIEDAITQVRNQVG.QDKVLLGLSGGV
GUAA_MARAV      EGFELLASTESAPIAAMQDLSRN....LYGVQFHPEVTHTLQGKRILEHFVLTISGCEALWTPAKIVDDAVRQIREQVG.SDKVLLGLSGGV
GUAA_PSESM      EDFHILASTPSCPIAGMADDARG....YYGVQFHPEVTHTKQGGRILSRFILDICGCEALWTPSKIAEDAIAQVRAQVG.TDSVLLGLSGGV
GUAA_PSE14      EDFHILASTPSCPIAGMADDARG....YYGVQFHPEVTHTKQGGRILSRFILDICGCEALWTPSKIAEDAIAQVRAQVG.TDNVLLGLSGGV
GUAA_PSEU2      ENFHILASTPSCPIAGMADDARG....YYGVQFHPEVTHTKQGGRILSRFILDICGCEALWTPSKIAEDAIAQVRAQVG.TDNVLLGLSGGV
GUAA_PSEFS      EDFHILASTPSCPIAGMFSDERR....YYGVQFHPEVTHTKQGGRILSRFILDICECEALWTPSKIAEDAIAQVRAQVG.TDNVLLGLSGGV
GUAA_PSEF5      EDFHILASTPSCPIAGMFNDDLR....YYGVQFHPEVTHTKQGGRILSRFILDICGCEALWTPSKIAEDAIAQIRAQVG.TDNVLLGLSGGV
GUAA_PSEPF      SDFHILASTPSCPIAGMFNDERA....YYGVQFHPEVTHTKQGGRILSRFVLDICGCEALWTPSKIAEDAIANIRAQVG.TDNVLLGLSGGV
```

GUAA_PSEPK	GNFNILASTPSCPIAGMYDDVRG....YYGVQFHPEVTHTKQGGRILSRFVQDICGCEALWTASNIVEDAIAQVRAQVG.SANVLLGLSGGV
GUAA_PSEP1	GNFNILASTPSCPIAGMYDDVRG....YYGVQFHPEVTHTKQGGRILSRFVQDICGCEALWTASNIVEDAIAQVRAQVG.SANVLLGLSGGV
GUAA_PSEPG	GNFNILASTPSCPIAGMYDDARG....YYGVQFHPEVTHTKQGGRILSRFVQDICGCEALWTASNIVEDAIAQVRAQVG.SANVLLGLSGGV
GUAA_PSEE4	GNFSVLASTPSCPIAGMYDDALG....YYGVQFHPEVTHTKQGGRILSRFVQDICGCEALWTASNIVEDAIAQVRAQVG.SANVLLGLSGGV
GUAA_PSEPW	GNFNILASTPSCPIAGMFDDARG....YYGVQFHPEVTHTKQGGRILSRFVQDICGCEALWTPSNIVEDAIAQVRQQVG.SANVLLGLSGGV
GUAA_PSEMY	EGFHILASTPSCPIAAMSDDTRH....YYGVQFHPEVTHTKQGGRILSRFVLEISGCEALWTPANIVEDAVAAVRAQVG.DANVLLGLSGGV
GUAA_PSEAE	AGFHILASTPSCPIAAMADDARA....YYGVQFHPEVTHTKQGLRILSRFVLDICGCAALWTPSNIVDDAIATVRAQVG.SSKVLLGLSGGV
GUAA_PSEA8	AGFHILASTPSCPIAAMADDARA....YYGVQFHPEVTHTKQGLRILSRFVLDICGCAALWTPSNIVDDAIATVRAQVG.SSKVLLGLSGGV
GUAA_PSEAB	AGFHILASTPSCPIAAMADDARA....YYGVQFHPEVTHTKQGLRILSRFVLDICGCAALWTPSNIVDDAIATVRAQVG.SSKVLLGLSGGV
GUAA_MARMS	EDFTLMASTDSCPIAAMANEAKK....FYGVQFHPEVTHTLQGGRILSRFIVDICGCDTLWTPANIAQDAIERMQEQVG.DKKVLLALSGGV
GUAA_ALCBS	AGFVATATTGSCPIAGMANDEKR....FYGIQFHPEVTHTLQGGRLLERFIRELCECEALWTPSNIISDAIDTIREQVG.TDEVILGLSGGV
GUAA_THICR	EGFKLMASTGNCPIAGMANEEKD....FYGIQFHPEVTHTTQGQRMIERFVVDLCGCEKLWTTENIIDDSIARIRQQVG.SDEVLLGLSGGV
GUAA_ALKEH	PGFKLIASTESCPIAGIGDDERG....YYGVQFHPEVTHTPQGARILSRFVHEICGCPADWTPGNIVDDLIERVRRQVG.GDKVLLGLSGGV
GUAA_METCA	PGFKLIASTESAPIAGMADEERR....FYALQFHPEVTHTRQGKRILERFVRKLCQCEARWTTGNIVEDAITRVRNRIG.AERVVLGLSGGV
GUAA_NITOC	EGFKRIAATAHAPLAGMADEKRR....FYGFQFHPEVTHTRQGIRILERFIYDICGCEALWEPRHIIAKSIENIRTKVG.ADTVLLGLSGGV
GUAA_LEGPL	PGFKVICETRNAPIAGMADESRQ....MYGLQFHPEVTHTLQGLRILQRFVVDICKASTDWTPEHIIDEAINKIREQVG.TEKVLLGLSGGV
GUAA_LEGPA	PGFKVICETRNAPIAGMADESRQ....MYGLQFHPEVTHTLQGLRILQRFVVDICKASTDWTPEHIIDEAINKIREQVG.TEKVLLGLSGGV
GUAA_LEGPC	PGFKVICETRNAPIAGMADESRQ....MYGLQFHPEVTHTLQGLRILQRFVVDICKAPTEWTPEHIIDEAINKIREQVG.TEKVLLGLSGGV
GUAA_LEGPH	PGFKIICETRNAPIAGMADESRQ....MYGLQFHPEVTHTLQGLRILQRFVVDICKASTDWTPEHIIDEAINKIREQVG.TEKVLLGLSGGV
GUAA_ACIAD	EGFSTTASTPSCPFAAASDEARR....FYGVQFHPEVTHTAKGAELLANFVHKICGCGGLWTPEHIIDLRVEQLRAQIG.DEKVLLGLSGGV
GUAA_PSYA2	QGFDIVASTPSCPIAIMADDSRH....YYGLQFHPEVTHTAQGQALLGRFVHEICDCAGSWTPDNIIDMRIEQLKKQIG.DKQVLLGLSGGV
GUAA_RUTMC	NGFKLIASTDNCNIAGFANVEKH....YYGLQFHPEVTHTKQGERILERFISGICQCEKNWTTDNIIAKLVQNLKDQIG.NANILLGLSGGV
GUAA_FRAP2	EHFEVIASSTNAPVAAVAHKSKP....FYGVQFHPETTHTENGKQIIENFALNICKCDALWNIENIIENDIKEIKKQVG.SDRVILGLSGGV
GUAA_BURPP	PGFALMASTESCPIAAMADEKRH....FYGLQWHPEVTHTVQGRAMLERFVLQICGARADWEMGNYIDEAVAKIREQVG.NEHVILGLSGGV
GUAA_BURXL	PGFALMASTESCPIAAMADETRH....FYGLQWHPEVTHTVQGRAMLERFVLKICGAQPDWEMGHYIDEAVAKIREQVG.QEHVILGLSGGV
GUAA_BURP8	QGFQLMASTESCPIAAMADETRH....FYGVQWHPEVTHTVQGRAMLERFVLKICGAKPDWEMGNYIDEAVENIRKQVG.DEHVILGLSGGV
GUAA_POLNS	SSFKLMASTESCPIAGMADEERS....FYAFQFHPEVTHTIQGTAIIERFVHEICKCKPDWVMGDYIAEAVENIRKQVG.DEEVILGLSGGV
GUAA_POLSQ	PAFKLMASTESCPIAGMADEDRR....FYAFQFHPEVTHTIQGTAIIERFVHEICHCKPDWVMGDYIAEAVEHIRKQVG.DEEVILGLSGGV
GUAA_DECAR	AGFVKMASTASCPIAAMADESRK....FYAVQFHPEVTHTQQGRAILERFVHGICGCGTDWVMGDYIAEAVDAIRRQVG.DEEVILGLSGGV
GUAA_HERAR	PGFKLMASTPNCPIAGMADEARH....MYAFQFHPEVTHTLQGKAIIARFVHDICGCKSDWNMPDYIAEAVEKIRQQVG.NDEVILGLSGGV
GUAA_JANMA	PGFKLMASTPNCPIAGMADEERR....MYAFQFHPEVTHTVQGKAIIARFVHDICGCKSDWNMPDYIAEAVEKIRQQVG.SDEVILGLSGGV
GUAA_AROAE	PGFKVIASNESCPVAGMADEVRK....FYAVQFHPEVTHTIKGRDMLARFVHEICGCGRDWNMPDYVNEAIEKVRAQVG.QEEVILGLSGGV
GUAA_AZOSB	AGFKVIGSNESCPVAAMADEERG....FYGVQFHPEVTHTIKGKEMLARFVHEICGCGNDWNMPDYISEAVEKIRAQVG.DEEVILGLSGGV
GUAA_METFK	EGFKIIASNSATPIAGMADENRR....FYAVQFHPEVTHTKQGKALLNRFVHDICGCDQSWNMPDYVETAVQRIREEVG.CDEVILGLSGGV
GUAA_THIDA	EGFKLMASNAATPIAGMADEARR....FYGVQFHPEVTHTLQGKHILNRFVHDICGCGSDWNMPDYVEEAIGKVRSEVG.SDEVILGLSGGV
GUAA_NITEC	PGFTAIAFNAATPFAAMADETRR....FYGVQFHPEVTHTLQGKAILDRFVHDICDAGYDWNMPDYVEEAIGRIRARVG.GDKVILGLSGGV
GUAA_NITEU	PGFTAIASNAATPFAAMADETRN....FYGVQFHPEVTHTRQGKAILDHFVHDICSAGYDWNMPDYVEEAIGRIRARVG.NDKVVLGLSGGV
GUAA_NITMU	PGFQVMASNPATPLAAIADGARR....FYGMQFHPEVTHTPQGKAIIDRFVHEICGLAYDWNMPDYLEEMVGRIRSEVG.PDEVILGLSGGV
GUAA_NEIMA	TGFTVIGDTPSCPIAMMENAEKQ....FYGIQFHPEVTHTKQGRALLNRFVLDICGAQPGWTMPNYIEEAVAKIREQVG.SDEVILGLSGGV
GUAA_NEIMF	TGFTVIGDTPSCPIAMMENAEKQ....FYGIQFHPEVTHTKQGRALLNRFVLDICGAQPSWTMPNYIEEAVAKIREQVG.SDEVILGLSGGV
GUAA_NEIG1	DGFAVIGDTPSCPIAMMENAEKQ....FYGIQFHPEVTHTKQGRALLNRFVLDICGAQPGWTMPNYIEEAVAKIREQVG.SDEVILGLSGGV
GUAA_NEIG2	DGFAVIGDTPSCPIAMMENTEKQ....FYGIQFHPEVTHTKQGRALLNRFVLDICGAQPGWTMPNYIEEAVAKIREQVG.SDEVILGLSGGV
GUAA_NEIM0	DGFAVIGDTPSCPIAMMENAEKQ....FYGIQFHPEVTHTKQGRALLNRFVLDICGAQPGWTMPNYIEEAVAKIREQVG.SDEVILGLSGGV
GUAA_NEIMB	DGFAVIGNTPSCPIAMMENAEKQ....FYGIQFHPEVTHTKQGRALLNRFVLDICGAQPGWTMPNYIEEAVAKIREQVG.SDEVILGLSGGV
GUAA_GEOMG	AGFQVVGETANAPVCAIQDLARN....LYGVQFHPEVNHTPRGELLIDTFVRKICGCSGQWTPGHIIDDAVSRIRAQVG.NDRVILGLSGGV
GUAA_GEOSL	QGFQVVAETANAPVCAIQDTARN....LYGVQFHPEVNHTPRGEILIDTFVRKICGCTGQWTPGHIIEDAVNRIRAQVG.RERVILGLSGGV
GUAA_GEODF	EGFQVAAGTANAPVCAIQNLACN....LYGVQFHPEVNHTPRGEQLIDTFVRKICGCSGKWTPGQIIEDAVARIKQQVG.SDRVILGLSGGV
GUAA_PELPD	DGFQVVAYTENAPVCAIQDVSRN....LYGVQFHPEVNHTPRGDQLIDTFVRAICGCTGHWTPGQIIEDAVTRIQEQVG.SERVILGLSGGV
GUAA_PELCD	DGFEVVGCSENAPVCAIQDIARD....LYGVQFHPEVNHTPRGEQLLDTFVRQICGCTGQWTPGQIIEDAVVRIREQVG.SERVILGLSGGV
GUAA_BARHE	EGFRVIGTSKGAPYAAIADEKRR....LYAVQFHPEVVHTLDGTKLLQNFVLKISNLKGNWSMASYREQTIAKIRQKVG.KSRVICGLSGGV
GUAA_BARQU	EGFRVIGTSKGAPYAAIADEKRC....LYAVQFHPEVVHTPDGTKLLQNFVHKISGLKGNWSMASYRDQAIATIRKKVG.KSRVICGLSGGV
GUAA_BARBK	EGFCVIGTSKGAPYAAISDEKRN....FYAVQFHPEVVHTPDGEKLLQNFVCKISGIKNNWSMAAYRDQAIAAIREKVG.KNRVICGLSGGV
GUAA_RHIE6	DGFEVVATSSNAPFAFIADEKRK....YYGVQFHPEVVHTPDGAKLIGNFIHNIAGIKGDWSMSAYRQKAVDEIRKQVG.DKRVICALSGGV
GUAA_RHILW	DGFEVVATSSNAPYAFIADEKRK....YYGVQFHPEVVHTPDGAKLIGNFIHNIAGIKGDWSMSAYRQKAVDEIRKQVG.DKRVICALSGGV
GUAA_RHIEC	DGFKVVATSSNAPFAFIADEKRK....YYGVQFHPEVVHTPDGAKLIGNFIHNVAGIKGDWSMSAYRQKAVEEIRKQVG.DKRVICALSGGV
GUAA_RHIL3	DGFEVVATSSNAPYAFIADEKRK....YYGVQFHPEVVHTPDGAKLIGNFIHNIAGIKGDWSMSAYRQKAVEQIRAQVG.DKRVICALSGGV
GUAA_AGRRK	DGFEVVATSSNAPYAFIADEKRK....YYGVQFHPEVVHTPDGAKLIGNFIHNIAGIKGDWSMSAYRAKAVQAIRDQVG.DKRVICALSGGV
GUAA_RHISN	EGFEVVATSSNAPFAFIADETRK....YYAVQFHPEVVHTPDGAKLISNFVHKIAGIKGDWTMSAYRAKAVEAIRKQVG.GKKVICALSGGV
GUAA_RHIME	EGFEVVATSSNAPFAFIADEKRK....YYAVQFHPEVVHTPDGAKLIANFVHKIAGITGDWTMSAYRAKAVEAIRKQVG.DKKVICALSGGV
GUAA_SINMW	DGFEVVATSSNAPFAFIADEKRR....YYAVQFHPEVVHTPDGAKLIANFVHKIAGIKGDWTMSAYRAKAVEAIRNQVG.DKKVICALSGGV
GUAA_AGRVS	PGFRVLATSSNAPFAFIADEARK....YYAVQFHPEVVHTPDGAKLIQNFVHNIAGITGDWSMSAYRAKAVEQIRAQVG.DKRVICALSGGV
GUAA_BRUSI	DGFTIIGTSPNAPYAVIADEKRK....YYGVQFHPEVVHTPDGAKLLQNFVHRIVGVKPGWTMGAYREQAVEAIRKQVG.SGKVICALSGGV
GUAA_BRUAB	DGFTIIGTSPNAPYAVIADEKRK....YYGVQFHPEVVHTPDGAKLLQNFVHRIVGVKPGWTMGAYREQAVEAIRKQVG.SGKVICALSGGV
GUAA_BRUA2	DGFTIIGTSPNAPYAVIADEKRK....YYGVQFHPEVVHTPDGAKLLQNFVHRIVGVKPGWTMGAYREQAVEAIRKQVG.SGKVICALSGGV
GUAA_BRUA1	DGFTIIGTSPNAPYAVIADEKRK....YYGVQFHPEVVHTPDGAKLLQNFVHRIVGVKPGWTMGAYREQAVEAIRKQVG.SGKVICALSGGV
GUAA_BRUO2	DGFTIIGTSPNAPYAVIADEKRK....YYGVQFHPEVVHTPDGAKLLQNFVHRIVGVKPGWTMGAYREQAVEAIRKQVG.SGKVICALSGGV
GUAA_BRUSU	DGFTIIGTSPNAPYAVIADEKRK....YYGVQFHPEVVHTPDGAKLLQNFVHRIVGVKPGWTMGAYREQAVEAIRKQVG.SGKVICALSGGV
GUAA_BRUC2	DGFTIIGTSPNAPYAVIADEKRK....YYGVQFHPEVVHTPDGAKLLQNFVHRIVGVKPGWTMGAYREQAVEAIRKQVG.SGKVICALSGGV
GUAA_BRUMB	DGFTIIGTSPNAPYAVIADEKRK....YYGVQFHPEVVHTPDGAKLLQNFVHRIVGVKPGWTMGAYREQAVEAIRKQVG.SGKVICALSGGV
GUAA_RHILO	PGFQVFGKSESSPFAIFGNVERK....MYGIMFHPEVVHTPDGARLLRNFVHNIAGIEGDWTMRAYREHAVEAIRKQVG.KGKVICALSGGV
GUAA_AGRT5	EGFEVIATSENAPFAIAADEKRH....YYTTMFHPEVVHTPDGGKLLSNFVHKIVGLKSDWTMAAYRAEMIRKIREQVG.TGRVLCALSGGV
GUAA_BRADU	PGFSVAGTSPNAPFAIIQDEKRK....YYGLMFHPEVVHTPDGAKLIRNFVRKIAGLTGDWTMRAFREEEIAKIRAQVG.KGKVICGLSGGV
GUAA_BRASO	PGFSVAGVSPNAPFAVIQDEARK....YYGLMFHPEVVHTPDGAKLIRNFVRNISGLGGDWTMHAFREEEIKKIRAQVG.QGKVICGLSGGV
GUAA_OLICO	EGFRGVARAAGSPIAIIADDTRK....FYAMQFHPEVVHTPDGAKLIRNFVRKVAGLKGDWTMRAFREEAIEKIRAQVG.QGRVICGLSGGV
GUAA_RUEST	PGFKVYGTSPNAPFAITADLERN....FYAVQFHPEVHHTPNGKTLYENFVR.LAGFSGDWTMGAYREQMVETIREQVG.DKKVICALSGGV
GUAA_RUEPO	PGFEVYGTSPGAPFAITADTSRH....FYAVQFHPEVHHTPNGAKLYENFVR.LAGFKGDWTMGAYREEMIRTIREQVG.DKKVICGLSGGV
GUAA_CAUCR	EGFEVIGTSTGAPFAAIANDAKK....IYALQFHPEVYHTVNGPAMYRNFLFNIAGLKGDWTMAAFRQEMVQKIRDQVG.DGKVICGLSGGV
GUAA_CAUCN	EGFEVIGTSTGAPFAAIANDAKK....IYALQFHPEVYHTVNGPAMYRNFLFNIAGLKGDWTMAAFRQEMVQKIRDQVG.DGKVICGLSGGV
GUAA_PHEZH	MGFETVAVSEGSPFAVIADEGRR....FYGIQFHPEVAHTPRGALILRNFTHKIAGLKGDWTMKAFREEAIARIRDQVG.DGRIICGLSGGV
GUAA_RHORT	EGFSVVAVSEGAPYAAIADEARH....FYGVQFHPEVVHTPKGAALLRNFTHGICGCGGDWTMAAFKDQAIARVREQVG.SGRVICGLSGGV
GUAA_GLUOX	PGFRAVAHSEGAPFAIIADEGRR....LYGVQFHPEVVHTPHGAALIRNFTHNVAGCSGTWTMAGFRELEIARIRKQVG.SGRVICGLSGGV
GUAA_RHOBA	DQFTAMAKTSTCPYAAIRHNERP....VFGMQFHPEVTHTPHGGQILRNFVIDVCGCDGSWKLGDFADAAIESIRKQVG.NKRVICGLSGGV
GUAA_BACFR	ENFKTIASTDKVAIAAYQVEGEQ....VWGVQFHPEVFHSEDGTQMLRNFVVDVCGCKQDWSPASFIESTVAELKAQLG.DDKVVLGLSGGV
GUAA_BACFN	ENFKTIASTDKVAIAAYQVEGEQ....VWGVQFHPEVFHSEDGTQMLRNFVVDVCGCKQDWSPASFIESTVAELKAQLG.DDKVVLGLSGGV
GUAA1_BACTN	DNFKKIASTDKVDIAAYQVEGEK....VWGVQFHPEVFHSEDGTQILRNFVVDVCGCKQDWSPASFIESTVAELKAQLG.DDKVVLGLSGGV
GUAA_CYTH3	SNFEILSSTETVRVAAYKVKGED....TFGIQFHPEVTHSLQGKELLRNFVVHICGCSQDWTPDAFVETTISELKAKIG.TDKVVLGLSGGV
GUAA_PELLD	EGFQITASSGNSEMCAIESFGPKAALKIYGLQFHPEVQHTLYGKQLLSNFLINIAGIRPDWSSKSFIEHQIEDIRQRAD.KGTVICGISGGV
GUAA_PROVI	EGFRVTASSGNSEMCAIESYGSKAALKIYGLQFHPEVQHSLYGKQLLGNFLLNIAGITPDWSSKSFIDHQIEDIRERAG.NNTVICGISGGV
GUAA_CHLL2	DGFRITASSANSEMCALETYGSKGALKVFGLQFHPEVQHTLYGKQLLSNFLIDIAGITPDWSPKHFIDHQIEEIRRTAG.DETVICGISGGV
GUAA_CHLPD	EGFRVTASSENSEMCALESYGSKAALKVYGLQFHPEVQHTLYGKQLLSNFLIDIAGIKPDWSPKSFIGHQIEEIRARAG.KDKVICGISGGV
GUAA_PELPB	EGFRVTASSENSEMCAFESFGSKGALKIYALQFHPEVQHTLYGKQLLSNFLITIAGITPDWSSKSFIEHQLEEISRKAG.KETVICGISGGV
GUAA_CHLTE	EGFRVTASTANAEVCAIESFGSKAALKVYGLQFHPEVQHSLYGKQLLSNFLIDIAGITPDWSPKSFIQHQIEEIKRVAG.DSTVVCGISGGV
GUAA_CHLCH	EGFRATASSDNSEICAFESTGVNSPAHIYGLQFHPEVQHTLYGKEMLGNFLLNIAAITPDWSSKSFIEHQIEEIRRKAG.NNTVICGISGGV
GUAA_CHLPB	EGFRVTASSGNSEMCAIEASGDKAALKVFGLQFHPEVQHTLYGKQLLSNFLLDIAGLTPDWSPKSFIEHQIEEIRTTVG.KNTVICGISGGV
GUAA_CHLT3	KGFHITAQTANSELCAIEVTEGEGAHRIFGLQFHPEVHHTEFGEKLLSNFLINIAGITPDWSPQSFIDSEIARIKELVG.DQTAICAISGGV
GUAA_SALRD	DGYEVIARTDNAPVAAVRDTDG....PYYGVQFHPEVVHTDYGRQILENFAHAICGCSGDWTPASFVEEQTETIRDRVG.DRHVILGLSGGV
GUAA_GEMAT	PGYVATAHSGN..TCAGFRHKTK....PIHAVQFHAEVHHSARGAEIIENFLFDICHCAPDWTPGHFIDLEVAKIREMVG.DRQVICGLSGGV
GUAA_KLULA	AGFKITATSENSPFCGIANEDK....QIYGIQFHPEVTHSTQGKTLLRNFAVDICKASQSWNMENFIDTEINRIRELVGPDAEVIGAVSGGV
GUAA_ASHGO	ADFHVTATTENSPFCGIAHDSK....PIFGIQFHPEVTHSSQGKTLLKNFAVEICQAAQTWTMENFIDTEIQRIRTLVGPTAEVIGAVSGGV
GUAA_YEAST	TGYKTIATSDNSPYCGIVHETK....PIYGIQFHPEVTHSTQGKTLLKNFAVDLCHAKQNWTMENFIDTEINRIRKLVGPTAEVIGAVSGGV
GUAA_CANGA	TGFKVIATSDNSPYCGIVHESK....QIYGIQFHPEVTHSSNGKTLLKNFAVDLCHAKQNWTMKNFIGTEVQRIRDLVGPTAEVIGAVSGGV
GUAA_DEBHA	TGFKVVATSDNSPFAAISNEKE....NIFGIQFHPEVTHTKQGKVLLRNFAIDICQASNNWTMENFIDTEIARIQKLVGPTAEVIGAVSGGV
GUAA_CANAL	TGFKVVATSDNSPFCGISNESE....HIYGIQFHPEVTHTVQGKKLLKNFAVDICQAKTNWSMENFIDTEIARIKKLVGPTAEVIGAVSGGV
GUAA_YARLI	TGYEVVATSDNSPYAGIAHTSE....KIWGIQFHPEVTHTTKGKQLLQNFAVDICGAAQKWSMENFVDTEIQRIRDLVGPHAEVIGAVSGGV
GUAA_PHANO	ENFTTVATTTNAPFAGIAHKEK....KYYGIQFHPEVTHTKMGKTVLKNFAVDICQSTTDWTMGKFVDQEITRIRKLVGEKGQVIGAVSGGV
GUAA_EMENI	EGFHTIGTTQNSEYAAIAHKSD....PIYGIQFHPEVTHTPQGGQLLKNFAVGICGAEQKWTMAEFIGQEIQRIRSLVGPDGQVLGAVSGGV
GUAA_ASPOR	EGFHTIATTPNSEYAGIAHETK....PIYGLQLHPEVTHTQNGTKLLKNFAVNICGCKQNWTMARFVDQEIARIRKLVGPTGQVLGAVSGGV
GUAA_ASPFU	EGFHTIATTANSEYAGIAHETK....PVYGIQFHPEVTHTPDGAKLLRNFAVDICGANPNWTMSKFVDQEILRIRKLVGETDHVLGAVSGGV
GUAA_USTMA	EDFVVIAKTPTAPFAAMAHKSK....PIYGVQFHPEVTHSLRGVELFDSF.VDICACRRDWTMETFIDKEIKRIREIVGPKGQVLGAISGGV
GUAA_DEHM1	AGFTALAYTENCPVAVMGNEAD.....IYGLQFHPEVVHSPNGKIILKNFVFNICKCHANWTMGNYIQESIHNIREQVG.DGQVICALSGGV
GUAA_DEHMC	AGFKSLAYTENCPVAVMGNESD.....IYGLQFHPEVVHSPHGKIILKNFVFNICKCHANWTMGNYIQESIQNIREQVG.DGQVICALSGGV
GUAA_DESPS	DQFEVSAESAHSPYAALRHRSKP....FVAVQFHPEVVHSIIGTDLLRNFVFGLCKCQATWTMQGFIESNVAAIKEKVG.DAHVICALSGGV
GUAA_BRAHW	EGFELIAKTPNTELAAIENKQKN....IYAIQFHPEVVHTENGIKIIENFLFNICKCERNWNMGSFIEYEIKRIRETVG.DKNVILGLSGGV
GUAA_DESVV	PGFKVVGRTATLEVAAMADEARR....IYAVQFHPEVHHSEDGARIINNFLFHVAKLKADWTMSSFVERAVKEMAEVVG.DRHVVCALSGGI
GUAA_DESVH	PGFKVVGRTATLEVAAMADEARR....IYAVQFHPEVHHSEDGARIINNFLFHVAKLKADWTMSSFVERAVKEMAEVVG.DRHVVCALSGGI
GUAA_DESVM	PGFAVVGRTATLDVAAMADDARR....IYAVQFHPEVHHTEEGTRIINNFLFHVAKLKADWTMSSFVERAIKEMAETVG.DRHVVCALSGGI
GUAA_DESAG	AGFTVTGSTETLEVAAMADDQRR....IYAVQFHPEVHHSEDGVRMLHNFLFKIAGIKADWSMSSFCERVIKEVSEQVGPDDQVVCALSGGI
GUAA_DESMR	DGFVVAARTKNVDIAALANASRR....IYALQFHPEVAHTEDGERILHNFLFEIAGLTSGWTMSSFLETELASLKEKVG.DDEVVCALSGGV
GUAA_DESAD	EGFKVCGTTESIPFAAMANEEKK....MYALQFHPEVAHTESGTTIINNFVFKVAGLKADWTMSSFVENCIEEMREKIG.DNQVVLGLSGGI
GUAA_CALBD	EGFKALASTENCSIAAFGDDTRK....IYGVQFHPEVVHTEYGQKIIENFLFNICNCHGDWKTSSFIEEKVNEIKDIVG.NQKVVCALSGGV
GUAA_CALS8	EGFEILASTENCAIAAFGSREKK....IYGVQFHPEVVHTEFGQEIIKNFLFNICGCKGDWKTSSFIEERINEIRKIVG.NQKVVCALSGGV
GUAA_CARHZ	EGFLVTAKTEYTPIAAMENRERN....LYAVQFHPEVVHTPKGKEILKNFLYEICGLTPDWTMESFAQKAIREIKEQVG.EEKVVCALSGGV
GUAA_CLOBL	KGFKIIATTDECPCAAMENAEKK....LYGVQFHPEVEHTLFGKKMLKNFLFNVCNLKGDWSMSSFAEQQIKAIKEKVG.DKKVICALSGGV
GUAA_CLOBK	KGFKIIATTDECPCAAMENAEKK....LYGVQFHPEVEHTLFGKKMLKNFLFNVCNLKGDWSMSSFAEQQIKAIKEKVG.DKKVICALSGGV
GUAA_CLOBM	KGFKIIATTDECPCAAMENAEKK....LYGVQFHPEVEHTLFGKKMLKNFLFNVCNLKGDWSMSSFAEQQIKAIKEKVG.DKKVICALSGGV
GUAA_CLOBJ	KGFKIIATTDECPCAAMENVEKK....LYGVQFHPEVEHTLFGKKMLKNFLFNVCNLKGDWSMSSFAEQQIKAIKEKVG.DKKVICALSGGV
GUAA_CLOB6	KGFKIIATTDECPCAAMENAEKK....LYGVQFHPEVEHTLFGKKMLKNFLFNVCNLKGDWTMSSFAEQQIKAIKEKVG.NKKVICALSGGV
GUAA_CLOBH	KGFKIIATTDECPCAAMENAEKK....LYGVQFHPEVEHTLFGKKMLKNFLFNVCNLKGDWSMSSFAEQQIKAIKEKVG.DKKVICALSGGV
GUAA_CLOB1	KGFKIIATTDECPCAAMENAEKK....LYGVQFHPEVEHTLFGKKMLKNFLFNVCNLKGDWSMSSFAEQQIKAIKEKVG.DKKVICALSGGV
GUAA_CLONN	EGFKIIATTDQCPVAAMANEEKK....IYGVQFHPEVEHTLFGQKMLGNFLFKVAGLKADWSMASFAEEKIKAIKELVG.DKKVLCALSGGV
GUAA_CLOAB	EGFKVVGHTNVCPVAAMANTEKK....IYGVQFHPEVLHTPFGEQLFSNFLFKICGLKEDWSMSSFAKEKIQEIKDIVG.DKKVLCALSGGV
GUAA_CLOTE	SGFNIVAYTDGCKVAAMANEDKK....IYGVQFHPEVEHTPFGKKMLKNFLFNICELKGDWSVTSFAEEKIKEIRELVG.DKKVICALSGGV
GUAA_CLOPE	EGFKIIAHTDVCPVAAMENEERR....LYGVQFHPEVEHTPFGQNMMRNFLYNICGLENSWSMASFAEEKIAEIKKIVG.DKKLICALSGGV
GUAA_CLOB8	KGFKVIATTEVCPVAAMENEEKR....LYGVQFHAEVEHTQFGQKMLENFIHNICGLENSWSMGSFAEEKIKEIKELVG.DKKVLCALSGGV
GUAA_ALKMQ	AGFKITGTTADCPVAAMENQEKA....LYAVQFHPEVEHTEKGKEILKNFLYEVCHCQGDWTMENYIEKEIEAIRNLVG.DRKVLCALSGGV
GUAA_CLOTH	EGFVKCADTPNCPVAAIENREKK....LYGVQFHPEVVHTPKGRDILNNFLYKICGCSGDWKMASFIEHSINSIREKVG.DKKVLCALSGGV
GUAA_CLOCE	KGFEVIAKSANCPTGAMQHVEKN....LYAVQFHPEVMHTPKGKEMLKNFLYNICGCKGDWKMSSFVENSINAIREKVG.DKKVLCALSGGV
GUAA_CLOBA	EGFKVIGKTPVCPVAAMECEDKN....LYAVQFHPEVMHTEEGTKMLSNFVYNICGCTGDWKMDSFVEKTIEEVRQKVG.NGKVLCALSGGV
GUAA_PEPD6	EGFTIVANTNDCPVAAMENVERN....LYGVQFHPEVEHCLEGDKILTNFLYNICKVKGDWTTDSFIEDKIKELKEKIG.DKKALCALSGGV
GUAA_FINM2	EGFDIIQTTDSCPVAAIANEDKK....IFAVQYHPEVNHTVEGKTLIKNFLYEICKADGDWTMENFLEEQIQKIRKTVG.DKKVLLALSGGV
GUAA_TREDE	EGFVSAAQTANTKNAAMANKEKK....LYGIQFHAEVEHSEEGQNIIKNFLYNVCGAKGDWNMKSFLAEAIKDVQNTVK.DGKVLLALSGGV
GUAA_BACCR	EGFVVDATSESCPIAGMSNEAKN....LYGVQFHPEVRHSEHGNDLIKNFVFGVCGCSEGWNMENFIEVELEKIRETVG.DKKVLCALSGGV
GUAA_BACC4	EGFVVDATSESCPIAGMSNEAKN....LYGVQFHPEVRHSEHGNDLIKNFVFGVCGCSEGWNMENFIEVELEKIRETVG.DKKVLCALSGGV
GUAA_BACC2	EGFVVDATSESCPIAGMSNEEKN....LYGVQFHPEVRHSEHGNDLIKNFVFGVCGCSEGWNMENFIEVELEKIRETVG.DKKVLCALSGGV

```
GUAA_BACWK              EGFVVDATSESCPIAGMSNEGEN....LYGVQFHPEVRHSEHGNDLIKNFVFGVCGCSEGWNMENFIEVELEKIRETVG.DKKVLCALSGGV
GUAA_BACCZ              EGFVVDATSESCPIAGMSNEAKN....LYGVQFHPEVRHSEHGNDLIKNFVFGVCGCSEGWNMENFIEVELEKIRETVG.DKKVLCALSGGV
GUAA_BACC3              EGFVVDATSESCPIAGMSNEAKN....LYGVQFHPEVRHSEHGNDLIKNFVFGVCGCSEGWNMENFIEVELEKIRETVG.DKKVLCALSGGV
GUAA_BACAH              EGFVVDATSESCPIAGMSNEAKN....LYGVQFHPEVRHSEHGNDLIKNFVFGVCGCSEGWNMENFIEVELEKIRETVG.DKKVLCALSGGV
GUAA_BACAC              EGFVVDATSESCPIAGMSNEAKN....LYGVQFHPEVRHSEHGNDLIKNFVFGVCGCSEGWNMENFIEVELEKIRETVG.DKKVLCALSGGV
GUAA_BACAA              EGFVVDATSESCPIAGMSNEAKN....LYGVQFHPEVRHSEHGNDLIKNFVFGVCGCSEGWNMENFIEVELEKIRETVG.DKKVLCALSGGV
GUAA_BACHK              EGFVVDATSESCPIAGMSNEAKN....LYGVQFHPEVRHSEHGNDLIKNFVFGVCGCSEGWNMENFIEVELEKIRETVG.DKKVLCALSGGV
GUAA_BACC0              EGFVVDATSESCPIAGMSNEAKN....LYGVQFHPEVRHSEHGNDLIKNFVFGVCGCSEGWNMENFIEVELEKIRETVG.DKKVLCALSGGV
GUAA_BACAN              EGFVVDATSESCPIAGMSNEAKN....LYGVQFHPEVRHSEHGNDLIKNFVFGVCGCSEGWNMENFIEVELEKIRETVG.DKKVLCALSGGV
GUAA_BACC1              EGFVVDATSESCPIAGMSNEAKN....LYGVQFHPEVRHSEHGNDLIKNFVFGVCGCSEGWNMENFIEVELEKIRETVG.DKKVLCALSGGV
GUAA_BACCN              EGFVVDATSESCPIAGMSNEEKQ....LYGVQFHPEVRHSEHGNDLIKNFVFGVCGCSEGWNMENFIEVELEKIRETVG.DKKVLCALSGGV
GUAA_BACA2              EGFTVDATSHHCPNSAMSKADKN....WYGVQFHPEVRHSEYGNDLLKNFVFGVCDCEGKWSMENFIEIEMQKIRETVG.DKQVLCALSGGV
GUAA_BACSU              EGFTVDATSHHCPNSAMSKADKK....WYGVQFHPEVRHSEYGNDLLKNFVFGVCECEGEWSMENFIEIEMQKIRETVG.DKQVLCALSGGV
GUAA_BACLD              EGFTVDATSHHCPNSAMSKKDKK....WYGVQFHPEVRHSEYGNDLLKNFVFGVCECVGEWSMENFIEIEMQKIRETVG.DKQVLCALSGGV
GUAA_BACP2              EGFTVDATSAHCPNSAMSLAEKN....FYGVQFHPEVRHSEYGNDLLKNFVFGVCDCDGKWSMENFIEIEMQKIRETVG.DKQVLCALSGGV
GUAA_BACSK              EGFTVDASNPSCPVAAMSDESRN....LYGVQFHPEVRHSEFGNDLLKNFAFDICGAKGDWSMENFIEEEIKKIREQVG.DRHVLCALSGGV
GUAA_BACHD              EGFVVDAQNPSCPVAAMSDEARN....YYGVQFQPEVRHSQFGDDMLKNFAFAVCGCEGNWSMENFIELEMEKIREQVG.DKQVLCALSGGV
GUAA_GEOKA              AGFAVDATSPSCPIAAMSDERRK....WYGVQFHPEVRHSVYGNDLLKKFVFEVCGCRGDWTMENFIDEQVRRIREQVG.GKKVLCALSGGV
GUAA_LISMF              AGFEVTITSKSCPIAGIADEERS....LYGVQFHPEVRHSVYGNELLKNFALNVCGCKGDWTMENFSEVEIAKIQEIVG.DKKVLLALSGGV
GUAA_LISMO              AGFEVTVTSKSCPIAGIADEERS....LYGVQFHPEVRHSVYGNELLKNFALNVCGCKGDWTMENFSEVEIAKIQEIVG.DKKVLLALSGGV
GUAA_LISW6              AGFEVTATSPSCPIAGIADEERL....LYGVQFHPEVRHSVYGNELLKNFALNVCGCKGDWTMENFSEVEIAKIQEIVG.DKKVLLALSGGV
GUAA_LISIN              AGFEVTATSKSCPIAGIADEDRL....LYGVQFHPEVRHSAYGNELLKNFALNICGCKGDWTMENFSEVEIAKIQEIVG.DKKVLLALSGGV
GUAA_EXIS2              NGFVVDGTNVSCPIASIKNEELK....MYGVQYHPEVNHTMFGKELLKNFLFEVCQCTGDWSMENFIEIEIAKIKEEVG.DKKVLCALSGGV
GUAA_STAES              EGFEVIADSPSTNYAAIEDKKRR....IYGVQFHPEVRHTEYGNDLLRNFVRRVCNCTGEWTMENFIEIEIEKIRQQVG.NRKVLCAMSGGV
GUAA_STAEQ              EGFEVIADSPSTNYAAIEDKKRR....IYGVQFHPEVRHTEYGNDLLRNFVRRVCNCTGEWTMENFIEIEIEKIRQQVG.NRKVLCAMSGGV
GUAA_MACCJ              EGFNVIAHSPSCKYAAIENPERN....LYGVQFHPEVRHSEYGNDLLRNFIREICKCTGEWTMENFIEIEIEKIREKVG.DRKVICAMSGGV
GUAA_OCEIH              PSFNIDATSPSTPIAAISNVEKN....LYGVQFHPEVRHTEYGNDLLNRFVFDVCGCTGDWSIENFIDMEVEKIQETVG.DRKVLCALSGGV
GUAA_LACRJ              AGFEVTATSKNCPIASIADNDRK....FYGVQFHPEVRNSEYGLDILRRFAFDVCGAKANWTMDDFIDMQIDEIRKEVG.DKKVILGLSGGV
GUAA_LACRD              AGFEVTATSKNCPIASIADNDRK....FYGVQFHPEVRNSEYGLDILRRFAFDVCGAKANWTMDDFIDMQIDEIRKEVG.DKKVILGLSGGV
GUAA_LACJO              EGFTITAKSKNCPISAIANDEKK....FYGIQFHAEVRNSEYGLDILKNFAFKVCGAKANWTMDDFIEMQVDEIRKKVG.DKKVILGLSGGV
GUAA_LACGA              EGFKVTAKSKNCPISAIANDEKK....FYGIQFHAEVRNSEYGLDILKNFAFNVCDAKANWTMDDFIEMQVDEIRKEVG.DKKVILGLSGGV
GUAA_LACAC              EGFEVTASSKNCPIAAIANPDKK....FYGIQFHAEVRNSEYGLDILKHFAFDVCGAVANWTMADFIDMQVDEIRKEVG.DKKVILGLSGGV
GUAA_LACDB              EGFEVLASSKNCPIASMADPDRK....FYAVQFHAEVRNSEYGLDILRRFAFDVCGAKDNWTMDDFIKLSVDSIREEVG.NANVILGISGGV
GUAA_LACDA              EGFEVLASSKNCPIASMADPDRK....FYAVQFHAEVRNSEYGLDILRRFAFDVCGAKDNWTMDDFIKLSVDSIREEVG.NANVILGISGGV
GUAA_LACCB              EGFRRTATSINCPISAMDDDDRK....FYGIQFHAEVQNTQYGHEILHHFAFDICKAEANWSMDDFITKQIDKIRAEVG.DKRVLLGLSGGV
GUAA_LACC3              EGFRRTATSINCPISAMDDDDRK....FYGIQFHAEVQNTQYGHEILHHFAFDICKAEANWSMDDFITKQIDKIRAEVG.DKRVLLGLSGGV
GUAA_LACRH              DGFRTTATSVNCPISAMDDDDRK....FYGIQFHAEVQNTQYGHEILHHFAFDVCHAEANWSMDDFITKQIAKIRAEVG.DKRVLLGLSGGV
GUAA_LACS1              EGFEVTATSDNCPISAMANDAKK....FYGLQFHTEVRNTEYGNDILRHFAFDVCQARSNWSMDDFIDMQIQKIRETVG.DKKVLLGLSGGV
GUAA_LACLM              EGFHVVGTSPNSPFAAVENTERN....LYGIQFHPEVRHSVHGTEMLRNFALNICGAKGNWSMENFIDMQIKDIREKVG.DKKVLLGLSGGV
GUAA_LACLS              EGFHVVGTSPNSPFAAVENTERN....LYGIQFHPEVRHSVHGTEMLRNFALNTCGAKGNWSMENFIDMQIKDVREKVG.DKKVLLGLSGGV
GUAA_LACLA              EGFHVVGTSPNSPFAAVENTERN....LYGIQFHPEVRHSVHGTEMLRNFALNICGAKGNWSMENFIDMQIKNIREKVG.DKKVLLGLSGGV
GUAA_STRZJ              ADFVRTGTSADCLYAAIENPDKH....IYGIQFHPEVRHSVYGNDILRNFALNICKAKGDWSMDNFIDMQIKKIRETVG.DKRVLLGLSGGV
GUAA_STRPJ              ADFVRTGTSADCLYAAIENPDKH....IYGIQFHPEVRHSVYGNDILRNFALNICKAKGDWSMDNFIDMQIKKIRETVG.DKRVLLGLSGGV
GUAA_STRZT              ADFVRTGTSADCPYAAIENPDKH....IYGIQFHPEVRHSVYGNDILRNFALNICKAKGDWSMDNFIDMQIKKIRETVG.DKRVLLGLSGGV
GUAA_STRR6              ADFVRTGTSADCPYAAIENPDKH....IYGIQFHPEVRHSVYGNDILRNFALNICKAKGDWSMDNFIDMQIKKIRETVG.DKRVLLGLSGGV
GUAA_STRPN              ADFVRTGTSADCPYAAIENPDKH....IYGIQFHPEVRHSVYGNDILRNFALNICKAKGDWSMDNFIDMQIKKIRETVG.DKRVLLGLSGGV
GUAA_STRPI              ADFVRTGTSADCPYAAIENPDKH....IYGIQFHPEVRHSVYGNDILRNFALNICKAKGDWSMDNFIDMQIKKIRETVG.DKRVLLGLSGGV
GUAA_STRP7              ADFVRTGTSADCPYAAIENPDKH....IYGIQFHPEVRHSVYGNDILRNFALNICKAKGDWSMDNFIDMQIKKIRETVG.DKRVLLGLSGGV
GUAA_STRP4              ADFVRTGTSADCPYAAIENPDKH....IYGIQFHPEVRHSVYGNDILRNFALNICKAKGDWSMDNFIDMQIKKIRETVG.DKRVLLGLSGGV
GUAA_STRP2              ADFVRTGTSADCPYAAIENPDKH....IYGIQFHPEVRHSVYGNDILRNFALNICKAKGDWSMDNFIDMQIKKIRETVG.DKRVLLGLSGGV
GUAA_STRZP              ADFVRTGTSADCPYAAIENPDKH....IYGIQFHPEVRHSVYGNDILRNFALNICKAKGDWSMDNFIDMQIKKIRETVG.DKRVLLGLSGGV
GUAA_STRPS              ADFVRTGTSADCPYAAIENPDKH....IYGIQFHPEVRHSVYGNDILRNFALNICKAKGDWSMDNFIDMQIKKIRKTVG.DKRVLLGLSGGV
GUAA_SYMTH              SGFRVLGATENTAVAVMGDDARR....LYGVQFHPEVVHTAQGREILRNFLFNIAGCRGDWTTESFITRQVEEIRRQVG.SGRVLCALSGGV
GUAA_MOOTA              PGFQVTARSAYTPVAAMSDPARK....LYGVQFHPEVKHTPMGQEILRRFLFQVCGCRGDWSVSSFIEDQVAAIRRQVG.NGRVLCALSGGV
GUAA_HELMI              HGFVGSGKTDHAPFAAMADPVRR....FYGVQFHPEVRHTPQGMDMMRNFLFGVCGCTGEWTMENFIEEQVAAIRARVG.SGKVLCALSGGV
GUAA_HALOH              DGFKVTAKTDITPVAAMGNPELK....FYGVQFHPEVVHTIQGLNIIRNYLFKVCGVKGTWNMADFINEEIDRIKEEVG.SSQVISGLSGGV
GUAA_THETN              PDFKVVASTENCPIAAIANVEKK....LYGVQFHPEVSHTHRGTEIIRNFLFEVCDCSADWTMDSLIEQTVKEVRAKVG.NHKAVCALSGGV
GUAA_NATTJ              PGFKVMGKTENTPIAGLFNPDKN....LYGVQFHLEVMHTPKGREMLKNFLFNIADLSADWTMGSYIEETVEQIKTEAQ.GRKAVCGLSGGI
GUAA_MYCTU              DGFDVVASSAGAPVAAFEAFDRR....LAGVQYHPEVMHTPHGQQVLSRFLHDFAGLGAQWTPANIANALIEQVRTQIG.DGHAICGLSGGV
GUAA_MYCTA              DGFDVVASSAGAPVAAFEAFDRR....LAGVQYHPEVMHTPHGQQVLSRFLHDFAGLGAQWTPANIANALIEQVRTQIG.DGHAICGLSGGV
GUAA_MYCBT              DGFDVVASSAGAPVAAFEAFDRR....LAGVQYHPEVMHTPHGQQVLSRFLHDFAGLGAQWTPANIANALIEQVRTQIG.DGHAICGLSGGV
GUAA_MYCBP              DGFDVVASSAGAPVAAFEAFDRR....LAGVQYHPEVMHTPHGQQVLSRFLHDFAGLGAQWTPANIANALIEQVRTQIG.DGHAICGLSGGV
GUAA_MYCBO              DGFDVVASSAGAPVAAFEAFDRR....LAGVQYHPEVMHTPHGQQVLSRFLHDFAGLGAQWTPANIANALIEQVRTQIG.DGHAICGLSGGV
GUAA_MYCTO              DGFDVVASSAGAPVAAFEAFDRR....LAGVQYHPEVMHTPHGQQVLSRFLHDFAGLGAQWTPANIANALIEQVRTQIG.DGHAICGLSGGV
GUAA_MYCMM              DGFEVVASSAGAAVAAFENRERR....LAGVQYHPEVMHTPHGQQILGRFLHDFAGIGARWTPANIANALIEQVRTQIG.DGHAICGLSGGV
GUAA_MYCPA              DGFEVVASSPGAVVAAFENRARR....LAGVQYHPEVMHTPHGQQVLSRFLHDFAGLGADWTAANIAGVLVEQVRAQIG.DGHAICGLSGGV
GUAA_NOSS1              PGFEVLAHTDNTPCAAVADHEKK....LYGVQFHPEVVHSVGGLALIRNFVYHICECEPTWTTAAFVEEAIREVRAKVG.DKRVLLALSGGV
GUAA_ANAVT              PGFEVLAHTDNTPCAAVADHDKK....LYGVQFHPEVVHSIGGLALIRNFVYHICECEPTWTTAAFVEEAIREVRAKVG.DKRVLLALSGGV
GUAA_NOSP7              SGFELLAHTENTPCAAIADHERK....LYGVQFHPEVVHSIGGIALIRNFVYHICDCEPTWTTAAFVEEAIREIRTRVG.EKRVLLALSGGV
GUAA_THEEB              PGFRVLAHTDNTPIAAIAHPERK....LYGVQFHPEVVHSVGGIALIRNFVYHICDCEPTWTTAAFVEEAIREVRAKVG.DKRVLLALSGGV
GUAA_CYAA5              EGFSILAHTENTLCAAIADHDKK....LFGVQFHPEVVHSVDGIALIRNFVYHICECEPTWTTEAFVDETIREIRAKVR.DKRVLLALSGGV
GUAA_CYAP8              EGFDILAHTENTSCAAIAHHQKK....LFGVQFHPEVVHSVGGIALIRNFVYHICQCEPTWTTEAFVEESIREIRAKVG.DKRVLLALSGGV
GUAA_CYAP7              DGFKILAHTDNTPCAAIANHEQK....LFGVQFHPEVVHSVGGIALIRNFVYHICECEPTWTTEAFVEEAIREIRAKIG.DKRVLLALSGGV
GUAA_SYNY3              TGFEILAHTDNTPCAAIADHQKA....LFGVQFHPEVVHSVGGIALIRNFVYHICHCEPTWTTAAFIEESIREVRSQVG.DRRVLLALSGGV
GUAA_SYNP2              EGFEILAHTDNTPCAAIAHHGRQ....LYGVQFHPEVVHSQDGMALIRNFVYHICKCEPTWTTEAFVEEAIREIRARVG.DQRVLLALSGGV
GUAA_SYNP6              EGFELLAHTDNTPCAAIADHQRK....FYGVQFHPEVVHSVHGMALIRNFVYHICDCEPTWTTETFVEEAIREIRARVG.DKRVLLALSGGV
GUAA_GLOVI              EGFELLAHTENTPCAAIAHHSRH....LYGVQFHPEVVHSRGGMALLRNFVYHICGCEPEWTTAAFIEEAIREVRARVG.DKRVLLALSGGV
GUAA_PROMP              EGFIKISHTENTLHAAISNKQKK....LFGVQFHPEVIHSEFGMTVIKNFVYSIAKCKADWTTETFLEETIPRIKQQVG.DKRVLLALSGGV
GUAA_PROM5              NGFIKIAHTENTLHAAISNKEKK....LFGVQFHPEVIHSEYGMTVIKNFVYSISKCKADWNTETFLEETIPRIKEQVG.DKKVLLALSGGV
GUAA_PROMT              KGFVRLAHTSNTLEAAIALHDKS....FYGVQFHPEVVHSTHGMVVIRNFVYDICSCEPDWTTNLFIDEAVSQVQQHVG.DKKVLLALSGGV
GUAA_PROM1              KGFVRLAHTSNTLEAAIALHDKS....FYGVQFHPEVVHSTHGMVVIRNFVYDICSCEPDWTTNLFIDEAVSQVQQQVG.DKKVLLALSGGV
GUAA_THERP              PGFEVLARSANAPYAAMAR..DH....LIGLQFHPEVAHTPLGSVILRNFLFDVCGCAPTWTAESFVEQAIREIRERVG.KDRVLLALSGGV
GUAA_DEIGD              QGYEVIAATEDTPVAGIENTVTR....RYGVQFHPEVVHTPKGGQLLANFL.EICGVARDWNAEHIVDELIEDVRRQVG.AGRVLLAISGGV
GUAA_DEIDV              AGYEVVAETEDTPVTAIENPVSR....RYGVQFHPEVVHTPKGGQLLANFL.KICDVKRDWTAAHIVEELIADVQHQVGDSGRVLLGISGGV
GUAA_DEIRA              QGYQVVARTEHTPVTAIENNDTR....RYGVQFHPEVVHTPKGGQMLANFL.DICGVTRDWNAEHIVDELIEGVRAQVGDTGRVLLGISGGV
GUAA_FUSNN              PDFKIIAHTDSS.IAAIENSDKN....IYAFQYHPEVTHSQHGFDMLKNFIFGIAKAEKNWSMENYIESTVKQIKERVG.NKQVILGLSGGV
GUAA_MESFL              VDFKQIAHSNAS.IAAIAHNTKP....IYGIQFHAEVTHSLQGKEMLENFLYDIAKCNKDWNLDDFIDQQIKEIKETVK.DKQVILGLSGGV
GUAA_SPIKU              TNFIQIAHSENS.ISAIKHSEKK....IYGLQFHPEVTHTLIGQQLLSNFVFNICGCQPKWKITEFISAAITEIKNKVG.TDNVVLALSGGV
GUAA_BORGA              NNFKQLAFTKNC.IASISNEDQK....IYGLQFHPEVTHSEFGDQILKNFVFKICQSQTNWSLESNVETIVEKIKLKVG.SKKVILGLSGGT
GUAA_SULDN              AGFEPIAVSSNSPYAAIANEEKR....IYAMQYHPEVQHSEEGYLMLRNFARNICGVTEKWKMEHFLKEQIKNIRAKVG.SGKVLCGLSGGV
GUAA_WOLSU              EGFTRIAHSTNSPFAAIAHQEKP....FYAVQFHPEVVHSEEGALYLKNFALGICGCENDWNMHNFAEEQIKKIQERVK.SGKVLCAVSGGV
GUAA_SULNB              EGFEVVGTSENSPYAAIANESKH....IYAFQFHPEVHHSVEGTHMLKNFAKYICGCESTWNMGSFAKEKIAQIRAQVG.EKKVLCGVSGGV
GUAA_ARCB4              AGFEKIATSENSPFAAIADINRN....IYAFQFHPEVYHSQEGSKLLKNFAKHICGCESTWNMGSFAKEQIKQIQDKVG.NKKVLCAVSGGV
GUAA_NITSB              AGFIRIAHTDNSPYAAIANEEKQ....IYALQFHPEVSHSQEGQKILENFARKICGITSKWDMGHFAKEQIEKIRQRVG.EDKVLCALSGGV
GUAA_CAMHC              DGFKTIAVSENSPFCAFCDEKRK....IYALQFHPEVAHSECGDKILKNFAKYICDCESTWNMGNFAKIKCEEIKKQVG.KDRVLCAVSGGV
GUAA_THESQ              EGFHPIAVSETGVIAAATD.GKR....FWLLQFHPEVHHTEYGDRMISNFLFNVCKLEKNWKIGDLVEEKIRHIKETIG.NKKAILALSGGV
GUAA_THEMA              EGFHPIAVSETGVIAAATD.GKR....FWLLQFHPEVHHTEYGDRMISNFLFNVCKLEKNWKIGDLVEEKIRHIKETIG.NKKAILALSGGV
GUAA_THEP1              EGFHPIAVSETGVIAAATD.GKR....FWLLQFHPEVHHTEYGDRMISNFLFNVCKLEKNWKIGDLVEEKIRHIKETIG.NKKAILALSGGV
GUAA_PETMO              EKFKITSLTSNNIISSFENESEN....IYCIQFHPEVRHTEFGIDILKNFIQGICGLKGSWTLMDFVENKIKEIKDTIG.DKKAIIALSGGV
GUAA_AQUAE              EGFEVLAVSENSPYAVIANREKK....IYGFQFHPEVTHTVFGKEMLANFIYGVCKAEKNWEMGDFIHEKIEEIRKTVG.DAKVIAALSGGV
GUAA_THEAB              EGFLVIGESENSPYAAIRNKDGT....IYGVQFHPEVTHTSFGDKILENFVSKVAKMEKNWKMTDFIEEKINEIRKVVG.NDKVILGLSGGV
GUAA_THEM4              EGFYVIGRSENSPFAAIRNKNET....IYGVQFHPEVTHTQFGSKILENFVSKIAKMEKNWEMKDFVSEKIKEIKGIVG.NDKVILGLSGGV
GUAA_PELUB              KNFKIIASTKNSKLTIIENIKEN....FYGVQFHPEVTHTNKGKILLRNFVFLICKIKKNWSSKDQKLKLINEIKDQVG.DNKVICGLSGGV
GUAA_MYCPE              KGFKVIASTNKCLVASACNREKK....FYGVQFHPEVTQTTFGNQLLQNFVYSICDVAGDWEPKNIKLEKIEEIKSIVK.DDYVLCAISGGV
tr|Q7RIY7|Q7RIY7_PLAYO  ENYYLVNSSENCFICAFYNKENN....IYGVQYHPEVYETVDGDQMFYNFAYKICKCTKTFDPIKYHEVEFNNIKKHAH.DHYVIAAMSGGI
tr|Q4Z1X2|Q4Z1X2_PLABA  ENYYLVNSSENCFICAFYNKENN....IYGVQYHPEVYETADGDQMFYNFAYKICKCTKTFDPIKYHEVEFNNIKKHAH.DHYVIAAMSGGI
tr|Q4Y2M7|Q4Y2M7_PLACH  ............................................................................................
tr|B3L3R6|B3L3R6_PLAKH  ENFFLVNSTDDCLICAMYNEEYN....IYGVQYHPEVYESLDGEQMFYNFAYKICKCTKKFDPIRYHEIELNNIKKYAK.DHYVIAAMSGGI
tr|A5K7F1|A5K7F1_PLAVS  DNFFLVNSTDDCLICAMYNEEHN....IYGVQYHPEVYESVDGDQMFYNFAYNICECTKKFDPIRYHEIELNNIKKYAQ.DHYVIAAMSGGI
tr|Q8IJR9|Q8IJR9_PLAF7  ENFYLVSSSENCLICSIYNKEYN....IYGVQYHPEVYESLDGELMFYNFAYNICKCKKQFDPIRYHELELKNIEKYKH.DHYVIAAMSGGI
```

```
                 240       250       260       270        280       290       300        310       320
GUAA_ECO55  DSSVTAMLLHRAIG.KNLTCVFVDNGLLRLNEAEQVLDMFG..DHFGLNIVHVPAEDRFLSALAGEND.PEAKRKIIGRVFVEVFDEEALKL
GUAA_ECO45  DSSVTAMLLHRAIG.KNLTCVFVDNGLLRLNEAEQVLDMFG..DHFGLNIVHVPAEDRFLSALAGEND.PEAKRKIIGRVFVEVFDEEALKL
GUAA_ECO57  DSSVTAMLLHRAIG.KNLTCVFVDNGLLRLNEAEQVLDMFG..DHFGLNIVHVPAEDRFLSALAGEND.PEAKRKIIGRVFVEVFDEEALKL
GUAA_ECO7I  DSSVTAMLLHRAIG.KNLTCVFVDNGLLRLNEAEQVLDMFG..DHFGLNIVHVPAEDRFLSALAGEND.PEAKRKIIGRVFVEVFDEEALKL
GUAA_ECO5E  DSSVTAMLLHRAIG.KNLTCVFVDNGLLRLNEAEQVLDMFG..DHFGLNIVHVPAEDRFLSALAGEND.PEAKRKIIGRVFVEVFDEEALKL
GUAA_ECO81  DSSVTAMLLHRAIG.KNLTCVFVDNGLLRLNEAEQVLDMFG..DHFGLNIVHVPAEDRFLSALAGEND.PEAKRKIIGRVFVEVFDEEALKL
GUAA_ECO8A  DSSVTAMLLHRAIG.KNLTCVFVDNGLLRLNEAEQVLDMFG..DHFGLNIVHVPAEDRFLSALAGEND.PEAKRKIIGRVFVEVFDEEALKL
GUAA_ECOHS  DSSVTAMLLHRAIG.KNLTCVFVDNGLLRLNEAEQVLDMFG..DHFGLNIVHVPAEDRFLSALAGEND.PEAKRKIIGRVFVEVFDEEALKL
GUAA_ECOK1  DSSVTAMLLHRAIG.KNLTCVFVDNGLLRLNEAEQVLDMFG..DHFGLNIVHVPAEDRFLSALAGEND.PEAKRKIIGRVFVEVFDEEALKL
GUAA_ECOL5  DSSVTAMLLHRAIG.KNLTCVFVDNGLLRLNEAEQVLDMFG..DHFGLNIVHVPAEDRFLSALAGEND.PEAKRKIIGRVFVEVFDEEALKL
GUAA_ECOL6  DSSVTAMLLHRAIG.KNLTCVFVDNGLLRLNEAEQVLDMFG..DHFGLNIVHVPAEDRFLSALAGEND.PEAKRKIIGRVFVEVFDEEALKL
GUAA_ECOLC  DSSVTAMLLHRAIG.KNLTCVFVDNGLLRLNEAEQVLDMFG..DHFGLNIVHVPAEDRFLSALAGEND.PEAKRKIIGRVFVEVFDEEALKL
GUAA_ECOSE  DSSVTAMLLHRAIG.KNLTCVFVDNGLLRLNEAEQVLDMFG..DHFGLNIVHVPAEDRFLSALAGEND.PEAKRKIIGRVFVEVFDEEALKL
GUAA_ECOSM  DSSVTAMLLHRAIG.KNLTCVFVDNGLLRLNEAEQVLDMFG..DHFGLNIVHVPAEDRFLSALAGEND.PEAKRKIIGRVFVEVFDEEALKL
GUAA_ECOUT  DSSVTAMLLHRAIG.KNLTCVFVDNGLLRLNEAEQVLDMFG..DHFGLNIVHVPAEDRFLSALAGEND.PEAKRKIIGRVFVEVFDEEALKL
GUAA_ECO24  DSSVTAMLLHRAIG.KNLTCVFVDNGLLRLSEAEQVLDMFG..DHFGLNIVHVPAEDRFLSALAGEND.PEAKRKIIGRVFVEVFDEEALKL
GUAA_SHISS  DSSVTAMLLHRAIG.KNLTCVFVDNGLLRLNEAEQVLDMFG..DHFGLNIVHVPAEDRFLSALAGEND.PEAKRKIIGRVFVEVFDEEALKL
GUAA_SHIF8  DSSVTAMLLHRAIG.KNLTCVFVDNGLLRLNEAEQVLDMFG..DHFGLNIVHVPAEDRFLSALAGEND.PEAKRKIIGRVFVEVFDEEALKL
GUAA_SHIFL  DSSVTAMLLHRAIG.KNLTCVFVDNGLLRLNEAEQVLDMFG..DHFGLNIVHVPAEDRFLSALAGEND.PEAKRKIIGRVFVEVFDEEALKL
GUAA_SHIDS  DSSVTAMLLHRAIG.KNLTCVFVDNGLLRLNEAEQVLDMFG..DHFGLNIVHVPAEDRFLSALAGEND.PEAKRKIIGRVFVEVFDEEALKL
GUAA_ECOLI  DSSVTAMLLHRAIG.KNLTCVFVDNGLLRLNEAEQVLDMFG..DHFGLNIVHVPAEDRFLSALAGEND.PEAKRKIIGRVFVEVFDEEALKL
GUAA_ECODH  DSSVTAMLLHRAIG.KNLTCVFVDNGLLRLNEAEQVLDMFG..DHFGLNIVHVPAEDRFLSALAGEND.PEAKRKIIGRVFVEVFDEEALKL
GUAA_ECOBW  DSSVTAMLLHRAIG.KNLTCVFVDNGLLRLNEAEQVLDMFG..DHFGLNIVHVPAEDRFLSALAGEND.PEAKRKIIGRVFVEVFDEEALKL
GUAA_ESCF3  DSSVTAMLLHRAIG.KNLTCVFVDNGLLRLNEAEQVLDMFG..DHFGLNIVHVPAEDRFLSALAGEND.PEAKRKIIGRVFVEVFDEEALKL
GUAA_ECO27  DSSVTAMLLHRAIG.KNLTCVFVDNGLLRLNEAEQVLDMFG..DHFGLNIVHVPAEDRFLSALAGEND.PEAKRKIIGRVFVEVFDEEALKL
GUAA_SHIB3  DSSVTAMLLHRAIG.KNLTCVFVDNGLLRLNEAEQVLDMFG..DHFGLNIVHVPAEDRFLSALAGEND.PEAKRKIIGRVFVEVFDEEALKL
GUAA_SHIBS  DSSVTAMLLHRAIG.KNLTCVFVDNGLLRLNEAEQVLDMFG..DHFGLNIVHVPAEDRFLSALAGEND.PEAKRKIIGRVFVEVFDEEALKL
GUAA_ECOLU  DSSVTAMLLHRAIG.KNLTCVFVDNGLLRLNEAEQVLDMFG..DHFGLNIVHVPAEARFLSALAGEND.PEAKRKIIGRVFVEVFDEEALKL
GUAA_SALPK  DSSVTAMLLHRAIG.KNLTCVFVDNGLLRLNEAEQVMDMFG..DHFGLNIVHVPAEERFLSALAGEND.PEAKRKIIGRVFVEVFDEEALKL
GUAA_SALPC  DSSVTAMLLHRAIG.KNLTCVFVDNGLLRLNEAEQVMDMFG..DHFGLNIVHVPAEERFLSALAGEND.PEAKRKIIGRVFVEVFDEEALKL
GUAA_SALPB  DSSVTAMLLHRAIG.KNLTCVFVDNGLLRLNEAEQVMDMFG..DHFGLNIVHVPAEERFLSALAGEND.PEAKRKIIGRVFVEVFDEEALKL
GUAA_SALPA  DSSVTAMLLHRAIG.KNLTCVFVDNGLLRLNEAEQVMDMFG..DHFGLNIVHVPAEERFLSALAGEND.PEAKRKIIGRVFVEVFDEEALKL
GUAA_SALCH  DSSVTAMLLHRAIG.KNLTCVFVDNGLLRLNEAEQVMDMFG..DHFGLNIVHVPAEERFLSALAGEND.PEAKRKIIGRVFVEVFDEEALKL
GUAA_SALTY  DSSVTAMLLHRAIG.KNLTCVFVDNGLLRLNEAEQVMDMFG..DHFGLNIVHVPAEERFLSALAGEND.PEAKRKIIGRVFVEVFDEEALKL
GUAA_SALSV  DSSVTAMLLHCAIG.KNLTCVFVDNGLLRLNEAEQVMDMFG..DHFGLNIVHVPAEERFLSALAGEND.PEAKRKIIGRVFVEVFDEEALKL
GUAA_SALNS  DSSVTAMLLHRAIG.KNLTCVFVDNGLLRLNEAEQVMDMFG..DHFGLNIVHVPAEERFLSALAGEND.PEAKRKIIGRVFVEVFDEEALKL
GUAA_SALTI  DSSVTAMLLHRAIG.KNLTCVFVDNGLLRLNEAEQVMDMFG..DHFGLNIVHVPAEDRFLSALAGEND.PEAKRKIIGRVFVEVFDEEALKL
GUAA_SALHS  DSSVTAMLLHRAIG.KNLTCVFVDNGLLRLNEAEQVMDMFG..DHFGLNIVHVPAEDRFLSALAGEND.PEAKRKIIGRVFVEVFDEEALKL
GUAA_SALG2  DSSVTAMLLHRAIG.KNLTCVFVDNGLLRLNEAEQVMDMFG..DHFGLNIVHVPAEDRFLSALAGEND.PEAKRKIIGRVFVEVFDEEALKL
GUAA_SALEP  DSSVTAMLLHRAIG.KNLTCVFVDNGLLRLNEAEQVMDMFG..DHFGLNIVHVPAEDRFLSALAGEND.PEAKRKIIGRVFVEVFDEEALKL
GUAA_SALDC  DSSVTAMLLHRAIG.KNLTCVFVDNGLLRLNEAEQVMDMFG..DHFGLNIVHVPAEDRFLSALAGEND.PEAKRKIIGRVFVEVFDEEALKL
GUAA_SALA4  DSSVTAMLLHRAIG.KNLTCVFVDNGLLRLNEAEQVMDMFG..DHFGLNIVHVPAEDRFLSALAGEND.PEAKRKIIGRVFVEVFDEEALKL
GUAA_SALAR  DSSVTAMLLHRAIG.KNLTCVFVDNGLLRLNEAEQVMDMFG..DHFGLNIVHVPAEDRFLSALAGEND.PEAKRKIIGRVFVEVFDEEALKL
GUAA_KLEP7  DSSVTAMLLHRAIG.KNLTCVFVDNGLLRLNEAQQVMEMFG..DHFGLNIVHVEGEQRFLDALAGESD.PEAKRKIIGRVFVEVFDEEALKL
GUAA_KLEP3  DSSVTAMLLHRAIG.KNLTCVFVDNGLLRLNEAQQVMDMFG..DHFGLNIVHVEGEQRFLDALKGEND.PEAKRKIIGRVFVEVFDEEALKL
GUAA_ENT38  DSSVTAMLLHRAIG.KNLTCVFVDNGLLRLNEAEQVMDMFG..DHFGLNIVHVEGEERFLTALAGEND.PEAKRKIIGRVFVEVFDEQALRL
GUAA_CITK8  DSSVTAMLLHRAIG.KNLTCVFVDNGLLRLNEAEQVMDMFG..DHFGLNIVHVPAEERFLTALKGEND.PEAKRKIIGRVFVEVFDEEAFRL
GUAA_CROS8  DSSVTAMLLHRAIG.DRLTCVFVDNGLLRLNEAEQVMDMFG..DHFGLNIVHVPAEARFLDALAGIDE.PEAKRKTIGRVFVEVFDEEALKL
GUAA_SODGM  DSSVTAMLLHRAIG.ERLTCVFVDNGLLRLNEASQVMEMFG..DHYGLNIIAVPAEDRFLSALAGIDD.PETKRKTIGRVFVEVFDEQALSL
GUAA_PECCP  DSSVTAMLLHRAIG.DRLTCVFVDNGLLRLNEADQVLEMFG..DHFGLNIVHVAAEDRFLGELAGEND.PEAKRKIIGRVFVEVFDEEASKQ
GUAA_PECAS  DSSVTAMLLHRAIG.DRLTCVFVDNGLLRLNEADQVLEMFG..DNFGLNIVHVAAEDRFLNELAGEDE.PEAKRKIIGRVFVEVFDEEANKQ
GUAA_YERPY  DSSVTAMLLHRAIG.KRLTCVFVDNGLLRLNEADQVLEMFG..DKFGLNIVHVAAEDRFLSALAGVDE.PEAKRKIIGRVFVELFDEEACKQ
GUAA_YERPS  DSSVTAMLLHRAIG.KRLTCVFVDNGLLRLNEADQVLEMFG..DKFGLNIVHVAAEDRFLSALAGVDE.PEAKRKIIGRVFVELFDEEACKQ
GUAA_YERPB  DSSVTAMLLHRAIG.KRLTCVFVDNGLLRLNEADQVLEMFG..DKFGLNIVHVAAEDRFLSALAGVDE.PEAKRKIIGRVFVELFDEEACKQ
GUAA_YERP3  DSSVTAMLLHRAIG.KRLTCVFVDNGLLRLNEADQVLEMFG..DKFGLNIVHVAAEDRFLSALAGVDE.PEAKRKIIGRVFVELFDEEACKQ
GUAA_YERPP  DSSVTAMLLHRAIG.KRLTCVFVDNGLLRLNEADQVLEMFG..DKFGLNIVHVAAEDRFLSALTGVDE.PEAKRKIIGRVFVELFDEEACKQ
GUAA_YERPN  DSSVTAMLLHRAIG.KRLTCVFVDNGLLRLNEADQVLEMFG..DKFGLNIVHVAAEDRFLSALTGVDE.PEAKRKIIGRVFVELFDEEACKQ
GUAA_YERPG  DSSVTAMLLHRAIG.KRLTCVFVDNGLLRLNEADQVLEMFG..DKFGLNIVHVAAEDRFLSALTGVDE.PEAKRKIIGRVFVELFDEEACKQ
GUAA_YERPE  DSSVTAMLLHRAIG.KRLTCVFVDNGLLRLNEADQVLEMFG..DKFGLNIVHVAAEDRFLSALTGVDE.PEAKRKIIGRVFVELFDEEACKQ
GUAA_YERPA  DSSVTAMLLHRAIG.KRLTCVFVDNGLLRLNEADQVLEMFG..DKFGLNIVHVAAEDRFLSALTGVDE.PEAKRKIIGRVFVELFDEEACKQ
GUAA_YERE8  DSSVTAMLLHRAIG.KRLTCVFVDNGLLRLNEADQVLEMFG..DKFGLNIVHVAAEDRFLAALAGVDE.PEAKRKIIGRVFVELFDEEACKQ
GUAA_SERP5  DSSVTAMLLHRAIG.KRLTCVFVDNGLLRLNEAEQVLEMFG..DHFGLNIVHVAAEDRFLSALAGVDE.PEAKRKIIGRVFVELFDEEACKQ
GUAA_PHOLL  DSSVTALLLHRAIG.NRLTCVFVDNGLLRLNEATQVMEMFA..GKFGLNIVHVPAEDRFLTALAGIND.PEEKRKTIGRVFVEVFDEEASKQ
GUAA_PROMH  DSSVTALLLNRAIG.KRLTCVFVDNGLLRLNEAEQVMAMFK..GKFDLNIIHVEAEDRFLTALKGEND.PEKKRKIIGHTFIEIFDEEAVKL
GUAA_EDWI9  DSSVTALLLHRAIG.KRLTCVFVDNGLLRLNEAEQVMDMFG..DRFGLNIIHVNAEARFLSALSGIAD.PEAKRKTIGRVFVEVFDEEAAKL
GUAA_AERHH  DSSVVAMLVHRAIG.DRLTCVFVDNGLLRLNEGQQVMEMFG..DHFGLNIVKVDAEERFLSALAGEDE.PEAKRKIIGRVFVEVFDDEAKKL
GUAA_AERS4  DSSVVAMLVHRAIG.DRLTCVFVDNGLLRLNEGEQVMEMFG..DHFGLNIIKVEAEERFLSALAGEDE.PEAKRKIIGRVFVEVFDDEAKKL
GUAA_TOLAT  DSSVVAMLIHRAVG.KQLTCVFVDNGLLRLNEGQQVMEMFG..DHFGLNIIKVDAEERFLSALKGIAD.PELKRKAIGRVFVEVFDDESKKL
GUAA_VIBF1  DSSVVAMLAHRAIG.DKLTCVFVDNGLLRLNEADQVMEMFG..NKFGLNIVHVNAEQRFLDALEGESD.PETKRKIIGHVFVDIFDEESKKL
GUAA_VIBFM  DSSVVAMLAHRAIG.DKLTCVFVDNGLLRLNEADQVMEMFG..NKFGLNIIHVNAEQRFLDALEGESD.PEVKRKIIGHVFVDIFDEESKKL
GUAA_ALISL  DSSVVAMLAHRAIG.DKLTCVFVDNGLLRLNEAEQVMEMFS..EGFGLKIIHVEAEERFLSALSGESD.PEAKRKIIGHVFVDIFDEESKKL
GUAA_VIBSL  DSSVVAMLAHRAIG.DKLTCVFVDNGLLRLNEGEQVMEMFG..DQFGLNIIKVDAEDRFLDALEGEAE.PEAKRKIIGHVFVDIFDEESKKL
GUAA_VIBCM  DSSVVAMLVHRAIG.SKLTCVFVDNGLLRLNEGEQVMEMFG..DKFGLNIIKVDAEERFLKALEGIDE.PEAKRKTIGRVFVEVFDEESKKL
GUAA_VIBCH  DSSVVAMLVHRAIG.SKLTCVFVDNGLLRLNEGEQVMEMFG..DKFGLNIIKVDAEERFLKALEGIDE.PEAKRKTIGRVFVEVFDEESKKL
GUAA_VIBC3  DSSVVAMLVHRAIG.SKLTCVFVDNGLLRLNEGEQVMEMFG..DKFGLNIIKVDAEERFLKALEGIDE.PEAKRKTIGRVFVEVFDEESKKL
GUAA_VIBVU  DSSVVAMLVHRAIG.DKLTCVFVDNGLLRLNEGQQVMDMFG..DKFGLNIIKVDAEERFLKALEGKSD.PEEKRKTIGHVFVDVFDEESKKL
GUAA_VIBVY  DSSVVAMLVHRAIG.DKLTCVFVDNGLLRLNEGQQVMDMFG..DKFGLNIIKVDAEERFLKALEGKSD.PEEKRKTIGHVFVDVFDEESKKL
GUAA_VIBPA  DSSVVAMLVHRAIG.DKLTCVFVDNGLLRLNEGQQVMDMFG..DKFGLNIIKVDAEERFLEALKGKSD.PEEKRKTIGHVFVDVFDEESKKL
GUAA_VIBCB  DSSVVAMLVHRAIG.DKLTCVFVDNGLLRLNEGQQVMDMFG..DQFGLNIIKVDAEDRFLKALEGKSD.PEEKRKTIGHVFVDVFDEESKKL
GUAA_PHOPR  DSSVVAMLLHRAIG.DKLTCVFVDNGLLRLNEGEQVMDMFG..DHFGLNIVHVKAEDRFLNALAGEND.PEAKRKIIGHAFIDVFDEESKKL
GUAA_COLP3  DSSVVAMLLHRAIG.DKLTCVFVDNGLLRLDEGQQVMDMFG..DHFGLNIIKIEAEDRFLNRLAGESE.PEAKRKIIGNVFIDVFEEESNKL
GUAA_PSYIN  DSSVVAMLLQRAIG.DKLTCVFVDNGLLRLNEGQQVMDMFG..DHFGLKIIHVNAEHRFLEQMAGESD.PEAKRKIIGRVFVEIFDEESKKL
GUAA_PSEHT  DSSVVAMLIHRAIG.EKLTCVFVDNGLLRLNEGQQVMDMFG..NKFGLNIVKVEAEEQFLNDLAGKSD.PEDKRKAIGHTFINVFDEQAKKL
GUAA_PSEA6  DSSVVAMLLHRAIG.KNLTCVFVDNGLLRLNEGQQVMDMFG..DHFGLNIIKIDAEERFLSALAGTDE.PEAKRKIIGRVFVEVFDEESKKL
GUAA_ALTMD  DSSVTAMLLHRAIG.KNLTCVFVDNGLLRLNEADQVMEMFG..DHFGLNIIRVDAEGRFLDRLKGIED.PELKRKAIGNEFVRVFDEEASKL
GUAA_SHESW  DSSVVAMLLHRAIG.KKLTCVFVDNGLLRLNEAAQVMEMFG..DHFGLNIIHVDAENRFLDAMKGVAD.PEAKRKIIGRVFVEIFDEESKKC
GUAA_SHEPC  DSSVVAMLLHRAIG.KKLTCVFVDNGLLRLNEAAQVMEMFG..DHFGLNIIHVDAENRFLDAMKGVAD.PEAKRKIIGRVFVEIFDEESKKC
GUAA_SHEB9  DSSVVAMLLHRAIG.KKLTCVFVDNGLLRLNEAEQVMEMFG..DHFGLNIIHVDAENRFLDAMKGEAD.PEAKRKIIGRVFVEIFDEESKKC
GUAA_SHEB8  DSSVVAMLLHRAIG.KKLTCVFVDNGLLRLNEAEQVMEMFG..DHFGLNIIHVDAENRFLDAMKGEAD.PEAKRKIIGRVFVEIFDEESKKC
GUAA_SHEB5  DSSVVAMLLHRAIG.KKLTCVFVDNGLLRLNEAEQVMEMFG..DHFGLNIIHVDAENRFLDAMKGEAD.PEAKRKIIGRVFVEIFDEESKKC
GUAA_SHEB2  DSSVVAMLLHRAIG.KKLTCVFVDNGLLRLNEAEQVMEMFG..DHFGLNIIHVDAENRFLDAMKGEAD.PEAKRKIIGRVFVEIFDEESKKC
GUAA_SHESM  DSSVVAMLLHRAIG.KKLTCVFVDNGLLRLNEAKQVMEMFG..DHFGLNIVHVDAENRFLDALKGEAD.PEAKRKIIGRVFVEIFDEEAKKC
GUAA_SHESA  DSSVVAMLLHRAIG.KKLTCVFVDNGLLRLNEAKQVMEMFG..DHFGLNIVHVDAENRFLDALKGEAD.PEAKRKIIGRVFVEIFDEEAKKC
GUAA_SHESR  DSSVVAMLLHRAIG.KKLTCVFVDNGLLRLNEAKQVMEMFG..DHFGLNIVHIDAENRFLDALKGEAD.PEAKRKIIGRVFVEIFDEEAKKC
GUAA_SHEON  DSSVVAMLLHRAIG.KKLTCVFVDNGLLRLNEAKQVMEMFG..DHFGLNIVHVDAENRFLDALKGEAD.PEAKRKIIGRVFVEIFDEEAKKC
GUAA_SHEDO  DSSVVAMLLHRAIG.KKLTCVFVDNGLLRLNEAEQVLEMFG..DHFGLNIVHVDAENRFLDAMAGVAD.PEAKRKIIGRVFVEIFDEESKKC
GUAA_SHEPW  DSSVVAMLLHRAIG.DKLTCVFVDNGLLRLNEAQQVMDMFG..DHFGLNIIHVDAENRFLDAMAGEAD.PESKRKIIGHVFVEIFDEESKKC
GUAA_SHESH  DSSVVAMLLHRAIG.DKLTCVFVDNGLLRLNEADQVMDMFG..DHFGLNIVHVNAENRFLDAMKGEAD.PEAKRKIIGHVFVEIFDEESKKC
GUAA_SHEWM  DSSVVAMLLHRAIG.DKLTCVFVDNGLLRLNEAQQVMDMFG..DHFGLNIVHVDAENRFLDAMAGEAD.PEAKRKIIGHVFVDIFDEESKKC
GUAA_SHEHH  DSSVVAMLLHRAIG.DKLTCVFVDNGLLRLNEAEQVMEMFG..DHFGLNIVHVDAENRFLDAMAGEAE.PEAKRKIIGRVFVEIFDEESKKC
GUAA_SHEPA  DSSVVAMLLHRAIG.DKLTCVFVDNGLLRLNEAQQVMDMFG..DHFGLNIVHVDAENRFLDAMAGEAE.PEAKRKIIGRVFVEIFDEESKKC
GUAA_SHEFN  DSSVVAMLLHRAIG.DKLTCVFVDNGLLRLNEAEQVLEMFG..DHFGLNIVHVDAENRFLDAMAGEAE.PEAKRKIIGRVFVEIFDEESKKC
GUAA_SHELP  DSSVVAMLLHRAIG.SKLTCVFVDNGLLRLNEADQVLEMFG..DHFGLNIVHVDAESRFLDALAGEAE.PEAKRKIIGKVFVDIFDEESRKC
GUAA_SHEAM  DSSVTAMLLHRAIG.ERLTCVFVDNGLLRLNEAKQVLEMFG..DHFGLNIVHVDAENRFLEALKGEND.PEAKRKIIGRVFVEIFDEEASKC
GUAA_IDILO  DSSVTAMLLHRAIG.EQLTCVFVDNGLLRLNEAEQVMEMFG..DHYGLNILPIRAEDRFLDALAGEND.PEKKRKIIGNTFIEIFDEEAGKL
GUAA_ACTSZ  DSSVTALLLHRAIG.KNLHCVFVDNGLLRLNEGDQVMEMFG..DKFGLNIIRVNAEERFLDALKGIHD.PEAKRKMIGKVFVDVFDEESHKQ
GUAA_MANSM  DSSVTALLLHRAIG.KNLHCVFVDNGLLRLNEGDQVMEMFG..DKFGLNIIRVNAEDRFLDALKGIDE.PESKRKMIGKVFVDVFDEESHKQ
GUAA_ACTPJ  DSSVTALLLHRAIG.KNLHCVFVDNGLLRLNEGDQVMEMFG..DKFGLNIIRVNAEDRFLDALKGIDE.PEAKRKTIGKVFVDVFDDESKKL
GUAA_ACTP7  DSSVTALLLHRAIG.KNLHCVFVDNGLLRLNEGDQVMEMFG..DKFGLNIIRVNAEDRFLDALKGIDE.PEAKRKTIGKVFVDVFDDESKKL
GUAA_ACTP2  DSSVTALLLHRAIG.KNLHCVFVDNGLLRLNEGDQVMEMFG..DKFGLNIIRVNAEDRFLDALKGIDE.PEAKRKTIGKVFVDVFDDESKKL
GUAA_HAEPS  DSSVTALLLHRAIG.KNLHCVFVDNGLLRLNEGDQVMEMFG..DKFGLNIIRVNAEDRFLDALKGIDE.PEAKRKTIGKVFVDVFDDESKKL
GUAA_PASMU  DSSVTALLLHRAIG.KNLHCVFVDNGLLRLNEAEQVMEMFG..DKFGLNIIHVKAEDRFLDALKGIDE.PEAKRKMIGKVFVDVFDDESKKL
GUAA_HAEDU  DSSVTALLLHRAIG.KNLHCVFVDNGLLRLNEGDQVMEMFG..DKFGLNIIRVNAEERFLEALKGIDE.PEAKRKIIGKVFIDIFDDEAKKL
GUAA_HISS1  DSSVTALLLHRAIG.KNLHCVFVDNGLLRLNEGDQVMEMFG..DKFGLNIIRVEAEDRFLEALKGIDE.PEAKRKTIGKVFVDVFDDEAKKL
GUAA_HISS2  DSSVTALLLHRAIG.KNLHCVFVDNGLLRLNEGDQVMEMFG..DKFGLNIIRVEAEDRFLEALKGIDE.PEAKRKTIGKVFVDVFDDEAKKL
GUAA_HAEI8  DSSVVALLLHRAIG.KNLHCVFVDNGLLRLHEGDQVMEMFG..DKFGLNITRVDAESRFLGELAGVSD.PEAKRKIIGKVFVDVFDDESKKL
GUAA_HAEIG  DSSVVALLLHRAIG.KNLHCVFVDNGLLRLHEGDQVMEMFG..DKFGLNITRVDAESRFLGELAGVSD.PEAKRKIIGKVFVDVFDDESKKL
GUAA_HAEIN  DSSVVALLLHRAIG.KNLHCVFVDNGLLRLHEGDQVMEMFG..DKFGLNITRVDAESRFLGELAGVSD.PEAKRKIIGKVFVDVFDDESKKL
GUAA_HAEIE  DSSVVALLLHRAIG.KNLHCVFVDNGLLRLHEGDQVMEMFG..DKFGLNITRVDAESRFLGELAGVSD.PEAKRKIIGKVFVDVFDDESKKL
GUAA_HAMD5  DSAVTAILLHRAIG.KSLTCVFVDNGLLRLNESQQVLNIFQ..DQFGLNIIHVSAANRFMNALKGIQD.PEKKRKVIGRVFVDIFDEQAAKQ
GUAA_XANC5  DSSVVAALLHKAIG.DKLTCVFVDTGLLRWQEGDQVMAMFA..EHMGVKVIRVNAADRYFAKLEGVSD.PEAKRKIIGNLFVEIFDEESNKL
GUAA_XANAC  DSSVVAALLHKAIG.DKLTCVFVDTGLLRWQEGDQVMAMFA..EHMGVKVIRVNAADRYFAKLEGVRD.PEAKRKIIGNLFVEIFDEESNKL
GUAA_XANCP  DSSVVAALLHKAIG.DKLTCVFVDTGLLRWQEGDQVMAMFA..EHMGVKVIRVNAADRYFAKLEGVSD.PEAKRKIIGNLFVDIFDEESNKL
GUAA_XANCB  DSSVVAALLHKAIG.DKLTCVFVDTGLLRWQEGDQVMAMFA..EHMGVKVIRVNAADRYFAKLEGVSD.PEAKRKIIGNLFVDIFDEESNKL
GUAA_XANC8  DSSVVAALLHKAIG.DKLTCVFVDTGLLRWQEGDQVMAMFA..EHMGVKVIRVNAADRYFAKLEGVSD.PEAKRKIIGNLFVDIFDEESNKL
GUAA_XANOR  DSSVVAALLHKAIG.DKLTCVFVDTGLLRWQEGDQVMAMFA..EHMGVKVIRVNAADRYFAKLEGVSD.PEAKRKIIGNLFVEIFDEESNTL
GUAA_XANOM  DSSVVAALLHKAIG.DKLTCVFVDTGLLRWQEGDQVMAMFA..EHMGVKVIRVNAADRYFAKLEGVSD.PEAKRKIIGNLFVEIFDEESNTL
GUAA_XANOP  DSSVVAALLHKAIG.DKLTCVFVDTGLLRWQEGDQVMAMFA..EHMGVKVIRVNAADRYFAKLEGVSD.PEAKRKIIGNLFVEIFDEESNTL
GUAA_STRMK  DSSVVAALLHKAIG.EKLTCVFVDTGLLRWQEGDQVMAMFA..EHMGVKVVRVNAADRYFAALEGVSD.PEAKRKIIGNLFVEIFDEESNKL
GUAA_STRM5  DSSVVAALLHKAIG.EKLTCVFVDTGLLRWQEGDQVMAMFA..EHMGVKVVRVNAADRYFAALEGVSD.PEAKRKIIGNLFVEIFDEESNKL
GUAA_XYLFT  DSSVVAALLHKAIG.SQLTCVFVDTGMLRWGEGDQVMAMFA..EHMGVNVVRINAASRYFDALQGVYD.PEAKRKIIGNLFIQIFEEEASKR
GUAA_XYLF2  DSSVVAALLHKAIG.SQLTCVFVDTGMLRWGEGDQVMAMFA..EHMGVNVVRINAASRYFDALQGVYD.PEAKRKIIGNLFIQIFEEEASKR
GUAA_XYLFM  DSSVVAALLHKAIG.SQLTCVFVDTGLLRWGEGDQVMAMFE..EHMGVNVVRINAASRYFDALQGVYD.PEAKRKIIGNLFIQIFEEEASKR
GUAA_XYLFA  DSSVVAALLQKAIG.SQLTCVFVDTGLLRWGEGDQVMAMFA..EHMGVNVVRINAASRYFDALQGVYD.PEAKRKIIGNLFIQIFEEEASKR
GUAA_CELJU  DSSVVAALLHRAIG.DQLTCVFVDNGLLRKNEGDQVMDMFA..KNMGVRVIRADAEQLFLGKLAGVSD.PEKKRKIIGGTFIDVFDTEATKL
GUAA_TERTT  DSSVVAALLHRAIG.DQLTCVFVDNGLLRKQEGDQVMDMFA..KNMGVKVVRADAEDLFLSKLHGIND.PEQKRKIIGNTFIEVFDTEAAKL
GUAA_HAHCH  DSSVVAALLHRAIG.DQLTCVFVDNGLLRLHEGDQVMDMFA..KNMGIKVVRANAQDQFLSKLAGEND.PERKRKIIGHTFIEVFDAEAAKI
GUAA_MARAV  DSSVTAALLHKAIG.DQLTCVFVDNGLLRLHEGDQVMDMFA..SNMGVKVIRVDAEDLFLSKLKGVND.PEQKRKIIGNTFIDVFDDEAANI
GUAA_PSESM  DSSVVAALLHKAIG.DQLTCVFVDNGLLRLHEGEQVMAMFA..ENMGVKVIRANAEEQFLNNLEGESD.PEKKRKIIGRTFIDVFDAESCKL
GUAA_PSE14  DSSVVAALLHKAIG.DQLTCVFVDNGLLRLHEGEQVMAMFA..ENMGVKVIRANAEEQFLNNLEGESD.PEKKRKIIGRTFIDVFDAESCKL
GUAA_PSEU2  DSSVVAALLHKAIG.DQLTCVFVDNGLLRLHEGEQVMAMFA..ENMGVKVIRANAEEQFLNNLAGESD.PEKKRKIIGRTFIDVFDAESCKL
GUAA_PSEFS  DSSVVAALLHKAIG.DQLTCVFVDNGLLRLHEGEQVMAMFA..ENMGVKVIRANAEDQFLNNLAGESD.PEKKRKIIGRTFIDVFDAQSNKL
GUAA_PSEF5  DSSVVAALLHKAIG.DQLTCVFVDNGLLRLHEGEQVMAMFA..ENMGVKVIRANAEEQFLNNLAGEAD.PEKKRKIIGRTFIDVFDAESCKL
GUAA_PSEPF  DSSVVAALLHKAIG.DQLTCVFVDNGLLRLHEGEQVMAMFA..ENMGVKVIRANAEDQFLNNLAGEAD.PEKKRKIIGRTFIDVFDAESCKL
```

```
GUAA_PSEPK     DSSVVAALLHRAIG.DQLTCVFVDNGLLRLHEGDQVMAMFK..ENMGVKVIRADAEKQFLDNLEGEAD.PEKKRKIIGRTFIDVFDAEASKL
GUAA_PSEP1     DSSVVAALLHRAIG.DQLTCVFVDNGLLRLHEGDQVMAMFK..ENMGVKVIRADAEKQFLDNLAGEAD.PEKKRKIIGRTFIDVFDAEASKL
GUAA_PSEPG     DSSVVAALLHRAIG.DQLTCVFVDNGLLRLHEGDQVMAMFK..ENMGVKVIRADAEKQFLDNLEGEAD.PEKKRKIIGRTFIDIFDAEASKL
GUAA_PSEE4     DSSVVAALLHRAIG.DQLTCVFVDNGLLRLHEGDQVMAMFK..ENMGVKVIRADAEKQFLDNLEGEAD.PEKKRKIIGRTFIDVFDAEASKL
GUAA_PSEPW     DSSVVAALLHRAIG.DQLTCVFVDNGLLRLHEGDQVMAMFK..ENMGVKVIRADAEKQFLDNLEGEAD.PEKKRKIIGRTFIDVFDAEASKL
GUAA_PSEMY     DSSVVAALLHKAIG.DQLTCVFVDNGLLRLHEGDQVMAMFA..ENMGVKVIRANAEEQFLSNLAGESD.PEKKRKIIGRTFIDVFDAEASKL
GUAA_PSEAE     DSSVVAALLHKAIG.DQLTCVFVDNGLLRLHEGDQVMAMFA..ENMGVKVIRANAEDKFLGRLAGVAD.PEEKRKIIGRTFIEVFDEEATKL
GUAA_PSEA8     DSSVVAALLHKAIG.DQLTCVFVDNGLLRLHEGDQVMAMFA..ENMGVKVIRANAEDKFLGRLAGVAD.PEEKRKIIGRTFIEVFDEEATKL
GUAA_PSEAB     DSSVVAALLHKAIG.DQLTCVFVDNGLLRLHEGDQVMAMFA..ENMGVKVIRANAEDKFLGRLAGVAD.PEEKRKIIGRTFIEVFDEEATKL
GUAA_MARMS     DSSVVAALLHKAIG.DQLTCVFVDNGLLRLHEGDQVMKMFA..DNMGVKVIRVDAEDLFLGKLANEAD.PEKKRKIIGNTFIDVFDTEATKL
GUAA_ALCBS     DSSVVAALLHKAIG.DQLTCVFVDNGLLRHQEGDQVMEMFA..DNMGVKVIRVDAEDNFLGKLAGEAD.PEKKRKIIGNTFIDIFDDEASKL
GUAA_THICR     DSSVVAALLHKAIG.DQLTCVFVDHGLLRHQEGDQVMSMFA..ENMGIKVIRVDAEDYFMNALAGEAD.PEKKRKIIGHAFIEMFDQESAKL
GUAA_ALKEH     DSSVVAALLHRAIG.DQLTCVFVDNGLLRKDEGDQVMATFA..RHMGVNVIRVDAEARFLEALKGVDD.PERKRKIIGNMFIEVFDEQAARL
GUAA_METCA     DSSVVAALLQHAIG.EQLTCVFVDTGLLRLNEGDQVMATFA..EHMGVRVIRVNAEERFLQALQGIAD.PEDKRKIIGRLFVEIFDEEAAKI
GUAA_NITOC     DSSVAAALLHKAIG.DQLICVFVDNGLLRQGEADQVMATFA..RHLGIKVIHISAETRFLEALAGVID.PEAKRKIIGRVFIEVFEEEAAKL
GUAA_LEGPL     DSSVVAALLHRAIG.EQLVCVFVDTGLLRLNEAEQVLSMFG..RHMGIRIIAVNAEDKFLTALKGVTC.PEEKRKIIGRTFIEVFDEEAQKL
GUAA_LEGPA     DSSVVAALLHRAIG.EQLVCVFVDTGLLRLNEAEQVLSMFG..RHMGIRIIAVNAEDKFLTALKGVTC.PEEKRKIIGRTFIEVFDEEAQKL
GUAA_LEGPC     DSSVVAALLHRAIG.EQLVCVFVDTGLLRLNEAEQVLSMFG..RHMGIRIIAVNAEDKFLTALKGVTC.PEEKRKIIGRTFIEVFDEEAQKL
GUAA_LEGPH     DSSVVAALLHRAIG.EQLVCVFVDTGLLRLNEAEQVLSMFG..RHMGIRIIAVNAEDKFLTALKGVTC.PEEKRKIIGRTFIEVFDEEAQKL
GUAA_ACIAD     DSSVVAALLHKAIG.DQLTCVFVDNGLLRLNEGDQVMQMFA..DNMGIRVIRADAEERFLTALAGEVD.PEKKRKIIGREFIEVFAEEARKL
GUAA_PSYA2     DSSVVAALLHKAIG.DQLTCVFVDTGLLRLHEGDQVMQIFA..ENMGVKVIRVDAEELFLTALAGESD.PEAKRKIIGKTFIDVFANSAREI
GUAA_RUTMC     DSLVVAVLLQQAVG.KQLTCVFVDNGLLRFNEGNEVMQIFA..QNMDMKVIRANAHKKFYDALLNENE.PEAKRKIIGRAFIEVFEEEAKKL
GUAA_FRAP2     DSSVVAAILHEAIG.DQLTCIFVDTGLLRLNEGDQVMEVFA..EHMDINVIRVNAKNRFLDALKGIGD.PEEKRKIIGKLFVDIFDEEAAKI
GUAA_BURPP     DSSVAAALLHRAIG.DQLTCVFVDHGLLRLNEAEQVMATFA..DHLGVKVIHVDASEVFLRKLAGVTD.PEAKRKIIGAEFVEVFQAEAGRL
GUAA_BURXL     DSSVAAALLHRAIG.DQLTCVFVDHGLLRLNEAEQVMATFA..DHLGVKVIHVDASEVFLRKLAGVTD.PEAKRKIIGAEFVEVFQTEAGKL
GUAA_BURP8     DSSVAAALLHRAIG.DQLTCVFVDHGLLRLNEAEQVMSMFA..DNLGVKVIHVDASEAFMSKLKGVTD.PEAKRKIIGAEFVEVFQTEAGKL
GUAA_POLNS     DSSVAAALIHRAIG.DQLTCVFVDHGLLRLNEGDMVMEMFA..RNLGVKVIRVDAKETFMGELAGVSD.PEAKRKIIGKEFVEIFQAESGKI
GUAA_POLSQ     DSSVAAALIHRAIG.DQLTCVFVDHGLLRLNEGDMVMEMFA..RNLGVKVIRVDAASTFMGKLTGVAD.PEAKRKIIGKEFVEIFQSESGKI
GUAA_DECAR     DSSVAAALIHKAIG.DQLTCVFVDHGLLRLNEGDMVMEMFA..RNLGVKVIRVDAVDDFMGKLAGVSD.PEQKRKIIGKEFVEVFQVESKKL
GUAA_HERAR     DSSVAAALIHRAIG.DQLTCVFVDHGLLRLDEGKMVMEMFA..DHLGVNVIRIDAVDQFMGHLAGVTD.PEAKRKIIGREFVEVFQVEAGKR
GUAA_JANMA     DSSVAAALIHRAIG.DQLTCVFVDHGLLRLDEGKMVMEMFA..ESLGVNVIHIDAVDQFMGHLAGVSD.PEAKRKIIGREFVEVFQVEAGKR
GUAA_AROAE     DSSVVAALLHRAIG.EQLTCVFVDNGLLRLNEAEQVMLTFA..RNLGVKVIHVDATEQFMGHLKGVSD.PEAKRKIIGREFVEVFQAEANKL
GUAA_AZOSB     DSSVAAALIHRAIG.DQLTCVFVDNGLLRLNEGAQVMETFN..RSLGVKVIHVDATEQFMGHLKGVSD.PEQKRKIIGREFVEVFQAEAAKL
GUAA_METFK     DSSVAAALIHRAIG.DQLTCVFVDNGLLRLNEAEQVMETFG..KNLGVKVIHVDASAKFLGDLSGVND.PEAKRKIIGREFVEVFQQEAAKL
GUAA_THIDA     DSSVVAALLHRAVG.DQLTCVFVDNGLLRLNEAEQVMETFA..QNLGVKVIHVDATERFMRELAGVSD.PEQKRKIIGREFVHVFQEEAEKL
GUAA_NITEC     DSSVAAALIHRAIG.DQLVCVFVDNGLLRLNEAKQTMETFS..RNLAVNVIYVDASRQFLEQLKGITD.PEQKRRTIGREFVEIFQQEAAKI
GUAA_NITEU     DSSVAAALIHRAIG.DQLVCVFVDNGLLRLNEAKQIMETFS..RNLAVNVIYIDAGRQFLEQLKGITD.PEQKRRTIGREFVEIFQQEAEKI
GUAA_NITMU     DSSVAAALIHRAIG.KQLTCVFVDNGLLRLNEAEQVMDTFS..RNLGVKVIHVDASEEFLGHLQGITD.PEAKRRIIGREFVEVFQREAAKI
GUAA_NEIMA     DSSVAAALIHRAIG.DQLTCVFVDHGLLRLNEGKMVMDMFA..RNLGVKVIHVDAEGQFMAKLAGVTD.PEKKRKIIGAEFIEVFDAEEKKL
GUAA_NEIMF     DSSVAAALIHRAIG.DQLTCVFVDHGLLRLNEGKMVMDMFA..RNLGVKVIHVDAEGQFMEKLAGVTD.PEKKRKIIGAEFIEVFDAEEKKL
GUAA_NEIG1     DSSVAAALIHRAIG.DQLTCVFVDHGLLRLNEGKMVMDMFA..RNLGVKVIHVDAEGQFMAKLAGVTD.PEKKRKIIGAEFIEVFDAEEKKL
GUAA_NEIG2     DSSVAAALIHRAIG.DQLTCVFVDHGLLRLNEGKMVMDMFA..RNLGVKVIHVDAEGQFMAKLAGVTD.PEKKRKIIGAEFIEVFDAEEKKL
GUAA_NEIM0     DSSVAAALIHRAIG.DQLTCVFVDHGLLRLNEGKMVMDMFA..RNLGVKVIHVDAEGQFMAKLAGVTD.PEKKRKIIGAEFIEVFDAEEKKL
GUAA_NEIMB     DSSVAAALIHRAIG.DQLTCVFVDHGLLRLNESKMVMDMFA..RNLGVKVIHVDAEGQFMAKLAGVTD.PEKKRKIIGAEFIEVFDAEEKKL
GUAA_GEOMG     DSSVAAALIHRAIG.DQLTCVFVDNGLLRLSEGDQVMATFA..ENLGVKVIRVDAEDRFLTALAGESD.PEKKRKIIGKLFVDIFEEESNKI
GUAA_GEOSL     DSSVAAALIHRAIG.DQLTCVFVDNGLLRLAEGDQVMATFA..ENLGVKVIRVDAEQRFLTALADEAD.PEKKRKIIGNLFVEIFEEESSRI
GUAA_GEODF     DSSVAAALIHRAIG.EQLTCVFVDNGLLRLGEGDQVMATFA..QNLGVKVIRVDAEDRFLSALAGVSD.PEKKRKIIGGLFVEIFEEESNKI
GUAA_PELPD     DSSVAAALIHRAIG.DQLTCVFVDNGLLRLEEGDQVMRTFA..ENLGVKVIRVNAEERFLSALAGESD.PEKKRKIIGSLFVEIFDEESNRI
GUAA_PELCD     DSSVAAALIQRAIG.DQLVCVFVDNGLLRLDEGDQVMRTFA..QNMGVNVIRVNAQDRFMTALAGESD.PERKRKIIGNLFVEIFEEESANI
GUAA_BARHE     DSSVVAVLLHEAIG.DQLTCILVDHGLMRKNEAQEVLTLFR..DHYNIKLIHVNAANMFLNALEGEID.PEKKRKTIGRLFIEVFEEETKKI
GUAA_BARQU     DSSVVAVLLHEAIG.DQLTCILVDHGLMRKNEAEEVLKLFR..DHYNIELIHVNAANIFLNALEGETD.PEKKRKTIGRLFIEVFEEETKKI
GUAA_BARBK     DSSVTAVLLHEAIG.DQLTCIFVDHGLIRKNEAEEVLKLFR..DNYNIELIHVNAADMFINALEGETD.PEKKRKTIGRLFIEVFEEETKKI
GUAA_RHIE6     DSSVAALLIHEAVG.DQLTCILVDHGLMRKDEAANVVAMFR..EHYNLHLLHVDASDRFIGELEGVSD.PETKRKIIGRLFIETFEEEAKKL
GUAA_RHILW     DSSVAALLIHEAVG.DQLTCILVDHGLMRKDEAANVVAMFR..EHYNLHLLHVDASDRFIGELEGVSD.PETKRKIIGRLFIETFEEEAKKL
GUAA_RHIEC     DSSVAALLIHEAVG.DQLTCILVDHGLMRKDEAANVVAMFR..EHYNLHLLHVDASDRFIGELEGVSD.PETKRKIIGRLFIETFEEEAKKL
GUAA_RHIL3     DSSVAALLIHEAVG.DQLTCILVDHGLMRKDEAAGVVAMFR..EHYNLHLLHVDAADRFIGELEGVSD.PETKRKIIGRLFIETFEEEARKL
GUAA_AGRRK     DSSVAALLIHEAVG.DQLTCILVDHGLMRKDEAANVVAMFS..EHYNLHLLHVDASERFIGELEGVSD.PETKRKIIGRLFIETFEEEAKKL
GUAA_RHISN     DSSVAALLIHEAVG.DQLTCILVDHGLMRKDEAANVVAMFQ..EHYNLHLLHIDASDRFIGELEGVSD.PETKRKIIGRLFIEVFEEEAKKL
GUAA_RHIME     DSSVAALLIHEAVG.DQLTCILVDHGLMRKDEAANVVAMFK..EHYNLHLLHIDASDRFIGELEGVSD.PETKRKIIGRLFIEVFEEEAKKL
GUAA_SINMW     DSSVAALLIHEAVG.DQLTCILVDHGLMRKDEAANVVAMFK..EHYNLHLLHVDASDRFIGELEGVSD.PETKRKIIGRLFIEVFEEEAGKL
GUAA_AGRVS     DSSVAALLIHEAVG.DQLTCILVDHGLMRKNEAADVVAMFK..EHYNLHLIHVDAIDRFVGELEGVSD.PETKRKIIGRLFIETFEEEAKKL
GUAA_BRUSI     DSSVAALLAHEAVG.DQLTCILVDHGLMRKDEAQQVVEMFR..EHYNLPLILVDASDRFIGALEGESD.PEKKRKTIGRLFIEVFEEEARKL
GUAA_BRUAB     DSSVAALLAHEAVG.DQLTCILVDHGLMRKDEAQQVVEMFR..EHYNLPLILVDASDRFIGALEGESD.PEKKRKTIGRLFIEVFEEEARKL
GUAA_BRUA2     DSSVAALLAHEAVG.DQLTCILVDHGLMRKDEAQQVVEMFR..EHYNLPLILVDASDRFIGALEGESD.PEKKRKTIGRLFIEVFEEEARKL
GUAA_BRUA1     DSSVAALLAHEAVG.DQLTCILVDHGLMRKDEAQQVVEMFR..EHYNLPLILVDASDRFIGALEGESD.PEKKRKTIGRLFIEVFEEEARKL
GUAA_BRUO2     DSSVAALLAHEAVG.DQLTCILVDHGLMRKDEAQQVVEMFR..EHYNLPLILVDASDRFIGALESESD.PEKKRKTIGRLFIEVFEEEARKL
GUAA_BRUSU     DSSVAALLAHEAVG.DQLTCILVDHGLMRKDEAQQVVEMFR..EHYNLPLILVDASDRFIGALEGESD.PEKKRKTIGRLFIEVFEEEARKL
GUAA_BRUC2     DSSVAALLAHEAVG.DQLTCILVDHGLMRKDEAQQVVEMFR..EHYNLPLILVDASDRFIGALEGESD.PEKKRKTIGRLFIEVFEEEARKL
GUAA_BRUMB     DSSVAALLAHEAVG.DQLTCILVDHGLMRKDEVQQVVEMFR..EHYNLPLILVDASDRFIGALEGESD.PEKKRKTIGRLFIEVFEEEARKL
GUAA_RHILO     DSSVAALLIHEAVG.DQLTCILVDHGLMRKDEAAGVVAMFR..QHYNLPLILVDASEKFISALEGEVD.PEKKRKTIGRLFIEVFEEEAKKL
GUAA_AGRT5     DSSVAAILIHEAIG.DQLTCVYVDHGLMRLGESEQVVGMFR..DHYNIPLVHVDAADLFLGELSGVSD.PEVKRKTIGRLFIEVFEAEAAKI
GUAA_BRADU     DSAVAAVLIHEAIG.DQLTCVFVDHGLMRLNEAEQVVDLFR..HHYNIPLVHVDASKQFLGELEGVTD.PETKRKTIGRLFIEVFEAEAKKI
GUAA_BRASO     DSAVAAVLIHEAIG.DQLTCVFVDHGLLRLNEAETVVDLFR..HHYNIPLVHVDASKLFLGELAGVTD.PEAKRKTIGRLFIDVFEAEAKKI
GUAA_OLICO     DSAVAAVLIHEAIG.DQLTCVFVDHGLLRMAEAETVVSLFR..DHYNIPLVHVDASKTFLGELAGVTD.PELKRKTIGRLFIDVFDEEAKKI
GUAA_RUEST     DSSVAAALIHEAIG.DQLTCVFVDHGLLRKNEAEEVVGMFR..DHMNLQVIHADETELFLGELEGQSD.PETKRKIIGKLFIDVFQKYADQI
GUAA_RUEPO     DSSVAAILIHEAIG.DQLTCVFVDHGLLRKNEATEVVGMFR..DNYNIQLIHADESDLFLGELDGQSD.PETKRKIIGKLFIDVFQKHADTI
GUAA_CAUCR     DSSVAAVLIHEAIG.DQLTCVFVDTGLLRKNEADQVVTLFR..DHYNIPLVHVDAGDLFLGELAGVSD.PETKRKTIGRLFIDVFDKEAAKI
GUAA_CAUCN     DSSVAAVLIHEAIG.DQLTCVFVDTGLLRKNEADQVVTLFR..DHYNIPLVHVDAGDLFLGELAGVSD.PETKRKTIGRLFIDVFDKEAAKI
GUAA_PHEZH     DSSVAAVLIHEAIG.DQLTCVFVDHGLLRLNEAEQVVTMFR..DHYNIPLVHVDAAEEFLGALAGVTD.PETKRKTIGRLFVEVFDREAAKI
GUAA_RHORT     DSSVVAALIHEAIG.EQLVCVLVDHGMMRQGETEQVVRVFR..DRFNITLVHRDASELFLGKLDGVVD.PEAKRKIIGATFIDVFDEEARKV
GUAA_GLUOX     DSSVAAKLIHDAIG.DQLTCIFVDPGIMRTGEADEVVKTFR..GRFNIRLVHRDASELFLKELAGVTD.PETKRKTIGRLFIEVFEEEAAKL
GUAA_RHOBA     DSSVVAALLYKAIG.PQLSCILVDNGLLRKNEQQIVLEEFS..NHFKTDLHIVEAEDRFLADLAGIDE.PQEKRRRIGHAFIECFKDEAAKI
GUAA_BACFR     DSSVAAVLLNRAIG.KNLTCIFVDHGMLRKNEFKNVMHDY...ECLGLNVIGVDASEKFFSELEGVTE.PERKRKIIGKGFIDVFDEEAHKL
GUAA_BACFN     DSSVAAVLLNRAIG.KNLTCIFVDHGMLRKNEFKNVMHDY...ECLGLNVIGVDASEKFFSELEGVTE.PERKRKIIGKGFIDVFDEEAHKL
GUAA1_BACTN    DSSVAAVLLNRAIG.KNLTCIFVDHGMLRKNEFKNVMNDY...ECLGLNVIGVDASEKFFAELAGVTE.PERKRKIIGKGFIDVFDVEAHKI
GUAA_CYTH3     DSSVAAVLIHKAIG.PNLTCIFVDNGLLRKNEYEEVLNSY...KHMGLNVIGVDAKKQFYKSLEGLSD.PEAKRKAIGKEFIEVFDQESHKI
GUAA_PELLD     DSTVAAVLVSKAIG.KQLHCVFVDNGLLRKNEARKVMEFLK...PLGLNITLADSADLFLGRLKGVAS.PEKKRKIIGRTFIRVFEEHIN..
GUAA_PROVI     DSTVAAVLVSKAIG.KQLHCVFVDNGLLRKNEAEKVMQFLK...PLGLKVTLADSRDLFLTRLKGVAS.PEKKRKIIGRTFIRVFEEHIH..
GUAA_CHLL2     DSSVAAVLVSRAIG.KNLHCVFVDNGLLRKNEADKVMQFLK...HLGLRLTLADASELFLKRLKNVAS.PEKKRKIIGRTFIQVFEEQLD..
GUAA_CHLPD     DSTVAAVLVSQAIG.KQLHCVFVDNGLLRKNEAVKVMNFLK...PLGLSVTLADASDLFLKRLDKVAS.PEKKRKIIGRTFIHVFEQHLN..
GUAA_PELPB     DSTVAAVMVGKAIG.KQLHCVFVDNGLLRKNEAEKVMTFLS...TLGLRVTLVDASDLFLKRLKTVAS.PEKKRKIIGRTFIQVFEEQME..
GUAA_CHLTE     DSTVAAVLVSKAIG.DKLHCVFVDNGLLRKDEAVKVMEFLK...PLGLNISLVDASDLFLGRLKGVAS.PEKKRKIIGRTFIQVFEKNIH..
GUAA_CHLCH     DSTVAAVLVSKAIG.KQLHCVFVDNGLLRKNEAEKVMHFLK...PLGLHITLADSSDLFLKRLKGVAS.PEKKRKIIGRTFIQVFEEQIH..
GUAA_CHLPB     DSTVAAVLVSKAIG.KQLHCVFVDNGLLRKHEGDKVMKILK...PLGLSVNLVDAEEIFLKRLKSIAS.PEKKRKIIGRTFIHIFEEQIH..
GUAA_CHLT3     DSSVAAVLVSRALG.DKLHCIFVDNGLLRKNEAEKVQEKLK...QLGLNLTTVDASELFLSRLKNVVS.PERKRKIIGRTFIKVFEEHIH..
GUAA_SALRD     DSSVAAALLQRALG.DQLHCIFVNNGLLRKGEWNQVQDTFRG..HFEMDLRTTDATDRFLERLDGVTD.PEEKRTIVGNTFIEVFEEQTEAI
GUAA_GEMAT     DSSVAAALVHKAIG.DQLTCIFVDTGLLRLHEREQVERTMRA..HLGIKLITVRAEDRFLNGLAGEGD.PEKKRKIIGHTFIDVFEDASAEA
GUAA_KLULA     DSTVAAKLMDRAIG.DRFHAIMVDNGVLRLNEAATVKKTLGE..GLGINLTVVDASDEFLDKLKGVTD.PEKKRKIIGNTFIHVFEREAAKI
GUAA_ASHGO     DSTVAAKLMTEAIG.DRFHAILVDNGVLRLNEAANVKKILGE..GLGINLTVVDASEEFLTKLKGVTD.PEKKRKIIGNTFIHVFEREAARI
GUAA_YEAST     DSTVASKLMTEAIG.DRFHAILVDNGVLRLNEAANVKKTLVE..GLGINLMVVDASEEFLSKLKGVTD.PEKKRKIIGNTFIHVFEREAEKI
GUAA_CANGA     DSTVASKLMTEAIG.DRFHAILVDNGVLRLNEAATVKKTLVD..GLGINLTVVDAADEFLDNLKGVTD.PEKKRKIIGNTFIHVFEREAEKI
GUAA_DEBHA     DSTVGAKIMKEAIG.DRFHAIYVDNGLMRLNETEQVYKTLTE..GLGINLTVVDATDLFLGKLQGVTD.PEKKRKIIGNNFIHVFEAEAAKI
GUAA_CANAL     DSTVGAKIMKEAIG.DRFHAIYVDNGVMRKNETESVKKTLDE..GLGINLTVVDAGDLFLGRLKGVTD.PEKKRKIIGNTFIHVFEEEAAKI
GUAA_YARLI     DSTVGAKLMKEAIG.DRFHAILVDNGVMRLNECENVKKTLGE..GLGINLNVVDAADLFLGKLKGVTD.PEKKRKIIGNTFIEVFEEEAAKI
GUAA_PHANO     DSTVAAKLMKEAIG.DRFHAVMVDNGVLRLNEAKQVKETLEK..GLGINLTVIDASDMFLDRLKGITDNPEQKRKVIGNTFIEIFQEKAKEI
GUAA_EMENI     DSTVAAKLMTEAIG.DRFHAVLVDNGCMRLNECEKVQEVLQE..QLGINLTVVDAGEQFLAGLKGVHD.PEQKRKFIGGKFIDVFEDEARKI
GUAA_ASPOR     DSTVAAKLMKEAIG.DRFHAVLVDTGFMRLNECEQVKQTLAE..HLGINLIVADASQVFMEGLKGISD.PEQKRKFIGNKFIDVFEEEAKKI
GUAA_ASPFU     DSTVAAKLMKEAIG.DRFHAVLVNNGCMRLNECETVAETLNK..HLGINLTVVDASKRFLDGLKGVTD.PEKKRMFIGATFIDVFEEEAEKI
GUAA_USTMA     DSSVAAKLMHEAIG.DRFHAVMVDNGVLRTNEAAQVYEMLSK..DLGVNLTVVDASEQFLSRLKDVED.PEQKRKIIGNTFIHVFEAEVAKL
GUAA_DEHM1     DSAVVASLIHKAIG.DQLTCIYVNNGLLRREEADRTLHVFKN..HMGMKIIYVDAVDRFLDSLSGVTD.PEQKRKVIGSEFIKVFEDEACKL
GUAA_DEHMC     DSAVVASLIHKAIG.DQLTCIYVNNGLLRREEADRTLHVFKN..HMGMKIIYVDAIDRFLESLGGITD.PEQKRKVIGSEFIKVFEDEACKL
GUAA_DESPS     DSSVVAAMIHKAIG.SQLTCVYVNNGLMRIGESEGIIKFFKE..NTDLHLIDVDASDYFLGKLEGVTD.PEVKRQHIGLGFIKIFEEEAHKI
GUAA_BRAHW     DSSVAAVLIEKAIG.KQLKCIFVNNGLLRKDEDKKVVEVFRD..NFNIDLIYVDASKRFLDKLAGVTD.PEQKRKVIGHEFVSVFNDEAKKI
GUAA_DESVV     DSTVVAVLLHKAIG.KQLHCIFVDNGVLRLNEGQEVVDYLRE..HFDLNLKYVQAQERFLSKLHGVDD.PEQKRKIIGYTFIEVFDEEAKAL
GUAA_DESVH     DSTVVAVLLHKAIG.KQLHCIFVDNGVLRLNEGQEVVDYLRE..HFDLNLKYVQAQERFLSKLHGVDD.PEQKRKIIGYTFIEVFDEEAKAL
GUAA_DESVM     DSTVVAVLLHKAIG.KRLHCIFVDNGVLRLNEGQEVVDYLRE..HFDLNLKYVQAQRRFLDKLEGVED.PEQKRKIIGYTFIEVFDEEAKAL
GUAA_DESAG     DSTVVAVLLHKAIG.HRLHCIFVDNGVLRLNEVDEVSGYLRE..HFDLNLTVVDARERFMKQFAGVED.PEKKRKIIGYTFIDVFDEESQKI
GUAA_DESMR     DSTVVAVMLHKAIG.KKLHCIFVDNGLLRMGEGEEVAAYLRE..HFDLNLHYVDAAKLFLDKLAGVTD.PEEKRKIIGKTFIEVFEVEAAKL
GUAA_DESAD     DSTVVAVLLHKAIG.KRLHCIFVDNGLLRMHEREEVIGFLEE..HFELNVKCVDSAELFLDKLKGVED.PEKKRKLIGYTFIDVFNEEASAL
GUAA_CALBD     DSSVAAVLVHKAIG.KNLYCIFVDHGLLRKGEAEEVVSVFK..DKFDMNVIKVDAKDRFLEALKGVTD.PEEKRKIIGREFIRVFEEEAEKL
GUAA_CALS8     DSSVAAVLVHKAIG.KNLFCIFVDHGLLRKGEAEEVIKTFK..GQFDMNVIKVDAKERFLKALCGVID.PERKRKIIGEEFIRVFEEEASKL
GUAA_CARHZ     DSSVAAVLVHKAIG.DNLTCIFVDHGLLRKGEAEEVVRTFK..EQFQMNLVFVDAKEQFLAKLKGVRD.PEQKRKIIGHEFIRVFEEEAAKL
GUAA_CLOBL     DSSVAAVIVHKAIG.KQLTCIFVDHGLLRKDEGDQVEKIFK..DQFDMNLIRVNAQDRFLGKLKGVSD.PERKRKIIGEEFIRVFEEEAKKL
GUAA_CLOBK     DSSVAAVIVHKAIG.KQLTCIFVDHGLLRKDEGDQVEKIFK..DQFDMNLIRVNAQDRFLGKLKGVSD.PERKRKIIGEEFIRVFEEEAKKL
GUAA_CLOBM     DSSVAAVIVHKAIG.KQLTCIFVDHGLLRKDEGDQVEKIFK..DQFEMNLIRVNAQDRFLGKLKGVSD.PERKRKIIGEEFIRVFEEEAKKL
GUAA_CLOBJ     DSSVAAVIVHKAIG.KQLTCIFVDHGLLRKDEGDQVEKIFK..DQFDMNLIRVNAQDRFLGKLKGVSD.PERKRKIIGEEFIRVFEEEAKKL
GUAA_CLOB6     DSSVAAVIVHKAIG.KQLTCIFVDHGLLRKDEGDQVEKIFK..DQFDMNLIRVNAQDRFLGKLKGVSD.PERKRKIIGEEFIRVFEEEAKKL
GUAA_CLOBH     DSSVAAVIVHKAIG.KQLTCVFVDHGLLRKDEGDQVERIFK..DQFDMNLIRVNAQDRFLGKLKGVSD.PERKRKIIGEEFIRVFEEEAKKL
GUAA_CLOB1     DSSVAAVIVHKAIG.KQLTCVFVDHGLLRKDEGDQVERIFK..DQFDMNLIRVNAQDRFLGKLKGVSD.PERKRKIIGEEFIRVFEEEAKKL
GUAA_CLONN     DSSVAAVLVHKAIG.KQLTCVFVDHGLLRKDEGDQVEAIFK..KQFDMNLIRVNAGERFLGKLKGVSD.PETKRKIIGEEFIRVFEEEAGKL
GUAA_CLOAB     DSSVAAVLVHKAIG.KQLTCVFVDHGLLRKDEGDQVESIFR..KQFDMNLIRVNAKDRFLGKLKGISD.PERKRKIIGEEFIRVFEEEANKL
GUAA_CLOTE     DSSVAAVIVHKAIG.DQLTCIFVDHGLLRKDEGDQVESIFK..EKFQMNLIRVNAQDRFLGKLKGVTE.PERKRKIIGEEFIRVFEEEANKL
GUAA_CLOPE     DSSVAAVMVHKAVG.KQLTCIFVDHGLLRKDEGDQVEKIFK..EGFDMNLIRVNAQDRFLGKLKGVSD.PETKRKIIGEEFIRVFEEEAGKL
GUAA_CLOB8     DSSVAAMIVHKAIG.HNLTCIFVDHGLLRKDEGDTVERVFK..KEFDMNIKRVNVADRFLGKLAGVSD.PETKRKIIGEEFIRVFEEEAKKV
GUAA_ALKMQ     DSSVAAVLVHQAIG.DNLTCVFVDHGLLRKDEGDWVEDIFR..NQFEMNFIRVNAEERFLGKLKGVTD.PELKRKAIGELFIRVFEEEAQKI
GUAA_CLOTH     DSSVAAVLVHKAVG.KQLTCIFVDHGLLRKYEGDQVEEVFK..KQFDISLIRVNAEDRFLEKLKGVTD.PERKRKIIGEEFIRVFEEEAKKI
GUAA_CLOCE     DSSVAAVLIHKAIG.KQLTCIFVDHGLLRKYEGDQVEQIFR..KQYDINLIRVNCEDRFLQRLKGVSD.PETKRKIIGEEFIRVFEDEAKKI
GUAA_CLOBA     DSSVAAVLLSRAVG.KQLTCVFVDHGLLRKNEGDEVEEIFGPNGQYDLNFIRVNAQERFYEKLAGIEE.PEQKRKIIGEEFIRVFEEEAKKI
GUAA_PEPD6     DSSVAAVLIHKAIG.DNLTCIFVDHGLLRKNEGNDVERIFR..EKFDINLIRVNAEDRFLSKLKGVSE.PEAKRKIIGEEFIRVFEEESNKL
GUAA_FINM2     DSSVCAALLSRAIG.KNLTCVFVDHGLMRKNEGDEVEAAFK...NDELNFVRVNAKDRFLNKLKGVSD.PEQKRKIIGEEFIRVFEDEAKKI
GUAA_TREDE     DSSVLAALLNRAVG.KNLTCIFVDHGLMRKNEGDEVEAAFR...DTPMNFIRVNAESRFLGKLKGVSD.PEKKRKIIGEEFIRVFEEEAEKI
GUAA_BACCR     DSSVVAVLIHKAIG.DQLTCIFVDHGLLRKGEAEGVMKTFS..EGFHMNVIKVDARDRFMDKLKGVED.PEQKRKIIGNEFIYVFDDEASKL
GUAA_BACC4     DSSVVAVLIHKAIG.DQLTCIFVDHGLLRKGEAEGVMKTFS..EGFHMNVIKVDAKDRFMDKLKGVED.PEQKRKIIGNEFIYVFDDEASKL
GUAA_BACC2     DSSVVAVLIHKAIG.DQLTCIFVDHGLLRKGEAEGVMKTFS..EGFHMNVIKVDAQERFMNKLKGVED.PEQKRKIIGNEFIYVFDDEASKL
```

```
GUAA_BACWK            DSSVVAVLIHKAIG.DQLTCIFVDHGLLRKDEAEGVMKTFS..EGFHMNVIKVDAQERFMNKLKGVED.PEQKRKIIGNEFIYVFDDEASKL
GUAA_BACCZ            DSSVVAVLIHKAIG.DQLTCIFVDHGLLRKGEAEGVMKTFS..EGFHMNVIKVDAKERFMNKLKGVED.PEQKRKIIGNEFIYVFDDEASKL
GUAA_BACC3            DSSVVAVLIHKAIG.DQLTCIFVDHGLLRKGEAEGVMKTFS..EGFHMNVIKVDAKERFMNKLKGVED.PEQKRKIIGNEFIYVFDDEASKL
GUAA_BACAH            DSSVVAVLIHKAIG.DQLTCIFVDHGLLRKGEAEGVMKTFS..EGFHMNVIKVDAKERFMNKLKGVED.PEQKRKIIGNEFIYVFDDEASKL
GUAA_BACAC            DSSVVAVLIHKAIG.DQLTCIFVDHGLLRKGEAEGVMKTFS..EGFHMNVIKVDAKERFMNKLKGVED.PEQKRKIIGNEFIYVFDDEASKL
GUAA_BACAA            DSSVVAVLIHKAIG.DQLTCIFVDHGLLRKGEAEGVMKTFS..EGFHMNVIKVDAKERFMNKLKGVED.PEQKRKIIGNEFIYVFDDEASKL
GUAA_BACHK            DSSVVAVLIHKAIG.DQLTCIFVDHGLLRKGEAEGVMKTFS..EGFHMNVIKVDAKERFMNKLKGVED.PEQKRKIIGNEFIYVFDDEASKL
GUAA_BACC0            DSSVVAVLIHKAIG.DQLTCIFVDHGLLRKGEAEGVMKTFS..EGFHMNVIKVDAKERFMNKLKGVED.PEQKRKIIGNEFIYVFDDEASKL
GUAA_BACAN            DSSVVAVLIHKAIG.DQLTCIFVDHGLLRKGEAEGVMKTFS..EGFHMNVIKVDAKERFMNKLKGVED.PEQKRKIIGNEFIYVFDDEASKL
GUAA_BACC1            DSSVVAVLIHKAIG.DQLTCIFVDHGLLRKGEAEGVMKTFS..EGFHMNVIKVDAKERFMNKLKGVED.PEQKRKIIGNEFIYVFDDEASKL
GUAA_BACCN            DSSVVAVLIHKAIG.DQLTCIFVDHGLLRKGEAEGVMKTFS..EGFHMNVIKIDARERFMKKLKGVED.PEQKRKIIGNEFIYVFDDEAAKL
GUAA_BACA2            DSSVVAALIHKAIG.DQLTCIFVDHGLLRKGEAEGVMKTFS..EGFNMNVIKVDAKDRFLNKLKGVSD.PEQKRKIIGNEFIYVFDDEADKL
GUAA_BACSU            DSSVVAVLIHKAIG.DQLTCIFVDHGLLRKGEAEGVMKTFS..EGFNMNVIKVDAKDRFLNKLKGVSD.PEQKRKIIGNEFIYVFDDEADKL
GUAA_BACLD            DSSVVAVLIHKAIG.DQLTCIFVDHGLLRKGEAEGVMKTFS..EGFNMNVIKVDAKDRFLNKLKGVSD.PEQKRKIIGNEFIYVFDDEAEKL
GUAA_BACP2            DSSVVAVLIHKAIG.DQLTCIFVDHGLLRKGEAEGVMKTFS..EGFNMNVIKVDAKDRFLNKLKGVSD.PEQKRKIIGNEFIYVFDDESDKL
GUAA_BACSK            DSSVVAVLIHKAIG.DQLTCMFIDHGLLRKGEAESVMETFT..QGFNMNVVKIDASERFLTKLKGVTD.PEQKRKIIGNEFIYVFDEESAKL
GUAA_BACHD            DSSVVAVLIHKAIG.DQLTCMFIDHGLLRKGEADSVMKTFS..EGFNMNVIKIDAKDRFLSKLEGVSD.PEQKRKIIGNEFIYVFEEEASKL
GUAA_GEOKA            DSSVAAVLVHRAIG.DQLTCIFVDHGLLRKGEAESVMKTFR..EQFQMNVIKVDAKDRFLAKLKGVTD.PEQKRKIIGNEFIYVFDDEAAKL
GUAA_LISMF            DSSVVGVLIHKAIG.DQLTCIFVDHGLLRKGEADQVMATLQ..GEFNMNIIKVDAKKRFMDKLAGVSD.PEQKRKIIGNEFIYVFDDEANKL
GUAA_LISMO            DSSVVGVLIHKAIG.DQLTCIFVDHGLLRKGEADQVMETLQ..GEFNMNIIKVDAKKRFMDKLAGVSD.PEQKRKIIGNEFIYVFDDEANKL
GUAA_LISW6            DSSVVGVLIHKAIG.DQLTCIFVDHGLLRKGEADQVMATLQ..GEFNMNIIKVDAKKRFMDKLAGVSD.PEQKRKIIGNEFIYVFDDEANKL
GUAA_LISIN            DSSVVGVLIHKAIG.DQLTCIFVDHGLLRKGEADQVMATLQ..GEFNMNIIKVDAKKRFMDKLAGVSD.PEQKRKIIGNEFIYVFDDEANKL
GUAA_EXIS2            DSSVVAALIHAAIG.DQLTCMFVDHGLLRKGEAESVMKTFA..DHFHMNVIKIDAQDRFLNKLKGISD.PEQKRKIIGNEFVYVFDEEASKL
GUAA_STAES            DSSVVAVLLHKAIG.EQLTCIFVDHGLLRKGEGDMVMEQFG..EGFDMNIIRVNAQERFMSKLKGVSD.PERKRKIIGNEFVYVFDDEAAKL
GUAA_STAEQ            DSSVVAVLLHKAIG.DQLTCIFVDHGLLRKGEGDMVMEQFG..EGFDMNIIRVNAQERFMSKLKGVSD.PERKRKIIGNEFVYVFDDEAAKL
GUAA_MACCJ            DSSVVAVLIHKAIG.DQLTCIFVDHGLLRKGEGDMVMKQFG..EGFNMNIIRVDAKERFMSKLAGVSD.PEQKRKIIGNEFVYLFDEEAAKL
GUAA_OCEIH            DSSVVAALIHKAIG.DQLTCIFVDHGLLRKNEGDDVMKVFA..EDFQMNIIKVDAKDRFLFKLEGVSD.PELKRKIIGNEFIYVFDDEASKL
GUAA_LACRJ            DSSVTAVLIHKAIG.DQLTCIFVDHGLLRKNEADQVMDALS..RDLGVNIIKVDAADRFLGKLEGVTD.PEQKRKIIGKEFIEVFNEEAKKI
GUAA_LACRD            DSSVTAVLIHKAIG.DQLTCIFVDHGLLRKNEADQVMDALS..RDLGVNIIKVDAADRFLGKLEGVTD.PEQKRKIIGKEFIEVFNEEAKKI
GUAA_LACJO            DSSVTATLLHKAIG.DQLTAIFVDHGMLRKDEGDQVMQALN..KDLGVNIIRVNAQERFLNKLKGVTD.PEQKRKIIGKEFIEVFNEEAKKL
GUAA_LACGA            DSSVTATLLHKAIG.DQLTAIFVDHGMLRKDEGDQVMQALN..KDLGVNIIRVNAQKRFLDKLKGVTD.PEQKRKIIGKEFIEVFNEEAKKL
GUAA_LACAC            DSSVTATLLHKAIG.DQLTAIFVDHGMLRKDEGDQVMKALN..KDLGVNIIRVNAQERFLNKLKGVTD.PEQKRKIIGKEFIEVFNEEAKKL
GUAA_LACDB            DSSVTATLLHKAIG.SQLTAIFVDHGLLRKNESDEVMAALK..EGLGVNIVRVDAKDRFMSKLAGVTD.PEKKRKIIGNEFVQVFNDEAKKI
GUAA_LACDA            DSSVTATLLHKAIG.SQLTAIFVDHGLLRKNESDEVMAALK..EGLGVNIVRVDAKDRFMSKLAGVTD.PEKKRKIIGNEFVQVFNDEAKKI
GUAA_LACCB            DSSVVGVLLHKAIG.TQLTSIFVDHGLLRKGEADQVMESLK..GKFGLNIIKVNAKDRFLGDLKGVTD.PEKKRKIIGRDFIEVFNEEAAKL
GUAA_LACC3            DSSVVGVLLHKAIG.TQLTSIFVDHGLLRKGEADQVMESLK..GKFGLNIIKVNAKDRFLGDLKGVTD.PEKKRKIIGRDFIEVFNEEAAKL
GUAA_LACRH            DSSVVGVLLHKAIG.TQLTSIFVDHGLLRKGEAEQVMDSLK..GKFGLNIIKVNAKDRFLNDLRGVTD.PEKKRKIIGRDFIEVFNEEAAKL
GUAA_LACS1            DSSVVGVLLHKAIG.NQLTSIFVDHGLLRKGEAEQVMDSLG..GKFGLNIIKVDAKERFLNKLAGVSD.PEKKRKIIGNEFIRVFDDESAKL
GUAA_LACLM            DSSVVGVLLQRAIG.DQLTSIFVDHGFLRKGEADQVMETLG..GKFGLNIIKVDAQKRFMDKLVGLSD.PETKRKIIGNEFVYVFDDEANKL
GUAA_LACLS            DSSVVGVLLQRAIG.DQLTSIFVDHGFLRKGEADQVMETLG..GKFGLNIIKVDAQKRFMDKLVGLSD.PETKRKIIGNEFVYVFDDEANKL
GUAA_LACLA            DSSVVGVLLQRAIG.DQLTSIFVDHGFLRKGEADQVMETLG..GKFGLNIIKVDAQKRFMDKLVGLSD.PETKRKIIGNEFVYVFDDEANKL
GUAA_STRZJ            DSSVVGVLLQKAIG.DQLICIFVDHGLLRKGEADQVMDMLG..GKFGLNIVKADAAKRFLDKLAGVSD.PEQKRKIIGNEFVYVFDDEASKL
GUAA_STRPJ            DSSVVGVLLQKAIG.DQLICIFVDHGLLRKGEADQVMDMLG..GKFGLNIVKADAAKRFLDKLAGVSD.PEQKRKIIGNEFVYVFDDEASKL
GUAA_STRZT            DSSVVGVLLQKAIG.DQLICIFVDHGLLRKGEADQVMDMLG..GKFGLNIVKADAAKRFLDKLAGVSD.PEQKRKIIGNEFVYVFDDEASKL
GUAA_STRR6            DSSVVGVLLQKAIG.DQLICIFVDHGLLRKGEADQVMDMLG..GKFGLNIVKADAAKRFLDKLAGVSD.PEQKRKIIGNEFVYVFDDEASKL
GUAA_STRPN            DSSVVGVLLQKAIG.DQLICIFVDHGLLRKGEADQVMDMLG..GKFGLNIVKADAAKRFLDKLAGVSD.PEQKRKIIGNEFVYVFDDEASKL
GUAA_STRPI            DSSVVGVLLQKAIG.DQLICIFVDHGLLRKGEADQVMDMLG..GKFGLNIVKADAAKRFLDKLAGVSD.PEQKRKIIGNEFVYVFDDEASKL
GUAA_STRP7            DSSVVGVLLQKAIG.DQLICIFVDHGLLRKGEADQVMDMLG..GKFGLNIVKADAAKRFLDKLAGVSD.PEQKRKIIGNEFVYVFDDEASKL
GUAA_STRP4            DSSVVGVLLQKAIG.DQLICIFVDHGLLRKGEADQVMDMLG..GKFGLNIVKADAAKRFLDKLAGVSD.PEQKRKIIGNEFVYVFDDEASKL
GUAA_STRP2            DSSVVGVLLQKAIG.DQLICIFVDHGLLRKGEADQVMDMLG..GKFGLNIVKADAAKRFLDKLAGVSD.PEQKRKIIGNEFVYVFDDEASKL
GUAA_STRZP            DSSVVGVLLQKAIG.DQLICIFVDHGLLRKGEADQVMDMLG..GKFGLNIVKADAAKRFLDKLAGVSD.PEQKRKIIGNEFVYVFDDEASKL
GUAA_STRPS            DSSVVGVLLQKAIG.DQLICIFVDHGLLRKGEADQVMDMLG..GKFGLNIVKADAAKRFLDKLAGVSD.PEQKRKIIGNEFVYVFDDEASKL
GUAA_SYMTH            DSSVAAVLVHKAVG.DQLTCIFVDHGLMRKGEPEQVVRTFR..DHFHIHLVHVDASERFLSKLRGVTD.PEQKRKIIGNEFIRLFEDEARKL
GUAA_MOOTA            DSSVAAALVHRAVG.DRLTCVFVNHGLLRQGEAEQVQRAFG..QAMGMNIVYVDASRRFLEKLTGVTD.PEEKRKIIGHEFIRVFEEEARKL
GUAA_HELMI            DSSVAALLVHRAVG.EQLTCVYVDHGFMRLNESERIIKTFR..DELGMNLIAVEASERFMAKVAGVSD.PETKRKSIGNEFIRVFEEEAAKL
GUAA_HALOH            DSAVASTMVHRAIG.DQLTCIFVDHGLLRKGEAEQVKRTFV..DEFNIPLVYVDARERFLNRLNGVKD.PETKRKIIGEEFIRVFEEEARKL
GUAA_THETN            DSAVAAVLVDRAIH.DQLVCIFVDTGLLRTNEGDMVIETFR..KNYDMNIIRVDAKDRFLSRLKGVTD.PEEKRKIIGNVFIEVFKEEAMKI
GUAA_NATTJ            DSTVAALLVQKAIG.DDLTCIFVNHGLLRKNEQDQVRELFE..QEFDINLVYVDASDRFLNKLQGVTD.PEQKRKIIGEEFIRVFEEEARKI
GUAA_MYCTU            DSAVAAALVQRAIG.DRLTCVFVDHGLLRAGERAQVQRDFV..AATGANLVTVDAAETFLEALSGVSA.PEGKRKIIGRQFIRAFEGAVRDV
GUAA_MYCTA            DSAVAAALVQRAIG.DRLTCVFVDHGLLRAGERAQVQRDFV..AATGANLVTVDAAETFLEALSGVSA.PEGKRKIIGRQFIRAFEGAVRDV
GUAA_MYCBT            DSAVAAALVQRAIG.DRLTCVFVDHGLLRAGERAQVQRDFV..AATGANLVTVDAAETFLEALSGVSA.PEGKRKIIGRQFIRAFEGAVRDV
GUAA_MYCBP            DSAVAAALVQRAIG.DRLTCVFVDHGLLRAGERAQVQRDFV..AATGANLVTVDAAETFLEALSGVSA.PEGKRKIIGRQFIRAFEGAVRDV
GUAA_MYCBO            DSAVAAALVQRAIG.DRLTCVFVDHGLLRAGERAQVQRDFV..AATGANLVTVDAAETFLEALSGVSA.PEGKRKIIGRQFIRAFEGAVRDV
GUAA_MYCTO            DSAVAAALVQRAIG.DRLTCVFVDHGLLRAGERAQVQRDFV..AATGANLVTVDAAETFLEALSGVSA.PEGKRKIIGRQFIRAFEGAVRDV
GUAA_MYCMM            DSAVAAALVQRAIG.DRLTCVFVDHGLLRAGERAQVERDFV..AATKANLVTVDVADTFLAALSGVTD.PEGKRKIIGRQFIRAFEGAVRDV
GUAA_MYCPA            DSAVAAALVQRAIG.DRLTCVFVDHGLLRAGERAQVQRDFV..AATGANLVTVDAADTFLQALAGVTN.PEGKRKIIGRQFIRAFEGAVRDV
GUAA_NOSS1            DSSTLAFLMHKAIG.DQLTCVFIDQGFMRKYEPERLVKLFQ..EQFHIPVEYVNARDRFLDIMSGVTD.PEEKRRRIGHEFIQVFEETSKNL
GUAA_ANAVT            DSSTLAFLMHKAIG.DQLTCVFIDQGFMRKYEPERLVKLFQ..EQFHIPVEYVNARDRFLDIMVGVTD.PEEKRRRIGHEFIQVFEETSRNL
GUAA_NOSP7            DSSTLAFLLYKAIG.EQLTCVFIDQGFMRKYEPERLVKLFQ..EQFHIPVEYVNARDRFLAKVAGVTD.PEEKRRRIGHEFINVFEETSKRL
GUAA_THEEB            DSSTLAFLLHRAIG.DQLTCMFIDQGFMRKGEPERLLKLFQ..EQFHIKVEYVNARDRFLAQLVGVTD.PEEKRKRIGHEFIRVFEEESQRL
GUAA_CYAA5            DSSTLAFLLHQAIG.DNLTCMFIDQGFMRKGEPERLMDIFD..KQFHIPVEYVNARDRFLKQLEGVTD.PEKKRRLIGHEFIQVFEEESKRL
GUAA_CYAP8            DSSTLAFLLHRAIG.DNLTCMFIDQGFMRKGEPERLVEIFD..KQFHIPVEYVNARTRFLEQLAGVTD.PETKRRLIGHEFIQVFEEESERL
GUAA_CYAP7            DSSTLAFLLHRAIG.DQLTCMFIDQGFMRKGEPERLMEIFD..HQFHIPVVYVNSREQFLEQLKGVTD.PEEKRRLIGHEFIKVFEEESNRL
GUAA_SYNY3            DSSTLAFLLHRAIG.DNLTCMFIDQGFMRKGEPERLVELFD..HQFHIPVQYVNARDRFLKQLEGVTD.PEEKRRLIGHEFIQVFEEESNRL
GUAA_SYNP2            DSSTLAFLLHQAIG.DQLTCMFIDQGFMRKGEPERLVEIFD..EQFHIPVAYINARDRFIDKLKGVTD.PEEKRRLIGHEFIAVFEEESKRL
GUAA_SYNP6            DSSTLAFLLHRAIG.DQLTCMFIDQGFMRKGEPERLMEVFN..EKFKIHVEYIQARDRFLQRLEGITD.PEEKRKRIGHEFIRVFEEESRRL
GUAA_GLOVI            DSSTLAFLLHRAIG.DNLTCMFIDQGFMRKNEPERLVKLFK..EQFHIPVAYVDAAERFIVRLEGVSD.PEQKRKIIGAEFIRVFESESQRL
GUAA_PROMP            DSSTLAFLLNKAIG.KKLTCMFIDQGFMRKGEPEFLMEFFD..KKFHIKVEYINARERFIDKLNGITD.PEEKRKIIGREFIRVFEEESNRL
GUAA_PROM5            DSSTLAFLLNKAIG.KQLTCMFIDQGFMRKGEPEFLMEFFA..KKFHIKVEYINARERFLHKLNGVTD.PEEKRKIIGGEFIRVFEEESHRL
GUAA_PROMT            DSSTLAFLLNKAIG.PQLTCMFIDQGFMRKGEPEFLMSFFD..EKFKINVEYINARERFISQLKGVTD.PEQKRKIIGREFIRVFEEESLRL
GUAA_PROM1            DSSTLAFLLNKAIG.PQLTCMFIDQGFMRKGEPEFLMSFFD..EKFKINVEYINARERFISQLKGVTD.PEQKRKIIGREFIRVFEEESLRL
GUAA_THERP            DSSVAAALIHRAIG.DQLTPVFVDTGLLREGEAETIREVFG..RHFRMPLVAIDARQRFLVRLRGVSD.PEQKRRLIGEEFVRVFEEIARSQ
GUAA_DEIGD            DSSTLGLLLARAIG.DRLTAVFIDHGLLRLGEREQVEAALR...PLGVNLVTVDASEEFLGALAGVSD.PEQKRKIIGREFIRAFEREARKY
GUAA_DEIDV            DSSTLALLLARAVG.ERLTAVFIDHGLLRLGEREQVEAALR...PLGVNLVTVDAREEFLGSLRGVSD.PEQKRKIIGREFIRAFERETAKL
GUAA_DEIRA            DSSTLALLLAKAVG.ERLTAVFIDHGLLRLGEREQVEAALT...PLGVNLVTVDAKDEFLGQLAGVSD.PEQKRKIIGREFIRAFERETAKL
GUAA_FUSNN            DSSVAAALINKAIG.RQLTCIFVDTGLLRKDEAKQVMEVYA..KNFDMNIKCINAEERFLTKLAGVTD.PETKRKIIGKEFVEVFNEEAKKI
GUAA_MESFL            DSSVAAAIIGKAIG.KQLTCIFVDTGLLRKNETIEVMKAYE..TNFDINIKLVEASDLFFSELKGIKE.PEAKRKIIGKCFIDVFTDAARQH
GUAA_SPIKU            DSSVCAVLLHKAIG.KQLTCIFVDTGLLRQNSGWNDLQKFQ..EKFKLNIIKINAQERFLTALKGVTN.PEEKRKIIGNLFIEIFNEEAIKI
GUAA_BORGA            DSLVCALLIKKAIK.ENLICVFVNTGLLRKNEDK.KILELK..HQYDLNIKYIDASEKFLNYLKNISD.PEEKRKIIGKEFVNVFEKITLED
GUAA_SULDN            DSSVVAAMLYEAIG.DQLVPVFVDNGLLRKGEREQVEEVFKIN..LKVPLVVVDARENFLSKLAGVSD.PEKKRKIIGHTFIEEFEKEAKKH
GUAA_WOLSU            DSSVVAALLYRAIG.EKLIPVFVDHGLLRAGEREAVEKMFKEN..LHVPLITVDASEIFLGKLKGVSD.PETKRKIIGETFIEVFDVEAKKH
GUAA_SULNB            DSSVVAAMLHEALPKEQLICVFVDQGLLRKDEAEQVQDMFKL...LDIPLITIDAKKEFMEALAGVTD.PEQKRKAIGEKFIEVFDKEAGKH
GUAA_ARCB4            DSSVVATLLAEAIG.EQVIPVFVDNGLLRANEREQVETMFKS...RGVNLITVDASETFLSKLAGVTD.PEKKRKIIGETFIEVFDVEAKKH
GUAA_NITSB            DSSVVTALLYEAIG.DKLIPVFVDNGLLREGEREKVEYVFKNM..LKVPLIVVDAKERFLNALQGITD.PEQKRKIIGHTFIEVFEEEAKKH
GUAA_CAMHC            DSSVVAALLAHCIK.ENLIVVFVDNGLLRTDEAKQVEQTFKLK..LGVELISIDASELFLGRLKGITD.PEEKRKIIGKTFIEVFEREAKKH
GUAA_THESQ            DSSVAAVLVHRAIG.KNLVCVFVDHGLLRKNEREEVERVFKEH..FDMNLVVVDARKRFLEKLRGVTD.PEKKRKIIGEEFIRVFEEEAKKH
GUAA_THEMA            DSSVAAVLVHRAIG.KNLVCVFVDHGLLRKNEREEVERVFKEH..FDMNLVVVDARKRFLEKLRGVTD.PEKKRKIIGEEFIRVFEEEAKKH
GUAA_THEP1            DSSVAAVLVHRAIG.KNLVCVFVDHGLLRKNEREEVERVFKEH..FDMNLVVVDARKRFLEKLRGVTD.PEKKRKIIGEEFIRVFEEEAKKH
GUAA_PETMO            DSSVAAVLTHRAIG.NNLKAIFVNHGFLRMNEVEEVESTFRDY..MGLNLTTVDAQERFLSKLKGVTD.PEQKRKIIGEEFIRVFEQEAKKE
GUAA_AQUAE            DSTVAAVLTHRAIG.DKLHCFFIDHGLLRYKEREEVEKNLRS...LGLPLTVVDASEEFLEKLKGVED.PEEKRKIIGRTFIEVFEREAKKI
GUAA_THEAB            DSSVVALLLDKAIG.KNSIPIFVDTGLLRLNERQEVEENFRK...LGIDIVVVDAKERFLSNLKGVED.PEEKRKIIGHTFIDVFYEASMKL
GUAA_THEM4            DSSVVAMLLHKAIG.DNLVPVFVDTGLLRLNEGEEVKENFER...LGIKIFVVDAKKRFLDALKDVED.PEEKRKIIGHTFIDVFYETSMQL
GUAA_PELUB            DSSVVAQLLSKAIG.KNLTCIFVNNGLLRKNEEIQVVNTFK..KKLKINLIYVNAEKEFIRKLTNVSD.PEKKRKIIGNLFIKIFERYANRI
GUAA_MYCPE            DSLVAAVLTAQAIG.DKLYCVFVDHGLLRKNESQEVCALLK..KLIGKNIFLVDQKQLFLSKLKGVVD.PEQKRKIIGKTFIEVFEKVANKI
tr|Q7RIY7|Q7RIY7_PLAYO DSTVAAAMTHKIFK.DRFYGIFIDNGLLRKNEGEKVYTFIKS.TFPDMNITKIDASENFLNQLKGVTD.PEQKRKIIGKLFIEEFEKAVYSM
tr|Q4Z1X2|Q4Z1X2_PLABA DSTVAAAMTHKIFK.DRFYGIFIDNGLLRKNEGEKVYKFIKS.TFPDMNITKIDASENFLNQLKGVTD.PEQKRKIIGKLFIEEFEKAVYSM
tr|Q4Y2M7|Q4Y2M7_PLACH ...................................................................................GKLFIEEFEKAVYSM
tr|B3L3R6|B3L3R6_PLAKH DSTVAAAFTHQIFK.ERFYGIFIDNGLLRKNEGEQVYSFLKG.LFPDMNLTKIDASEIFLNNLKGVTD.PEQKRKIIGKLFIEEFEKAVKNI
tr|A5K7F1|A5K7F1_PLAVS DSTVAAAFTHKIFK.ERFYGIFIDNGLLRKNEGEKVYSFLKG.IFPDMNLTKIDASEIFLNNLKGVTD.PEQKRKIIGKLFIEEFEKAVKNI
tr|Q8IJR9|Q8IJR9_PLAF7 DSTVAAAYTHKIFK.ERFFGIFIDNGLLRKNEAENVYTFLKS.TFPDMNITKIDASENFLSNLQGVTD.PEQKRKIIGKLFIEEFEKAVNNI
```

```
                                                       330        340              350        360        370             380
GUAA_ECO55      ED......................VKWLAQGTIYPDVIESAASAT......GKAHVIKSHHNVGGLPKEMKM....GLVEPLKELFKDEVRKI
GUAA_ECO45      ED......................VKWLAQGTIYPDVIESAASAT......GKAHVIKSHHNVGGLPKEMKM....GLVEPLKELFKDEVRKI
GUAA_ECO57      ED......................VKWLAQGTIYPDVIESAASAT......GKAHVIKSHHNVGGLPKEMKM....GLVEPLKELFKDEVRKI
GUAA_ECO7I      ED......................VKWLAQGTIYPDVIESAASAT......GKAHVIKSHHNVGGLPKEMKM....GLVEPLKELFKDEVRKI
GUAA_ECO5E      ED......................VKWLAQGTIYPDVIESAASAT......GKAHVIKSHHNVGGLPKEMKM....GLVEPLKELFKDEVRKI
GUAA_ECO81      ED......................VKWLAQGTIYPDVIESAASAT......GKAHVIKSHHNVGGLPKEMKM....GLVEPLKELFKDEVRKI
GUAA_ECO8A      ED......................VKWLAQGTIYPDVIESAASAT......GKAHVIKSHHNVGGLPKEMKM....GLVEPLKELFKDEVRKI
GUAA_ECOHS      ED......................VKWLAQGTIYPDVIESAASAT......GKAHVIKSHHNVGGLPKEMKM....GLVEPLKELFKDEVRKI
GUAA_ECOK1      ED......................VKWLAQGTIYPDVIESAASAT......GKAHVIKSHHNVGGLPKEMKM....GLVEPLKELFKDEVRKI
GUAA_ECOL5      ED......................VKWLAQGTIYPDVIESAASAT......GKAHVIKSHHNVGGLPKEMKM....GLVEPLKELFKDEVRKI
GUAA_ECOL6      ED......................VKWLAQGTIYPDVIESAASAT......GKAHVIKSHHNVGGLPKEMKM....GLVEPLKELFKDEVRKI
GUAA_ECOLC      ED......................VKWLAQGTIYPDVIESAASAT......GKAHVIKSHHNVGGLPKEMKM....GLVEPLKELFKDEVRKI
GUAA_ECOSE      ED......................VKWLAQGTIYPDVIESAASAT......GKAHVIKSHHNVGGLPKEMKM....GLVEPLKELFKDEVRKI
GUAA_ECOSM      ED......................VKWLAQGTIYPDVIESAASAT......GKAHVIKSHHNVGGLPKEMKM....GLVEPLKELFKDEVRKI
GUAA_ECOUT      ED......................VKWLAQGTIYPDVIESAASAT......GKAHVIKSHHNVGGLPKEMKM....GLVEPLKELFKDEVRKI
GUAA_ECO24      ED......................VKWLAQGTIYPDVIESAASAT......GKAHVIKSHHNVGGLPKEMKM....GLVEPLKELFKDEVRKI
GUAA_SHISS      ED......................VKWLAQGTIYPDVIESAASAT......GKAHVIKSHHNVGGLPKEMKM....GLVEPLKELFKDEVRKI
GUAA_SHIF8      ED......................VKWLAQGTIYPDVIESAASAT......GKAHVIKSHHNVGGLPKEMKM....GLVEPLKELFKDEVREI
GUAA_SHIFL      ED......................VKWLAQGTIYPDVIESAASAT......GKAHVIKSHHNVGGLPKEMKM....GLVEPLKELFKDEVRKI
GUAA_SHIDS      ED......................VKWLAQGTIYPDVIESAASAT......GKAHVIKSHHNVGGLPKEMKM....GLVEPLKELFKDEVRKI
GUAA_ECOLI      ED......................VKWLAQGTIYPDVIESAASAT......GKAHVIKSHHNVGGLPKEMKM....GLVEPLKELFKDEVRKI
GUAA_ECODH      ED......................VKWLAQGTIYPDVIESAASAT......GKAHVIKSHHNVGGLPKEMKM....GLVEPLKELFKDEVRKI
GUAA_ECOBW      ED......................VKWLAQGTIYPDVIESAASAT......GKAHVIKSHHNVGGLPKEMKM....GLVEPLKELFKDEVRKI
GUAA_ESCF3      ED......................VKWLAQGTIYPDVIESAASAT......GKAHVIKSHHNVGGLPKEMKM....GLVEPLKELFKDEVRKI
GUAA_ECO27      ED......................VKWLAQGTIYPDVIESAASAT......GKAHVIKSHHNVGGLPKEMKM....GLVEPLKELFKDEVRKI
GUAA_SHIB3      ED......................VKWLAQGTIYPDVIESAASAT......GKAHVIKSHHNVGGLPKEMKM....GLIEPLKELFKDEVRKI
GUAA_SHIBS      ED......................VKWLAQGTIYPDVIESAASAT......GKAHVIKSHHNVGGLPKEMKM....GLVEPLKELFKDEVRKI
GUAA_ECOLU      ED......................VKWLAQGTIYPDVIESAASAT......GKAHVIKSHHNVGGLPKEMKM....GLVEPLKELFKDEVRKI
GUAA_SALPK      ED......................VKWLAQGTIYPDVIESAASAT......GKAHVIKSHHNVGGLPKEMKM....GLVEPLKELFKDEVRKI
GUAA_SALPC      ED......................VKWLAQGTIYPDVIESAASAT......GKAHVIKSHHNVGGLPKEMKM....GLVEPLKELFKDEVRKI
GUAA_SALPB      ED......................VKWLAQGTIYPDVIESAASAT......GKAHVIKSHHNVGGLPKEMKM....GLVEPLKELFKDEVRKI
GUAA_SALPA      ED......................VKWLAQGTIYPDVIESAASAT......GKAHVIKSHHNVGGLPKEMKM....GLVEPLKELFKDEVRKI
GUAA_SALCH      ED......................VKWLAQGTIYPDVIESAASAT......GKAHVIKSHHNVGGLPKEMKM....GLVEPLKELFKDEVRKI
GUAA_SALTY      ED......................VKWLAQGTIYPDVIESAASAT......GKAHVIKSHHNVGGLPKEMKM....GLVEPLKELFKDEVRKI
GUAA_SALSV      ED......................VKWLAQGTIYPDVIESAASAT......GKAHVIKSHHNVGGLPKEMKM....GLVEPLKELFKDEVRKI
GUAA_SALNS      ED......................VKWLAQGTIYPDVIESAASAT......GKAHVIKSHHNVGGLPKEMKM....GLVEPLKELFKDEVRKI
GUAA_SALTI      ED......................VKWLAQGTIYPDVIESAASAT......GKAHVIKSHHNVGGLPKEMKM....GLVEPLKELFKDEVRKI
GUAA_SALHS      ED......................VKWLAQGTIYPDVIESAASAT......GKAHVIKSHHNVGGLPKEMKM....GLVEPLKELFKDEVRKI
GUAA_SALG2      ED......................VKWLAQGTIYPDVIESAASAT......GKAHVIKSHHNVGGLPKEMKM....GLVEPLKELFKDEVRKI
GUAA_SALEP      ED......................VKWLAQGTIYPDVIESAASAT......GKAHVIKSHHNVGGLPKEMKM....GLVEPLKELFKDEVRKI
GUAA_SALDC      ED......................VKWLAQGTIYPDVIESAASAT......GKAHVIKSHHNVGGLPKEMKM....GLVEPLKELFKDEVRKI
GUAA_SALA4      ED......................VKWLAQGTIYPDVIESAASAT......GKAHVIKSHHNVGGLPKEMKM....GLVEPLKELFKDEVRKI
GUAA_SALAR      ED......................VKWLAQGTIYPDVIESAASAT......GKAHVIKSHHNVGGLPKEMKM....GLVEPLKELFKDEVRKI
GUAA_KLEP7      DD......................VKWLAQGTIYPDVIESAASAT......GKAHVIKSHHNVGGLPKEMKM....GLVEPLRELFKDEVRKI
GUAA_KLEP3      DD......................VKWLAQGTIYPDVIESAASAT......GKAHVIKSHHNVGGLPKEMKM....GLVEPLRELFKDEVRKI
GUAA_ENT38      ED......................VKWLAQGTIYPDVIESAASAT......GKAHVIKSHHNVGGLPKEMKM....GLVEPLRELFKDEVRKI
GUAA_CITK8      ED......................VKWLAQGTIYPDVIESAASAT......GKAHVIKSHHNVGGLPKEMKM....GLVEPLKELFKDEVRKI
GUAA_CROS8      ED......................VKWLAQGTIYPDVIESAASAT......GKAHVIKSHHNVGGLPKEMKM....GLVEPLKELFKDEVRKI
GUAA_SODGM      SD......................VKWLAQGTIYPDVIESAASAT......GKAHVIKSHHNVGGLPKEMKM....GLVEPLKELFKDEVRKI
GUAA_PECCP      TD......................VKWLAQGTIYPDVIESAASAT......GKAHVIKSHHNVGGLPKEMKL....GLVEPLKELFKDEVRKI
GUAA_PECAS      AE......................VKWLAQGTIYPDVIESAASAT......GKAHVIKSHHNVGGLPKEMKL....GLVEPLKELFKDEVRKI
GUAA_YERPY      EQ......................VKWLAQGTIYPDVIESAASAT......GKAHVIKSHHNVGGLPKEMKL....GLVEPLKELFKDEVRKI
GUAA_YERPS      EQ......................VKWLAQGTIYPDVIESAASAT......GKAHVIKSHHNVGGLPKEMKL....GLVEPLKELFKDEVRKI
GUAA_YERPB      EQ......................VKWLAQGTIYPDVIESAASAT......GKAHVIKSHHNVGGLPKEMKL....GLVEPLKELFKDEVRKI
GUAA_YERP3      EQ......................VKWLAQGTIYPDVIESAASAT......GKAHVIKSHHNVGGLPKEMKL....GLVEPLKELFKDEVRKI
GUAA_YERPP      EQ......................VKWLAQGTIYPDVIESAASAT......GKAHVIKSHHNVGGLPKEMKL....GLVEPLKELFKDEVRKI
GUAA_YERPN      EQ......................VKWLAQGTIYPDVIESAASAT......GKAHVIKSHHNVGGLPKEMKL....GLVEPLKELFKDEVRKI
GUAA_YERPG      EQ......................VKWLAQGTIYPDVIESAASAT......GKAHVIKSHHNVGGLPKEMKL....GLVEPLKELFKDEVRKI
GUAA_YERPE      EQ......................VKWLAQGTIYPDVIESAASAT......GKAHVIKSHHNVGGLPKEMKL....GLVEPLKELFKDEVRKI
GUAA_YERPA      EQ......................VKWLAQGTIYPDVIESAASAT......GKAHVIKSHHNVGGLPKEMKL....GLVEPLKELFKDEVRKI
GUAA_YERE8      EQ......................VKWLAQGTIYPDVIESAASAT......GKAHVIKSHHNVGGLPKEMKL....GLVEPLKELFKDEVRKI
GUAA_SERP5      SE......................VKWLAQGTIYPDVIESAASAT......GKAHVIKSHHNVGGLPKEMKL....GLVEPLKELFKDEVRKI
GUAA_PHOLL      AQ......................VKWLAQGTIYPDVIESAASAT......GKAHVIKSHHNVGGLPEEMKL....GLVEPLKELFKDEVRKI
GUAA_PROMH      PQ......................VKWLAQGTIYPDVIESAASAT......GKAHVIKSHHNVGGLPEDMKL....GLVEPLKELFKDEVRKI
GUAA_EDWI9      PQ......................VKWLAQGTIYPDVIESAASAT......GKAHVIKSHHNVGGLPEEMKL....GLVEPLKELFKDEVRKI
GUAA_AERHH      KN......................ARWLAQGTIYPDVIESAASKT......GKAHVIKSHHNVGGLPEEMKM....GLVEPLRELFKDEVRRV
GUAA_AERS4      KN......................ARWLAQGTIYPDVIESAASKT......GKAHVIKSHHNVGGMPAEMKM....GLVEPLRELFKDEVRRV
GUAA_TOLAT      KN......................AKWLAQGTIYPDVIESAASKT......GKAHVIKSHHNVGGLPDDMKM....GLVEPLRELFKDEVRRV
GUAA_VIBF1      KN......................AKWLAQGTIYPDVIESAASKT......GKAHVIKSHHNVGGLPDDMEM....GLVEPLRELFKDEVRKI
GUAA_VIBFM      KN......................AKWLAQGTIYPDVIESAASKT......GKAHVIKSHHNVGGLPDDMEM....GLVEPLRELFKDEVRKI
GUAA_ALISL      SN......................AKWLAQGTIYPDVIESAASKT......GKAHVIKSHHNVGGLPDDMEM....GLVEPLRELFKDEVRKI
GUAA_VIBSL      KN......................AKWLAQGTIYPDVIESAASKT......GKAHVIKSHHNVGGLPDDMEM....GLVEPLRELFKDEVRKI
GUAA_VIBCM      SN......................AKWLAQGTIYPDVIESAASKT......GKAHVIKSHHNVGGLPDDMKM....GLVEPLRELFKDEVRKI
GUAA_VIBCH      SN......................AKWLAQGTIYPDVIESAASKT......GKAHVIKSHHNVGGLPDDMKM....GLVEPLRELFKDEVRKI
GUAA_VIBC3      SN......................AKWLAQGTIYPDVIESAASKT......GKAHVIKSHHNVGGLPDDMKM....GLVEPLRELFKDEVRKI
GUAA_VIBVU      KN......................AKWLAQGTIYPDVIESAASKT......GKAHVIKSHHNVGGLPDDMEM....GLVEPLRELFKDEVRKI
GUAA_VIBVY      KN......................AKWLAQGTIYPDVIESAASKT......GKAHVIKSHHNVGGLPDDMEM....GLVEPLRELFKDEVRKI
GUAA_VIBPA      KN......................AKWLAQGTIYPDVIESAASKT......GKAHVIKSHHNVGGLPDDMEM....GLVEPLRELFKDEVRKI
GUAA_VIBCB      KN......................AKWLAQGTIYPDVIESAASKT......GKAHVIKSHHNVGGLPDDMEM....GLVEPLRELFKDEVRKI
GUAA_PHOPR      KN......................AKWLAQGTIYPDVIESAASKN......GKAHVIKSHHNVGGLPDDMEM....GLVEPLRELFKDEVRKI
GUAA_COLP3      DN......................AKWLAQGTIYPDVIESAASAT......GKAHVIKSHHNVGGLPDYMKL....GLVEPLRELFKDEVRKI
GUAA_PSYIN      KN......................AKWLAQGTIYPDVIESAGSKT......GKAQVIKSHHNVGGLPDDMEM....GLVEPLRELFKDEVRKI
GUAA_PSEHT      KN......................AKWLAQGTIYPDVIESAASAT......GKAHVIKSHHNVGGLPADMKM....GLVEPLRELFKDEVRKI
GUAA_PSEA6      KN......................AKWLAQGTIYPDVIESAGSAT......GKAHVIKSHHNVGGLPDDMAM....GLVEPLRELFKDEVRKI
GUAA_ALTMD      EN......................AKWLAQGTIYPDVIESAGSAT......GKAHVIKSHHNVGGLPEDMEL....GLVEPLRELFKDEVRKI
GUAA_SHESW      AN......................AKWLAQGTIYPDVIESAGSAT......GKAHVIKSHHNVGGLPDDMAL....GLVEPLRELFKDEVRKI
GUAA_SHEPC      AN......................AKWLAQGTIYPDVIESAGSAT......GKAHVIKSHHNVGGLPDDMAL....GLVEPLRELFKDEVRKI
GUAA_SHEB9      AN......................AKWLAQGTIYPDVIESAGSAT......GKAHVIKSHHNVGGLPDDMEL....GLVEPLRELFKDEVRKI
GUAA_SHEB8      AN......................AKWLAQGTIYPDVIESAGSAT......GKAHVIKSHHNVGGLPDDMEL....GLVEPLRELFKDEVRKI
GUAA_SHEB5      AN......................AKWLAQGTIYPDVIESAGSAT......GKAHVIKSHHNVGGLPDDMEL....GLVEPLRELFKDEVRKI
GUAA_SHEB2      AN......................AKWLAQGTIYPDVIESAGSAT......GKAHVIKSHHNVGGLPDHMEL....GLVEPLRELFKDEVRKI
GUAA_SHESM      VN......................AKWLAQGTIYPDVIESAGSAT......GKAHVIKSHHNVGGLPDDMEL....GLVEPLRELFKDEVRKI
GUAA_SHESA      VN......................AKWLAQGTIYPDVIESAGSAT......GKAHVIKSHHNVGGLPDDMEL....GLVEPLRELFKDEVRKI
GUAA_SHESR      VN......................AKWLAQGTIYPDVIESAGSAT......GKAHVIKSHHNVGGLPDDMEL....GLVEPLRELFKDEVRKI
GUAA_SHEON      VN......................AKWLAQGTIYPDVIESAGSAT......GKAHVIKSHHNVGGLPDDMEL....GLVEPLRELFKDEVRKI
GUAA_SHEDO      AN......................AKWLAQGTIYPDVIESAGSAT......GKAHVIKSHHNVGGLPDDMEM....GLVEPLRELFKDEVRKI
GUAA_SHEPW      AN......................AKWLAQGTIYPDVIESAGSAT......GKAHVIKSHHNVGGLPDDMEL....GLVEPLRELFKDEVRKI
GUAA_SHESH      KN......................AKWLAQGTIYPDVIESAGSAT......GKAHVIKSHHNVGGLPDDMEL....GLVEPLRELFKDEVRKI
GUAA_SHEWM      VN......................AKWLAQGTIYPDVIESAGSAT......GKAHVIKSHHNVGGLPDDMEM....GLVEPLRELFKDEVRKI
GUAA_SHEHH      VN......................AKWLAQGTIYPDVIESAGSAT......GKAHCIKSHHNVGGLPDDMEM....GLVEPLRELFKDEVRKI
GUAA_SHEPA      VN......................AKWLAQGTIYPDVIESAGSAT......GKAHCIKSHHNVGGLPDDMAM....GLVEPLRELFKDEVRKI
GUAA_SHEFN      AN......................AKWLAQGTIYPDVIESAGSAT......GKAHVIKSHHNVGGLPADMKM....GLVEPLRELFKDEVRKI
GUAA_SHELP      VN......................AKWLAQGTIYPDVIESAGSAT......GKAHVIKSHHNVGGLPDDMEL....GLVEPLRELFKDEVRKI
GUAA_SHEAM      VN......................AKWLAQGTIYPDVIESAGSAT......GKAHVIKSHHNVGGLPDDMEL....GLVEPLRELFKDEVRKI
GUAA_IDILO      TE......................VDWLAQGTIYPDVIESAGSKT......GKAHVIKSHHNVGGLPEDMKL....GLVEPLRELFKDEVRKI
GUAA_ACTSZ      TS......................VKWLAQGTIYPDVIESAASKT......GKAHVIKSHHNVGGLPDYMKL....GLVEPLRELFKDEVRKI
GUAA_MANSM      TS......................VKWLAQGTIYPDVIESAASKT......GKAHVIKSHHNVGGLPDYMKL....GLVEPLRELFKDEVRKI
GUAA_ACTPJ      TS......................VKWLAQGTIYPDVIESAASKT......GKAHVIKSHHNVGGLPDYMKL....GLVEPLRELFKDEVRKI
GUAA_ACTP7      TS......................VKWLAQGTIYPDVIESAASKT......GKAHVIKSHHNVGGLPDYMKL....GLVEPLRELFKDEVRKI
GUAA_ACTP2      TS......................VKWLAQGTIYPDVIESAASKT......GKAHVIKSHHNVGGLPDYMKL....GLVEPLRELFKDEVRKI
GUAA_HAEPS      TS......................VKWLAQGTIYPDVIESAASKT......GKAHVIKSHHNVGGLPDYMKL....GLVEPLRELFKDEVRKI
GUAA_PASMU      TS......................VKWLAQGTIYPDVIESAASKT......GKAHVIKSHHNVGGLPDYMKL....GLVEPLRELFKDEVRKI
GUAA_HAEDU      TD......................VKWLAQGTIYPDVIESAASKT......GKAHVIKSHHNVGGLPDYMKL....GLVEPLRELFKDEVRKI
GUAA_HISS1      TD......................VKWLAQGTIYPDVIESAASKT......GKAHVIKSHHNVGGLPDYMKL....GLVEPLRELFKDEVRKI
GUAA_HISS2      TD......................VKWLAQGTIYPDVIESAASKT......GKAHVIKSHHNVGGLPDYMKL....GLVEPLRELFKDEVRKI
GUAA_HAEI8      TN......................VKWLAQGTIYPDVIESAASKT......GKAHVIKSHHNVGGLPDYMKL....GLVEPLRELFKDEVRKI
GUAA_HAEIG      TN......................VKWLAQGTIYPDVIESAASKT......GKAHVIKSHHNVGGLPDYMKL....GLVEPLRELFKDEVRKI
GUAA_HAEIN      TN......................VKWLAQGTIYPDVIESAASKT......GKAHVIKSHHNVGGLPDYMKL....GLVEPLRELFKDEVRKI
GUAA_HAEIE      TN......................VKWLAQGTIYPDVIESAASKT......GKAHVIKSHHNVGGLPDYMKL....GLVEPLRELFKDEVRKI
GUAA_HAMD5      KG......................VKWLAQGTIYTDLVESAASGT......AKSHLIKSHHNVAGLPKEMKL....GLIEPLKELFKDEVRQL
GUAA_XANC5      AN......................AKWLAQGTIYPDVIESAGSKT......GKAHVIKSHHNVGGLPEHMKL....GLVEPLRELFKDEVRRL
GUAA_XANAC      AN......................AKWLAQGTIYPDVIESAGSKT......GKAHVIKSHHNVGGLPEHMKL....GLVEPLRELFKDEVRRL
GUAA_XANCP      AN......................AKWLAQGTIYPDVIESAGSKT......GKAHVIKSHHNVGGLPEHMKL....GLVEPLRELFKDEVRRL
GUAA_XANCB      AN......................AKWLAQGTIYPDVIESAGSKT......GKAHVIKSHHNVGGLPEHMKL....GLVEPLRELFKDEVRRL
GUAA_XANC8      AN......................AKWLAQGTIYPDVIESAGSKT......GKAHVIKSHHNVGGLPEHMKL....GLVEPLRELFKDEVRRL
GUAA_XANOR      AN......................AKWLAQGTIYPDVIESAGSKT......GNAHVIKSHHNVGGLPQHMKL....GLVEPLRELFKDEVRRL
GUAA_XANOM      AN......................AKWLAQGTIYPDVIESAGSKT......GNAHVIKSHHNVGGLPQHMKL....GLVEPLRELFKDEVRRL
GUAA_XANOP      AN......................AKWLAQGTIYPDVIESAGSKT......GKAHVIKSHHNVGGLPQHMKL....GLVEPLRELFKDEVRRL
GUAA_STRMK      KN......................AKWLAQGTIYPDVIESAGSKT......GKAHVIKSHHNVGGLPEHMKL....GLVEPLRELFKDEVRRL
GUAA_STRM5      KN......................AKWLAQGTIYPDVIESAGSKT......GKAHVIKSHHNVGGLPEHMKL....GLVEPLRELFKDEVRRL
GUAA_XYLFT      KQ......................AKWLAQGTIYPDVIESAGSKT......GKAHVIKSHHNVGGLPEQMTL....GMVEPLRELFKDEVRRL
GUAA_XYLF2      KQ......................AKWLAQGTIYPDVIESAGSKT......GKAHVIKSHHNVGGLPEQMTL....GMVEPLRELFKDEVRRL
GUAA_XYLFM      KQ......................AKWLAQGTIYPDVIESAGSKT......GKAHVIKSHHNVGGLPEQMTL....GMVEPLRELFKDEVRRL
GUAA_XYLFA      KQ......................AKWLAQGTIYPDVIESAGSKT......GKAHVIKSHHNVGGLPEQMTL....GMVEPLRELFKDEVRRL
GUAA_CELJU      QE......................VKWLAQGTIYPDVIESAAAKT......GKAHVIKSHHNVGGLPEDMAF....KLVEPLRELFKDEVRAI
GUAA_TERTT      QN......................VKWLAQGTIYPDVIESAASKT......GKAHVIKSHHNVGGLPEDMQF....ELVEPLRELFKDEVRKI
GUAA_HAHCH      KD......................VKWLAQGTIYPDVIESAAAKT......GKAHVIKSHHNVGGLPEDMEM....QLVEPLRELFKDEVRKI
GUAA_MARAV      KD......................VNWLAQGTIYPDVIESAASKT......GKAHVIKSHHNVGGLPETMKM....KLVEPLRELFKDEVRKI
GUAA_PSESM      DN......................IKYLAQGTIYPDVIESAGAKS......GKAHVIKSHHNVGGLPEEMNL....KLVEPLRELFKDEVRRL
GUAA_PSE14      DN......................IKYLAQGTIYPDVIESAGAKS......GKAHVIKSHHNVGGLPEEMNL....KLVEPLRELFKDEVRRL
GUAA_PSEU2      DN......................IKYLAQGTIYPDVIESAGAKS......GKAHVIKSHHNVGGLPEEMNL....KLVEPLRELFKDEVRRL
GUAA_PSEFS      DN......................IKYLAQGTIYPDVIESAGAKS......GKAHVIKSHHNVGGLPEEMNL....KLVEPLRELFKDEVRRL
GUAA_PSEF5      EN......................IKYLAQGTIYPDVIESAGAKS......GKAHVIKSHHNVGGLPEEMNL....KLVEPLRELFKDEVRRL
GUAA_PSEPF      DN......................IKYLAQGTIYPDVIESAGAKS......GKAHVIKSHHNVGGLPEEMNL....KLVEPLRELFKDEVRRL
```

```
GUAA_PSEPK   EN.....................IQFLAQGTIYPDVIESAGAKS......GKAHVIKSHHNVGGLPEEMNL....KLVEPLRELFKDEVRKI
GUAA_PSEP1   EN.....................IQFLAQGTIYPDVIESAGAKS......GKAHVIKSHHNVGGLPEEMNL....KLVEPLRELFKDEVRKI
GUAA_PSEPG   EN.....................IQFLAQGTIYPDVIESAGAKS......GKAHVIKSHHNVGGLPEEMNL....KLVEPLRELFKDEVRKI
GUAA_PSEE4   EN.....................IQFLAQGTIYPDVIESAGAKS......GKAHVIKSHHNVGGLPEEMNL....KLVEPLRELFKDEVRKI
GUAA_PSEPW   DN.....................IQFLAQGTIYPDVIESAGAKS......GKAHVIKSHHNVGGLPEEMNL....KLVEPLRELFKDEVRKI
GUAA_PSEMY   DN.....................IQFLAQGTIYPDVIESAGAKS......GKAHVIKSHHNVGGLPEEMNL....KLVEPLRELFKDEVRKI
GUAA_PSEAE   QD.....................VKFLAQGTIYPDVIESAGAKT......GKAHVIKSHHNVGGLPEDMQF....ELVEPLRELFKDEVRKI
GUAA_PSEA8   QD.....................VKFLAQGTIYPDVIESAGAKT......GKAHVIKSHHNVGGLPEDMQF....ELVEPLRELFKDEVRKI
GUAA_PSEAB   QD.....................VKFLAQGTIYPDVIESAGAKT......GKAHVIKSHHNVGGLPEDMQF....ELVEPLRELFKDEVRKI
GUAA_MARMS   TD.....................VEFLAQGTIYPDVIESAASKT......GKAHVIKSHHNVGGLPEDMQF....KLVEPLRELFKDEVRKL
GUAA_ALCBS   QN.....................ANWLAQGTIYPDVIESAASKT......GKAHVIKSHHNVGGLPEDMKL....KLVEPLRELFKDEVRKI
GUAA_THICR   TG.....................VKWLAQGTIYPDVIESAGSKT......GKAKVIKSHHNVGGLPEDMKL....ELLEPLRELFKDEVRRL
GUAA_ALKEH   TE.....................VDWLAQGTIYPDVIESAGSAT......GKAHVIKSHHNVGGLPEDMTL....KLVEPLRELFKDEVRRI
GUAA_METCA   ED.....................ARWLAQGTIYPDVIESAGSKT......GKAHVIKSHHNVGGLPETMKL....QLVEPLRELFKDEVRQI
GUAA_NITOC   PN.....................AQWLAQGTIYPDVIESAGGKT......GKAQVIKSHHNVGGLPEELNL....KLLEPLRELFKDEVRQL
GUAA_LEGPL   TD.....................IKWLAQGTIYPDVIESAATSTN.....DAAVVIKSHHNVGGLPDTLNL....KLLEPIRELFKDEVRQV
GUAA_LEGPA   TD.....................IKWLAQGTIYPDVIESAATSTN.....DAAVVIKSHHNVGGLPDTLNL....KLLEPIRELFKDEVRHV
GUAA_LEGPC   TD.....................IKWLAQGTIYPDVIESAATSTN.....DAAVVIKSHHNVGGLPDTLNL....KLLEPIRELFKDEVRQV
GUAA_LEGPH   TE.....................IKWLAQGTIYPDVIESAATSTN.....DAAVVIKSHHNVGGLPDTLNL....KLLEPIRELFKDEVRQV
GUAA_ACIAD   DG.....................VKFLAQGTIYPDVIESAASKQ......GKAHVIKSHHNVGGLPDDLEF....ELVEPLRDLFKDEVRKL
GUAA_PSYA2   SEQSDGKV...............IEFLAQGTIYPDVIESAKSHQ......GKAHVIKSHHNVGGLPDDLAF....ELVEPLRDLFKDEVRKL
GUAA_RUTMC   DN.....................IQFLAQGTIYPDVIESADAKY......GKAKLIKSHHNVGGLPNDLQF....KLVEPLKELFKDEVRKI
GUAA_FRAP2   EN.....................AKWLAQGTIYSDVIESAGNSQ......SKAHVIKSHHNVGGLPKEMKL....KLLEPLRELFKDEVRKL
GUAA_BURPP   TD.....................AKWLAQGTIYPDVIESAGKGK......KGAQTIKSHHNVGGLPETLNL....KLLEPLRELFKDEVREL
GUAA_BURXL   TD.....................AKWLAQGTIYPDVIESAGKGK......KATQTIKSHHNVGGLPETLNL....KLLEPLRELFKDEVREL
GUAA_BURP8   TD.....................AKWLAQGTIYPDVIESAGKGK......KAAHTIKSHHNVGGLPETLNL....KLLEPLRELFKDEVREL
GUAA_POLNS   KN.....................AKWLAQGTIYPDVIESAGKGK......KGAHTIKSHHNVGGLPEDMHL....KLLEPLRELFKDEVREL
GUAA_POLSQ   EN.....................AKWLAQGTIYPDVIESAGKGK......KGAHTIKSHHNVGGLPEDMHL....KLLEPLRELFKDEVREL
GUAA_DECAR   AA.....................AKWLAQGTIYPDVIESAGKGK......KGAHTIKSHHNVGGLPEDMHL....KLLEPLRELFKDEVREL
GUAA_HERAR   KN.....................AKWLAQGTIYPDVIESAGKGK......KG.HTIKSHHNVGGLPETLNL....QLLEPLRELFKDEVRQL
GUAA_JANMA   KN.....................AKWLAQGTIYPDVIESAGKGK......KG.HTIKSHHNVGGLPETLNL....QLLEPLRELFKDEVRKL
GUAA_AROAE   PN.....................AKWLAQGTIYPDVIESAGSKT......GKAHTIKSHHNVGGLPETLNL....KLLEPLRELFKDEVREL
GUAA_AZOSB   PK.....................ARWLAQGTIYPDVIESAGAKT......GKAHAIKSHHNVGGLPETLNL....KLLEPLRDLFKDEVREL
GUAA_METFK   PQ.....................AKWLAQGTIYPDVIESAGAKT......KKAHTIKSHHNVGGLPDTLKL....KLLEPLRELFKDEVREL
GUAA_THIDA   PK.....................AKWLAQGTIYPDVIESASAKT......KKAHTIKSHHNVGGLPETLHL....QLLEPLRELFKDEVREL
GUAA_NITEC   EN.....................VKWLAQGTIYPDVIESAGSHT......KKSGLIKSHHNVGGLPETLRL....KLLEPLRELFKDEVREL
GUAA_NITEU   EN.....................AKWLAQGTIYPDVIESAGSHT......KKAGVIKSHHNVGGLPETLHL....KLLEPLRELFKDEVREL
GUAA_NITMU   AN.....................ARWLAQGTIYPDVIESAGAKT......KKANTIKSHHNVGGLPETLHL....KLLEPLRELFKDEVREL
GUAA_NEIMA   TN.....................AKWLAQGTIYPDVIESAGAKT......KKAHAIKSHHNVGGLPENMKL....KLLEPLRDLFKDEVREL
GUAA_NEIMF   TN.....................AKWLAQGTIYPDVIESAGAKT......KKAHAIKSHHNVGGLPENMKL....KLLEPLRDLFKDEVREL
GUAA_NEIG1   TN.....................AKWLAQGTIYPDVIESAGAKT......KKAHAIKSHHNVGGLPENMKL....KLLEPLRDLFKDEVREL
GUAA_NEIG2   TN.....................AKWLAQGTIYPDVIESAGAKT......KKAHAIKSHHNVGGLPENMKL....KLLEPLRDLFKDEVREL
GUAA_NEIM0   TN.....................AKWLAQGTIYPDVIESAGAKT......QKAHAIKSHHNVGGLPENMKL....KLLEPLRDLFKDEVREL
GUAA_NEIMB   TN.....................AKWLAQGTIYPDVIESAGAKT......KKAHAIKSHHNVGGLPENMKL....KLLEPLRDLFKDEVREL
GUAA_GEOMG   TD.....................ARWLAQGTIYPDVIESAGAKT......GKAHNIKSHHNVGGLPDYMKL....KLLEPLRELFKDEVRAI
GUAA_GEOSL   EG.....................ATWLAQGTIYPDVIESAGAKT......GKAHNIKSHHNVGGLPEYMKL....KLLEPLRELFKDEVRAI
GUAA_GEODF   TD.....................ARWLAQGTIYPDVIESAGAKT......GKAHNIKSHHNVGGLPEHMKL....KLLEPLRELFKDEVRAI
GUAA_PELPD   ED.....................ARWLAQGTIYPDVIESAGAKT......GKAHNIKSHHNVGGLPEYMKL....KLLEPLRELFKDEVRAI
GUAA_PELCD   ED.....................ATWLAQGTIYPDVIESAGAKT......GKAHNIKSHHNVGGLPEHMKL....KLLEPLRELFKDEVRAI
GUAA_BARHE   GG.....................AEFLAQGTLYPDVIES.ISAIG......KSVTIKSHHNVGGLPERMNM....KLVEPLRELFKDEVRSL
GUAA_BARQU   EG.....................VEFLAQGTLYPDVIES.VSAIG......ESVTIKSHHNVGGLPERMNM....KLVEPLRELFKDEVRSL
GUAA_BARBK   GG.....................AKFLAQGTLYPDVIES.VSAIG......EAITIKSHHNVGGLPERMNM....KLVEPLRELFKDDVRSL
GUAA_RHIE6   GG.....................ADFLGQGTLYPDVIES.VSFTG.....GPSVTIKSHHNVGGLPERMKM....QLVEPLRELFKDEVRAL
GUAA_RHILW   GG.....................ADFLGQGTLYPDVIES.VSFTG.....GPSVTIKSHHNVGGLPERMKM....QLVEPLRELFKDEVRAL
GUAA_RHIEC   GG.....................ADFLGQGTLYPDVIES.VSFTG.....GPSVTIKSHHNVGGLPERMKM....QLVEPLRELFKDEVRAL
GUAA_RHIL3   GG.....................ADFLGQGTLYPDVIES.VSFTG.....GPSVTIKSHHNVGGLPERMKM....QLVEPLRELFKDEVRAL
GUAA_AGRRK   GG.....................ADFLGQGTLYPDVIES.VSFTG.....GPSVTIKSHHNVGGLPERMNM....QLVEPLRELFKDEVRAL
GUAA_RHISN   GG.....................ADFLAQGTLYPDVIES.VSFTG.....GPSVTIKSHHNVGGLPERMNM....QLVEPLRELFKDEVRVL
GUAA_RHIME   GG.....................ADFLAQGTLYPDVIES.VSFTG.....GPSVTIKSHHNVGGLPERMNM....QLVEPLRELFKDEVRVL
GUAA_SINMW   GG.....................ADFLAQGTLYPDVIES.VSFTG.....GPSVTIKSHHNVGGLPERMNM....QLVEPLRELFKDEVRVL
GUAA_AGRVS   GG.....................ADFLGQGTLYPDVIES.VSFSG.....GPSVTIKSHHNVGGLPDRMNM....KLVEPLRELFKDEVRVL
GUAA_BRUSI   GG.....................ADFLVQGTLYPDVIES.VSFTG.....GPSVTIKSHHNVGGLPERMKM....QLVEPLRELFKDEVRLL
GUAA_BRUAB   GG.....................ADFLVQGTLYPDVIES.VSFTG.....GPSVTIKSHHNVGGLPERMKM....QLVEPLRELFKDEVRLL
GUAA_BRUA2   GG.....................ADFLVQGTLYPDVIES.VSFTG.....GPSVTIKSHHNVGGLPERMKM....QLVEPLRELFKDEVRLL
GUAA_BRUA1   GG.....................ADFLVQGTLYPDVIES.VSFTG.....GPSVTIKSHHNVGGLPERMKM....QLVEPLRELFKDEVRLL
GUAA_BRUO2   GG.....................ADFLVQGTLYPDVIES.VSFTG.....GPSVTIKSHHNVGGLPERMKM....QLVEPLRELFKDEVRLL
GUAA_BRUSU   GG.....................ADFLVQGTLYPDVIES.VSFTG.....GPSVTIKSHHNVGGLPERMKM....QLVEPLRELFKDEVRLL
GUAA_BRUC2   GG.....................ADFLVQGTLYPDVIES.VSFTG.....GPSVTIKSHHNVGGLPERMKM....QLVEPLRELFKDEVRLL
GUAA_BRUMB   GG.....................ADFLVQGTLYPDVIES.VSFTG.....GPSVTIKSHHNVGGLPERMKM....QLVEPLRELFKDEVRLL
GUAA_RHILO   GG.....................ADFLAQGTLYPDVIES.VSFTG.....GPSVTIKSHHNVGGLPERMNM....QLVEPLRELFKDEVRAL
GUAA_AGRT5   AADGKGA................PKFLAQGTLYPDVIES.VSFSG.....GPSVTIKSHHNVGGLPERMNM....QLVEPLRELFKDEVRAL
GUAA_BRADU   GG.....................ADFLAQGTLYPDVIES.VSFTG.....GPSVTIKSHHNVGGLPERMNM....KLVEPLRELFKDEVRKL
GUAA_BRASO   GG.....................ADFLAQGTLYPDVIES.VSFTG.....GPSVTIKSHHNVGGLPARMNM....KLVEPLRELFKDEVRVL
GUAA_OLICO   GG.....................ADFLAQGTLYPDVIES.VSFTG.....GPSVTIKSHHNVGGLPARMKM....KLVEPLRELFKDEVRAL
GUAA_RUEST   EG.....................AEFLAQGTLYPDVIES.VSFSG.....GPSVTIKSHHNVGGLPEKMGL....KLVEPLRELFKDEVRAL
GUAA_RUEPO   EG.....................AEFLAQGTLYPDVIES.VSFSG.....GPSVTIKSHHNVGGLPEKMGL....KLVEPLRELFKDEVRAL
GUAA_CAUCR   EG.....................ATFLAQGTLYPDVVES.VSARG.....GPSAVIKSHHNVGGLPDYMKL....KLVEPLRELFKDEVRAL
GUAA_CAUCN   EG.....................ATFLAQGTLYPDVVES.VSARG.....GPSAVIKSHHNVGGLPDYMKL....KLVEPLRELFKDEVRAL
GUAA_PHEZH   EG.....................AAFLAQGTLYPDVIES.VSARG.....GPSAVIKSHHNVGGLPDYMKL....KLVEPLRELFKDEVRAL
GUAA_RHORT   GG.....................ADFLAQGTLYPDVIES.VSFTG.....GPSVTIKSHHNVGGLPERMKM....ALVEPLRELFKDEVRDL
GUAA_GLUOX   GG.....................AQFLAQGTLYPDVIES.VSFSG.....GPSVTIKSHHNVGGLPDRMNM....ELVEPLRELFKDEVRML
GUAA_RHOBA   ED.....................AHFLAQGTLYPDVIESGADKD......GPAATIKLHHNVGGLPEELGF....ELIEPLRDLFKDEVRRL
GUAA_BACFR   KD.....................VKWLAQGTIYPDCIES..LSIT.....G..TVIKSHHNVGGLPEKMNL....KLCEPLRLLFKDEVRRV
GUAA_BACFN   KD.....................VKWLAQGTIYPDCIES..LSIT.....G..TVIKSHHNVGGLPEKMNL....KLCEPLRLLFKDEVRRV
GUAA1_BACTN  KD.....................VKWLAQGTIYPDCIES..LSIT.....G..TVIKSHHNVGGLPEKMHL....KLCEPLRLLFKDEVRRV
GUAA_CYTH3   AD.....................VKWLGQGTIYPDIIES..VSVK.....GPSATIKSHHNVGGLPEKMKL....KIVEPLNTLFKDEVRRV
GUAA_PELLD   ......................EEKFLVQGTLYPDVIESQSVKG.......PSETIKSHHNVGGLPKRMK....LKLIEPLRELFKDEVRAV
GUAA_PROVI   ......................EEKFLVQGTLYPDVIESVSVKG.......PSETIKSHHNVGGLPKRMK....LKLIEPLRELFKDEVRAV
GUAA_CHLL2   ......................KEKFLVQGTLYPDVIESVSVKG.......PSETIKSHHNVGGLPKRMK....LKLIEPLRELFKDEVRAV
GUAA_CHLPD   ......................EEKYLVQGTLYPDVIESVSVKG.......PSETIKSHHNVGGLPKRMK....LKLIEPLRELFKDEVRAV
GUAA_PELPB   ......................QEKFLVQGTLYPDVIESVSVKG.......PSETIKSHHNVGGLPKRMK....LKLIEPLRELFKDEVRAV
GUAA_CHLTE   ......................DEKFLVQGTLYPDVIESVSVKG.......PSETIKSHHNVGGLPKRMK....LKLIEPLRELFKDEVRAV
GUAA_CHLCH   ......................HEKFLVQGTLYPDVIESISVKG.......PSETIKSHHNVGGLPKRMK....LKLIEPLRELFKDEVRAV
GUAA_CHLPB   ......................EEKFLVQGTLYPDVIESVSVKG.......PSETIKSHHNVGGLPKRMK....LKLIEPLRELFKDEVRAV
GUAA_CHLT3   ......................NEKFLVQGTLYPDVIESVSVKG.......PSQTIKTHHNVGGLPKRMK....LKLIEPLRELFKDEVRKV
GUAA_SALRD   AADLG................HRPTYLAQGTLYPDLIESVSFKG.......PSVTIKTHHNVGGLPEELD....FDLIEPFRELFKDEVREI
GUAA_GEMAT   G....................KNAEFLVQGTLYPDVIESVSAKGG......PSATIKTHHNVGGLKPDMK....FKLIEPLRELFKDEVRNV
GUAA_KLULA   QPKN................GKEIEFLLQGTLYPDVIESISFKG.......PSQTIKTHHNVGGLLENMK....LKLIEPLRELFKDEVREL
GUAA_ASHGO   QPKN................GEEIEFLLQGTLYPDVIESISFKG.......PSQTIKTHHNVGGLLDNMK....LKLIEPLRELFKDEVRHL
GUAA_YEAST   KPKD................GKEIQFLLQGTLYPDVIESISFKG.......PSQTIKTHHNVGGLLENMK....LKLIEPLRELFKDEVRHL
GUAA_CANGA   KPKD................GKAIEFLLQGTLYPDVIESISFKG.......PSQTIKTHHNVGGLLENMK....LKLIEPLRELFKDEVREL
GUAA_DEBHA   KPAS................GQEIEYLLQGTLYPDVIESISFKG.......PSQTIKTHHNVGGLLEDMK....LKLIEPLRELFKDEVRHL
GUAA_CANAL   KPRD................GSEIEYLLQGTLYPDVIESISFKG.......PSQTIKTHHNVGGLLEDMK....LKLIEPLRELFKDEVRHL
GUAA_YARLI   QPTHPE..............AGEIEFLLQGTLYPDVIESISFKG.......PSQTIKTHHNVGGLLDNMK....LKLIEPLRELFKDEVRKL
GUAA_PHANO   AAAAKNS...........SNAGEIEWLLQGTLYPDVIESLSFKG.......PSQTIKTHHNVGGLPANMN....LKLIEPLRELFKDEVRAL
GUAA_EMENI   EAKSN................GKVEWFLQGTLYPDVIESISFKG.......PSQTIKTHHNVGGIAERLMRGHGLKLIEPLRELFKDEVREL
GUAA_ASPOR   EDAAAHS...........ETAGKIGFFLQGTLYPDVIESISFKG.......PSATIKTHHNVGGLPKRMTEGQGLKLIEPLRELFKDEVRDL
GUAA_ASPFU   EALAENS.............GAKVKWFLQGTLYPDVIESISFKG.......PSATIKTHHNVGALPKRMIEGQGMKLIEPLRELFKDEVRQL
GUAA_USTMA   EKQAEDEEAAALAAGKPQEAKGKFEYLLQGTLYPDVIESISFKG.......PSATIKTHHNVGGLLEDMK....LKLIEPLRELFKDEVRAL
GUAA_DEHM1   G.....................KIDFLAQGTLYPDVIESVSSVSK......ASAKIKSHHNVGGLPAHMK....LKLIEPLRYLFKDEVRLL
GUAA_DEHMC   G.....................QIDFLAQGTLYPDVIESVSSVSK......ASAKIKSHHNVGGLPAHMK....LKLIEPLRYLFKDEVRLL
GUAA_DESPS   DG....................DVKFLAQGTLYPDVVESVS..FR......GAAPIKSHHNVGCLPDIMK....LSLIEPLRELFKDEVREL
GUAA_BRAHW   E.....................NVGFLAQGTLYPDVIESVS.LRG......SSAVIKSHHNVGGLPKDMK....FELLEPFRELFKDEVREI
GUAA_DESVV   G.....................HVDFLAQGTLYPDVIESVS.HKG......PSAVIKSHHNVGGLPEKMN....LKLIEPLRELFKDEVRKV
GUAA_DESVH   G.....................HVDFLAQGTLYPDVIESVS.HKG......PSAVIKSHHNVGGLPEKMN....LKLIEPLRELFKDEVRKV
GUAA_DESVM   G.....................HVDFLAQGTLYPDVIESVS.HKG......PSAVIKSHHNVGGLPEKMN....LKLIEPLRELFKDEVRKV
GUAA_DESAG   P.....................NVKYLAQGTLYPDVIESIS.HKG......PSAVIKSHHNVGGLPETMK....LKLIEPLRELFKDEVRKL
GUAA_DESMR   P.....................KVKYLAQGTLYPDVIESVS.FKG......PSAVIKSHHNVGGLPEVMK....LALIEPLRELFKDEVRKV
GUAA_DESAD   K.....................DVKFLAQGTLYPDVIESES.FKG......PSAVIKSHHNVGGLPEDMD....LDLVEPLRELFKDEVRKV
GUAA_CALBD   GD.....................IKFLVQGTIYPDVVESG.........VGKAATIKSHHNVGGLPEKIKFK...QIIEPLRDLFKDEVRRV
GUAA_CALS8   GD.....................VKFLVQGTIYPDVVESG.........VGKAATIKSHHNVGGLPEHIKFE...RIIEPLRELFKDEVRRV
GUAA_CARHZ   GD.....................IRFLVQGTLYPDVVESG.........TATAATIKSHHNVGGLPEDMKFQ....LIEPLKWLFKDEVREL
GUAA_CLOBL   GD.....................ISFLVQGTIYPDIVESG.........TNTSATIKSHHNVGGLPEDMEF....KLIEPLRELFKDEVRAV
GUAA_CLOBK   GD.....................ISFLVQGTIYPDIVESG.........TNTSATIKSHHNVGGLPEDMEF....KLIEPLRELFKDEVRAV
GUAA_CLOBM   GD.....................ISFLVQGTIYPDIVESG.........TNTSATIKSHHNVGGLPEDMEF....KLIEPLRELFKDEVRAV
GUAA_CLOBJ   GN.....................ISFLVQGTIYPDIVESG.........TNTSATIKSHHNVGGLPEDMEF....KLIEPLRELFKDEVRAV
GUAA_CLOB6   GD.....................ISFLVQGTIYPDIVESG.........TNTSATIKSHHNVGGLPEDMEF....KLIEPLRELFKDEVRAV
GUAA_CLOBH   GD.....................ISFLVQGTIYPDIVESG.........TNTSATIKSHHNVGGLPEDMEF....KLIEPLRELFKDEVRAV
GUAA_CLOB1   GD.....................ISFLVQGTIYPDIVESG.........TNTSATIKSHHNVGGLPEDMEF....KLIEPLRELFKDEVRAV
GUAA_CLONN   GQ.....................IDFLVQGTIYPDVVESG.........TNTSATIKSHHNVGGLPEDMQF....SLIEPLRELFKDEVRAV
GUAA_CLOAB   GQ.....................IDFLVQGTIYPDVVESG.........TDTSATIKSHHNVGGLPEDMQF....ELIEPLRELFKDEVRAV
GUAA_CLOTE   GK.....................IDYLVQGTIYPDVVESG.........TDTSATIKSHHNVGGLPEDIEF....ELIEPLRELFKDEVRRV
GUAA_CLOPE   GD.....................IKFLVQGTIYPDVVESG.........TDTSAVIKSHHNVGGLPEDMEF....SLIEPLRELFKDEVRAV
GUAA_CLOB8   GQ.....................IDYLVQGTIYPDIVESG.........TKTSATIKSHHNVGGLPEDMQF....ELIEPLRELFKDEVRAV
GUAA_ALKMQ   GD.....................FDFLVQGTLYPDIIESG.........TETAAVIKSHHNVGGLPEDMKF....QLIEPFKFLFKDEVRIA
GUAA_CLOTH   GT.....................VDFLVQGTIYPDVIESG.........VGDAAVIKSHHNVGGLPDYIDFK...EIIEPLRSLFKDEVRKV
GUAA_CLOCE   GK.....................VDFLVQGTIYPDVIESG.........IGDAAVIKSHHNVGGLPEHVDFK...EIIEPLRSLFKDEVRRA
GUAA_CLOBA   GT.....................VDYLVQGTIYPDVIESG.........LGKSAVIKSHHNVGGLPDYVDFK...EIIEPLRLLFKDEVRKA
GUAA_PEPD6   GK.....................MDFLVQGTIYPDVIESG.........HGNAATIKSHHNVGGIPEDVDFQ...EIVEPLRELFKDEVRKI
GUAA_FINM2   GS.....................VDFLAQGTIYPDVIESG.........QGDASVIKSHHNVGGLPDVVDFK...DLIEPLRDLFKDEVRRL
GUAA_TREDE   GT.....................VDFLAQGTIYADVVESG.........TKGSAVIKSHHNVGGLPDHISFK...SLIEPLKTLFKDEIRNL
GUAA_BACCR   QG.....................MDFLAQGTLYTDIVESG.........TATAQTIKSHHNVGGLPEDMQF....KLIEPLNTLFKDEVRVL
GUAA_BACC4   QG.....................MDFLAQGTLYTDIVESG.........TATAQTIKSHHNVGGLPEDMQF....KLIEPLNTLFKDEVRVL
GUAA_BACC2   QG.....................MDFLAQGTLYTDIVESG.........TATAQTIKSHHNVGGLPEDMQF....KLIEPLNTLFKDEVRVL
```

```
GUAA_BACWK              QG.....................MDFLAQGTLYTDIVESG.........TATAQTIKSHHNVGGLPEDMQF....KLIEPLNTLFKDEVRVL
GUAA_BACCZ              EG.....................MDFLAQGTLYTDIVESG.........TATAQTIKSHHNVGGLPEDMQF....KLIEPLNTLFKDEVRVL
GUAA_BACC3              EG.....................MDFLAQGTLYTDIVESG.........TATAQTIKSHHNVGGLPEDMQF....KLIEPLNTLFKDEVRVL
GUAA_BACAH              EG.....................MDFLAQGTLYTDIVESG.........TATAQTIKSHHNVGGLPEDMQF....KLIEPLNTLFKDEVRVL
GUAA_BACAC              EG.....................MDFLAQGTLYTDIVESG.........TATAQTIKSHHNVGGLPEDMQF....KLIEPLNTLFKDEVRVL
GUAA_BACAA              EG.....................MDFLAQGTLYTDIVESG.........TATAQTIKSHHNVGGLPEDMQF....KLIEPLNTLFKDEVRVL
GUAA_BACHK              EG.....................MDFLAQGTLYTDIVESG.........TATAQTIKSHHNVGGLPEDMQF....KLIEPLNTLFKDEVRVL
GUAA_BACC0              EG.....................MDFLAQGTLYTDIVESG.........TATAQTIKSHHNVGGLPEDMQF....KLIEPLNTLFKDEVRVL
GUAA_BACAN              EG.....................MDFLAQGTLYTDIVESG.........TATAQTIKSHHNVGGLPEDMQF....KLIEPLNTLFKDEVRVL
GUAA_BACC1              EG.....................MDFLAQGTLYTDIVESG.........TATAQTIKSHHNVGGLPEDMQF....KLIEPLNTLFKDEVRVL
GUAA_BACCN              EG.....................IDFLAQGTLYTDIIESG.........TATAQTIKSHHNVGGLPEDMQF....KLIEPLNTLFKDEVRVL
GUAA_BACA2              KG.....................IDYLAQGTLYTDIIESG.........TATAQTIKSHHNVGGLPEDMQF....ELIEPLNTLFKDEVRAL
GUAA_BACSU              KG.....................IDYLAQGTLYTDIIESG.........TATAQTIKSHHNVGGLPEDMQF....ELIEPLNTLFKDEVRAL
GUAA_BACLD              KG.....................IDFLAQGTLYTDIIESG.........TATAQTIKSHHNVGGLPEDMQF....ELIEPLNTLFKDEVRAL
GUAA_BACP2              KG.....................IDYLAQGTLYTDIIESG.........TATAQTIKSHHNVGGLPEDMQF....ELIEPLNTLFKDEVRAL
GUAA_BACSK              KDK...................KMDFLAQGTLYTDIIESG.........TDTAQTIKSHHNVGGLPEDMAF....ELIEPLNALFKDEVRAL
GUAA_BACHD              KD.....................MDFLAQGTLYTDIIESG.........TATAQTIKSHHNVGGLPEDMRF....ELIEPLNTLFKDEVRKL
GUAA_GEOKA              EG.....................IEFLVQGTLYTDIIESG.........TATAQTIKSHHNVGGLPEDMKF....ELIEPLNTLFKDEVRAL
GUAA_LISMF              DG.....................VEFLAQGTLYTDIIESG.........TATAQTIKSHHNVGGLPEDMQF....KLIEPLNTLFKDEVRAL
GUAA_LISMO              DG.....................VEFLAQGTLYTDIIESG.........TATAQTIKSHHNVGGLPEDMQF....KLIEPLNTLFKDEVRAL
GUAA_LISW6              DG.....................VEFLAQGTLYTDIIESG.........TATAQTIKSHHNVGGLPEDMQF....KLIEPLNTLFKDEVRAL
GUAA_LISIN              DG.....................VEFLAQGTLYTDIIESG.........TATAQTIKSHHNVGGLPEDMQF....KLIEPLNTLFKDEVRAL
GUAA_EXIS2              TD.....................MDFLAQGTLYTDIIESG.........TDTAQTIKSHHNVGGLPEDMQF....KLIEPINTLFKDEVREL
GUAA_STAES              TD.....................VDFLAQGTLYTDVIESG.........TKTAQTIKSHHNVGGLPEDMEF....ELIEPINTLFKDEVRAL
GUAA_STAEQ              TD.....................VDFLAQGTLYTDVIESG.........TKTAQTIKSHHNVGGLPEDMEF....ELIEPINTLFKDEVRAL
GUAA_MACCJ              KD.....................ADFLAQGTLYTDIIESG.........TKTAQTIKSHHNVGGLPEDMQF....ELIEPVNTLFKDEVRAL
GUAA_OCEIH              KD.....................IDFLAQGTLYTDVIESG.........TDTAQTIKSHHNVGGLPEDMQF....TLIEPLNTLFKDEVREL
GUAA_LACRJ              KD.....................ADFLAQGTLYTDVIESG.........TDTAQTIKSHHNVGGLPKKLGF....KLIEPLRKLFKDETREL
GUAA_LACRD              KD.....................ADFLAQGTLYTDVIESG.........TDTAQTIKSHHNVGGLPKKLGF....KLIEPLRKLFKDETREL
GUAA_LACJO              KD.....................VDFLAQGTLYTDVIESG.........TNTAQTIKSHHNVGGLPEDMHF....ELIEPLRKLFKDEVREL
GUAA_LACGA              KD.....................VDFLAQGTLYTDVIESG.........TNTAQTIKSHHNVGGLPEDMHF....ELIEPLRKLFKDEVREL
GUAA_LACAC              KD.....................VDFLAQGTLYTDVIESG.........TNTAQTIKSHHNVGGLPEDMNF....KLIEPLRKLFKDEVREL
GUAA_LACDB              KD.....................AKFLAQGTLYTDVIESG.........TDTAHTIKSHHNVGGLPEDMQF....KLIEPLRQLFKDEVRVL
GUAA_LACDA              KD.....................AKFLAQGTLYTDVIESG.........TDTAHTIKSHHNVGGLPEDMQF....KLIEPLRQLFKDEVRVL
GUAA_LACCB              NG.....................IDFLAQGTLYTDVVESG.........TDTAQTIKSHHNVGGLPEDLKF....KLIEPLNKLFKDEVREL
GUAA_LACC3              NG.....................IDFLAQGTLYTDVVESG.........TDTAQTIKSHHNVGGLPEDLKF....KLIEPLNKLFKDEVREL
GUAA_LACRH              NG.....................IEFLAQGTLYTDVVESG.........TDTAQTIKSHHNVGGLPEDLKF....KLIEPLNKLFKDEVREL
GUAA_LACS1              EG.....................IEFLAQGTLYTDIIESG.........TDTAQTIKSHHNVGGLPEDVQF....KLIEPLNTLFKDEVREL
GUAA_LACLM              EG.....................VDFLAQGTLYTDVIESG.........TDTAQTIKSHHNVGGLPEDMQF....QLIEPLNTLFKDEVRAL
GUAA_LACLS              EG.....................VDFLAQGTLYTDVIESG.........TDTAQTIKSHHNVGGLPEDMQF....QLIEPLNTLFKDEVRAL
GUAA_LACLA              EG.....................VDFLAQGTLYTDVIESG.........TDTAQTIKSHHNVGGLPEDMQF....QLIEPLNTLFKDEVRAL
GUAA_STRZJ              KD.....................VKFLAQGTLYTDVIESG.........TDTAQTIKSHHNVGGLPEDMQF....ELIEPLNTLYKDEVRAL
GUAA_STRPJ              KD.....................VKFLAQGTLYTDVIESG.........TDTAQTIKSHHNVGGLPEDMQF....ELIEPLNTLYKDEVRAL
GUAA_STRZT              KD.....................VKFLAQGTLYTDVIESG.........TDTAQTIKSHHNVGGLPEDMQF....ELIEPLNTLYKDEVRAL
GUAA_STRR6              KD.....................VKFLAQGTLYTDVIESG.........TDTAQTIKSHHNVGGLPEDMQF....ELIEPLNTLYKDEVRAL
GUAA_STRPN              KD.....................VKFLAQGTLYTDVIESG.........TDTAQTIKSHHNVGGLPEDMQF....ELIEPLNTLYKDEVRAL
GUAA_STRPI              KD.....................VKFLAQGTLYTDVIESG.........TDTAQTIKSHHNVGGLPEDMQF....ELIEPLNTLYKDEVRAL
GUAA_STRP7              KD.....................VKFLAQGTLYTDVIESG.........TDTAQTIKSHHNVGGLPEDMQF....ELIEPLNTLYKDEVRAL
GUAA_STRP4              KD.....................VKFLAQGTLYTDVIESG.........TDTAQTIKSHHNVGGLPEDMQF....ELIEPLNTLYKDEVRAL
GUAA_STRP2              KD.....................VKFLAQGTLYTDVIESG.........TDTAQTIKSHHNVGGLPEDMQF....ELIEPLNTLYKDEVRAL
GUAA_STRZP              KD.....................VKFLAQGTLYTDVIESG.........TDTAQTIKSHHNVGGLPEDMQF....ELIEPLNTLYKDEVRAL
GUAA_STRPS              KD.....................VKFLAQGTLYTDVIESG.........TDTAQTIKSHHNVGGLPEDMQF....ELIEPLNTLYKDEVRAL
GUAA_SYMTH              G.....................QIDFLVQGTVYPDVIESG........TETARV.IKSHHNVGGLPEDMRF....QLIEPFRQLFKDEVRAV
GUAA_MOOTA              G.....................RVDFLVQGTLYPDVIESG........TETAAV.IKSHHNVGGLPEDMEL....ELIEPLRLLFKDEVRRV
GUAA_HELMI              G.....................QVDFLVQGTLYPDVVESG........TSTAET.IKTHHNVGGLPEDMKF....ELIEPLRTLFKDEVREV
GUAA_HALOH              G.....................DARYLVQGTLYSDVIESG........SETAET.IKSHHNVGGLPDNMDF....KLIEPLRNLFKDEVRKI
GUAA_THETN              G.....................DVKFLVQGTLYPDVIESG........HGISST.IKSHHNVGGLPEDIGF....ELIEPLRMLFKDEVRQV
GUAA_NATTJ              G.....................DVDFLVQGTLYTDVVESG........TETASV.IKSHHNVGGLPEKMDL....SLIEPLNSLFKDEVRQV
GUAA_MYCTU              LDGK..................TAEFLVQGTLYPDVVESG........GGSGTANIKSHHNVGGLPDDLKF....TLVEPLRLLFKDEVRAV
GUAA_MYCTA              LDGK..................TAEFLVQGTLYPDVVESG........GGSGTANIKSHHNVGGLPDDLKF....TLVEPLRLLFKDEVRAV
GUAA_MYCBT              LDGK..................TAEFLVQGTLYPDVVESG........GGSGTANIKSHHNVGGLPDDLKF....TLVEPLRLLFKDEVRAV
GUAA_MYCBP              LDGK..................TAEFLVQGTLYPDVVESG........GGSGTANIKSHHNVGGLPDDLKF....TLVEPLRLLFKDEVRAV
GUAA_MYCBO              LDGK..................TAEFLVQGTLYPDVVESG........GGSGTANIKSHHNVGGLPDDLKF....TLVEPLRLLFKDEVRAV
GUAA_MYCTO              LDGK..................TAEFLVQGTLYPDVVESG........GGSGTANIKSHHNVGGLPDDLKF....TLVEPLRLLFKDEVRAV
GUAA_MYCMM              LDGR..................DIEFLVQGTLYPDVVESG........GGSGTANIKSHHNVGGLPDDLKF....ALVEPLRLLFKDEVRAV
GUAA_MYCPA              LGDK..................PVEFLVQGTLYPDVVESG........GGSGTANIKSHHNVGGLPGDLKF....TLVEPLRLLFKDEVRAV
GUAA_NOSS1              G.....................PFDYLAQGTLYPDVIESADTNVDPQTGERVAVKIKSHHNVGGLPKDLRF....KLVEPLRKLFKDEVRKV
GUAA_ANAVT              G.....................PFDYLAQGTLYPDVIESADTNVDPQTGERVAVKIKSHHNVGGLPKDLRF....KLVEPLRKLFKDEVRKV
GUAA_NOSP7              G.....................HFDYLAQGTLYPDVIESADTNADPQTGERVAVKIKSHHNVGGLPKDLRF....KLVEPLRKLFKDEVRKV
GUAA_THEEB              G.....................PFDYLAQGTLYPDVIESANTNIDPQTGERVAVKIKSHHNVGGLPENLRF....KLVEPLRKLFKDEVRQV
GUAA_CYAA5              G.....................PFDYLAQGTLYPDVIESADTNIDPKTGERVAVKIKSHHNVGGLPKDLRF....KLVEPLRKLFKDEVRKV
GUAA_CYAP8              G.....................PFDYLAQGTLYPDVIESADTNVDPQTGERVAVKIKSHHNVGGLPKNLRF....KLVEPLRKLFKDEVRKL
GUAA_CYAP7              G.....................PFDYLAQGTLYPDVIESADSNVDPQTGERVAVKIKSHHNVGGLPKNLRF....KLVEPLRKLFKDEVRKV
GUAA_SYNY3              G.....................PFDYLAQGTLYPDVIESADSNVDPKTGERVAVKIKSHHNVGGLPKNLRF....KLVEPLRKLFKDEVRKL
GUAA_SYNP2              G.....................PFDYLAQGTLYPDVIESADTNVDPKTGERVAVKIKSHHNVGGLPEDLRF....KLVEPLRKLFKDEVRKV
GUAA_SYNP6              G.....................PFDYLAQGTLYPDVIESADTNVDPKTGERVAVKIKSHHNVGGLPKDLQF....KLVEPLRRLFKDEVRQV
GUAA_GLOVI              G.....................PFDYLAQGTLYPDIIESAGENIDPKTGERVAVKIKSHHNVGGLPENLRF....KLVEPLKRLFKDEVRQV
GUAA_PROMP              G.....................PFQYLAQGTLYPDVIESAGTNLDPKTGERIAVKIKSHHNVGGLPKDLQF....KLVEPLRKLFKDEVRKV
GUAA_PROM5              G.....................PFEYLAQGTLYPDVIESAGTNLDPKTGERIAVKIKSHHNVGGLPKDLQF....KLVEPLRKLFKDEVRKL
GUAA_PROMT              G.....................PFDYLAQGTLYPDVIESSGTNIDPKTGERIAVKIKSHHNVGGLPKDLQF....KLVEPLRRLFKDEVRKV
GUAA_PROM1              G.....................PFDYLAQGTLYPDVIESSGTNIDPKTGERIAVKIKSHHNVGGLPKDLQF....KLVEPLRRLFKDEVRKV
GUAA_THERP              G.....................PFRFLAQGTLYPDVIESAAPGAS.....RTAAKIKTHHNVGGLPQDLEF....ELLEPLRYLFKDEVRAI
GUAA_DEIGD              GP.....................FDFLAQGTLYPDVIESAGGE........GAANIKSHHNVGGLPEDLAF....KLVEPFRTLFKDEVREI
GUAA_DEIDV              GD.....................FDFLAQGTLYPDVIESAGGHHG...DKSGAANIKSHHNVGGLPEDLKF....KLVEPFRTLFKDEVREI
GUAA_DEIRA              GD.....................FEFLAQGTLYPDVIESAGGE........GAANIKSHHNVGGLPDDVQF....KLVEPFRTLFKDEVREI
GUAA_FUSNN              EG.....................AEFLAQGTIYPDVIESVSVKG.......PSVTIKSHHNVGGLPEDLKF....ELLEPLRELFKDEVRKV
GUAA_MESFL              KD.....................ADFLAQGTIYPDVIESSSHGA.......SSKTIKSHHNVGGLPEDLKF....RLIEPLRNLFKDEVRQV
GUAA_SPIKU              QN.....................VKWLGQGTIYPDVIESVSVKG.......PSATIKSHHNVGGLPKDLPF....QLIEPLRELFKDEVRRT
GUAA_BORGA              QN.....................IEYLAQGTIYSDVIESKSKNN.......ASSKIKSHHNVGGLPDKMRL....KLLEPLNEFFKDEIIQI
GUAA_SULDN              DG.....................IKFLAQGTLYPDVIESISVNG.......PSEVIKSHHNVGGLPDWMDF....ELIEPLRELFKDEVRKI
GUAA_WOLSU              DG.....................IQYLAQGTLYPDVIESVSVKG.......PSKTIKSHHNVGGLPDWMTF....ELIEPLRELFKDEVRAL
GUAA_SULNB              TD.....................VSFLAQGTLYTDVIESVSVKG.......PSKTIKSHHNVGGLPDWMTF....ELVEPLREIFKDEVRKL
GUAA_ARCB4              SG.....................IEFLAQGTLYTDVIESVSVKG.......PSKTIKSHHNVGGLPDWMKF....ELIEPLREIFKDEVRAL
GUAA_NITSB              TD.....................VKYLAQGTLYPDVIESVSVKG.......PSETIKSHHNVGGLPDWMKF....ELIEPLRELFKDEVRKL
GUAA_CAMHC              QN.....................VKYLAQGTLYTDIIES.SVVG.......SSKTIKSHHNVGGLPKDMKF....KLIEPLKEIFKDEVRKL
GUAA_THESQ              D......................VEFLVQGTIYSDVIESAAS.......GKTTAKIKSHHNVGGLPEKMNL....KLVEPLRDLFKDEVRKV
GUAA_THEMA              D......................VEFLVQGTIYSDVIESAAS.......GKTTAKIKSHHNVGGLPEKMNL....KLVEPLRDLFKDEVRKV
GUAA_THEP1              D......................VEFLVQGTIYSDVIESAAS.......GKTTAKIKSHHNVGGLPEKMNL....KLVEPLRDLFKDEVRKV
GUAA_PETMO              EG.....................CEYLIQGTIYSDVIESAKS.......GKKTFKIKSHHNVGGLPEDIDL....KIVEPLKELFKDEVRSV
GUAA_AQUAE              EG.....................AEFLLQGTLYPDVVESAG........IKGSAKIKTHHNVGGLPERMNL....KLLEPFRELFKDEVRKI
GUAA_THEAB              LEKHG.................NIKYLAQGTLYPDIIESKVSE......RKAAAKIKTHHNVGGLPEKLPF....KIIEPFRYLFKDEVRKI
GUAA_THEM4              LEEFG.................NIKYLAQGTLYPDIIESKVSE......RKSAAKIKTHHNVGGLPEKLPF....KIIEPLRYLFKDEVRKI
GUAA_PELUB              KN.....................VKFLAQGTLYPDLIESKSVTG......SQTSKIKSHHNVGGLPKRMKL....KLVEPLKFLFKDEVRKL
GUAA_MYCPE              NVP....................FKYLLQGTIYPDIVESGS.........KFSKTIKSHHNVGGLPERLQF....ELLEPLKYLFKDEVRKL
tr|Q7RIY7|Q7RIY7_PLAYO  DID..................IEKTYLLQGTLYPDIIESKCSKN.......LSDTIKTHHNVGGLPKNLKF....KLFEPFKFLFKDDVKKL
tr|Q4Z1X2|Q4Z1X2_PLABA  DID..................IEKTYLLQGTLYPDIIESKCSKN.......LSDTIKTHHNVGGLPKNLKF....KLFEPFKFLFKDDVKKL
tr|Q4Y2M7|Q4Y2M7_PLACH  DID..................IEKTYLLQGTLYPDIIESKCSKN.......LSDTIKTHHNVGGLPKNLKF....KLFEPFKFLFKDDVKKL
tr|B3L3R6|B3L3R6_PLAKH  NID..................IEKTYLLQGTLYPDIIESKCSKK.......LSDTIKTHHNVGGLPENLKF....KLFEPFKYLFKDDVKKL
tr|A5K7F1|A5K7F1_PLAVS  NID..................IEKTYLLQGTLYPDIIESKCSKR.......LSDTIKTHHNVGGLPENLKF....KLFEPFKYLFKDDVKKL
tr|Q8IJR9|Q8IJR9_PLAF7  DID..................INKTFLLQGTLYPDIIESKCSKN.......LSDTIKTHHNVGGLPKNLKF....KLFEPFKYLFKDDVKTL
```

```
             390       400       410        420       430                          440       450
GUAA_ECO55 GLELGLPYDMLYRHPFPGPGLGVRVLG.EVKKEYCDLLRRADAIFIEELRK...................ADLYDKVSQAFTVFL.PVRSVG
GUAA_ECO45 GLELGLPYDMLYRHPFPGPGLGVRVLG.EVKKEYCDLLRRADAIFIEELRK...................ADLYDKVSQAFTVFL.PVRSVG
GUAA_ECO57 GLELGLPYDMLYRHPFPGPGLGVRVLG.EVKKEYCDLLRRADAIFIEELRK...................ADLYDKVSQAFTVFL.PVRSVG
GUAA_ECO7I GLELGLPYDMLYRHPFPGPGLGVRVLG.EVKKEYCDLLRRADAIFIEELRK...................ADLYDKVSQAFTVFL.PVRSVG
GUAA_ECO5E GLELGLPYDMLYRHPFPGPGLGVRVLG.EVKKEYCDLLRRADAIFIEELRK...................ADLYDKVSQAFTVFL.PVRSVG
GUAA_ECO81 GLELGLPYDMLYRHPFPGPGLGVRVLG.EVKKEYCDLLRRADAIFIEELRK...................ADLYDKVSQAFTVFL.PVRSVG
GUAA_ECO8A GLELGLPYDMLYRHPFPGPGLGVRVLG.EVKKEYCDLLRRADAIFIEELRK...................ADLYDKVSQAFTVFL.PVRSVG
GUAA_ECOHS GLELGLPYDMLYRHPFPGPGLGVRVLG.EVKKEYCDLLRRADAIFIEELRK...................ADLYDKVSQAFTVFL.PVRSVG
GUAA_ECOK1 GLELGLPYDMLYRHPFPGPGLGVRVLG.EVKKEYCDLLRRADAIFIEELRK...................ADLYDKVSQAFTVFL.PVRSVG
GUAA_ECOL5 GLELGLPYDMLYRHPFPGPGLGVRVLG.EVKKEYCDLLRRADAIFIEELRK...................ADLYDKVSQAFTVFL.PVRSVG
GUAA_ECOL6 GLELGLPYDMLYRHPFPGPGLGVRVLG.EVKKEYCDLLRRADAIFIEELRK...................ADLYDKVSQAFTVFL.PVRSVG
GUAA_ECOLC GLELGLPYDMLYRHPFPGPGLGVRVLG.EVKKEYCDLLRRADAIFIEELRK...................ADLYDKVSQAFTVFL.PVRSVG
GUAA_ECOSE GLELGLPYDMLYRHPFPGPGLGVRVLG.EVKKEYCDLLRRADAIFIEELRK...................ADLYDKVSQAFTVFL.PVRSVG
GUAA_ECOSM GLELGLPYDMLYRHPFPGPGLGVRVLG.EVKKEYCDLLRRADAIFIEELRK...................ADLYDKVSQAFTVFL.PVRSVG
GUAA_ECOUT GLELGLPYDMLYRHPFPGPGLGVRVLG.EVKKEYCDLLRRADAIFIEELRK...................ADLYDKVSQAFTVFL.PVRSVG
GUAA_ECO24 GLELGLPYDMLYRHPFPGPGLGVRVLG.EVKKEYCDLLRRADAIFIEELRK...................ADLYDKVSQAFTVFL.PVRSVG
GUAA_SHISS GLELGLPYDMLYRHPFPGPGLGVRVLG.EVKKEYCDLLRRADAIFIEELRK...................ADLYDKVSQAFTVFL.PVRSVG
GUAA_SHIF8 GLELGLPYDMLYRHPFPGPGLGVRVLG.EVKKEYCDLLRRADAIFIEELRK...................ADLYDKVSQAFTVFL.PVRSVG
GUAA_SHIFL GLELGLPYDMLYRHPFPGPGLGVRVLG.EVKKEYCDLLRRADAIFIEELRK...................ADLYDKVSQAFTVFL.PVRSVG
GUAA_SHIDS GLELGLPYDMLYRHPFPGPGLGVRVLG.EVKKEYCDLLRRVDAIFIEELRK...................ADLYDKVSQAFTVFL.PVRSVG
GUAA_ECOLI GLELGLPYDMLYRHPFPGPGLGVRVLG.EVKKEYCDLLRRADAIFIEELRK...................ADLYDKVSQAFTVFL.PVRSVG
GUAA_ECODH GLELGLPYDMLYRHPFPGPGLGVRVLG.EVKKEYCDLLRRADAIFIEELRK...................ADLYDKVSQAFTVFL.PVRSVG
GUAA_ECOBW GLELGLPYDMLYRHPFPGPGLGVRVLG.EVKKEYCDLLRRADAIFIEELRK...................ADLYDKVSQAFTVFL.PVRSVG
GUAA_ESCF3 GLELGLPYDMLYRHPFPGPGLGVRVLG.EVKKEYCDLLRRADAIFIEELRK...................ADLYDKVSQAFTVFL.PVRSVG
GUAA_ECO27 GLELGLPYDMLYRHPFPGPGLGVRVLG.EVKKEYCDLLRRADAIFIEELRK...................ADLYDKVSQAFTVFL.PVRSVG
GUAA_SHIB3 GLELGLPYDMLYRHPFPGPGLGVRVLG.EVKKEYCDLLRRADAIFIEELRK...................ADLYNKVSQAFTVFL.PVRSVG
GUAA_SHIBS GLELGLPYDMLYRHPFPGPGLGVRVLG.EVKKEYCDLLRRADAIFIEELRK...................ADLYDKVSQAFTVFL.PVRSVG
GUAA_ECOLU GLELGLPYDMLYRHPFPGPGLGVRVLG.EVKKEYCDLLRRADAIFIEELRK...................ADLYDKVSQAFTVFL.PVRSVG
GUAA_SALPK GLELGLPYDMLYRHPFPGPGLGVRVLG.EVKKEYCDLLRRADAIFIEELRK...................ADLYDKVSQAFTVFL.PVRSVG
GUAA_SALPC GLELGLPYDMLYRHPFPGPGLGVRVLG.EVKKEYCDLLRRADAIFIEELRK...................ADLYDKVSQAFTVFL.PVRSVG
GUAA_SALPB GLELGLPYDMLYRHPFPGPGLGVRVLG.EVKKEYCDLLRRADAIFIEELRK...................ADLYDKVSQAFTVFL.PVRSVG
GUAA_SALPA GLELGLPYDMLYRHPFPGPGLGVRVLG.EVKKEYCDLLRRADAIFIEELRK...................ADLYDKVSQAFTVFL.PVRSVG
GUAA_SALCH GLELGLPYDMLYRHPFPGPGLGVRVLG.EVKKEYCDLLRRADAIFIEELRK...................ADLYDKVSQAFTVFL.PVRSVG
GUAA_SALTY GLELGLPYDMLYRHPFPGPGLGVRVLG.EVKKEYCDLLRRADAIFIEELRK...................ADLYDKVSQAFTVFL.PVRSVG
GUAA_SALSV GLELGLPYDMLYRHPFPGPGLGVRVLG.EVKKEYCDLLRRADAIFIEELRK...................ADLYDKVSQAFTVFL.PVRSVG
GUAA_SALNS GLELGLPYDMLYRHPFPGPGLGVRVLG.EVKKEYCDLLRRADAIFIEELRK...................ADLYDKVSQAFTVFL.PVRSVG
GUAA_SALTI GLELGLPYDMLYRHPFPGPGLGVRVLG.EVKKEYCDLLRRADAIFIEELRK...................ADLYDKVSQAFTVFL.PVRSVG
GUAA_SALHS GLELGLPYDMLYRHPFPGPGLGVRVLG.EVKKEYCDLLRRADAIFIEELRK...................ADLYDKVSQAFTVFL.PVRSVG
GUAA_SALG2 GLELGLPYDMLYRHPFPGPGLGVRVLG.EVKKEYCDLLRRADAIFIEELRK...................ADLYDKVSQAFTVFL.PVRSVG
GUAA_SALEP GLELGLPYDMLYRHPFPGPGLGVRVLG.EVKKEYCDLLRRADAIFIEELRK...................ADLYDKVSQAFTVFL.PVRSVG
GUAA_SALDC GLELGLPYDMLYRHPFPGPGLGVRVLG.EVKKEYCDLLRRADAIFIEELRK...................ADLYDKVSQAFTVFL.PVRSVG
GUAA_SALA4 GLELGLPYDMLYRHPFPGPGLGVRVLG.EVKKEYCDLLRRADAIFIEELRK...................ADLYDKVSQAFTVFL.PVRSVG
GUAA_SALAR GLELGLPYDMLYRHPFPGPGLGVRVLG.EVKKEYCDLLRRADAIFIEELRK...................ADLYDKVSQAFTVFL.PVRSVG
GUAA_KLEP7 GLELGLPYDMLYRHPFPGPGLGVRVLG.EVKKEYCDLLRRADAIFIEELHK...................ADLYNKVSQAFTVFL.PVRSVG
GUAA_KLEP3 GLELGLPYDMLYRHPFPGPGLGVRVLG.EVKKEYCDLLRRADAIFIEELHK...................ADLYNKVSQAFTVFL.PVRSVG
GUAA_ENT38 GLELGLPYDMLYRHPFPGPGLGVRVLG.EVKKEYCDLLRRADAIFIEELHK...................ADLYNKVSQAFTVFL.PVRSVG
GUAA_CITK8 GLELGLPYDMLYRHPFPGPGLGVRVLG.EVKKEYCDLLRRADAIFIEELHK...................ADLYNKVSQAFTVFL.PVRSVG
GUAA_CROS8 GLELGLPYDMLYRHPFPGPGLGVRVLG.EVKKEYCDLLRRADAIFIEELRR...................ADLYDKVSQAFAVFL.PVRSVG
GUAA_SODGM GLELGLPYDMLYRHPFPGPGLGVRVLG.EVKKEYCDLLRRADAIFIEELYK...................ADLYNKVSQAFTVFL.PVRSVG
GUAA_PECCP GLELGLPYNMLYRHPFPGPGLGVRVLG.EVKKEYCDLLRRADAIFIEELHK...................ADLYNKVSQAFTVFL.PVRSVG
GUAA_PECAS GLELGLPYNMLYRHPFPGPGLGVRVLG.EVKKEYCDLLRRADAIFIEELHK...................ADLYNKVSQAFTVFL.PVRSVG
GUAA_YERPY GLELGLPYDMLYRHPFPGPGLGVRVLG.EVKKEYCDLLRRADAIFIEELHK...................ADLYNKVSQAFTVFL.PVRSVG
GUAA_YERPS GLELGLPYDMLYRHPFPGPGLGVRVLG.EVKKEYCDLLRRADAIFIEELHK...................ADLYNKVSQAFTVFL.PVRSVG
GUAA_YERPB GLELGLPYDMLYRHPFPGPGLGVRVLG.EVKKEYCDLLRRADAIFIEELHK...................ADLYNKVSQAFTVFL.PVRSVG
GUAA_YERP3 GLELGLPYDMLYRHPFPGPGLGVRVLG.EVKKEYCDLLRRADAIFIEELHK...................ADLYNKVSQAFTVFL.PVRSVG
GUAA_YERPP GLELGLPYDMLYRHPFPGPGLGVRVLG.EVKKEYCDLLRRADAIFIEELHK...................ADLYNKVSQAFTVFL.PVRSVG
GUAA_YERPN GLELGLPYDMLYRHPFPGPGLGVRVLG.EVKKEYCDLLRRADAIFIEELHK...................ADLYNKVSQAFTVFL.PVRSVG
GUAA_YERPG GLELGLPYDMLYRHPFPGPGLGVRVLG.EVKKEYCDLLRRADAIFIEELHK...................ADLYNKVSQAFTVFL.PVRSVG
GUAA_YERPE GLELGLPYDMLYRHPFPGPGLGVRVLG.EVKKEYCDLLRRADAIFIEELHK...................ADLYNKVSQAFTVFL.PVRSVG
GUAA_YERPA GLELGLPYDMLYRHPFPGPGLGVRVLG.EVKKEYCDLLRRADAIFIEELHK...................ADLYNKVSQAFTVFL.PVRSVG
GUAA_YERE8 GLELGLPYDMLYRHPFPGPGLGVRVLG.EVKKEYCDLLRRADAIFIEELHK...................ADLYNKVSQAFTVFL.PVRSVG
GUAA_SERP5 GLELGLPYDMLFRHPFPGPGLGVRVLG.EVKKEYCDLLRRADAIFIEELHK...................ADLYNKVSQAFTVFL.PVRSVG
GUAA_PHOLL GLELGLPYDMLNRHPFPGPGLGVRVLG.EVKKEYCDLLRQADAIFIEELHK...................ADLYNKVSQAFTVFL.PVRSVG
GUAA_PROMH GLELGLPYDMLYRHPFPGPGLGVRVLG.EIKKEYCDLLRRADAIFIEELHK...................ADLYNKVSQAFTVFL.PVRSVG
GUAA_EDWI9 GLELGLPYDMLYRHPFPGPGLGVRVLG.EVKKEYCDLLRRADAIFIEELHK...................ADLYNKVSQAFTVFL.PVRSVG
GUAA_AERHH GLELGLPYDMLYRHPFPGPGLGVRVLG.EVKKEYCDLLRKADAVFIEELRK...................ADLYNQVSQAFAVFL.PVRSVG
GUAA_AERS4 GLELGLPYDMLYRHPFPGPGLGVRVLG.EVKKEYCDILRRADAVFIEELRK...................ADLYNQVSQAFAVFL.PVRSVG
GUAA_TOLAT GLELGLPYNMLYRHPFPGPGLGVRVLG.EVKKEYCDLLRRADAIFIEELHN...................ADLYNKVSQAFTVFL.PVRSVG
GUAA_VIBF1 GLELGLPYNMLYRHPFPGPGLGVRVLG.EIKKEYCDLLRRADAIFIEELHN...................ADLYNKVSQAFTVFL.PVRSVG
GUAA_VIBFM GLELGLPYNMLYRHPFPGPGLGVRVLG.EIKKEYCDLLRRADAIFIEELHN...................ADLYNKVSQAFTVFL.PVRSVG
GUAA_ALISL GLELGLPYNMLYRHPFPGPGLGVRVLG.EVKKEYCDLLRRADAIFIEELHN...................ADLYNKVSQAFTVFL.PVRSVG
GUAA_VIBSL GLELGLPYNMLYRHPFPGPGLGVRVLG.EVKKEYCDLLRRADAIFIEELHA...................ADLYHKVSQAFTVFL.PVRSVG
GUAA_VIBCM GLELGLPYNMLYRHPFPGPGLGVRVLG.EVKKEYCDLLRRADAIFIEELHA...................ADLYNKVSQAFTVFL.PVRSVG
GUAA_VIBCH GLELGLPYNMLYRHPFPGPGLGVRVLG.EVKKEYCDLLRRADAIFIEELHA...................ADLYNKVSQAFTVFL.PVRSVG
GUAA_VIBC3 GLELGLPYNMLYRHPFPGPGLGVRVLG.EVKKEYCDLLRRADAIFIEELHA...................ADLYNKVSQAFTVFL.PVRSVG
GUAA_VIBVU GLELGLPYEMLYRHPFPGPGLGVRVLG.EIKKEYCDLLRRADAIFIEELHA...................ADLYNKVSQAFTVFL.PVRSVG
GUAA_VIBVY GLELGLPYEMLYRHPFPGPGLGVRVLG.EIKKEYCDLLRRADAIFIEELHA...................ADLYNKVSQAFTVFL.PVRSVG
GUAA_VIBPA GLELGLPYNMLYRHPFPGPGLGVRVLG.EIKKEYCDLLRRADAIFIEELHA...................ADLYDKVSQAFTVFL.PVRSVG
GUAA_VIBCB GLELGLPYNMLYRHPFPGPGLGVRVLG.EIKKEYCDLLRRADAIFIEELHA...................ADLYNKVSQAFTVFL.PVRSVG
GUAA_PHOPR GLELGLPYNMLYRHPFPGPGLGVRVLG.EVKKEYCDLLRRADAIFIEELHS...................ADLYNKVSQAFTVFL.PVRSVG
GUAA_COLP3 GLELGLPYDMLYRHPFPGPGLGVRILG.EVKKEYADLLRRADAIFIEELHK...................HDLYTKVSQAFTVFL.PVKSVG
GUAA_PSYIN GLELGLPYEMLYRHPFPGPGLGVRVLG.EVKKEYCDLLRRADAIFIEELHK...................ADLYHKVSQAFVVFL.PVRSVG
GUAA_PSEHT GLELGLPYDMLYRHPFPGPGLGVRVLG.EIKKEYCDLLRRADAIFIEELHK...................AELYHKVSQAFTVFL.PVKSVG
GUAA_PSEA6 GLELGLPYDMLYRHPFPGPGLGVRVLG.EVKKEYCDLLRRADAIFIDELHK...................HDLYNKVSQAFTVFL.PVKSVG
GUAA_ALTMD GLELGLPYDMLYRHPFPGPGLGVRVLG.EIKKEYCDLLRRADAIFIEELYA...................ADLYQKVSQAFTVFL.PVRSVG
GUAA_SHESW GLELGLPYNMLYRHPFPGPGLGVRVLG.EVKKEYCDLLRRADAIFIEELHK...................ADLYNKVSQAFTVFL.PVRSVG
GUAA_SHEPC GLELGLPYNMLYRHPFPGPGLGVRVLG.EVKKEYCDLLRRADAIFIEELHK...................ADLYNKVSQAFTVFL.PVRSVG
GUAA_SHEB9 GLELGLPYNMLYRHPFPGPGLGVRVLG.EVKKEYCDLLRRADAIFIEELHK...................ADLYNKVSQAFTVFL.PVRSVG
GUAA_SHEB8 GLELGLPYNMLYRHPFPGPGLGVRVLG.EVKKEYCDLLRRADAIFIEELHK...................ADLYNKVSQAFTVFL.PVRSVG
GUAA_SHEB5 GLELGLPYNMLYRHPFPGPGLGVRVLG.EVKKEYCDLLRRADAIFIEELHK...................ADLYNKVSQAFTVFL.PVRSVG
GUAA_SHEB2 GLELGLPYNMLYRHPFPGPGLGVRVLG.EVKKEYCDLLRRADAIFIEELHK...................ADLYNKVSQAFTVFL.PVRSVG
GUAA_SHESM GLELGLPYNMLYRHPFPGPGLGVRVLG.EVKKEYCDLLRRADAIFIEELHK...................ADLYNKVSQAFTVFL.PVRSVG
GUAA_SHESA GLELGLPYNMLYRHPFPGPGLGVRVLG.EVKKEYCDLLRRADAIFIEELHK...................ADLYNKVSQAFTVFL.PVRSVG
GUAA_SHESR GLELGLPYNMLYRHPFPGPGLGVRVLG.EVKKEYCDLLRRADAIFIEELHK...................ADLYNKVSQAFTVFL.PVRSVG
GUAA_SHEON GLELGLPYNMLYRHPFPGPGLGVRVLG.EVKKEYCDLLRRADAIFIEELHK...................ADLYNKVSQAFTVFL.PVRSVG
GUAA_SHEDO GLELGLPYNMLYRHPFPGPGLGVRVLG.EVKKEYCDLLRRADAIFIEELHK...................ADLYNKVSQAFTVFL.PVRSVG
GUAA_SHEPW GLELGLPYDMLYRHPFPGPGLGVRVLG.EVKKEYCDLLRLADAIFIEELHK...................ADLYNKVSQAFTVFL.PVRSVG
GUAA_SHESH GLELGLPYDMLYRHPFPGPGLGVRVLG.EVKKEYCDLLRLADAIFIEELHK...................AELYHKVSQAFTVFL.PVRSVG
GUAA_SHEWM GLELGLPYDMLYRHPFPGPGLGVRVLG.EVKKEYCDLLRLADAIFIEELHK...................ADLYNKVSQAFTVFL.PVRSVG
GUAA_SHEHH GLELGLPYNMLYRHPFPGPGLGVRVLG.EVKKEYCDLLRLADAIFIEELHK...................ADLYNKVSQAFTVFL.PVRSVG
GUAA_SHEPA GLELGLPYDMLYRHPFPGPGLGVRVLG.EVKKEYCDLLRLADAIFIEELHK...................ADLYNKVSQAFTVFL.PVRSVG
GUAA_SHEFN GLELGLPYNMLYRHPFPGPGLGVRVLG.EVKKEYCDLLRSADAIFIEELHK...................ADLYNKVSQAFTVFL.PVRSVG
GUAA_SHELP GLELGLPYNMLYRHPFPGPGLGVRVLG.EVKKEYCDLLRRADAIFIEELHK...................ADLYNKVSQAFTVFL.PVRSVG
GUAA_SHEAM GLELGLPYDMLYRHPFPGPGLGVRVLG.EVKKEYCDLLRRADAIFIEELHR...................ADLYNKVSQAFTVFL.PVRSVG
GUAA_IDILO GLELGLPYNMLYRHPFPGPGLGVRVLG.EIKKEYCDLLRRADAIFIEELHN...................ADWYNKVSQAFAVFL.PVRSVG
GUAA_ACTSZ GLALGLPAEMLNRHPFPGPGLGVRVLG.EIKKEYCDLLRKADAIFIEELYN...................SGWYYKVSQAFTVFL.PVKSVG
GUAA_MANSM GLALGLPAEMLNRHPFPGPGLGVRVLG.EIKKEYCDLLRKADAIFIEELYN...................SGWYYKVSQAFTVFL.PVKSVG
GUAA_ACTPJ GLALGLPAEMLNRHPFPGPGLGVRVLG.EIKKEYCDLLRKADAIFIEELYK...................ADWYYKVSQAFTVFL.PVKSVG
GUAA_ACTP7 GLALGLPAEMLNRHPFPGPGLGVRVLG.EIKKEYCDLLRKADAIFIEELYK...................ADWYYKVSQAFTVFL.PVKSVG
GUAA_ACTP2 GLALGLPAEMLNRHPFPGPGLGVRVLG.EIKKEYCDLLRKADAIFIEELYK...................ADWYYKVSQAFTVFL.PVKSVG
GUAA_HAEPS GLALGLPAEMLNRHPFPGPGLGVRVLG.EIKKEYCDLVRRADAIFMEELHA...................SGWYYKVSQAFTVFL.PVKSVG
GUAA_PASMU GLALGLPAEMLNRHPFPGPGLGVRVLG.EIKKEYCDLVRLADAIFMEELHA...................SGWYYKVSQAFTVFL.PVKSVG
GUAA_HAEDU GLTLGLPAEMLNRHPFPGPGLGVRVLG.EIKKEYCDLLRNADAIFIEELYK...................SGWYYKVSQAFTVFL.PVKSVG
GUAA_HISS1 GLALGLPAEMLNRHPFPGPGLGVRVLG.EIKKEYCDLLRKADAIFIEELHK...................ADWYYKVSQAFSVFL.PVKSVG
GUAA_HISS2 GLALGLPAEMLNRHPFPGPGLGVRVLG.EIKKEYCDLLRKADAIFIEELHK...................ADWYYKVSQAFGVFL.PVKSVG
GUAA_HAEI8 GLALGLPAEMINRHPFPGPGLGVRVLG.EVKKEYCDLLRRADAIFIEELRN...................SGWYEKTSQAFSVFL.PVKSVG
GUAA_HAEIG GLALGLPAEMINRHPFPGPGLGVRVLG.EVKKEYCDLLRRADAIFIEELRN...................SGWYEKTSQAFSVFL.PVKSVG
GUAA_HAEIN GLALGLPAEMINRHPFPGPGLGVRVLG.EVKKEYCDLLRRADAIFIEELRN...................SGWYEKTSQAFSVFL.PVKSVG
GUAA_HAEIE GLALGLPAEMINRHPFPGPGLGVRVLG.EVKKEYCDLLRRADAIFIEELRN...................SGWYEKTSQAFSVFL.PVKSVG
GUAA_HAMD5 GLELGLPNAMLHRHPFPGPGLGVRVLG.EVKQEYCDLLRQADAIFIQELQK...................AELYQKVSQAFAVFL.PIRSVG
GUAA_XANC5 GVELGLPRTMVYRHPFPGPGLGVRILG.EVKREYAELLAKADAIFIDELRK...................ADLYDKTSQAFAVFL.PVKSVG
GUAA_XANAC GVELGLPRTMVYRHPFPGPGLGVRILG.EVKREYAELLAKADAIFIDELRK...................ADLYDTTSQAFAVFL.PVKSVG
GUAA_XANCP GVELGLPRTMVYRHPFPGPGLGVRILG.EVKREYAELLAKADAIFIDELRK...................ADLYDKTSQAFAVFL.PVKSVG
GUAA_XANCB GVELGLPRTMVYRHPFPGPGLGVRILG.EVKREYAELLAKADAIFIDELRK...................ADLYDKTSQAFAVFL.PVKSVG
GUAA_XANC8 GVELGLPRTMVYRHPFPGPGLGVRILG.EVKREYAELLAKADAIFIDELRK...................ADLYDKTSQAFAVFL.PVKSVG
GUAA_XANOR GVELGLPRTMVYRHPFPGPGLGVRILG.EVKREYAELLAKADAIFIDELRK...................ADLYDKISQAFAVFL.PVKSVG
GUAA_XANOM GVELGLPRTMVYRHPFPGPGLGVRILG.EVKREYAELLAKADAIFIDELRK...................ADLYDKISQAFAVFL.PVKSVG
GUAA_XANOP GVELGLPRTMVYRHPFPGPGLGVRILG.EVKREYAELLAKADAIFIDELRK...................ADLYDKISQAFAVFL.PVKSVG
GUAA_STRMK GVELGLPRTMVYRHPFPGPGLGVRILG.EVKREYAELLAKADAIFIDELRK...................ADLYDKTSQAFAVFL.PVKSVG
GUAA_STRM5 GVELGLPRTMVYRHPFPGPGLGVRILG.EVKREYAELLAKADAIFIDELRK...................ADLYDKTSQAFAVFL.PVKSVG
GUAA_XYLFT GVALGLPHAMVYRHPFPGPGLGVRILG.EVKPEYAELLAKADSIFIDELHQ...................ADLYDKVSQAFAVFL.PVKSVG
GUAA_XYLF2 GVALGLPHAMVYRHPFPGPGLGVRILG.EVKPEYAELLAKADSIFIDELHQ...................ADLYDKVSQAFAVFL.PVKSVG
GUAA_XYLFM GVALGLPHAMVYRHPFPGPGLGVRILG.EVKPEYAELLAKADSIFIDELHQ...................ADLYDKVSQAFAVFL.PVKSVG
GUAA_XYLFA GVALGLPHAMVYRHPFPGPGLGVRILG.EVKPEYAELLAKADAIFIDELHQ...................ADLYDKVSQAFAVFL.PVKSVG
GUAA_CELJU GLELGLPYDMVYRHPFPGPGLGVRILG.EVKKEYADILREADAIFLEELRA...................ADWYHKTSQAFAVFL.PVKSVG
GUAA_TERTT GLELGLPYDMVYRHPFPGPGLGVRILG.EVKKEYADILREADAIFIEELRN...................ADWYHKTSQAFAVFL.PVKSVG
GUAA_HAHCH GLELGLPYDMVYRHPFPGPGLGVRILG.EVKKEYADILREADAIFIEELHR...................AELYHKVSQAFAVFL.PVKSVG
GUAA_MARAV GLELGLPYDMVYRHPFPGPGLGVRILG.EVKKEYADILRRADAIFLEELHR...................ADLYHKTSQAFAVFL.PVKSVG
GUAA_PSESM GLELGLPYDMVYRHPFPGPGLGVRILG.EVKKEYADILRRADHIFIEELRK...................ADWYHKVSQAFVVFQ.PVKSVG
GUAA_PSE14 GLELGLPYDMVYRHPFPGPGLGVRILG.EVKKEYADLLRRADHIFIEELRK...................ADWYHKVSQAFVVFQ.PVKSVG
GUAA_PSEU2 GLELGLPYDMVYRHPFPGPGLGVRILG.EVKKEYADLLRRADHIFIEELRK...................ADWYHKVSQAFVVFQ.PVKSVG
GUAA_PSEFS GLELGLPYDMVYRHPFPGPGLGVRILG.EVKKEYADLLRRADHIFIEELRK...................ADWYHKVSQAFVVFQ.PVKSVG
GUAA_PSEF5 GLELGLPYDMVYRHPFPGPGLGVRILG.EVKKEYADLLRRADHIFIEELRK...................ADWYHKVSQAFVVFQ.PVKSVG
GUAA_PSEPF GLELGLPYDMVYRHPFPGPGLGVRILG.EVKKEYADLLRRADHIFIEELRK...................ADWYHKVSQAFVVFQ.PVKSVG
```

```
GUAA_PSEPK    GLELGLPYDMVYRHPFPGPGLGVRILG.EVKKEYADILRRADHIFIEELRK...................ADWYHKTSQAFVVFQ.PVKSVG
GUAA_PSEP1    GLELGLPYDMVYRHPFPGPGLGVRILG.EVKKEYADILRRADHIFIEELRK...................ADWYHKTSQAFVVFQ.PVKSVG
GUAA_PSEPG    GLELGLPYDMVYRHPFPGPGLGVRILG.EVKKEYADILRRADHIFIEELRK...................ADWYHKTSQAFVVFQ.PVKSVG
GUAA_PSEE4    GLELGLPYDMVYRHPFPGPGLGVRILG.EVKKEYADILRRADHIFIEELRK...................ADWYHKTSQAFVVFQ.PVKSVG
GUAA_PSEPW    GLELGLPYDMVYRHPFPGPGLGVRILG.EVKKEYADILRRADHIFIEELRK...................ADWYHKTSQAFVVFQ.PVKSVG
GUAA_PSEMY    GLELGLPYDMVYRHPFPGPGLGVRILG.EVKKEYADLLRRADHIFIEELRK...................ADWYHKTSQAFVVFQ.PVKSVG
GUAA_PSEAE    GLELGLPYDMVYRHPFPGPGLGVRILG.EVKKEYADLLRQADHIFIEELRA...................FDWYHKTSQAFVVFQ.PVKSVG
GUAA_PSEA8    GLELGLPYDMVYRHPFPGPGLGVRILG.EVKKEYADLLRQADHIFIEELRA...................FDWYHKTSQAFVVFQ.PVKSVG
GUAA_PSEAB    GLELGLPYDMVYRHPFPGPGLGVRILG.EVKKEYADLLRQADHIFIEELRA...................FDWYHKTSQAFVVFQ.PVKSVG
GUAA_MARMS    GLELGLPYDMVYRHPFPGPGLGVRILG.EVKKEYADILRRADAIFIEELRN...................AGWYEKTSQAFAVFL.PVKSVG
GUAA_ALCBS    GLELGLPYDMVYRHPFPGPGLGVRILG.EVKKEYADTLRLADHIFIEELRA...................AGLYHKTSQAFAVFL.PVKSVG
GUAA_THICR    GVALGLPSDMVYRHPFPGPGLGVRILG.EVKKEYADILRLADHIFIEELRA...................ADLYDKTSQAFTVFL.PVKSVG
GUAA_ALKEH    GLELGLPADMVYRHPFPGPGLGVRILG.EVRKEYADLLREADAIFIEELRA...................HDLYDQVSQAFAVFL.PVKSVG
GUAA_METCA    GLELGLPYEMVYRHPFPGPGLGVRILG.EVRKEYADLLRRADAIFIEELYR...................HDLYDKVSQAFAVFL.PVKSVG
GUAA_NITOC    GTELGLPFELVYRHPFPGPGLGVRILG.EVKKEYADLLRLADAIFIEELHA...................HDWYDKVSQAFAVFL.PVKSVG
GUAA_LEGPL    GLELGLPHDMVYRHPFPGPGLGVRILA.EVKKEYADILRKADAIFIEELHN...................AQLYHKISQAFAVFL.PVKSVG
GUAA_LEGPA    GLELGLPHDMVYRHPFPGPGLGVRILA.EVKKEYADILRKADAIFIEELHN...................AQLYHKISQAFAVFL.PVKSVG
GUAA_LEGPC    GLELGLPHDMVYRHPFPGPGLGVRILA.EVKKEYADILRKADAIFIEELHN...................AQLYHKISQAFAVFL.PVKSVG
GUAA_LEGPH    GLELGLPHDMVYRHPFPGPGLGVRILA.EVKKEYADILRKADAIFIEELHN...................AQLYHKISQAFAVFL.PVKSVG
GUAA_ACIAD    GTTLGLPHSMIYRHPFPGPGLGVRILG.EVKKEYADILRLADDIFMQELRA...................SGWYDKTAQAFAVFQ.PVKSVG
GUAA_PSYA2    GITLGLPAKMINRHPFPGPGLGVRILG.EVTKEFADILRSADAIFMEELEK...................SGWYEKTAQAFAVFQ.PIKSVG
GUAA_RUTMC    SVKLSIPPHIIYRHPFPGPGLGVRILG.QVKQEYANILRQADAIFMDELHK...................NNLYDTVNQAFAVFL.PIKSVG
GUAA_FRAP2    GLGLGLPYNMLYRHPFPGPGLGVRILG.EIKKEYVETLQKADAIFTEELYK...................HNLYHDISQAFGVFL.PVKSVG
GUAA_BURPP    GVKLGLPPAMVYRHPFPGPGLGVRILG.EVKRDFADLLRRADAIFIETLRT...FIDKETG.........KSWYDLTSQAFAVFL.PVKSVG
GUAA_BURXL    GVKLGLPPAMVYRHPFPGPGLGVRILG.EVKRDFADLLRRADAIFIETLRT...FIDKETG.........KSWYDLTSQAFAVFL.PVKSVG
GUAA_BURP8    GVKLGLPPSMVYRHPFPGPGLGVRILG.EVKREYADLLRRADAIFIESLRT...FIDKDTD.........KSWYDLTSQAFAVFL.PVKSVG
GUAA_POLNS    GVALGLPREMVYRHPFPGPGLGVRILG.EVKAEFASLLQRADAIFIEELRN...TIDEVSQ.........KSWYDLTSQAFAVFL.PVKSVG
GUAA_POLSQ    GVALGLPREMVYRHPFPGPGLGVRILG.EVKAEFASLLQRADAIFIEELRN...TIDEVSQ.........KSWYDLTSQAFAVFL.PVKSVG
GUAA_DECAR    GIALGLPREMVYRHPFPGPGLGVRILG.EVTLKAAHLLQRADAIFIDELRATHATERDVAAGACTEADIGKSWYDLTSQAFAVFL.PVKSVG
GUAA_HERAR    GVALGLPHDMVYRHPFPGPGLGVRILG.EVKKDFADLLRRADAIFIEELRNTPYVLAAGASESTDNGIPHRNWYDATSQAFAVFL.PVKSVG
GUAA_JANMA    GVALGLPHDMVYRHPFPGPGLGVRILG.EVKKEFADLLRRADAIFIEELRKTPFVATPGASEATDNGIPHRNWYDATSQAFAVFL.PVKSVG
GUAA_AROAE    GIALGLPHEMVYRHPFPGPGLGVRILG.EVKQEFAELLRRADAIFIDELR.........AA..........DWYDKTSQAFAVFL.PVKSVG
GUAA_AZOSB    GVALGLPHDMVYRHPFPGPGLGVRILG.EVKKEFADLLRRADAIFIDELR.........AA..........DWYDKTSQAFAVFL.PVKSVG
GUAA_METFK    GIALGLPHDMVYRHPFPGPGLGVRILG.EVKREYADLLRQADHIFIEELRS...TKDADGK..........SWYDKTSQAFAVFL.PVKSVG
GUAA_THIDA    GIALGLPHDMVYRHPFPGPGLGVRILG.KVKREYADLLRRADAIFIEELR.......ASG............WYEKTSQAFTVFL.PVKSVG
GUAA_NITEC    GLALGLPRDLVFRHPFPGPGLGVRILG.EVKYEYTELLRQADAIFIEELRN........AG...........WYEKTSQAFAVFL.PIKSVG
GUAA_NITEU    GLALGLPHDLVFRHPFPGPGLGVRILG.EVKYEYTELLRQADAIFIEELRN........AG...........WYEKTSQAFAVFL.PIKSVG
GUAA_NITMU    GLTLGLPHDMVFRHPFPGPGLGVRILG.EVKREYAELLRRADAIFIEELRN........AG...........WYEKTSQAFAVFL.PVKSVG
GUAA_NEIMA    GVALGLPREMVYRHPFPGPGLGVRILG.EVKKEYADLLRQADDIFIQELRN....TTDENG.........TSWYDLTSQAFAVFL.PVKSVG
GUAA_NEIMF    GVALGLPREMVYRHPFPGPGLGVRILG.EVKKEYADLLRQADDIFIQELRN....TTDENG.........TSWYDLTSQAFAVFL.PVKSVG
GUAA_NEIG1    GVALGLPREMVYRHPFPGPGLGVRILG.EVKKEYADLLRQADDIFIQELRN....TTDENG.........TSWYDLTSQAFAVFL.PVKSVG
GUAA_NEIG2    GVALGLPREMVYRHPFPGPGLGVRILG.EVKKEYADLLRQADDIFIQELRN....TTDENG.........TSWYDLTSQAFAVFL.PVKSVG
GUAA_NEIM0    GVALGLPREMVYRHPFPGPGLGVRILG.EVKKEYADLLRQADDIFIQELRN....TTDENG.........TSWYDLTSQAFAVFL.PVKSVG
GUAA_NEIMB    GVALGLPREMVYRHPFPGPGLGVRILG.EVKKEYADLLRQADDIFIQELRN....TTDENG.........TSWYDLTSQAFAVFL.PVKSVG
GUAA_GEOMG    GEELGLPHQMVWRHPFPGPGLGVRILG.EVKKEYADILRRADAIYIEELYA...................AGHYDKISQAFAVFL.PVKSVG
GUAA_GEOSL    GEELGLPRQMVWRHPFPGPGLGVRILG.EVKKEYADILRRADAIYIEELYA...................AGHYDKISQAFAVFL.PVKSVG
GUAA_GEODF    GEELGLPHQMVWRHPFPGPGLGVRILG.EVRKDYADILRQADAIYIEELYA...................ADHYHKISQAFAVFL.PVKSVG
GUAA_PELPD    GEELGLPHQMVWRHPFPGPGLAVRILG.EVKKEYADILRRADAIFIEELYS...................SDHYHKISQAFAVFL.PVKSVG
GUAA_PELCD    GEELGLPHPMVYRHPFPGPGLGVRILG.EVKPEYADILRRADAIYIEELYR...................TGHYDKISQAFAVFL.PVKSVG
GUAA_BARHE    GKELGLPEQFIGRHPFPGPGLAIRCPG.AVTREKLEILREADAIYLDEIRK...................AGLYDEIWQAFAILL.PVQTVG
GUAA_BARQU    GRELGLPEQFLGRHPFPGPGLAIRCPG.AVTREKLEIIREADAIYLDEICK...................AGLYDEIWQAFVVLL.PVQTVG
GUAA_BARBK    GRELGLPEEFIKRHPFPGPGLAIRCPG.AVTREKIEILREADAIYLDEIRK...................AGLYDKIWQAFAILL.PVQTVG
GUAA_RHIE6    GRELGLPDSFIGRHPFPGPGLAIRCPG.GISREKLEILREADAIYLDEIRK...................AGLYDAIWQAFAVLL.PVQTVG
GUAA_RHILW    GRELGLPDSFIGRHPFPGPGLAIRCPG.GISREKLEILREADAIYLDEIRK...................AGLYDAIWQAFAVLL.PVQTVG
GUAA_RHIEC    GRELGLPDSFIGRHPFPGPGLAIRCPG.GITREKLEILREADAIYLDEIRK...................AGLYDAIWQAFAVLL.PVQTVG
GUAA_RHIL3    GRELGLPDSFIGRHPFPGPGLAIRCPG.GITREKLEILREADAIYLDEIRK...................AGLYDAIWQAFAVLL.PVQTVG
GUAA_AGRRK    GKELGLPDSFIGRHPFPGPGLAIRCPG.GITREKLEILREADAIYLDEIRK...................AGLYDAIWQAFAVLL.PVQTVG
GUAA_RHISN    GRELGLPESFIGRHPFPGPGLAIRCPG.GISREKLEILREADAIYLDEIRK...................AGLYDAIWQAFAVLL.PVQTVG
GUAA_RHIME    GRELGLPESFIGRHPFPGPGLAIRCPG.GITREKLEILREADAIYLDEIRK...................AGLYDAIWQAFAVLL.PVQTVG
GUAA_SINMW    GRELGLPDSFIGRHPFPGPGLAIRCPG.GISREKLEILREADAIYLDEIRK...................AGLYDAIWQAFAVLL.PVQTVG
GUAA_AGRVS    GKELGLPDSFIGRHPFPGPGLAIRCPG.GVTREKLDILREADAIYLDEIRK...................AGLYDAIWQAFAVLL.PVQTVG
GUAA_BRUSI    GKELGLPDSFIGRHPFPGPGLAIRCPG.GVTREKLEILREADAIYLDEIRK...................AGLYDAIWQAFAVLL.PVQTVG
GUAA_BRUAB    GKELGLPDSFIGRHPFPGPGLAIRCPG.GVTREKLEILREADAIYLDEIRK...................AGLYDAIWQAFAVLL.PVQTVG
GUAA_BRUA2    GKELGLPDSFIGRHPFPGPGLAIRCPG.GVTREKLEILREADAIYLDEIRK...................AGLYDAIWQAFAVLL.PVQTVG
GUAA_BRUA1    GKELGLPDSFIGRHPFPGPGLAIRCPG.GVTREKLEILREADAIYLDEIRK...................AGLYDAIWQAFAVLL.PVQTVG
GUAA_BRUO2    GKELGLPDSFIGRHPFPGPGLAIRCPG.GVTREKLEILREADAIYLDEIRK...................AGLYDAIWQAFAVLL.PVQTVG
GUAA_BRUSU    GKELGLPDSFIGRHPFPGPGLAIRCPG.GVTRGKLEILREADAIYLDEIRK...................AGLYDAIWQAFAVLL.PVQTVG
GUAA_BRUC2    GKELGLPDSFIGRHPFPGPGLAIRCPG.GVTRGKLEILREADAIYLDEIRK...................AGLYDAIWQAFAVLL.PVQTVG
GUAA_BRUMB    GKELGLPDSFIGRHPFPGPGLAIRCPG.GVTREKLEILREADAIYLDEIRK...................AGLYDAIWQAFAVLL.PVQTVG
GUAA_RHILO    GKELGLPESFIGRHPFPGPGLAIRCPG.GITREKLEILREADAIYLDEIRK...................AGLYDAIWQAFAVLL.PVQTVG
GUAA_AGRT5    GRELGLPESFIGRHPFPGPGLAIRCPG.AITREKLDILRKADAIYLDEIRK...................AGLYDTIWQAFAVLL.PVQTVG
GUAA_BRADU    GRELGLPEIFVGRHPFPGPGLAIRCPG.DITSDKLDILRKADAVYIDQIRK...................HGLYDDIWQAFAVLL.PVKTVG
GUAA_BRASO    GRELGLPDVFVGRHPFPGPGLAIRCPG.EITTDKLDILRKADAIYIDEIRK...................AGLYDTIWQAFAVLL.PVKTVG
GUAA_OLICO    GRELGLPDVFVGRHPFPGPGLAIRCPG.EITPEKLDILRQADAIYIEEIRR...................AGLYDTIWQAFAVLL.PVKTVG
GUAA_RUEST    GRELGLPDSFIGRHPFPGPGLAIRCPG.EITRDKLDILREADAIYIDQIRK...................HGLYDEIWQAFVAIL.PVRTVG
GUAA_RUEPO    GRELGLPQSFIGRHPFPGPGLAIRCPG.EITREKLEILREADAIYIDQIRK...................HGLYDEIWQAFVAIL.PVRTVG
GUAA_CAUCR    GVELGLAPAFVGRHPFPGPGLAIRIPG.EITPEKVKVLQDADAIYLEEIRN...................AGLYDQIWQAFAVLL.PVKTVG
GUAA_CAUCN    GVELGLAPAFVGRHPFPGPGLAIRIPG.EITPEKVKVLQDADAIYLEEIRN...................AGLYDQIWQAFAVLL.PVKTVG
GUAA_PHEZH    GVELGLPPQFVGRHPFPGPGLAIRIPG.EVTPERVAILQKADAIYLDEIRK...................AGLYDSIWQAFAVLL.PVRTVG
GUAA_RHORT    GRELGLPDEMVGRHPFPGPGLAIRIPGQPLTREKLDILRRADAIYLEEIRN...................AGLYDVIWQAFAVLL.PVRTVG
GUAA_GLUOX    GRELGIPESIVGRHPFPGPGLAIRIPG.DVTKEKLDLLRKVDAIYLEEIRR...................AGLYDAIWQAFAVLL.PVRTVG
GUAA_RHOBA    GLELGLPEQLVWRHPFPGPGLAVRCLG.EITRDKLVVLREADAIVVEEIEN...................AGLYRETSQAFAVLL.PVQSVG
GUAA_BACFR    GRELGMPEHLITRHPFPGPGLAVRILG.DITPEKVRILQDADDIFIQGLRD...................WGLYDQVWQAGVILL.PVQSVG
GUAA_BACFN    GRELGMPEHLITRHPFPGPGLAVRILG.DITPEKVRILQDADDIFIQGLRD...................WGLYDQVWQAGVILL.PVQSVG
GUAA1_BACTN   GRELGMPEHLITRHPFPGPGLAVRILG.DITREKVRILQDADDIYIQGLRD...................WGLYDQVWQAGVILL.PVQSVG
GUAA_CYTH3    GKTLGIDDIILKRHPFPGPGLAIRILG.DITAEKVAVLQEVDYIFISRLKE...................KGLYDQVWQAGAILL.PVKSVG
GUAA_PELLD    GRELGIPEDILMRHPFPGPGLAVRVLG.SLTRERLDILREADEIYIDELKS...................SGLYQQVWQAFSVLL.PVQSVG
GUAA_PROVI    GRELGIAEDILMRHPFPGPGLAVRVLG.SVNPERVEILQNADEIFIEELKS...................SGLYQQVWQAFSVLL.PVQSVG
GUAA_CHLL2    GRELGIAEDILMRHPFPGPGLAVRVLG.SVSKERLDILRDADEIFLEELKS...................SGLYQKVWQAFSVLL.PVQSVG
GUAA_CHLPD    GRELGIAEDILMRHPFPGPGLAVRVLG.SVSRPRLDILREADEIFIEELKT...................SGLYQHVWQAFSVLL.PVQSVG
GUAA_PELPB    GRELGIAEDILMRHPFPGPGLAVRVLG.SVTKERLDILRDADEIFIEELKS...................TGLYQKVWQAFAVLL.PVQSVG
GUAA_CHLTE    GRELGIAEDILMRHPFPGPGLAVRVLG.SLTRERLDVLRDADQIFIDELKS...................SGLYSKVWQAFSVLL.PVQSVG
GUAA_CHLCH    GRELGIPEDILMRHPFPGPGLAVRVLG.SLTHERLEILREADEIYIEELQA...................SGLYSKVWQAFAVLL.PVQSVG
GUAA_CHLPB    GRELGIDEDILMRHPFPGPGLAVRVLG.SVSKTRLDILRDADEIFLEELKS...................QNLYHHVWQAFSVLL.PVQSVG
GUAA_CHLT3    GKLLDVPDEILTRHPFPGPGLAVRVLG.SVSKERLDVLREADAIFIDELKK...................HDLYLQIWQAFTVLL.PVQTVG
GUAA_SALRD    GRLLDVPDPIVGRHPFPGPGLAIRILG.QITRERLALLREADAIFIEELRA...................NDLYDEVWQAFAVLL.PVQAVG
GUAA_GEMAT    GRELGLPEEMVGRHPFPGPGLAIRVLG.EVTPHALDVLRRSDAIYLEEIRA...................AGLYPDIWQAFAVFL.PVKSVG
GUAA_KLULA    GELLGISHELVWRHPFPGPGIAIRVLG.EVTREQVAIARKADYIYIEEIRK...................AGLYNNISQAFACLL.PVKSVG
GUAA_ASHGO    GELLGISHELVWRHPFPGPGIAIRVLG.EVTKEQVEIARKADHIYIEEIRK...................AGLYNKISQAFACLL.PVKSVG
GUAA_YEAST    GELLGIPHDLVWRHPFPGPGIAIRVLG.EVTKEQVEIARKADNIYIEEIKK...................AGLYNQISQAFACLL.PVKSVG
GUAA_CANGA    GELLGISHELVWRHPFPGPGIAIRVLG.EVTREQVEIARKADHIYIEEIRK...................AGLYDKISQAFACLL.PVKSVG
GUAA_DEBHA    GEIMGVPHDLVWRHPFPGPGLAIRVLG.EVTREQLKIAREADNIFIEEIRK...................AGLYKDISQAFAALL.PVKSVG
GUAA_CANAL    GELLGVPEDLVWRHPFPGPGLAIRVLG.EVTKEQVKIAREADAIFIEEIKK...................AGLYRQISQAFAALL.PVKSVG
GUAA_YARLI    GEIMGIPHDLVWRHPFPGPGIAIRVLG.EVTPSQVEIARKADFIYIEEIKK...................AGIYEEISQAFACLL.PVKSVG
GUAA_PHANO    GVELGIPEDLVWRHPFPGPGIAIRILG.EVTREQVRIAREADYIFIEEIKA...................AGLYRNISQAFAALL.PVKAVG
GUAA_EMENI    GRQLGISPELVGRHPFPGPGIAIRVLG.EVTREKVEMARQADHIFISMIRE...................AGIYDEIGQAYAALD.PSRAVG
GUAA_ASPOR    GRQLGIAHEMVMRHPFPGPGIAIRILG.EITPERVEMARKADHIFISMIRE...................AGLYDKIGQAFAALD.PSRAVG
GUAA_ASPFU    GRELGIAHELVMRHPFPGPGIAIRVLG.EVTPERVDIARKADHIFISMIRE...................AGLYDKISQAYAALD.PSKAVG
GUAA_USTMA    GKLLGIPAHLVGRHPFPGPGLAIRILG.PVTREQVKILQHADSIYIDEIRA...................AGLYDQISQAFAVLL.PVRAVG
GUAA_DEHM1    GKELGLPDEMIWRQPFPGPGLAIRIIG.EVTREKLEILRAADWIVMSEIKK...................AKMYHQVWQSFAILT.DVKSVG
GUAA_DEHMC    GKELGLPDEMIWRQPFPGPGLAIRIIG.EVTREKLEILRSADWIVMSEIKK...................AKMYHQVWQSFAILT.DVKSVG
GUAA_DESPS    GVELGLPDEAVHRQPFPGPGLSIRIMG.EVTPERLDIVRRADVIVLDEMKK...................HGYYNKVWQSFAVLL.PIQTVG
GUAA_BRAHW    GLELKLPEDIVYRQPFPGPGLAVRILG.DITEERVKILQEADDIVVSEIKK...................AGLYRKLWQSFAILL.PVKSVG
GUAA_DESVV    AAELGLPDFIIWRHPFPGPGLAIRVIG.EITEERLDILRRADKVVQQELMS...................SGWYRKVWQGFAVLL.PLKTVG
GUAA_DESVH    AAELGLPDFIIWRHPFPGPGLAIRVIG.EITEERLDILRRADKVVQQELMS...................SGWYRKVWQGFAVLL.PLKTVG
GUAA_DESVM    AGELGLPDFIIWRHPFPGPGLAIRVIG.EITEERLDILRKADKIVQAELMS...................SGWYRKVWQGFAVLL.PLKTVG
GUAA_DESAG    AVELGLPDFIVWRHPFPGPGLAIRVIG.EVTEERLDILRKADKIVQNELTA...................SGWYRKVWQGFAVLL.PLKTVG
GUAA_DESMR    AVELGMPDFIIWRHPFPGPGLAIRIIG.EVTEERLEILRRTDKIVQSELVA...................SGWYRKVWQGFAVLL.PLKTVG
GUAA_DESAD    AYELGLPEFIIWRQPFPGPGLAIRVLG.EITEERLEILRQADKIVQNEMHA...................SGWYRKVWQGFAVLL.PLKTVG
GUAA_CALBD    GIELGIPEKIVKRQPFPGPGLAIRIIG.EVTEEKLDILREVDWIFRKEIEQ...................SGLDNEIWQYFAVLT.NMKSVG
GUAA_CALS8    GVELGIPEKIVKRQPFPGPGLAIRIIG.EVTEEKLEILREVDWIFRKEIEA...................CGLDEEIWQYFAVLT.DMRSVG
GUAA_CARHZ    GLELGLPESIVWRHPFPGPGLAVRVLG.EITEEKLAILREADYIFIDELKK...................SGWYRKTWQAFAVLP.NLQSVG
GUAA_CLOBL    GEELGIPHKLVWRQPFPGPGLAIRVLG.EVTEEKLAITREADAIFREEIAK...................AGLEEKIWQYFACLP.NIQSVG
GUAA_CLOBK    GEELGIPHKLVWRQPFPGPGLAIRVLG.EVTEEKLAITREADAIFREEIAK...................AGLEEKIWQYFACLP.NIQSVG
GUAA_CLOBM    GEELGIPHKLVWRQPFPGPGLAIRVLG.EVTEEKLAITREADAIFREEIAK...................AGLEEKIWQYFACLP.NIQSVG
GUAA_CLOBJ    GEELGIPHKLVWRQPFPGPGLAIRVLG.EVTEEKLAITREADAIFREEIAK...................AGLEEKIWQYFACLP.NIQSVG
GUAA_CLOB6    GEELGIPHKLVWRQPFPGPGLAIRVLG.EVTEEKLAITREADAIFREEIAK...................AGLEEKIWQYFACLP.NIQSVG
GUAA_CLOBH    GEELGIPHKLVWRQPFPGPGLAIRVLG.EVTEEKLAITREADAIFREEIAK...................AGLEEKIWQYFACLP.NIQSVG
GUAA_CLOB1    GEELGIPHKLVWRQPFPGPGLAIRVLG.EVTEEKLAITREADAIFREEIAK...................AGLEEKIWQYFACLP.NIQSVG
GUAA_CLONN    GEELGIPHKLVWRQPFPGPGLAIRVLG.EITEEKLEITREADAIFREEIAL...................AGLEEKIWQYFACLP.NIQSVG
GUAA_CLOAB    GEELGIPHKLVWRQPFPGPGLGIRVLG.EVTEEKLEIVREADAIFREEIAN...................AGLEEKIWQYFACLP.NIHSVG
GUAA_CLOTE    GEELGIPHKLVWRQPFPGPGLGIRVLG.EVTEEKLEIVREADAIFREEIAN...................AGLDEKIWQYFACLP.NIRSVG
GUAA_CLOPE    GEELGIPHHLVWRQPFPGPGLAIRVLG.EVTEDKLEVVREADAIFREEIAL...................AGLESEIWQYFAVLP.NIQSVG
GUAA_CLOB8    GEELGIPHKLVWRQPFPGPGLAIRVLG.EITEEKLEIVREADAIFREEIAN...................ANLDESIWQYFACLP.NIRSVG
GUAA_ALKMQ    GLELGVPEEIVWRQPFPGPGLAVRVLG.EITEEKLHIVREADAIVRDEIIK...................AGLHRQIWQYFAVLP.NIMSVG
GUAA_CLOTH    GIELGIPEDIVMRQPFPGPGLAVRVIG.EVTKEKVDILRDADYIFREEIKN...................AGLDREINQYFAVLT.GMRSVG
GUAA_CLOCE    GEELGIPEDLVWRQPFPGPGLAIRVIG.DLTKEKLDTLRDTDYIFREEIKA...................AGLDKEINQYFTVLT.NMRSVG
GUAA_CLOBA    GLELGIPEKLVFRQPFPGPGLGIRIIG.EVTAEKVKIVQDADAIYREEIAN...................AGIDKEIGQYFAALT.NMRSVG
GUAA_PEPD6    GLELGIEEGLIFRHPFPGPGLGIRVIG.DVTKEKCDILREADAVYMDELRK...................AGLYREIWQAFATLP.DVKTVG
GUAA_FINM2    GLELEMPEYLVYRQPFPGPGLGIRVMG.EITEEKLEVLREADFIFRDEVAK...................AGIDKDINQYFAVIT.NNRTVG
GUAA_TREDE    GTELGLPDYLVHRQPFPGPGLAIRIMG.EITEEKLDILREADAIWRSELEH...................ADIKKDLSQYFAVLT.STKTVG
GUAA_BACCR    GSELGIPDEIVWRQPFPGPGLGIRVLG.EITEEKLEIVRESDAILREEIIK...................AGLDREIWQYFTALP.GMRSVG
GUAA_BACC4    GSELGIPDEIVWRQPFPGPGLGIRVLG.EITEEKLEIVRESDAILREEIIK...................AGLDREIWQYFTALP.GMRSVG
GUAA_BACC2    GSELGIPDEIVWRQPFPGPGLGIRVLG.EITEEKLEIVRESDAILREEIIK...................AGLDREIWQYFTALP.GMRSVG
```

```
GUAA_BACWK              GSELGIPDEIVWRQPFPGPGLGIRVLG.EITEEKLEIVRESDAILREEIIK...................AGLDREVWQYFTALP.GMRSVG
GUAA_BACCZ              GSELGIPDEIVWRQPFPGPGLGIRVLG.EITEEKLEIVRESDAILREEIIK...................AGLDREIWQYFTALP.GMRSVG
GUAA_BACC3              GSELGIPDEIVWRQPFPGPGLGIRVLG.EITEEKLEIVRESDAILREEIIK...................AGLDREIWQYFTALP.GMRSVG
GUAA_BACAH              GSELGIPDEIVWRQPFPGPGLGIRVLG.EITEEKLEIVRESDAILREEIIK...................AGLDREIWQYFTALP.GMRSVG
GUAA_BACAC              GSELGIPDEIVWRQPFPGPGLGIRVLG.EITEEKLEIVRESDAILREEIIK...................AGLDREIWQYFTALP.GMRSVG
GUAA_BACAA              GSELGIPDEIVWRQPFPGPGLGIRVLG.EITEEKLEIVRESDAILREEIIK...................AGLDREIWQYFTALP.GMRSVG
GUAA_BACHK              GSELGIPDEIVWRQPFPGPGLGIRVLG.EITEEKLEIVRESDAILREEITK...................AGLDREIWQYFTALP.GMRSVG
GUAA_BACC0              GSELGIPDEIVWRQPFPGPGLGIRVLG.EITEEKLEIVRESDAILREEIIK...................AGLDREIWQYFTALP.GMRSVG
GUAA_BACAN              GSELGIPDEIVWRQPFPGPGLGIRVLG.EITEEKLEIVRESDAILREEIIK...................AGLDREIWQYFTALP.GMRSVG
GUAA_BACC1              GSELGIPDEIVWRQPFPGPGLGIRVLG.EITEEKLEIVRESDAILREEIIK...................AGLDREIWQYFTALP.GMRSVG
GUAA_BACCN              GSELGIPDEIVWRQPFPGPGLGIRVLG.EITEEKLEIVRESDAILREEIQK...................AGLDREIWQYFTALP.GMRSVG
GUAA_BACA2              GTELGLPDDIVWRQPFPGPGLGIRVLG.EVTEEKLEIVRESDAILREEVAN...................HGLERDIWQYFTVLP.DIRSVG
GUAA_BACSU              GTELGIPDEIVWRQPFPGPGLGIRVLG.EVTEEKLEIVRESDAILREEIAN...................HGLERDIWQYFTVLP.DIRSVG
GUAA_BACLD              GTELGIPDEIVWRQPFPGPGLGIRVLG.EVTEEKLEIVRESDAILREEIAK...................ADLEKDIWQYFTVLP.DIRSVG
GUAA_BACP2              GSELGIPDDIVWRQPFPGPGLGIRVLG.EISEEKLEIVRESDAILREEIAN...................FGLERDIWQYFTVLP.DIRSVG
GUAA_BACSK              GTELGIPDEIVWRQPFPGPGLGIRVLG.EITDEKLKIVRESDAILREEIKK...................AGLDREIWQYFTALP.NMRSVG
GUAA_BACHD              GTELGIPDEVVWRQPFPGPGLGIRVLG.EITEEKLEIVRESDAILREEIKK...................AGLDREIWQYFTALP.NMRSVG
GUAA_GEOKA              GTQLGIPDEIVWRQPFPGPGLGIRVLG.EVTEEKLEIVRESDAILREEVKK...................AGLDREIWQYFTVLP.DIRSVG
GUAA_LISMF              GTELGMPDAIVWRQPFPGPGLGIRVLG.EITEEKLEIVRDSDYILREEIKN...................AGLEREIWQYFTALP.NIRSVG
GUAA_LISMO              GTELGMPDAIVWRQPFPGPGLGIRVLG.EITEEKLEIVRDSDYILREEIKN...................AGLEREIWQYFTALP.NIRSVG
GUAA_LISW6              GTELGMPDAIVWRQPFPGPGLGIRVLG.EITEEKLEIVRDSDYILREEIKK...................AGLEREIWQYFTALP.NIRSVG
GUAA_LISIN              GTELGMPDAIVWRQPFPGPGLGIRVLG.EITEEKLEIVRDSDYILREEIKN...................AGLEREIWQYFTALP.NIRSVG
GUAA_EXIS2              GKELGLSDEIVWRQPFPGPGLGIRVLG.EITDEKLEIVRESDFILRDEIKK...................AGLEREIWQYFTVLP.PLRSVG
GUAA_STAES              GIELGIPEHLVWRQPFPGPGLGIRVLG.EITEDKLEIVRESDAILREVIRE...................EGLERDIWQYFTVLP.GIQSVG
GUAA_STAEQ              GIELGIPEHLVWRQPFPGPGLGIRVLG.EITEDKLEIVRESDAILREVIRE...................EGLERDIWQYFTVLP.GIQSVG
GUAA_MACCJ              GIELGIPEHLVWRQPFPGPGLGIRVLG.EITEEKLEIVRESDAILREVIAE...................EGLERDIWQYFTVLP.DIRSVG
GUAA_OCEIH              GSQLGVPDRIVWRQPFPGPGLAIRILG.EVTEEHLEIVRESDAILREEIAK...................AGLDRDIWQYFTVLP.NIKSVG
GUAA_LACRJ              GEKLGIPHELVWRQPFPGPGLGIRVIG.EITPEKLEIVRESDAILREEIKK...................AGLDEEIWQYFTVLP.GIRSVG
GUAA_LACRD              GEKLGIPHELVWRQPFPGPGLGIRVIG.EITPEKLEIVRESDAILREEIKK...................AGLDEEIWQYFTVLP.GIRSVG
GUAA_LACJO              GEKLGIPHDLVWRQPFPGPGLGIRVIG.EVTEDKLKIVRESDAILREEIKN...................AGLQEDIWQYFTVLP.GIRSVG
GUAA_LACGA              GEKLGIPHDLVWRQPFPGPGLAIRVLG.EVTEDKLKIVRESDAILRDEIKK...................AGLQEDIWQYFTVLP.GIRSVG
GUAA_LACAC              GEKLGIPHDLVWRQPFPGPGLGIRVLG.EVTEDKLKIVRESDAILREEIKK...................AGLQEKIWQYFTVLP.GIRSVG
GUAA_LACDB              GEKLGLPHDLVWRQPFPGPGLAIRVIG.EITEEKLRIVRDADAILREEVKN...................AGLQESIWQYFVALP.GMRSVG
GUAA_LACDA              GEKLGLPHDLVWRQPFPGPGLAIRVIG.EITEEKLRIVRDADAILREEVKN...................AGLQESIWQYFVALP.GMRSVG
GUAA_LACCB              GEKLGMPHALVWRQPFPGPGLGIRVIG.EVTEDKLEIVRDSDYILREEIAK...................HGLDKDIWQYFTVLP.GFRSVG
GUAA_LACC3              GEKLGMPHALVWRQPFPGPGLGIRVIG.EVTEDKLEIVRDSDYILREEIAK...................HGLDKDIWQYFTVLP.GFRSVG
GUAA_LACRH              GEKLGMPHSLVWRQPFPGPGLGIRVIG.EVTEDKLEIVRDSDYILREEIAK...................HGLDKDIWQYFTVLP.GIRSVG
GUAA_LACS1              GEKLGMPHELVWRQPFPGPGLGIRVIG.EITEEKLEIVRDSDLILREEIAK...................HGLDKEIWQYFTVLP.GIRSVG
GUAA_LACLM              GTQLGMPDEIVWRQPFPGPGLAIRVLG.DLTEEKLETVRESDAILREEIAA...................SGLERDVWQYFTVNT.DVKSVG
GUAA_LACLS              GTQLGMPDEIVWRQPFPGPGLAIRVLG.DLTEEKLETVRESDAILREEIAA...................SGLERDVWQYFTVNT.DVKSVG
GUAA_LACLA              GTQLGMPDEIVWRQPFPGPGLAIRVLG.DLTEEKLETVRESDAILREEIAA...................AGLERDVWQYFTVNT.DVKSVG
GUAA_STRZJ              GTELGMPDHIVWRQPFPGPGLAIRVMG.EITEEKLETVRESDAILREEIAK...................AGLDRDIWQYFTVNT.GVRSVG
GUAA_STRPJ              GTELGMPDHIVWRQPFPGPGLAIRVMG.EITEEKLETVRESDAILREEIAK...................AGLDRDIWQYFTVNT.GVRSVG
GUAA_STRZT              GTELGMPDHIVWRQPFPGPGLAIRVMG.EITEEKLETVRESDAILREEIAK...................AGLDRDIWQYFTVNT.GVRSVG
GUAA_STRR6              GTELGMPDHIVWRQPFPGPGLAIRVMG.EITEEKLETVRESDAILREEIAK...................AGLDRDIWQYFTVNT.GVRSVG
GUAA_STRPN              GTELGMPDHIVWRQPFPGPGLAIRVMG.EITEEKLETVRESDAILREEIAK...................AGLDRDIWQYFTVNT.GVRSVG
GUAA_STRPI              GTELGMPDHIVWRQPFPGPGLAIRVMG.EITEEKLETVRESDAILREEIAK...................AGLDRDIWQYFTVNT.GVRSVG
GUAA_STRP7              GTELGMPDHIVWRQPFPGPGLAIRVMG.EITEEKLETVRESDAILREEIAK...................AGLDRDIWQYFTVNT.GVRSVG
GUAA_STRP4              GTELGMPDHIVWRQPFPGPGLAIRVMG.EITEEKLETVRESDAILREEIAK...................AGLDRDIWQYFTVNT.GVRSVG
GUAA_STRP2              GTELGMPDHIVWRQPFPGPGLAIRVMG.EITEEKLETVRESDAILREEIAK...................AGLDRDIWQYFTVNT.GVRSVG
GUAA_STRZP              GTELGMPDHIVWRQPFPGPGLAIRVMG.EITEEKLETVRESDAILREEIAK...................AGLDRDIWQYFTVNT.GVRSVG
GUAA_STRPS              GTELGMPDHIVWRQPFPGPGLAIRVMG.EITEEKLETVRESDAILREEIAK...................AGLDRDIWQYFTVNT.GVRSVG
GUAA_SYMTH              GRALGVPEEIVGRHPFPGPGLGVRVLG.EVTPEKLEILREADRIFVEEIRK...................AGLYDELWQAFTVLP.DVRSVG
GUAA_MOOTA              GEELGLPEEIVWRQPFPGPGLAIRILG.EVTPEKLEILRQADAIVTEEIRR...................AGLYREIWQSFAVLP.SMKSVG
GUAA_HELMI              GQRLGLPEDIVWRQPFPGPGLAIRVLG.EITKESLDILRHADDIVFQEIKK...................AGLYRQIWQSFVVLPTTVRSVG
GUAA_HALOH              GEVLGLPDEIVWRQPFPGPGLAIRIIG.EIDGKKLEILRKADSIIQEEIKE...................AGYYKEIWQSFAVLP.DLKSVG
GUAA_THETN              GKELGIPDEILYRQPFPGPGLAVRIVG.EVTEEKLEILRLADSIVQREMKR...................FGWYNKVWQSFAILP.GIKSVG
GUAA_NATTJ              ARELGLHREIVERHPFPGPGLAIRVLG.EITKEKLDILREADAVVSDELKK...................IGLYHDIWQLFTVLP.DIKTVG
GUAA_MYCTU              GRELGLPEEIVARQPFPGPGLGIRIVG.EVTAKRLDTLRHADSIVREELTA...................AGLDNQIWQCPVVLLADVRSVG
GUAA_MYCTA              GRELGLPEEIVARQPFPGPGLGIRIVG.EVTAKRLDTLRHADSIVREELTA...................AGLDNQIWQCPVVLLADVRSVG
GUAA_MYCBT              GRELGLPEEIVARQPFPGPGLGIRIVG.EVTAKRLDTLRHADSIVREELTA...................AGLDNQIWQCPVVLLADVRSVG
GUAA_MYCBP              GRELGLPEEIVARQPFPGPGLGIRIVG.EVTAKRLDTLRHADSIVREELTA...................AGLDNQIWQCPVVLLADVRSVG
GUAA_MYCBO              GRELGLPEEIVARQPFPGPGLGIRIVG.EVTAKRLDTLRHADSIVREELTA...................AGLDNQIWQCPVVLLADVRSVG
GUAA_MYCTO              GRELGLPEEIVARQPFPGPGLGIRIVG.EVTAKRVDTLRHADSIVREELTA...................AGLDNQIWQCPVVLLADVRSVG
GUAA_MYCMM              GRELGLPEEIVARQPFPGPGLGIRIVG.EVTGQRLDTLRRADSIAREELTA...................AGLDNQIWQCPVVLLADVRSVG
GUAA_MYCPA              GRELGLPEEIVARQPFPGPGLGIRIVG.EVTATRLDTLRRADSIAREELTA...................AGLDSLIWQCPVVLLGEVRSVG
GUAA_NOSS1              GRSVGLPEEIVQRQPFPGPGLAIRILG.EVTADRLNILRDADLIVRQEINQ...................RGLYNEYWQAFAVLL.PIRSVG
GUAA_ANAVT              GRSVGLPEEIVQRQPFPGPGLAIRILG.EVTADRLNILRDADLIVRQEINQ...................RGLYNEYWQAFAVLL.PIRSVG
GUAA_NOSP7              GRSIGLPEEIVQRQPFPGPGLAIRILG.EVTSERLNILRDADLIVRQEINQ...................RGMYHDFWQAFAVLL.PIRSVG
GUAA_THEEB              GRSLGLPEEIVQRHPFPGPGLAIRILG.EVTPERLEILRDADLIVRQEINR...................AQMYHELWQAFAVLL.PIRTVG
GUAA_CYAA5              GRSIGLPEEIVRRHPFPGPGLAIRIIG.EVTSERLNILRDADFVVRDEIGK...................QGMYHDFWQAFAVLL.PIRSVG
GUAA_CYAP8              GRAIGLPEEIVRRHPFPGPGLAIRIIG.EVTAERLNILRDADFIVRDEISK...................QGMYHDFWQAFAVLL.PVRSVG
GUAA_CYAP7              ARSIGLPEEIVRRHPFPGPGLAIRIIG.EVTSERLKILRDADFVVRDEISK...................QGMYHDFWQAFAVLL.PVRSVG
GUAA_SYNY3              GRSIGLPEEIVRRHPFPGPGLAIRIIG.EVTSERLNILRDADFIVRDEISK...................RGIYHDYWQAFAVLL.PIRSVG
GUAA_SYNP2              AASIGLPEEIIRRHPFPGPGLAIRIIG.EVTAERLNILRDADWVVRDEIKK...................QGVYSDFWQAFAVLL.PIRSVG
GUAA_SYNP6              GRSLGLPEEIVKRHPFPGPGLAIRILG.EVTAEKLNILRDADLIVRQEVNR...................QGYYDEFWQAFAVLL.PVKSVG
GUAA_GLOVI              GRALGLPEEIVQRQPFPGPGLAIRVLG.ELTKDKVDILREADFILRQEVNR...................SGRYNDYWQSFAVLL.PVKTVG
GUAA_PROMP              GGALGLPDEIIKRHPFPGPGLAIRILG.DVSHEKLNCLRDADWIVRDEIKK...................AGLYNDIWQAFAVLL.PVKTVG
GUAA_PROM5              GNALGLPDEIIKRHPFPGPGLAIRILG.EVSIEKLNCLRDADWIVRDEIKK...................AGLYNDIWQAFAVLL.PVKTVG
GUAA_PROMT              GKSLGLPDEIVRRHPFPGPGLAIRILG.EVTHEKLNCLRDADLIVREEINN...................AGLYNKIWQAFAVLL.PVYSVG
GUAA_PROM1              GKSLGLPDEIVRRHPFPGPGLAIRILG.EVTHEKLNCLRDADLIVREEINN...................AGLYNKIWQAFAVLL.PVYSVG
GUAA_THERP              GRLLGLPEEIVQRQPFPGPGLAVRILG.EVTEEALAIVRRADTIVREEVEA...................AGLSDGLWQFFAVLL.PVHSTG
GUAA_DEIGD              ARLLGLPDAIRMRHPFPGPGLAIRCLG.EVTREKLDILKRVDDIFISGLRE...................FGLYDGCSQALAVLT.PIQSVG
GUAA_DEIDV              AQLLGLPDHIRMRHPFPGPGLAIRCLG.EVTEEKLEILKRVDDIFISGLRE...................FGLYDGCSQALAVLT.PIQSVG
GUAA_DEIRA              ARLLGLPDHIRMRHPFPGPGLAIRCLG.EVTAEKVDILQRVDDIFISGLRE...................FGLYDGCSQALAVLT.PIQSVG
GUAA_FUSNN              GRELGIPDYMVDRHPFPGPGLGIRILG.EVTKEKADILREADAIFIEELRK...................ADLYNKVSQAFVVLL.PVKSVG
GUAA_MESFL              GFKLGLPEEMINRHPFPGPGLGIRVIE.EVTKDKVEILQEADAIFIKKLRE...................WNLYNTVSQAFVTLL.PVKTVG
GUAA_SPIKU              GEALGIDFKFVYKHPFPGPGLAVRIIG.EITAEKIALLQAADQIFIDELYQ...................ANLYDQVAQAFVVLL.PVQSVG
GUAA_BORGA              GINLGIKKEALYRHPFPGPGLAIRIIG.EVTQEKINILQEAENILTEELFT...................NDLYYEIRQAFVVLL.PVKSVG
GUAA_SULDN              GLELGLPESMINRHPFPGPGLAIRIMG.DVNEADLTLLREADVILLDELKA...................SGYYAKTWQAFVVLL.NVKSVG
GUAA_WOLSU              GAELGMPREMLMRHPFPGPGLAIRIMG.EVNFEDLELLRKADVIMLDELKK...................SGFYDKTWQAFTVLL.NVRSVG
GUAA_SULNB              GLELGLPKHMIGRHPFPGPGLAIRVMG.EVNEEALRLLRESDAIMQEELRS...................TGYYDKVWQAFTVLL.NVQSVG
GUAA_ARCB4              GLELGLPKSMISRHPFPGPGLAIRIMG.EVNKPDLELLRKADVIMLDVLHS...................TGYYDKTWQAFTVLL.NVKSVG
GUAA_NITSB              GLELGLPREMVYRHPFPGPGLAIRILG.EVNEEALDLVRKADTILLEEIKA...................HGLYEKLWQSFAVLL.NVKSVG
GUAA_CAMHC              GTELGLSPDLVYRHPFPGPGLAIRILG.EITPEKLCVLRKADVILRDELKS...................SGWYNKTWQAFCVLL.NVNSVG
GUAA_THESQ              GKYLGIPDRIINRHPFPGPGLAVRVLG.EVTEEKLEILREADYIFIETLRK...................HDYYDKVWQAFAVLL.PIKSVG
GUAA_THEMA              GKYLGIPDRIINRHPFPGPGLAVRVLG.EVTEEKLEILREADYIFIETLRK...................HDYYDKVWQAFAVLL.PIKSVG
GUAA_THEP1              GKYLGIPDRIINRHPFPGPGLAVRVLG.EVTEEKLEILREADYIFIETLRK...................HDYYDKVWQAFAVLL.PIKSVG
GUAA_PETMO              GEILGLPREILYRHPFPGPGLAIRIMG.EINDEKLTILKKVDNIFINTLKE...................TGWYDKVWQAFAVLI.PVKTVG
GUAA_AQUAE              GKLLGVPEEILRRHPFPGPGLAIRIIG.EVNKKDLEILRKADYIFIQELKK...................EGLYDRVWQAFAVLL.PVKSVG
GUAA_THEAB              GKILGLPDEMINRHPFPGPGLAVRIIG.EVTNEAIKILQHADHIFIEELKK...................NDLYDKVWQAFAVFL.PIRSVG
GUAA_THEM4              GEILELPQSMINRHPFPGPGLAVRIIG.KVTEEAISILQKADYIFIEELKK...................NNLYDKVWQAFAVFL.PIKSVG
GUAA_PELUB              GLELNLSNDIISRHPFPGPGLAIRMPG.IITNEKIKILKEADFYFIKALRE...................HGLYDKIWQAYAALL.PVKTVG
GUAA_MYCPE              GLSLDIPYQNVYRQPFPGPGLAVRVLG.EITEDKLEIVREADFLFRSYIDQLY.................KNSEHKPWQYFTVLT.NSKSVG
tr|Q7RIY7|Q7RIY7_PLAYO  SQELNLPKELTNRHPFPGPGLAIRVIG.EIDKHKLDILREVDDIFINSLKE...................YNLYDDIGQAFAVIF.SSKSVG
tr|Q4Z1X2|Q4Z1X2_PLABA  SQELNLPKELTNRHPFPGPGLAIRVIG.EIDKHKLDILREVDDIFINSLKE...................YNLYDDIGQAFAVIF.SSKSVG
tr|Q4Y2M7|Q4Y2M7_PLACH  SQELNLPKELTNRHPFPGPGLAIRVIG.EIDKHKLDILREVDDIFINSLKE...................FNLYDDIGQAFAVIF.SSKSVG
tr|B3L3R6|B3L3R6_PLAKH  SKELNLPDEITNRHPFPGPGLAIRVIG.EIDKHKLSILREVDDIFIKDLKA...................YNLYNDISQAFAVLL.PTKSVG
tr|A5K7F1|A5K7F1_PLAVS  SQELNLPEEITNRHPFPGPGLAIRVIG.EIDKHKLSILREVDDIFINDLKA...................YNLYNDISQAFAVLL.PTKSVG
tr|Q8IJR9|Q8IJR9_PLAF7  SRELNLPEEITNRHPFPGPGLAIRVIG.EINKHKLNILREVDDIFINDLKQ...................YGLYNQISQAFAVLL.SSKSVG
```

```
                 460        470                             480        490        500        510        520
GUAA_ECO55       VMGDGRKYDWVVSLRAVETID..................FMTAHWAHLPYDFLGRVSNRIINEVNGISRVVYDISGKPPATIEWE
GUAA_ECO45       VMGDGRKYDWVVSLRAVETID..................FMTAHWAHLPYDFLGRVSNRIINEVNGISRVVYDISGKPPATIEWE
GUAA_ECO57       VMGDGRKYDWVVSLRAVETID..................FMTAHWAHLPYDFLGRVSNRIINEVNGISRVVYDISGKPPATIEWE
GUAA_ECO7I       VMGDGRKYDWVVSLRAVETID..................FMTAHWAHLPYDFLGRVSNRIINEVNGISRVVYDISGKPPATIEWE
GUAA_ECO5E       VMGDGRKYDWVVSLRAVETID..................FMTAHWAHLPYDFLGRVSNRIINEVNGISRVVYDISGKPPATIEWE
GUAA_ECO81       VMGDGRKYDWVVSLRAVETID..................FMTAHWAHLPYDFLGRVSNRIINEVNGISRVVYDISGKPPATIEWE
GUAA_ECO8A       VMGDGRKYDWVVSLRAVETID..................FMTAHWAHLPYDFLGRVSNRIINEVNGISRVVYDISGKPPATIEWE
GUAA_ECOHS       VMGDGRKYDWVVSLRAVETID..................FMTAHWAHLPYDFLGRVSNRIINEVNGISRVVYDISGKPPATIEWE
GUAA_ECOK1       VMGDGRKYDWVVSLRAVETID..................FMTAHWAHLPYDFLGRVSNRIINEVNGISRVVYDISGKPPATIEWE
GUAA_ECOL5       VMGDGRKYDWVVSLRAVETID..................FMTAHWAHLPYDFLGRVSNRIINEVNGISRVVYDISGKPPATIEWE
GUAA_ECOL6       VMGDGRKYDWVVSLRAVETID..................FMTAHWAHLPYDFLGRVSNRIINEVNGISRVVYDISGKPPATIEWE
GUAA_ECOLC       VMGDGRKYDWVVSLRAVETID..................FMTAHWAHLPYDFLGRVSNRIINEVNGISRVVYDISGKPPATIEWE
GUAA_ECOSE       VMGDGRKYDWVVSLRAVETID..................FMTAHWAHLPYDFLGRVSNRIINEVNGISRVVYDISGKPPATIEWE
GUAA_ECOSM       VMGDGRKYDWVVSLRAVETID..................FMTAHWAHLPYDFLGRVSNRIINEVNGISRVVYDISGKPPATIEWE
GUAA_ECOUT       VMGDGRKYDWVVSLRAVETID..................FMTAHWAHLPYDFLGRVSNRIINEVNGISRVVYDISGKPPATIEWE
GUAA_ECO24       VMGDGRKYDWVVSLRAVETID..................FMTAHWAHLPYDFLGRVSNRIINEVNGISRVVYDISGKPPATIEWE
GUAA_SHISS       VMGDGRKYDWVVSLRAVETID..................FMTAHWARLPYDFLGRVSNRIINEVNGISRVVYDISGKPPATIEWE
GUAA_SHIF8       VMGDGRKYDWVVSLRAVETID..................FMTAHWAHLPYDFLGRVSNRIINEVNGISRVVYDISGKPPATIEWE
GUAA_SHIFL       VMGDGRKYDWVVSLRAVETID..................FMTAHWAHLPYDFLGRVSNRIINEVNGISRVVYDISGKPPATIEWE
GUAA_SHIDS       VMGDGRKYDWVVSLRAVETID..................FMTAHWAHLPYDFLGRVSNRIINEVNGISRVVYDISGKPPATIEWE
GUAA_ECOLI       VMGDGRKYDWVVSLRAVETID..................FMTAHWAHLPYDFLGRVSNRIINEVNGISRVVYDISGKPPATIEWE
GUAA_ECODH       VMGDGRKYDWVVSLRAVETID..................FMTAHWAHLPYDFLGRVSNRIINEVNGISRVVYDISGKPPATIEWE
GUAA_ECOBW       VMGDGRKYDWVVSLRAVETID..................FMTAHWAHLPYDFLGRVSNRIINEVNGISRVVYDISGKPPATIEWE
GUAA_ESCF3       VMGDGRKYDWVVSLRAVETID..................FMTAHWAHLPYDFLGRVSNRIINEVNGISRVVYDISGKPPATIEWE
GUAA_ECO27       VMGDGRKYDWVVSLRAVETID..................FMTAHWAHLPYDFLGRVSNRIINEVNGISRVVYDISGKPPATIEWE
GUAA_SHIB3       VMGDGRKYDWVVSLRAVETID..................FMTAHWAHLPYDFLGRVSNRIINEVNGISRVVYDISGKPPATIEWE
GUAA_SHIBS       VMGDGRKYDWVVSLRAVETID..................FMTAHWAHLPYDFLGRVSNRIINEVNGISRVVYDISGKPPATIEWE
GUAA_ECOLU       VMGDGRKYDWVVSLRAVETID..................FMTAHWAHLPYDFLGRVSNRIINEVNGISRVVYDISGKPPATIEWE
GUAA_SALPK       VMGDGRKYDWVVSLRAVETID..................FMTAHWAHLPYDFLGRVSNRIINEVNGISRVVYDISGKPPATIEWE
GUAA_SALPC       VMGDGRKYDWVVSLRAVETID..................FMTAHWAHLPYDFLGRVSNRIINEVNGISRVVYDISGKPPATIEWE
GUAA_SALPB       VMGDGRKYDWVVSLRAVETID..................FMTAHWAHLPYDFLGRVSNRIINEVNGISRVVYDISGKPPATIEWE
GUAA_SALPA       VMGDGRKYDWVVSLRAVETID..................FMTAHWAHLPYDFLGRVSNRIINEVNGISRVVYDISGKPPATIEWE
GUAA_SALCH       VMGDGRKYDWVVSLRAVETID..................FMTAHWAHLPYDFLGRVSNRIINEVNGISRVVYDISGKPPATIEWE
GUAA_SALTY       VMGDGRKYDWVVSLRAVETID..................FMTAHWAHLPYDFLGRVSNRIINEVNGISRVVYDISGKPPATIEWE
GUAA_SALSV       VMGDGRKYDWVVSLRAVETID..................FMTAHWAHLPYDFLGRVSNRIINEVNGISRVVYDISGKPPATIEWE
GUAA_SALNS       VMGDGRKYDWVVSLRAVETID..................FMTAHWAHLPYDFLGRVSNRIINEVNGISRVVYDISGKPPATIEWE
GUAA_SALTI       VMGDGRKYDWVVSLRAVETID..................FMTAHWAHLPYDFLGRVSNRIINEVNGISRVVYDISGKPPATIEWE
GUAA_SALHS       VMGDGRKYDWVVSLRAVETID..................FMTAHWAHLPYDFLGRVSNRIINEVNGISRVVYDISGKPPATIEWE
GUAA_SALG2       VMGDGRKYDWVVSLRAVETID..................FMTAHWAHLPYDFLGRVSNRIINEVNGISRVVYDISGKPPATIEWE
GUAA_SALEP       VMGDGRKYDWVVSLRAVETID..................FMTAHWAHLPYDFLGRVSNRIINEVNGISRVVYDISGKPPATIEWE
GUAA_SALDC       VMGDGRKYDWVVSLRAVETID..................FMTAHWAHLPYDFLGRVSNRIINEVNGISRVVYDISGKPPATIEWE
GUAA_SALA4       VMGDGRKYDWVVSLRAVETID..................FMTAHWAHLPYDFLGRVSNRIINEVNGISRVVYDISGKPPATIEWE
GUAA_SALAR       VMGDGRKYDWVVSLRAVETID..................FMTAHWAHLPYGFLGRVSNRIINEVNGISRVVYDISGKPPATIEWE
GUAA_KLEP7       VMGDGRKYDWVVSLRAVETID..................FMTAHWAHLPYDFLGRVSNRIINEVNGISRVVYDISGKPPATIEWE
GUAA_KLEP3       VMGDGRKYDWVVSLRAVETID..................FMTAHWAHLPYDFLGRVSNRIINEVNGISRVVYDISGKPPATIEWE
GUAA_ENT38       VMGDGRKYDWVVSLRAVETID..................FMTAHWAHLPYDFLGRVSNRIINEVNGISRVVYDISGKPPATIEWE
GUAA_CITK8       VMGDGRKYDWVVSLRAVETID..................FMTAHWAHLPYDFLGRVSNRIINEVNGISRVVYDISGKPPATIEWE
GUAA_CROS8       VMGDGRKYDWVVSLRAVETID..................FMTAHWAHLPYDFLGRVSNRIINEINGISRVVYDISGKPPATIEWE
GUAA_SODGM       VMGDGRKYDWVVSLRAVETID..................FMTAHWAHLPYDFLGRVSNRIINEIDGISRVVYDISGKPPATIEWE
GUAA_PECCP       VMGDGRKYDWVVSLRAVETID..................FMTAHWAHLPYDFLGRVSNRIINEVDGISRVVYDVSGKPPATIEWE
GUAA_PECAS       VMGDGRKYDWVVALRAVETID..................FMTAHWAHLPYDFLGRVSNRIINEVDGISRVVYDVSGKPPATIEWE
GUAA_YERPY       VMGDGRKYDWVVSLRAVETVD..................FMTAHWAHLPYDFLGRVSNRIINEVNGISRVVYDISGKPPATIEWE
GUAA_YERPS       VMGDGRKYDWVVSLRAVETVD..................FMTAHWAHLPYDFLGRVSNRIINEVNGISRVVYDISGKPPATIEWE
GUAA_YERPB       VMGDGRKYDWVVSLRAVETVD..................FMTAHWAHLPYDFLGRVSNRIINEVNGISRVVYDISGKPPATIEWE
GUAA_YERP3       VMGDGRKYDWVVSLRAVETVD..................FMTAHWAHLPYDFLGRVSNRIINEVNGISRVVYDISGKPPATIEWE
GUAA_YERPP       VMGDGRKYDWVVSLRAVETVD..................FMTAHWAHLPYDFLGRVSNRIINEVNGISRVVYDISGKPPATIEWE
GUAA_YERPN       VMGDGRKYDWVVSLRAVETVD..................FMTAHWAHLPYDFLGRVSNRIINEVNGISRVVYDISGKPPATIEWE
GUAA_YERPG       VMGDGRKYDWVVSLRAVETVD..................FMTAHWAHLPYDFLGRVSNRIINEVNGISRVVYDISGKPPATIEWE
GUAA_YERPE       VMGDGRKYDWVVSLRAVETVD..................FMTAHWAHLPYDFLGRVSNRIINEVNGISRVVYDISGKPPATIEWE
GUAA_YERPA       VMGDGRKYDWVVSLRAVETVD..................FMTAHWAHLPYDFLGRVSNRIINEVNGISRVVYDISGKPPATIEWE
GUAA_YERE8       VMGDGRKYDWVVSLRAVETID..................FMTAHWAHLPYDFLGRVSNRIINEVNGISRVVYDISGKPPATIEWE
GUAA_SERP5       VMGDGRKYDWVVSLRAVETID..................FMTAHWAHLPYDFLGRVSNRIINEVNGISRVVYDISGKPPATIEWE
GUAA_PHOLL       VMGDGRKYDWVVSLRAVETID..................FMTAHWAHLPYDFLGRVSNRIINEVNGISRVVYDVSGKPPATIEWE
GUAA_PROMH       VMGDGRKYDWVVSLRAVETVD..................FMTAHWAHLPYDFLGRVSNRIINEVNGISRVVYDISGKPPATIEWE
GUAA_EDWI9       VMGDGRKYDWVVSLRAVETID..................FMTAHWAHLPYDFLGRVSNRIINEVNGISRVVYDISGKPPATIEWE
GUAA_AERHH       VMGDGRKYDWVIALRAVETID..................FMTAHWAHLPYDFLGHVSNRIINEINGISRVVYDVSGKPPATIEWE
GUAA_AERS4       VMGDGRKYDWVIALRAVETID..................FMTAHWAHLPYDFLGHVSNRIINEINGISRVVYDVSGKPPATIEWE
GUAA_TOLAT       VMGDGRKYDWVVSLRAVETID..................FMTAHWAHLPYDFLGHVSNRIINEINGISRVVYDISGKPPATIEWE
GUAA_VIBF1       VMGDGRKYDWVVSLRAVETID..................FMTAHWAHLPYDFLGKVSNRIINEVNGISRVVYDISGKPPATIEWE
GUAA_VIBFM       VMGDGRKYDWVVSLRAVETID..................FMTAHWAHLPYDFLGKVSNRIINEVNGISRVVYDISGKPPATIEWE
GUAA_ALISL       VMGDGRKYDWVVSLRAVETID..................FMTAHWAHLPYDFLGKVSNRIINEVGGISRVVYDISGKPPATIEWE
GUAA_VIBSL       VMGDGRKYDWVVSLRAVETID..................FMTAHWAHLPYDFLGKVSNRIINEVNGISRVVYDISGKPPATIEWE
GUAA_VIBCM       VMGDGRKYDWVVSLRAVETID..................FMTAHWAHLPYEFLGKVSNRIINEVNGISRVVYDISGKPPATIEWE
GUAA_VIBCH       VMGDGRKYDWVVSLRAVETID..................FMTAHWAHLPYEFLGKVSNRIINEVNGISRVVYDISGKPPATIEWE
GUAA_VIBC3       VMGDGRKYDWVVSLRAVETID..................FMTAHWAHLPYEFLGKVSNRIINEVNGISRVVYDISGKPPATIEWE
GUAA_VIBVU       VMGDGRKYDWVVSLRAVETID..................FMTAHWAHLPYDFLGKVSNRIINEVNGISRVVYDISGKPPATIEWE
GUAA_VIBVY       VMGDGRKYDWVVSLRAVETID..................FMTAHWAHLPYDFLGKVSNRIINEVNGISRVVYDISGKPPATIEWE
GUAA_VIBPA       VMGDGRKYDWVVSLRAVETID..................FMTAHWAHLPYDFLGKVSNRIINEVEGISRVVYDISGKPPATIEWE
GUAA_VIBCB       VMGDGRKYDWVVSLRAVETID..................FMTAHWAHLPYDFLGKVSNRIINEVDGISRVVYDISGKPPATIEWE
GUAA_PHOPR       VMGDGRKYDWVVSLRAVETID..................FMTAHWAHLPYDFLGKVSNRIINEVDGISRVVYDISGKPPATIEWE
GUAA_COLP3       VMGDARKYDWVVSLRCVETID..................FMTARWSHLPYDFLGLVSNRIINEIDGISRVVYDISGKPPATIEWE
GUAA_PSYIN       VMGDCRKYDWVVSLRCVETID..................FMTARWSHLPYDLIGHVSNRIINEIDGISRVVYDVSGKPPATIEWE
GUAA_PSEHT       VMGDARKYDWVVSLRCVETID..................FMTARWSHLPYEFLGVVSNRIINEIDGISRVVYDISGKPPATIEWE
GUAA_PSEA6       VMGDARKYDWVVSLRAVETID..................FMTAHWAHLPYEFLGKVSNRIINEIDGISRVVYDISGKPPATIEWE
GUAA_ALTMD       VMGDARKYDWVVSLRAVETID..................FMTAHWAHLPYDFLGKVSNRIINEIDGISRVVYDISGKPPATIEWE
GUAA_SHESW       VMGDGRKYDWVVSLRAVETID..................FMTAHWAHLPYDFLGRVSNRIINEVDGISRVVYDISGKPPATIEWE
GUAA_SHEPC       VMGDGRKYDWVVSLRAVETID..................FMTAHWAHLPYDFLGRVSNRIINEVDGISRVVYDISGKPPATIEWE
GUAA_SHEB9       VMGDGRKYDWVVSLRAVETVD..................FMTAHWAHLPYDFLGRVSNRIINEVDGISRVVYDISGKPPATIEWE
GUAA_SHEB8       VMGDGRKYDWVVSLRAVETVD..................FMTAHWAHLPYDFLGRVSNRIINEVDGISRVVYDISGKPPATIEWE
GUAA_SHEB5       VMGDGRKYDWVVSLRAVETVD..................FMTAHWAHLPYDFLGRVSNRIINEVDGISRVVYDISGKPPATIEWE
GUAA_SHEB2       VMGDGRKYDWVVSLRAVETVD..................FMTAHWAHLPYDFLGRVSNRIINEVDGISRVVYDISGKPPATIEWE
GUAA_SHESM       VMGDGRKYDWVVSLRAVETID..................FMTAHWAHLPYDFLGRVSNRIINEIDGISRVVYDISGKPPATIEWE
GUAA_SHESA       VMGDGRKYDWVVSLRAVETID..................FMTAHWAHLPYDFLGRVSNRIINEIDGISRVVYDISGKPPATIEWE
GUAA_SHESR       VMGDGRKYDWVVSLRAVETID..................FMTAHWAHLPYDFLGRVSNRIINEIDGISRVVYDISGKPPATIEWE
GUAA_SHEON       VMGDGRKYDWVVSLRAVETID..................FMTAHWAHLPYDFLGRVSNRIINEVDGISRVVYDISGKPPATIEWE
GUAA_SHEDO       VMGDGRKYDWVVSLRAVETID..................FMTAHWAHLPYDFLGRVSNRIINEVDGISRVVYDISGKPPATIEWE
GUAA_SHEPW       VMGDGRKYDWVVSLRAVETID..................FMTAHWAHLPYDFLGRVSNRIINEIDGISRVVYDISGKPPATIEWE
GUAA_SHESH       VMGDGRKYDWVVSLRAVETID..................FMTAHWAHLPYDFLGRVSNRIINEIDGISRVVYDISGKPPATIEWE
GUAA_SHEWM       VMGDGRKYDWVVSLRAVETID..................FMTAHWAHLPYDFLGRVSNRIINEIDGISRVVYDISGKPPATIEWE
GUAA_SHEHH       VMGDGRKYDWVVSLRAVETID..................FMTAHWAHLPYDFLGRVSNRIINEVNGISRVVYDISGKPPATIEWE
GUAA_SHEPA       VMGDGRKYDWVVSLRAVETID..................FMTAHWAHLPYDFLGRVSNRIINEVDGISRVVYDISGKPPATIEWE
GUAA_SHEFN       VMGDGRKYDWVVSLRAVETID..................FMTAHWAHLPYDFLGRVSNRIINEVDGISRVVYDISGKPPATIEWE
GUAA_SHELP       VMGDGRKYDWVVSLRAVETID..................FMTAHWAHLPYDFLGRVSNRIINEIDGISRVVYDISGKPPATIEWE
GUAA_SHEAM       VMGDGRKYDWVVSLRAVETID..................FMTAHWAHLPYDFLGRVSNRIINEVDGISRVVYDISGKPPATIEWE
GUAA_IDILO       VMGDQRKYDWVIAIRAVETID..................FMTARWAHLPYELLEKVSNRIINEVNGISRVTYDISGKPPATIEWE
GUAA_ACTSZ       VMGDGRKYDWVVSLRAVETID..................FMTAHWAQLPYDLLGKISNRIINEVDGISRVVYDVSGKPPATIEWE
GUAA_MANSM       VMGDGRKYDWVVSLRAVETID..................FMTAHWAHLPYDLLGKISNRIINEVDGISRVVYDVSGKPPATIEWE
GUAA_ACTPJ       VMGDGRKYDWVVSLRAVETID..................FMTAHWAHLPYDLLGKISNRIINEVNGISRVVYDVSGKPPATIEWE
GUAA_ACTP7       VMGDGRKYDWVVSLRAVETID..................FMTAHWAHLPYDLLGKISNRIINEVNGISRVVYDVSGKPPATIEWE
GUAA_ACTP2       VMGDGRKYDWVVSLRAVETID..................FMTAHWAHLPYDLLGKISNRIINEVNGISRVVYDVSGKPPATIEWE
GUAA_HAEPS       VMGDGRKYDWAVSLRAVETID..................FMTAHWAHLPYDLLGKISNRIINEVNGISRVVYDVSGKPPATIEWE
GUAA_PASMU       VMGDGRKYDWVVSLRAVETID..................FMTAHWAHLPYDLLGKISNRIINEVNGISRVVYDVSGKPPATIEWE
GUAA_HAEDU       VMGDGRKYDWVVSLRAVETID..................FMTAHWAHLPYELLGKISNRIINEVNGISRVVYDVSGKPPATIEWE
GUAA_HISS1       VMGDGRKYDWVISLRAVETID..................FMTAHWANLPYDLLGKISNRIINEVNSISRVVYDISGKPPATIEWE
GUAA_HISS2       VMGDGRKYDWVISLRAVETID..................FMTAHWANLPYDLLGKISNRIINEVNGISRVVYDISGKPPATIEWE
GUAA_HAEI8       VMGDGRKYDWVISLRAVETID..................FMTAHWAHLPYDLLGKVSNRIINEVNGISRVVYDISGKPPATIEWE
GUAA_HAEIG       VMGDGRKYDWVISLRAVETID..................FMTAHWAHLPYDLLGKVSNRIINEVNGISRVVYDISGKPPATIEWE
GUAA_HAEIN       VMGDGRKYDWVISLRAVETID..................FMTAHWAHLPYDLLGKVSNRIINEVNGISRVVYDISGKPPATIEWE
GUAA_HAEIE       VMGDGRKYDWVISLRAVETID..................FMTAHWAHLPYDLLGKVSNRIINEVNGISRVVYDISGKPPATIEWE
GUAA_HAMD5       VMGDGRKYEWVIALRAIETVD..................FMTAHWAELPYDLLGRVSNRIINEVKGISRVVYDISGKPPATIEWE
GUAA_XANC5       VVGDARAYEWVIALRAVETID..................FMTAHWAHLPYDFLGTVSNRIINELRGVSRVVYDISGKPPATIEWE
GUAA_XANAC       VVGDARAYEWVIALRAVETID..................FMTAHWAHLPYDFLGTVSNRIINELRGVSRVVYDISGKPPATIEWE
GUAA_XANCP       VVGDARAYEWVIALRAVETID..................FMTAHWAHLPYDFLGTVSNRIINELRGVSRVVYDISGKPPATIEWE
GUAA_XANCB       VVGDARAYEWVIALRAVETID..................FMTAHWAHLPYDFLGTVSNRIINELRGVSRVVYDISGKPPATIEWE
GUAA_XANC8       VVGDARAYEWVIALRAVETID..................FMTAHWAHLPYDFLGTVSNRIINELRGVSRVVYDISGKPPATIEWE
GUAA_XANOR       VVGDARAYEWVIALRAVETID..................FMTAHWAHLPYDFLGTVSNRIINELRGVSRVVYDISGKPPATIEWE
GUAA_XANOM       VVGDARAYEWVIALRAVETID..................FMTAHWAHLPYDFLGTVSNRIINELRGVSRVVYDISGKPPATIEWE
GUAA_XANOP       VVGDARAYEWVIALRAVETID..................FMTAHWAHLPYDFLGTVSNRIINELRGVSRVVYDISGKPPATIEWE
GUAA_STRMK       VVGDARAYEWVIALRAVETID..................FMTAHWAHLPYEFLGTVSNRIINELRGVSRVVYDISGKPPATIEWE
GUAA_STRM5       VVGDARAYEWVIALRAVETID..................FMTAHWAHLPYEFLGTVSNRIINELRGVSRVVYDISGKPPATIEWE
GUAA_XYLFT       VVGDARAYEWVIALRAVETVD..................FMTAHWAPLPYDFLSTVSNRIINELRGVSRVVYDISGKPPATIEWE
GUAA_XYLF2       VVGDARAYEWVIALRAVETVD..................FMTAHWAPLPYDFLSTVSNRIINELRGVSRVVYDISGKPPATIEWE
GUAA_XYLFM       VVGDARAYEWVIALRAVETVD..................FMTAHWAPLPYDFLSTVSNRIINELRGVSRVVYDISGKPPATIEWE
GUAA_XYLFA       VVGDARAYEWVIALRAVETVD..................FMTAQWAPLPYDFLSTVSNRIINELRGVSRVVYDISGKPPATIEWE
GUAA_CELJU       VVGDGRRYEWVISLRAVETVD..................FMTARWAHLPYELLEKVSNRIINEISGVSRVCYDVSSKPPATIEWE
GUAA_TERTT       VVGDARRYEWVISLRAVETVD..................FMTARWAHLPYDLLEKVSNRIINEISGVSRVAYDVSSKPPATIEWE
GUAA_HAHCH       VVGDARRYEYVIALRAVETID..................FMTARWAHLPYELLEKISNRIINEISGVSRVVYDVSSKPPATIEWE
GUAA_MARAV       VVGDARRYEYVIAIRAVETVD..................FMTARWARLPYELLETVSNRIINEISGVSRVTYDISSKPPATIEWE
GUAA_PSESM       VVGDGRRYAWVVALRAVETID..................FMTARWAHLPYELLETVSGRIINEIEGISRVTYDVSSKPPATIEWE
GUAA_PSE14       VVGDGRRYAWVVALRAVETID..................FMTARWAHLPYELLETVSGRIINEIEGISRVTYDVSSKPPATIEWE
GUAA_PSEU2       VVGDGRRYAWVVALRAVETID..................FMTARWAHLPYELLETVSGRIINEIEGISRVTYDVSSKPPATIEWE
GUAA_PSEFS       VVGDGRRYAWVVALRAVETID..................FMTARWAHLPYELLETVSGRIINEIEGISRVTYDVSSKPPATIEWE
GUAA_PSEF5       VVGDGRRYAWVVALRAVETID..................FMTARWAHLPYELLETVSGRIINEIEGISRVTYDVSSKPPATIEWE
GUAA_PSEPF       VVGDGRRYAWVVALRAVETID..................FMTARWAHLPYELLETVSGRIINEIEGISRVTYDVSSKPPATIEWE
```

```
GUAA_PSEPK    VVGDGRRYAWVVALRAVETVD..................FMTARWAHLPYELLETVSGRIINEIDGISRVTYDVSSKPPATIEWE
GUAA_PSEP1    VVGDGRRYAWVVALRAVETVD..................FMTARWAHLPYELLETVSGRIINEIDGISRVTYDVSSKPPATIEWE
GUAA_PSEPG    VVGDGRRYAWVVALRAVETVD..................FMTARWAHLPYELLETVSGRIINEIDGISRVTYDVSSKPPATIEWE
GUAA_PSEE4    VVGDGRRYAWVVALRAVETVD..................FMTARWAHLPYELLETVSGRIINEIDGISRVTYDVSSKPPATIEWE
GUAA_PSEPW    VVGDGRRYAWVVALRAVETVD..................FMTARWAHLPYDLLETVSGRIINEIDGISRVTYDVSSKPPATIEWE
GUAA_PSEMY    VVGDGRRYAWVVALRAVETVD..................FMTARWAHLPYELLETVSGRIINEIEGISRVTYDVSSKPPATIEWE
GUAA_PSEAE    VVGDGRRYAWVVALRAVETID..................FMTARWAHLPYELLEKVSNRIINEIAGISRVTYDVSSKPPATIEWE
GUAA_PSEA8    VVGDGRRYAWVVALRAVETID..................FMTARWAHLPYELLEKVSNRIINEIAGISRVTYDVSSKPPATIEWE
GUAA_PSEAB    VVGDGRRYAWVVALRAVETID..................FMTARWAHLPYELLEKVSNRIINEIAGISRVTYDVSSKPPATIEWE
GUAA_MARMS    VVGDGRRYEYVVALRAVETID..................FMTARWAHLPYELLEKVSGRIINEIEHISRVTYDVSSKPPATIEWE
GUAA_ALCBS    VVGDARRYEYVVALRAVETID..................FMTARWAHLPYDFLELVSSRIINEIPGISRVTYDISSKPPATIEWE
GUAA_THICR    VVGDARRYDYVVSLRAVETID..................FMTARWAHLPYDFLEKVSNRIINEIPRITRVTYDISSKPPATIEWE
GUAA_ALKEH    VTGDGRRYEYVVALRAVETID..................FMTARWAHLPYEFLDHVSRRIINEIPGISRVAYDISGKPPATIEWE
GUAA_METCA    VMGDGRRYDYVVALRAVETID..................FMTARWAHLPYDFLDLVSRRIINEIPGISRVTYDISGKPPATIEWE
GUAA_NITOC    VMGDGRCYDFVVALRAVESVD..................FMTARWAHLPYDFLDHVSRRIINEVAGISRVTYDISGKPPATIEWE
GUAA_LEGPL    VMGDGRRYDYVICLRAVETVD..................FMTAHWSQLPWDFLGKVSNRIINEVEGVSRVTYDISGKPPATIEWE
GUAA_LEGPA    VMGDGRRYDYVICLRAVETVD..................FMTAHWSQLPWDFLGKVSNRIINEVEGVSRVTYDISGKPPATIEWE
GUAA_LEGPC    VMGDGRRYDYVICLRAVETVD..................FMTAHWSQLPWDFLGKVSNRIINEVEGVSRVTYDISGKPPATIEWE
GUAA_LEGPH    VMGDGRRYDYVICLRAVETVD..................FMTAHWSQLPWDFLGKVSNRIINEVEGVSRVTYDISGKPPATIEWE
GUAA_ACIAD    VVGDGRRYAWVIALRAVETVD..................FMTARFAHLPYELVDKISTRIMNEIKDVSRVVYDVSSKPPATIEWE
GUAA_PSYA2    VVGDGRRYAWVIALRAVETVD..................FMTARFAHLPYDLIETVSNRIMNEIAEVSRVTYDVSSKPPATIEWE
GUAA_RUTMC    ITGDERRYDYVIALRAVETID..................FMTARWARLPYDFLDLVSNRIMNEISRISRVVYDVSGKPPATIEWE
GUAA_FRAP2    VVGDQRRYEYVIALRAVVSID..................FMTATWANLPYDFLSIVSNRIVNEVKQVSRVVYDVTGKPPGTIEWE
GUAA_BURPP    VMGDGRTYEYVVALRAVQTLD..................FMTAHWAHLPHELLGHVSNRIINEVRGINRVVYDISGKPPATIEWE
GUAA_BURXL    VMGDGRTYEYVVALRAVQTLD..................FMTAHWAHLPHELLGHVSNRIINEVRGINRVVYDISGKPPATIEWE
GUAA_BURP8    VMGDGRTYEYVVALRAVQTLD..................FMTAHWAHLPHDLLGHVSNRIINEVRGINRVVYDISGKPPATIEWE
GUAA_POLNS    VMGDGRTYEYVVALRAVQTQD..................FMTAHWAHLPHELLGKVFNRIINEVRGINRVVYDISGKPPATIEWE
GUAA_POLSQ    VMGDGRTYEYVVALRAVQTQD..................FMTAHWAHLPHELLGKVSNRIINEVRGINRVVYDISGKPPATIEWE
GUAA_DECAR    VMGDGRTYENVVALRAVVTSD..................FMTAHWAHLPYELLGRVSNRIINEVRGLNRVVYDVSGKPPATIEWE
GUAA_HERAR    VMGDGRTYEYVVALRAVQTQD..................FMTAHWAHLPYELLGKVSNRIINEVRGINRVVYDISGKPPATIEWE
GUAA_JANMA    VMGDGRTYEYVVALRAVQTQD..................FMTAHWAHLPHELLGNVSNRIINEVRGINRVVYDISGKPPATIEWE
GUAA_AROAE    VMGDGRTYEYVVALRAVETQD..................FMTAHWAELPHSLLGKVSNRIINEVRGINRVVYDISGKPPATIEWE
GUAA_AZOSB    VMGDGRTYEYVVALRAVQTQD..................FMTAHWAELPHSLLGKVSNRIINEVRGINRVVYDISGKPPATIEWE
GUAA_METFK    VMGDGRTYDYVVALRAVVTTD..................FMTAHWAELPYDLLGKVSNRIINEVRGINRVVYDISGKPPATIEWE
GUAA_THIDA    VMGDGRTYDYVVALRAVQTQD..................FMTAHWAELPYTLLGKVSNRIINEVRGINRVVYDVSGKPPATIEWE
GUAA_NITEC    VMGDNRSYEYVIALRAVQTED..................FMTAQWAELPYTLLARISNRIINEIRGINRVVYDISGKPPATIEWE
GUAA_NITEU    VMGDNRSYEYVVALRAVQTED..................FMTAHWAELPYTLLSRISNRIINEIRGINRVVYDISGKPPATIEWE
GUAA_NITMU    VMGDGRTYDFVVALRAVQTED..................FMTARWAELPYALLARISSRIVNEVRGINRVVYDISGKPPATIEWE
GUAA_NEIMA    VMGDGRTYDYVVALRAVITSD..................FMTAHWAELPYSLLGRVSNRIINEVKGINRVVYDVSGKPPATIEWE
GUAA_NEIMF    VMGDGRTYDYVVALRAVITSD..................FMTAHWAELPYSLLGRVSNRIINEVKGINRVVYDVSGKPPATIEWE
GUAA_NEIG1    VMGDGRTYDYVVALRAVITSD..................FMTAHWAELPYSLLGRVSNRIINEVKGINRVVYDVSGKPPATIEWE
GUAA_NEIG2    VMGDGRTYDYVVALRAVITSD..................FMTAHWAELPYSLLGRVSNRIINEVKGINRVVYDVSGKPPATIEWE
GUAA_NEIM0    VMGDGRTYDYVVALRAVITSD..................FMTAHWAELPYSLLGRVSNRIINEVKGINRVVYDVSGKPPATIEWE
GUAA_NEIMB    VMGDGRTYDYVIALRAVITSD..................FMTAHWAELPYSLLGKVSNRIINEVKGINRVVYDVSGKPPATIEWE
GUAA_GEOMG    VMGDGRTYEYVVALRAVETKD..................FMTAGWYPLPYEDMARISSRIINEVKGVNRVVYDISSKPPATIEWE
GUAA_GEOSL    VMGDGRTYEYVIALRAVETKD..................FMTAGWYPLPYEDMARISGRIINEVKGVNRVVYDISSKPPATIEWE
GUAA_GEODF    VMGDGRTYEYVVALRAVETKD..................FMTAGWYPLPYEDLARISSRIINEVKGINRVVYDISSKPPATIEWE
GUAA_PELPD    VMGDGRTYEYVVALRAVETKD..................FMTASWYPLPYEDMARISARIINEVKGVNRVVYDISSKPPATIEWE
GUAA_PELCD    VMGDGRTYEYVIALRAVETKD..................FMTAGWYPLPYADMAHISGRIINEVKGVNRVVYDISSKPPATIEWE
GUAA_BARHE    VMGDGRTYEFVCALRAVTSVD..................GMSADFYPYDMEFLSKTAARIINEVRGINRVVYDVTSKPPGTIEWE
GUAA_BARQU    VMGDGRTYEFVCALRAVTSVD..................GMTADFYPYDMEFLSKTAARIINEVRGINRVVYDITSKPPGTIEWE
GUAA_BARBK    VMGDGRTYEFVCALRAVTSVD..................GMTADFYPHDMDFLSRTAARIINEVRGINRVVYDITSKPPGTIEWE
GUAA_RHIE6    VMGDGRTYEFVCALRAVTSVD..................GMTADFYHYDMEFLGRAATRIINEVRGINRVVYDVTSKPPGTIEWE
GUAA_RHILW    VMGDGRTYEFVCALRAVTSVD..................GMTADFYHYDMEFLGRAATRIINEVRGINRVVYDVTSKPPGTIEWE
GUAA_RHIEC    VMGDGRTYEFVCALRAVTSVD..................GMTADFYHYDMEFLGRAATRIINEVRGINRVVYDVTSKPPGTIEWE
GUAA_RHIL3    VMGDGRTYEFVCALRAVTSVD..................GMTADFYHYDMEFLGRAATRIINEVRGINRVVYDVTSKPPGTIEWE
GUAA_AGRRK    VMGDGRTYEFVCALRAVTSVD..................GMTADFYHYDMEFLGRAATRIINEVRGINRVVYDVTSKPPGTIEWE
GUAA_RHISN    VMGDGRTYEFVCALRAVTSVD..................GMTADFYHYDMEFLGRAATRIINEVRGINRVVYDVTSKPPGTIEWE
GUAA_RHIME    VMGDGRTYEFVCALRAVTSVD..................GMTADFYHYDMEFLGRAATRIINEVRGINRVVYDVTSKPPGTIEWE
GUAA_SINMW    VMGDGRTYEFVCALRAVTSVD..................GMTADFYHYDMEFLGRAATRIINEVRGINRVVYDVTSKPPGTIEWE
GUAA_AGRVS    VMGDGRTYEFVCALRAVTSVD..................GMTADFYHYDMEFLGRAATRIINEVRGINRVVYDVTSKPPGTIEWE
GUAA_BRUSI    VMGDGRTYEFVCALRAVTSVD..................GMTADFYHYDMNFLGNAATRIINEVRGINRVVYDVTSKPPGTIEWE
GUAA_BRUAB    VMGDGRTYEFVCALRAVTSVD..................GMTADFYHYDMNFLGNAATRIINEVRGINRVVYDVTSKPPGTIEWE
GUAA_BRUA2    VMGDGRTYEFVCALRAVTSVD..................GMTADFYHYDMNFLGNAATRIINEVRGINRVVYDVTSKPPGTIEWE
GUAA_BRUA1    VMGDGRTYEFVCALRAVTSVD..................GMTADFYHYDMNFLGNAATRIINEVRGINRVVYDVTSKPPGTIEWE
GUAA_BRUO2    VMGDGRTYEFVCALRAVTSVD..................GMTADFYHYDMNFLGNAATRIINEVRGINRVVYDVTSKPPGTIEWE
GUAA_BRUSU    VMGDGRTYEFVCALRAVTSVD..................GMTADFYHYDMNFLGNAATRIINEVRGINRVVYDVTSKPPGTIEWE
GUAA_BRUC2    VMGDGRTYEFVCALRAVTSVD..................GMTADFYHYDMNFLGNAATRIINEVRGINRVVYDVTSKPPGTIEWE
GUAA_BRUMB    VMGDGRTYEFVCALRAVTSVD..................GMTADFYHYDMNFLGNAATRIINEVRGINRVVYDVTSKPPGTIEWE
GUAA_RHILO    VMGDGRTYEFVCALRAVTSVD..................GMTADFYHYDMAFLGAAATRIINEVRGINRVVYDVTSKPPGTIEWE
GUAA_AGRT5    VMGDYRTYDFVCALRAVTSVD..................GMTADFYPYDMNFLGRAATRIINEVRGINRVVYDVTSKPPGTIEWE
GUAA_BRADU    VMGDGRTYDYVVGLRAVTSTD..................GMTADFYQFDMKFLGETATRIINEVKGVNRVVYDVTSKPPGTIEWE
GUAA_BRASO    VMGDGRTYDYVVGLRAVTSTD..................GMTADFYPFDMKFLGNTATRIINEVKGVNRVVYDVTSKPPGTIEWE
GUAA_OLICO    VMGDGRTYEFVVGLRAVTSTD..................GMTADFYPFDMRFLGETATRIINEVKGVNRVVYDVTSKPPGTIEWE
GUAA_RUEST    VMGDGRTYDYACALRAVTSVD..................GMTADYYPFSHEFLGETATRIINEVKGINRCTYDITSKPPGTIEWE
GUAA_RUEPO    VMGDGRTYDYACALRAVTSVD..................GMTADYYPFTHEFLGETATRIINEVKGINRCTYDITSKPPGTIEWE
GUAA_CAUCR    VMGDARTYENVLALRAVTSTD..................GMTADFFEFPWPVLGKTATRIINEVRGVNRVVYDVTSKPPGTIEWE
GUAA_CAUCN    VMGDARTYENVLALRAVTSTD..................GMTADFFEFPWPVLGKTATRIINEVRGVNRVVYDVTSKPPGTIEWE
GUAA_PHEZH    VMGDARTYDEVCALRAVTSTD..................GMTADFFEFPWEVLSRAATRIINEVRGINRVVYDVTSKPPGTIEWE
GUAA_RHORT    VMGDARSYDFALALRAVTSTD..................GMTADYYPFDHTFLGRVANRIINEVKGVNRVVYDITSKPPGTIEWE
GUAA_GLUOX    VMGDGRTYDQACALRAVTSTD..................GMTAEVYPFDFAFLNRVAGRIVNEVRGINRVTYDITSKPPGTIEWE
GUAA_RHOBA    VMGDARTYDNAVAVRCVNTDD..................FMTADWSHLPYELLARISTRIINEVKGVNRVCYDISSKPPATIEWE
GUAA_BACFR    VMGDERTYERAVALRAVTSTD..................AMTADWAHLPYEFLGKVSNDIINKVKGVNRVTYDISSKPPATIEWE
GUAA_BACFN    VMGDERTYERAVALRAVTSTD..................AMTADWAHLPYEFLGKVSNDIINKVKGVNRVTYDISSKPPATIEWE
GUAA1_BACTN   VMGDERTYERAVALRAVTSTD..................AMTADWAHLPYEFLGKISNDIINKVKGVNRVTYDISSKPPATIEWE
GUAA_CYTH3    VMGDERTYENVVALRAVSSMD..................GMTADWIHLPYDFLADISNEIINRVKGVNRVVYDISSKPPATIEWE
GUAA_PELLD    VMGDKRTYENVLALRAVESTD..................GMTADWAHLPHDFLAKVSNRIINEVRGINRVAYDISSKPPATIEWE
GUAA_PROVI    VMGDKRTYENVLALRAVESTD..................GMTADWAHLPHDFLAKVSNRIINEVRGINRVAYDISSKPPATIEWE
GUAA_CHLL2    VMGDKRTYENVLALRAVESSD..................GMTADWAQLPHDFLARVSNRIINEVRGINRVAYDISSKPPATIEWE
GUAA_CHLPD    VMGDKRTYENVLALRAVESTD..................GMTADWAQLPHDFLARVSNRIINEVRGINRVAYDISSKPPATIEWE
GUAA_PELPB    VMGDKRTYENVLALRAVESSD..................GMTADWAQLPHDFLARVSNRIINEVRGINRVAYDISSKPPATIEWE
GUAA_CHLTE    VMGDKRTYENVLALRAVESTD..................GMTADWAHLPHDFLAKVSNRIINEVRGINRVVYDISSKPPATIEWE
GUAA_CHLCH    VMGDKRTYENVLALRAVESSD..................GMTADWAPLPHDFLARVSNRIINEVRGINRVAYDISSKPPATIEWE
GUAA_CHLPB    VMGDKRTYENVLALRAVESSD..................GMTADWAHLPHEFLSRVSNRIINEVRGINRVAYDISSKPPATIEWE
GUAA_CHLT3    VMGDNRTYENAVALRAVNSTD..................GMTADWARLPNDFLAHVSNRIINEVRGINRVTYDISSKPPATIEWE
GUAA_SALRD    VMGDERTYENVCALRAVTSVD..................GMTADWAHLPHDFLGHVSNRIVNEVPGINRIVYDVSSKPPATIEWE
GUAA_GEMAT    VMGDGRTYENVVALRAVTSTD..................GMTADWYPFPYEVLGRISNRIINEVDGVNRVVYDISSKPPATIEWE
GUAA_KLULA    VMGDQRTYEQVIALRAIETTD..................FMTADWYPFEHSFLKKVASRIVNEVDGVARVTYDITSKPPATVEWE
GUAA_ASHGO    VMGDQRTYDQVIALRAIETTD..................FMTADWYPFEHEFLKHVASRIVNEVEGVARVTYDITSKPPATVEWE
GUAA_YEAST    VMGDQRTYDQVIALRAIETTD..................FMTADWFPFEHSFLKKVASRIVNEVDGVARVTYDITSKPPATVEWE
GUAA_CANGA    VMGDQRTYEQVIALRAIETTD..................FMTADWYPFEHSFLRKVASRIVNEVDGVARVTYDITSKPPATVEWE
GUAA_DEBHA    VMGDQRTYEQVIALRAIETTD..................FMTADWFVFEAAFLKKTASRIVNEVDGVARVTYDITSKPPATVEWE
GUAA_CANAL    VMGDQRTYEQVIALRAIETLD..................FMTADWFIFEAAFLKKVASRIVNEVDGVARVTYDITSKPPATVEWE
GUAA_YARLI    VMGDQRTYEQVIALRAIETVD..................FMTADWYIFDANFLKTVARRIVNEVPGVARVVYDITSKPPATVEWE
GUAA_PHANO    VMGDKRVHDQVIALRAVETTD..................FMTADWYPFDGEFLKRVSRRIVNEVNGVCRVVYDITSKPPGTIEME
GUAA_EMENI    VMGDKRVYANIILLRAISTKD..................FMTATPYPFSYEFLSKVSTRIVNEVAGVCRVCYDYTSKPPGTIEME
GUAA_ASPOR    VMGDKRVYENIVLLRAVETTD..................FMTAIAYPFEHEFLTRVSTRIVNEVSGVCRVAYDYTSKPPGTIELE
GUAA_ASPFU    VMGDKRVYAEIIILRAVETTDCKLTLGIDGDFVLRYIISVMTARAFPFDNEFLSKCATRIINEVHGVSRVLYDISSKPPATIEME
GUAA_USTMA    VQGDKRTYDQVIALRAAATTD..................FMTATWYPFPAEFLSKVSNRITNEVQGVNRVVLDISSKPPATIEWL
GUAA_DEHM1    VMGDFRTYGYLVAIRAVTSED..................AMTADWAKLPYDLLSVISNRIVNEVKEVNRVVYDISSKPPSTIEWE
GUAA_DEHMC    VMGDFRTYGYLVAIRAVTSED..................AMTADWAKLPYDLLSIISNRIVNEVKEVNRVVYDISSKPPSTIEWE
GUAA_DESPS    VMGDFRTYEHVVALRVVDSRD..................AMTADWSRVPYDILGDISTRIINEVRGVNRVVYDISSKPPATIEWE
GUAA_BRAHW    VMGDGRTYEQVCAVRAVESVD..................AMTADWAKIDYNVLGIISNRIINEVKGINRVVYDISSKPPATIEWE
GUAA_DESVV    VMGDGRTYEHVIALRIVDSVD..................AMTADWARLPSELLERISSRIINEVKGVNRVVYDISSKPPSTIEWE
GUAA_DESVH    VMGDGRTYEHVIALRIVDSVD..................AMTADWARLPSELLERISSRIINEVKGVNRVVYDISSKPPSTIEWE
GUAA_DESVM    VMGDGRTYEHVIALRIVDSVD..................AMTADWARLPSELLERISSRIINEVKGVNRVVYDISSKPPSTIEWE
GUAA_DESAG    VMGDDRTYEHVIALRVVDSVD..................AMTADWVRLPSEVLERISSRIINEVKGVNRVVYDISSKPPSTIEWE
GUAA_DESMR    VMGDGRTYENVAAIRVVDSLD..................AMTADWSRLPSEILAVMSNRIINEVKGVNRVVFDVSSKPPATIEWE
GUAA_DESAD    VMGDDRTYEHVIALRIVDSID..................AMTADWSRIPNDILARMSNRIINEVKGVNRVVLDISSKPPATIEWE
GUAA_CALBD    VMGDERTYDYTVALRAVTSID..................GMTADWAKIPYEILERVSNEIVNSVKKVNRVVYDITSKPPATIEWE
GUAA_CALS8    VMGDERTYDYTVALRAVTSVD..................GMTADWARIPYDVLERVSNEIVNTVRKVNRVVYDITSKPPATIEWE
GUAA_CARHZ    VMGDERTYAYTIALRAVTSED..................GMTADWVRLPYELLEKISARIVGEVKGVNRVVYDITSKPPATIEWE
GUAA_CLOBL    VMGDERTYCHTIALRAVTSSD..................AMTSDWARIPYEVLDKVSRRIVNEVKEVNRIVYDVTSKPPATIEWE
GUAA_CLOBK    VMGDERTYCHTIALRAVTSSD..................AMTSDWARIPYEVLDKVSRRIVNEVKEVNRIVYDVTSKPPATIEWE
GUAA_CLOBM    VMGDERTYCHTIALRAVTSSD..................AMTSDWARIPYEVLDKVSRRIVNEVKEVNRIVYDVTSKPPATIEWE
GUAA_CLOBJ    VMGDERTYCHTIALRAVTSSD..................AMTSDWARIPYEVLDKVSRRIVNEVKEVNRIVYDVTSKPPATIEWE
GUAA_CLOB6    VMGDERTYCHTIALRAVTSSD..................AMTSDWARIPYEVLDKVSRRIVNEVKEVNRIVYDVTSKPPATIEWE
GUAA_CLOBH    VMGDERTYCHTIALRAVTSSD..................AMTSDWARIPYEVLDKVSRRIVNEVKEVNRIVYDVTSKPPATIEWE
GUAA_CLOB1    VMGDERTYCHTIALRAVTSSD..................AMTSDWARIPYEVLDKVSRRIVNEVKEVNRIVYDVTSKPPATIEWE
GUAA_CLONN    VMGDERTYCHTIALRAVTSSD..................AMTSDWARIPYEVLDKVSRRIVNEVKGVNRIVYDVTSKPPATIEWE
GUAA_CLOAB    VMGDGRTYCETIALRAVTSSD..................AMTSDWARIPYEVLDKVSRRIVNEVKGVNRIVYDVTSKPPATIEWE
GUAA_CLOTE    VMGDERTYSHTIGLRAVNSSD..................GMTSDWAKIPYEVLDKVSIRIVNEVKGVNRIVYDITSKPPSTIEWE
GUAA_CLOPE    VMGDERTYCHTVGLRAVTSSD..................GMTSNWAHIPYEVIDKVSRRIVNEVKGVNRIVYDVTSKPPATIEWE
GUAA_CLOB8    VMGDERTYSHTIALRAVTSSD..................AMTSEWARIPYEILDLVSRRIVNEVKGVNRIVYDITSKPPATIEWE
GUAA_ALKMQ    VMGDERTYAHTIGIRAITSSD..................AMTADWARIPFEVLENMSRRIVNEVEGANRIVYDITSKPPSTVEWE
GUAA_CLOTH    VMGDERTYDYTLALRAVTTID..................FMTADWAKIPYDVLEKVSNRIVNEVKHINRIVYDITTKPPATIEWE
GUAA_CLOCE    VMGDERTYDYALALRAVTTTD..................FMTADWARIPYDILEKVSTRIVNEVKQINRIVYDITSKPPATIEWE
GUAA_CLOBA    VMGDERTYDYAIALRAVTTSD..................FMTAESADLPWEVLGKVTTRIVNEVKGVNRVMYDCTGKPPATIEFE
GUAA_PEPD6    VMGDERTYAYLVGLRAVTSSD..................GMTSDWYKMPYDVLERISNRIINEVDGVNRVVYDITSKPPGTIEWE
GUAA_FINM2    VMGDFRTYDYTLALRAVTTTD..................FMTADWARIPYEVLDKTSVRIINEVDHINRIVYDITSKPPATIEWE
GUAA_TREDE    VMGDFRTYDYTLALRAVKTSD..................FMSADWVRIPYEVLDKVSSRIINEVKGINRIVYDITSKPPATIEWE
GUAA_BACCR    VMGDERTYDYTVGIRAVTSID..................GMTADWARIPWDVLEKISVRIVNEVKHVNRIVYDVTSKPPATIEWE
GUAA_BACC4    VMGDERTYDYTVGIRAVTSID..................GMTADWARIPWDVLEKISVRIVNEVKHVNRIVYDVTSKPPATIEWE
GUAA_BACC2    VMGDERTYDYTVGIRAVTSID..................GMTADWARIPWDVLEKISVRIVNEVKHVNRIVYDVTSKPPATIEWE
```

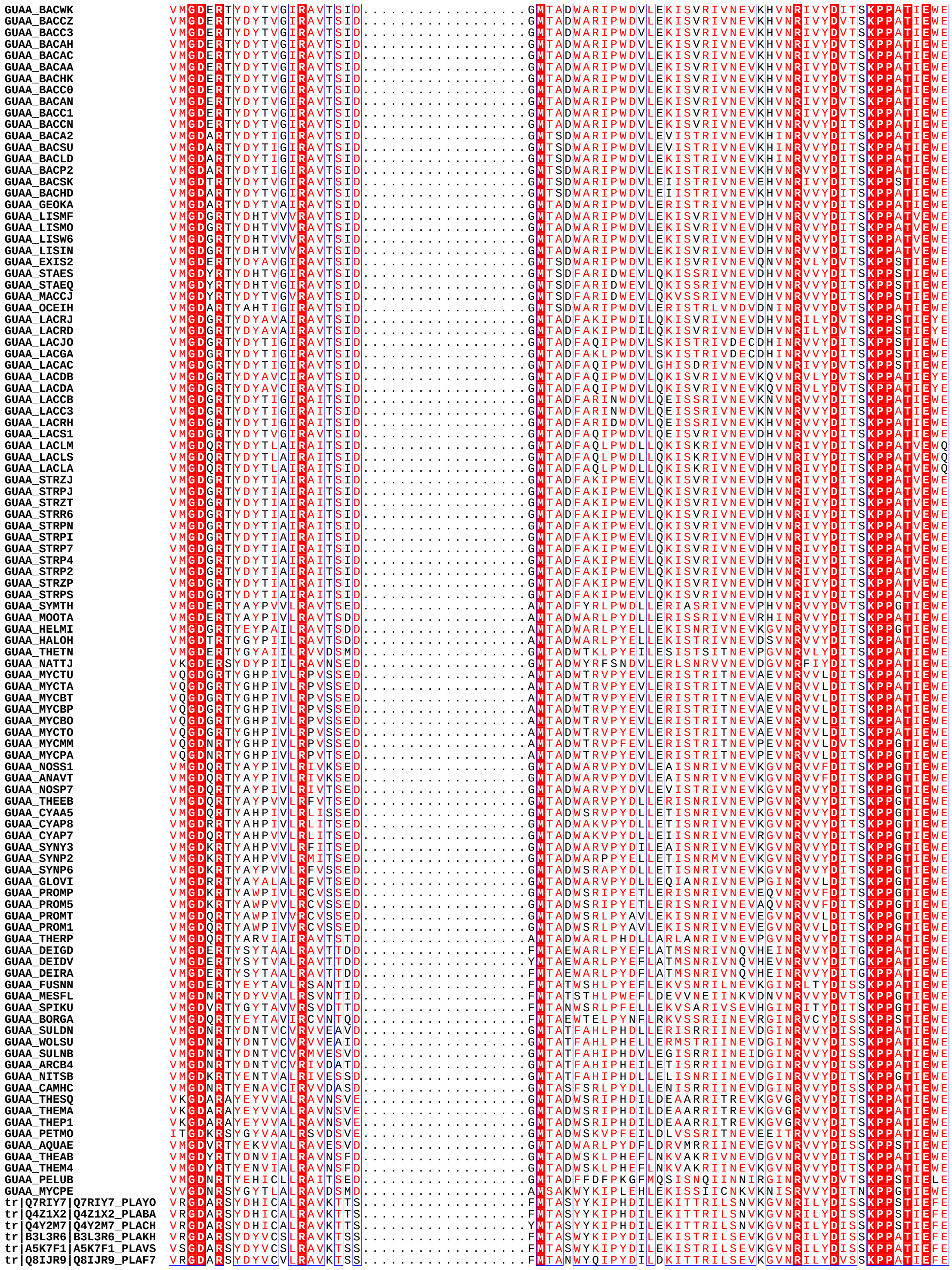

Supplementary Figure 7. Multiple sequence alignment of *Plasmodium* GMPS (Q7RIY7, Q4Z1X2, Q4Y2M7, B3L3R6, A5K7F1 and Q8IJR9) against the Swiss-Prot database. The figure has been generated using the program ESPript (Robert, X. & Gouet, P. Deciphering key features in protein structures with the new ENDscript server.*Nucleic acids research* (2014)).

Supplementary Table 1. Steady-state kinetic parameters for *Pf*GMPS and mutants[a].

Varied substrate		***Pf*GMPS**	***Pf*ATPPase**	***Pf*GATase**[b]	**Y18F**	**H20A**	**W167F**	**N169S**
**Q**	K_m (mM)	0.36 ± 0.03	NA	101 ± 7	0.3 ± 0.01	0.5 ± 0.01	0.52 ± 0.02	0.21 ± 0.004
	k_{cat} (min^{-1})	37 ± 3		ND	31 ± 1	20 ± 0.2	55 ± 5	45 ± 2
	k_{cat}/K_m ($mM^{-1} min^{-1}$)	103		ND	103	40	106	214
**NH_4Cl**	K_m (mM)	19 ±1	7.0 ± 0.3	NA	7 ± 1	8 ± 1	7 ± 1	8 ± 1
	k_{cat} (min^{-1})	55 ± 1	9.3 ± 0.1		39 ± 0.3	26 ± 0.4	58 ± 1	57 ± 1
	k_{cat}/K_m ($mM^{-1} min^{-1}$)	2.9	1.3		5.6	3.3	8.3	7.1
**ATP**	K_m (µM)	102 ± 5	56 ± 16	NA	ND	56 ± 8	ND	ND
	k_{cat} (min^{-1})	43 ± 1	8 ± 1			17 ± 1		
	k_{cat}/K_m ($mM^{-1} min^{-1}$)	422	143			305		
**XMP**	K_m (µM)	10 ± 2	11 ± 4	NA	ND	8 ± 4	ND	ND
	k_{cat} (min^{-1})	44 ± 1	10 ± 1			17 ± 3		
	k_{cat}/K_m ($mM^{-1} min^{-1}$)	4400	909			2083		

[a] In all cases except *Pf*GATase, the assay involved monitoring GMP formation as drop in absorbance at 290 nm. The steady-state kinetic parameters for ATP and XMP for *Pf*GMPS and *Pf*GMPS_H20A concern the Gln-dependent GMP formation while for *Pf*ATPPase the parameters concern the NH_4Cl-dependent activity. The concentration of the fixed substrates were ATP 2 mM, XMP 150 µM, Gln 5 mM, NH_4Cl 100 mM and 15 µg enzyme in a total volume of 250 µl. The reactions were carried out in 90 mM Tris-HCl, pH 8.5, 20 mM $MgCl_2$, 0.1 mM EDTA and 0.1 mM DTT at 25 °C.

[b] In assays with *Pf*GATase, glutamate formation was measured using the *Pf*AAT-coupled assay. The assay mixture consisted of 90 mM Tris-HCl, pH 8.5, 0.1 mM EDTA, 0.1 mM DTT, 1 mM OAA, 20 µg ml^{-1} pyridoxal phosphate (PLP), 26 µg *Pf*AAT and 13 µg of *Pf*GATase in a total reaction volume of 300 µl. The Michaelis-Menten plots for Gln for *Pf*GATase did not saturate even at high substrate concentrations. Hence, k_{cat} values could not be determined and the K_m value is an apparent value.

ND, not determined; NA, not applicable. All data are expressed as mean ± s.e.m. of 2 to 4 independent measurements.

Supplementary Table 2. List of primers.

Mutation	Method	Restriction enzyme site	Primer Sequence
**Y18F**	Single Primer	EcoRI	5'CAAGATTTTGGTATTGAATTTCGGTTCTGAATTCTTCCATTTGATTGTAAAAAGATTAAAC3'<br>5'GATTTTGGTATTGAATTTCGGTTCTCAATTCTTCCATTTGATTGTAAAAAG3'
**H20A**	Double Primer	None	5'GAATTTCGGTTCTCAATACTTCGCTTTGATTGTAAAAAGATTAAAC3'<br>5'GTTTAATCTTTTTACAATCAAAGCGAAGTATTGAGAACCGAAATTC3'
**C113A**	Single Primer	KpnI	5'TAAGAAATCTAAGACATCCGAATATGGAGGTACCGATGTAAACATATTAAGAAATGATAATAT3'<br>5'CTAAGACATCCGAATATGGAGCTACCGATGTAAACATATTAAG3'
**W167F**	Single Primer	KpnI	5'ATTTGAAAATATTAAAAGTGACATTACAACGGTACCGATGAATCATAATGATGAAGTTACAAAAATTCC3'<br>5'GTTTATTTGAAAATATTAAAAGTGACATTACAACGGTATTTATGAATCATAATGATGAAGTTACAAAAATTCCAGAAA3'
**N169S**	Double Primer	None	5'AAGTGACATTACAACGGTTTGGATGAGTCATAATGATGAAGTTACAA3'<br>5'TTGTAACTTCATCATTATGACTCATCCAAACCGTTGTAATGTCACTT3'
**D172A**	Double Primer	NA	5' CGGTTTGGATGAATCATAATGCTGAAGTTACAAAAATTCCAG 3'<br>5' CTGGAATTTTTGTAACTTCAGCATTATGATTCATCCAAACCG 3'
**Y212W**	Double Primer	NA	5'GGTGTTCAATATCATCCAGAGGTGTGGGAATCATTAGATGGAGAATTAATGT3'<br>5'ACATTAATTCTCCATCTAATGATTCCCACACCTCTGGATGATATTGAACACC3'
**D371A**	Double Primer	NA	5'CATTTTTATTACAAGGTACTCTATATCCAGCTATTATTGAAAGTAAATGTTC3'<br>5'GAACATTTACTTTCAATAATAGCTGGATATAGAGTACCTTGTAATAAAAATG3'
**E374L**	Double Primer	NA	5'AAACATTTTTATTACAAGGTACTCTATATCCAGATATTATTTTAAGTAAATGTTCAAAAAATTTATCAGATACTA3'<br>5'TAGTATCTGATAAATTTTTTGAACATTTACTTAAAATAATATCTGGATATAGAGTACCTTGTAATAAAAATGTTT3'
***Pf*GATase**	NA		5'GTCGCTAGCCATCACCATCACCATCACGGA3'<br>5'ACGTGAGCTCTCATGGATCAAATTGTTTTTTACATTT3'
***Pf*ATPPase**	NA		5'GTCGCTAGCATACGTTATCATGAATTAGAATTAAAAAATATTG3'<br>5'GCAGAGCTCTCATTCGAATTCAATCGTTGCTGG3'
